# Structure-Based Optimization of Pyridone α-Ketoamides as Inhibitors of the SARS-CoV-2 Main Protease

**DOI:** 10.1021/acs.jmedchem.4c02172

**Published:** 2025-01-16

**Authors:** Ravi Kumar Akula, Haifa El Kilani, Alina Metzen, Judith Röske, Kaixuan Zhang, Matthias Göhl, Nanaji Arisetti, Graham P. Marsh, Hannah J. Maple, Mark S. Cooper, Burhan Karadogan, Dirk Jochmans, Johan Neyts, Katharina Rox, Rolf Hilgenfeld, Mark Brönstrup

**Affiliations:** † Department of Chemical Biology, Helmholtz Centre for Infection Research, Inhoffenstr. 7, Braunschweig 38124, Germany; ‡ Institute of Molecular Medicine, University of Lübeck, Ratzeburger Allee 160, Lübeck 23562, Germany; § German Center for Infection Research (DZIF), Hannover-Braunschweig Site, Braunschweig 38124, Germany; ∥ German Center for Infection Research (DZIF), Hamburg-Lübeck-Borstel-Riems Site, Lübeck 23562, Germany; ⊥ Bio-Techne (Tocris), Bristol BS11 9QD, U.K.; # Department of Microbiology, Immunology and Transplantation, Rega Institute, KU Leuven, Leuven 3000, Belgium; ¶ Institute of Organic Chemistry and Biomolecular Drug Research Centre (BMWZ), Leibniz University Hannover, Schneiderberg 1B, Hannover 30167, Germany

## Abstract

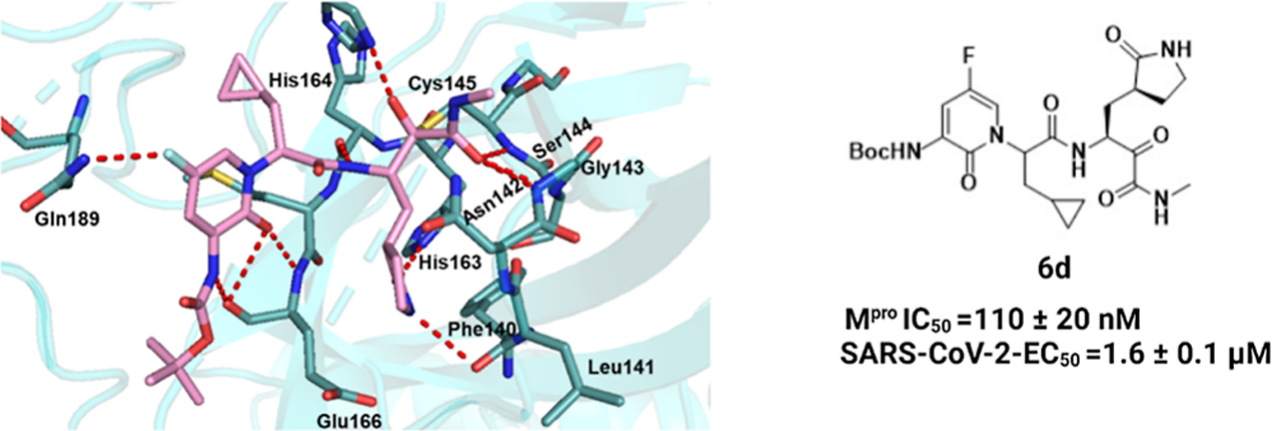

The main protease M^pro^ is a clinically validated target to treat infections by the coronavirus SARS-CoV-2. Among the first reported M^pro^ inhibitors was the peptidomimetic α-ketoamide **13b**, whose cocrystal structure with M^pro^ paved the way for multiple lead-finding studies. We established structure–activity relationships for the **13b** series by modifying residues at the P1′, P3, and P4 sites. Guided by cocrystal structures, we reduced the P1′ substituent size to better fill the pocket and added a fluorine substituent to the pyridone ring, enabling a new hydrogen bond with Gln189 in P3. Among 22 novel analogues, **6d** and **12d** inhibited M^pro^ with IC_50_s of 110 nM and 40 nM, improving the potency of **13b** by up to 9.5-fold. Compound **6d** had pronounced antiviral activity with an EC_50_ of 1.6 μM and was stable in plasma and microsomes. The study illustrates the potential of structure-based design to systematically improve peptidomimetic α-ketoamides.

## Introduction

**Figure 1:**
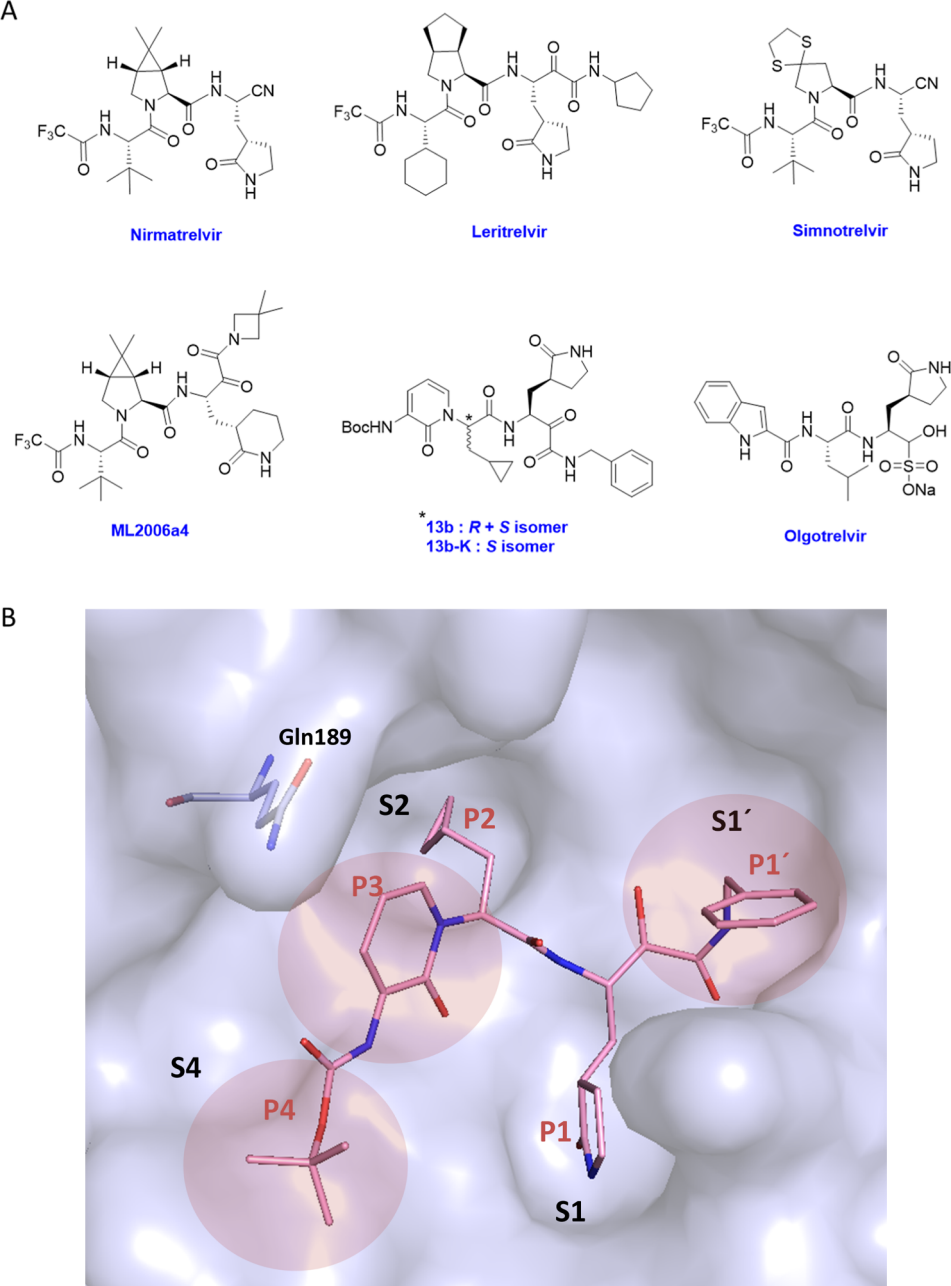
Structures of selected, previously reported M^pro^ inhibitors and design considerations for this study. (A) Structures of selected M^pro^ inhibitors with covalent warheads. (B) Rationale for modifying P1′, P3, and P4 residues based on the cocrystal structure of **13b-K** with M^pro^ (PDB Code: 6Y2G). S1′: reduce the site of P1′ rest to improve pocket fit, S3: add a substituent to enable H-bonding to Gln189, S4: modify P4 rest to benefit from the spacious S4 pocket. **13b-K** is represented as sticks in pink, and the M^pro^ surface is shown in light blue.

The outbreak of the coronavirus SARS-CoV-2 at the end of 2019^1,2^ has led to the pandemic of COVID-19 that affected more than 775 million people, led to more than 7 million deaths (as of August, 2024) and induced massive economic consequences. Vaccines were rapidly developed and approved, and more than 13 billion doses administered helped contain the disease and prevent progression to severe sequelae.^3^ However, escape mutants in vaccine-targeted epitopes have emerged that impair their efficacy, and limited social acceptance and distribution challenges associated with vaccines led to the uniform conclusion that the preventive vaccination measures need to be complemented with treatment options using antiviral drugs. In addition to monoclonal antibodies and repurposed RNA polymerase inhibitors such as remdesivir,^4,5^ the most advanced direct-acting antiviral small-molecule drugs against SARS-CoV-2 are inhibitors of the coronavirus main protease (M^pro^, also known as 3C-like protease, 3CL^pro^, or Nsp5). M^pro^ cleaves the two polyproteins pp1a and pp1ab, which are translated from viral RNA, at 11 conserved sites^6^ and is essential for viral replication and multiplication. It also cleaves host proteins such as NEMO, thereby inducing neurological symptoms and blocking an antiviral interferon λ response.^7,8^ Building on previous knowledge gained after the SARS-CoV outbreak in 2003, the crystal structure of M^pro^ of SARS-CoV-2 with the peptidomimetic α-ketoamide **13b** was published by us in early 2020,^6^ which facilitated the design and optimization of a rich arsenal of inhibitors from 2020 until today (Figure 1).^9−15^ The most advanced M^pro^-targeting drug is nirmatrelvir,^9^ a covalently binding peptidomimetic that gained market authorization in combination with the CYP3A4 inhibitor ritonavir under the tradename Paxlovid. However, drug–drug interaction issues and viral rebound effects associated with Paxlovid demonstrated that a larger arsenal of protease inhibitors is needed in order to counteract the current as well as future coronavirus outbreaks. Advances in this direction are illustrated by noncovalent M^pro^ inhibitors such as ensitrelvir,^16^ 23R,^17^ or GC-14,^18^ as well as covalently binding analogues with α-ketoamide warheads such as^13^ leritrelvir,^14^ which were approved in China, or ML2006a4^15^ (Figure 1).

Compared to many other reactive groups covalently modifying Cys145, the α-ketoamide warhead has an additional interaction with Ser144 in the S1′ site of the enzyme that confers selectivity.^6,14,15^ For the **13b** lead series, we found that an S-configuration at the P2 cyclopropyl amino acid as in **13b-K** (the active (*S*,*S*,*S*)-diastereomer of **13b**)^19^ is favorable, but further structure–activity relationships have not been reported yet. Recent studies have highlighted that various M^pro^ inhibitors, including **13b**, GC376, MG-132, Calpain II, Calpain XII, and the clinical-stage agent olgotrelvir^20^ (Figure 1), also inhibit the human cysteine protease cathepsin L (CTSL) as an off-target.^21^

Here, we describe the synthesis of **13b** analogues with a systematic variation of residues populating the S1′, S3, and S4 sites of M^pro^ and characterize their properties as dual inhibitors of M^pro^ and CTSL by a combination of structural and biophysical studies, enzymatic and antiviral activity assays as well as in vitro ADME and in vivo pharmacokinetic experiments.

## Results

### Design Rationale

The active site of SARS-CoV-2 M^pro^ features a catalytic dyad comprising Cys145 and His41, with Cys145 being covalently bound to the *N*-benzyl-α-ketoamide in **13b**. We noticed that in our cocrystal structures (Protein Data Bank (PDB) codes: 6Y2E, 6Y2F, 6Y2G, 8A4T), the benzyl group does not occupy the small S1′ pocket due to space restrictions but points outside the pocket.^6,19^ We therefore investigated the replacement of the benzyl group with a hydrogen atom or small alkyl groups such as methyl, isopropyl, or cyclopropyl.

The γ-lactam moiety, designed as a surrogate for glutamine residues of proteinogenic substrates, is tightly bound within the S1 pocket through hydrogen bonds to the main-chain oxygen of Phe140 and the Glu166 carboxylate, while its carbonyl oxygen accepts an H-bond from the imidazole of His163. Because this motif has been found as an optimal solution for binding of peptidomimetics to coronavirus M^pro^ or enterovirus 3C^pro^,^22,23^ we kept it and refrained from structural changes. The same was true for the P2 cyclopropylalanine moiety, which fits well into the S2 subsite and resulted from our previous optimization efforts.^6^ The 3-amino-pyridone moiety occupying the P3/P2 position constitutes a key feature of the **13b** structure and reduces the number of hydrogen donors compared to an amide motif.^24^ In order to investigate whether a reduction of electron density in the aromatic ring might strengthen interactions with Glu166, we targeted the synthesis of the corresponding pyrimidone and pyrazinone analogues. Furthermore, we noted that the pyridone ring in **13b** came close to Gln189, with a minimal distance of 4.3 Å between the pyridone and Gln189 Nε2, too far to form a bonding interaction. In order to enable hydrogen bonding with Gln189, we designed a fluorine substitution at the 5-position.

Finally, we noticed that the Boc capping group of **13b** does not fully occupy the S4 pocket. Therefore, broader structural variations using acyl and urea linkers were introduced at this position.

### Compound Synthesis and In Vitro Testing

**Scheme 1:**
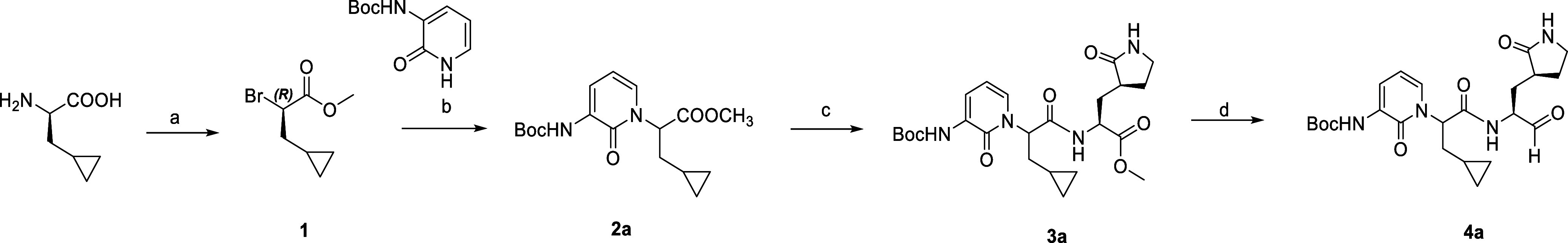
Improved Synthesis of Aldehyde **4a** Reagents and conditions: (a) (i) KBr, H_2_SO_4_, NaNO_2_, 0 °C—rt, 16 h; (ii) SOCl_2_/CH_3_OH, 0 °C—rt, 16 h; 90% over two steps; (b) Cs_2_CO_3_/CH_3_CN, 16 h, 50 °C, 70%; (c) (i) LiOH·H_2_O, aq.CH_3_OH, 0 °C—rt, 1.5 h; (ii) methyl (S)-2-amino-3-((*S*)-2-oxopyrrolidin-3-yl)propanoate hydrochloride, HATU/TEA, DMF, 0 °C—rt, 10 h, 92% over two steps; (d) (i) LiBH_4_, CH_2_Cl_2_, 2 h; (ii) DMP, CH_2_Cl_2_, 2 h, 96% over two steps.

Most target compounds were prepared from the aldehyde **4a** as a central intermediate. Its modified synthesis started with the conversion of (*R*)-cyclopropylalanine to (*R*)-3-cyclopropyl-2-bromopropanoic acid, which was further reacted with SOCl_2_ in methanol to the corresponding methyl ester **1** in a 90% yield over two steps (Scheme 1 and Supporting Information S3). Its *N*-alkylation with the Boc-protected aminopyridone to give **2a** was achieved with Cs_2_CO_3_ in acetonitrile in a yield of 70% after purification. The ester **2a** was hydrolyzed to the carboxylic acid and coupled with methyl-(*S*)-2-amino-3-((*S*)-2-oxopyrrolidin-3-yl)-propanoate using HATU and triethylamine to the tripeptide **3a** in a 92% yield. The ester function of **3a** was first reduced with LiBH_4_ in CH_2_Cl_2_ for 2 h and then oxidized with Dess–Martin periodinane (DMP) in CH_2_Cl_2_ to the intermediate **4a** in a 96% yield over two steps. Compared to the previously published synthesis,^6^ this route has a reduced number of steps, shorter reaction times, and a higher overall yield (55.6% instead of 4.9%).

**Scheme 2:**
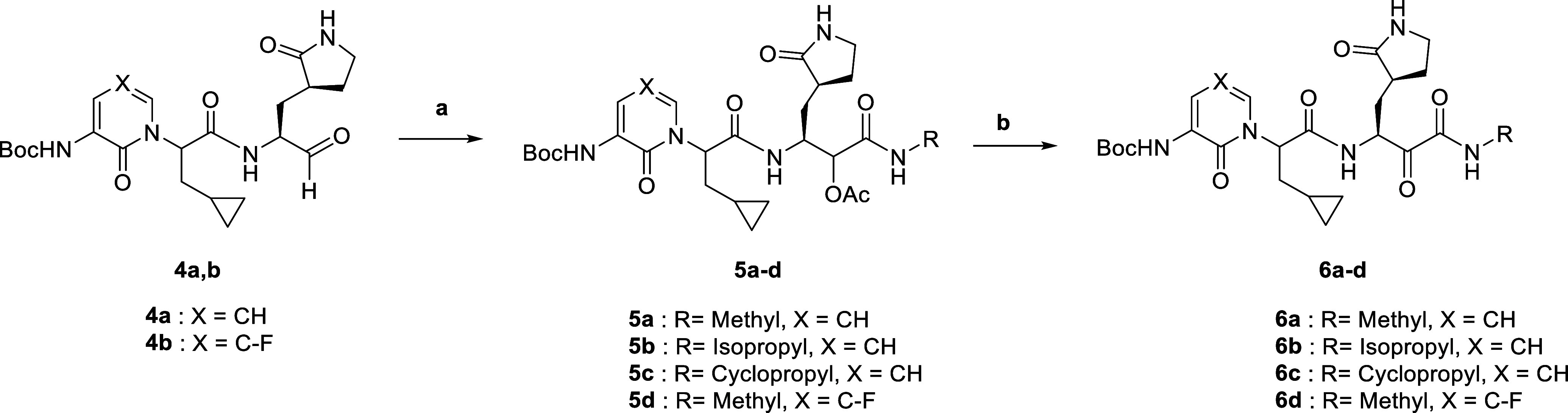
Synthesis of α-Ketoamides **6a–d** Reagents and conditions: (a) alkyl isocyanide, CH_3_COOH, CH_2_Cl_2_, 0 °C—rt, 22 h, 85–92%; (b) (i) LiOH·H_2_O, MeOH, H_2_O, 0 °C—rt, 1.5 h; (ii) DMP, CH_2_Cl_2_, rt, 2 h, 49–63% over two steps.

The preparation of α-ketoamides with varying S1′ residues commenced with a Passerini reaction with **4a**, three alkyl isocyanides, and acetic acid to the α-acetoxy amides **5a–c** (Scheme 2). An ester hydrolysis and a subsequent DMP oxidation converted **5a–c** to the corresponding α-ketoamides **6a–c**, bearing methyl, isopropyl, and cyclopropyl substituents at the amide nitrogen, respectively. We also prepared the fluoro-substituted analogue of α-ketoamide **6a** starting from aldehyde **4b** in a similar manner (Supporting Information S6). After purification by RP-18 HPLC, **6a–d** were obtained as inseparable mixtures of diastereomers because the pyridone moiety facilitated racemization of the α-carbon of the cyclopropyl alanine group under basic conditions.^19^

**Scheme 3:**
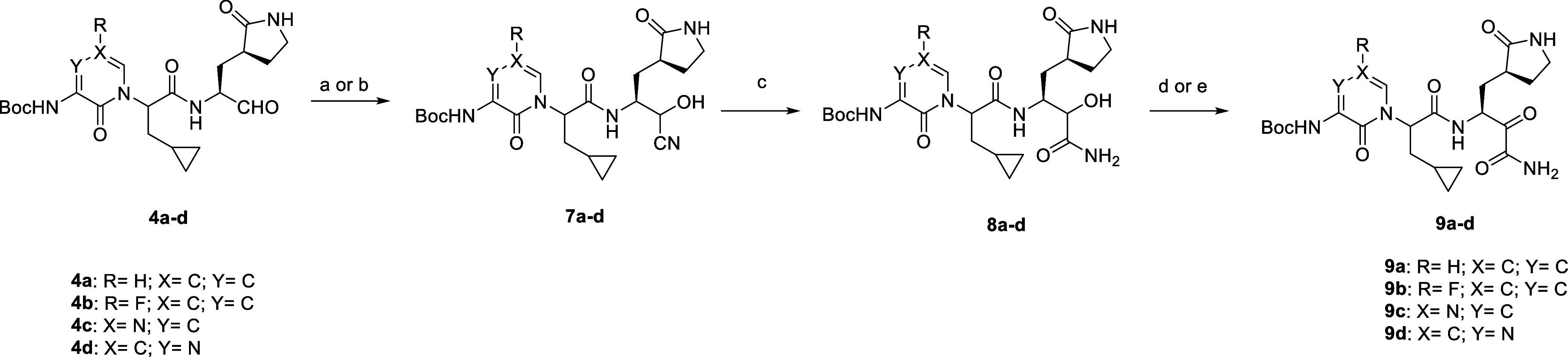
Synthesis of α-Ketoamides (**9a–d**) Reagents and conditions: (a) acetone cyanohydrin, TEA, DCM, 0 °C—rt, 3 h, 82%; (b) NaCN, sodium bisulfite, MeOH, H_2_O, 0 °C, 88–92%; (c) 30% H_2_O_2_ (aq), LiOH·H_2_O, MeOH, 0 °C—rt, 1 h, 33–68%; (d) for **9a**: DMP, DMF, rt, 3 h, 31%; (e) for **9b–d**: IBX, acetone, reflux, 5 h, 58–69%.

The primary α-ketoamides **9a-d** were prepared by a different synthetic strategy that involved a cyanohydration reaction (Scheme 3). For example, the treatment of **4a** with acetone cyanohydrin under basic conditions gave 82% of the desired product **7a** as a mixture of diastereomers.^25^ Cyanohydrin **7a** was subsequently hydrolyzed to the hydroxyamide **8a** using H_2_O_2_/LiOH in methanol as a solvent. Finally, **8a** was oxidized by DMP to the α-ketoamide **9a** under the optimized reaction conditions described above.

The analogues **9c** and **9d** featured electron-deficient pyrimidone and pyrazinone heterocycles, respectively, and **9b** was substituted with fluorine at the 5-position. The intermediate hydroxyamides **8b–d** were prepared as described for **8a** from **4b–d** (Supporting Information S3) and isolated in 33–48% yields. Their oxidation to the α-ketoamides was achieved with IBX in yields of 58–69%.

### Activities of Optimized α-Ketoamides against M^pro^ and Structural Basis of Inhibition

**Figure 2:**
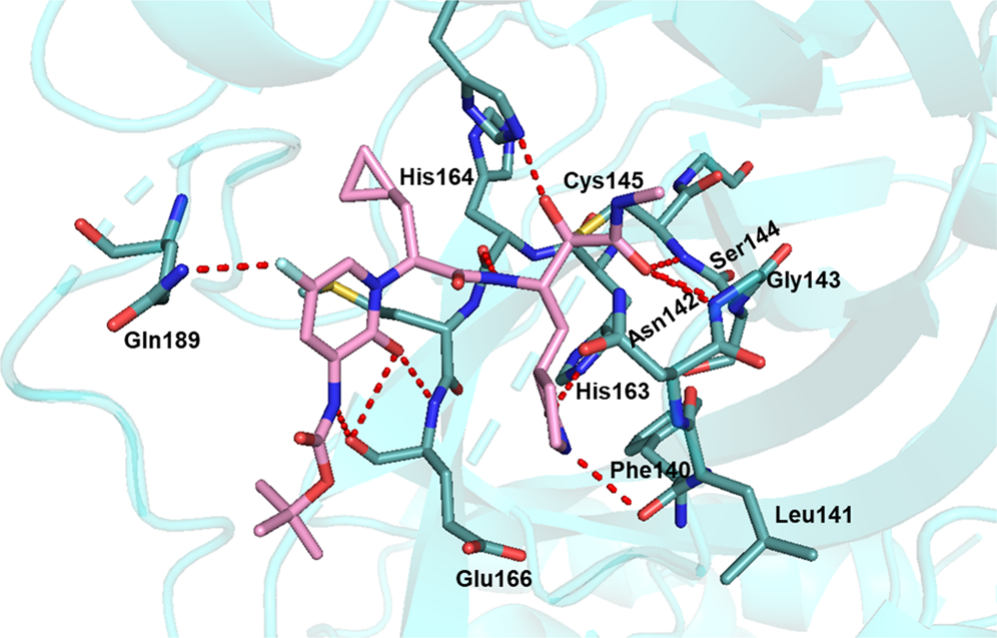
Overall cocrystal structure of the M^pro^ in complex with **6d** (PDB: 9F3A). Compound **6d** is colored in pink, and all H-bonds between the inhibitor and the corresponding M^pro^ residues are colored in red.

**Figure 3:**
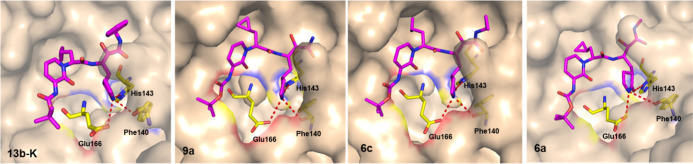
Overall crystal structure of M^pro^ in complex with **13b-K** (PDB: 6Y2E), **9a** (9GMQ), **6c** (8AIU), and **6a** (8AIV). The overall pattern of interactions between inhibitors and M^pro^ in the S1 pocket is conserved.

The inhibitory activity of the primary α-ketoamides **6a–c** and **9a**, which had smaller P1′ residues compared to **13b**, was probed with recombinant SARS-CoV-2 M^pro^ by measuring the compound-mediated reduction of the cleavage rate of a model substrate. The *N*-methyl-substituted **6a** inhibited M^pro^ activity with an IC_50_ of 400 nM, thereby reaching equipotency with **13b** (IC_50_ = 380 nM; Table 1). A further increase and branching of the alkyl chain at the P1′ position to an isopropyl group as in **6b** or a cyclopropyl group as in **6c** was detrimental for activity because the IC_50_s increased to 3450 nM and 500 nM, respectively. On the other hand, the unsubstituted primary α-ketoamide **9a** displayed the highest activity in the series with an IC_50_ of 110 nM. To gain a structural understanding of these findings, we elucidated the cocrystal structures of SARS-CoV-2 M^pro^ with **6a**, **6c**, and **9a**. The overall pattern of interactions between the inhibitor and M^pro^ was conserved and corresponded to what we previously described for compounds **13b** and **13b-K** (Figures 2, 3, and S2).^6,19^ Briefly, the amide oxygen of the warhead interacts with the main-chain amides of the amino acid residues forming the oxyanion hole, i.e., Gly143 and Cys145. The alpha-keto group, after its conversion to a thiohemiketal via reaction with the sulfhydryl of Cys145, accepts a hydrogen bond (2.5–2.7 Å) from His41 Nε2. The P1 lactam oxygen accepts a hydrogen bond (2.6–2.7 Å) from His163 Nε2, and the lactam nitrogen is involved in a bifurcated interaction with Glu166 Oε2 (2.9–3.2 Å) and Phe140 O (3.2–3.5 Å). The P1-main-chain amide donates a 3.0 Å hydrogen bond to His164 O. As in most peptidomimetic M^pro^ inhibitors of this type, the P2 carbonyl is devoid of direct interactions with the protein. The P2 nitrogen is engaged in the formation of the pyridone and therefore not available for interactions with the protein. The cyclopropyl moiety resulted from our earlier optimization of the size and shape for P2-substituents.^6^ The P2/P3 pyridone oxygen accepts a hydrogen bond (2.8–2.9 Å) from Glu166 N, and the P3 amide interacts (3.0 Å) with the carbonyl oxygen of the same amino-acid residue. Boc as the P4 capping group does not interact directly with the S4 site but is displaced toward Pro168.

**Table 1:**
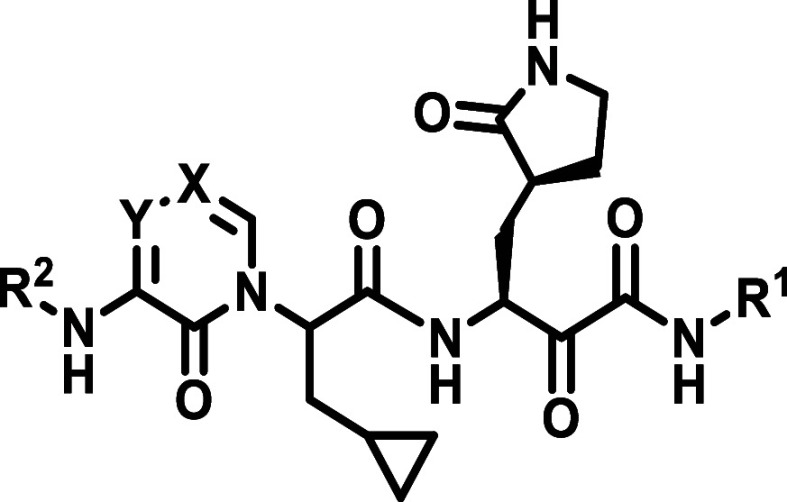

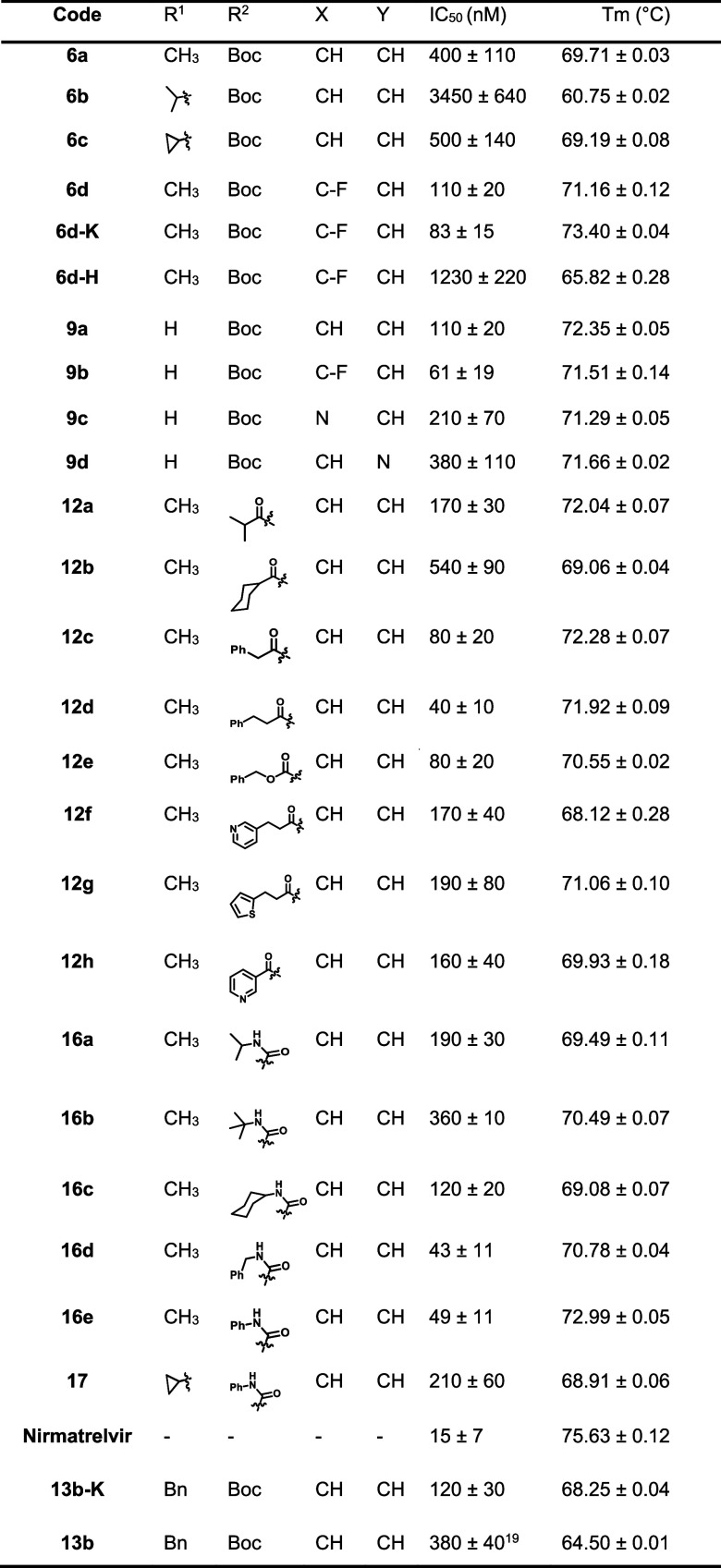
Inhibitory Activity and *T*_m_ Values of α-Ketoamides against Recombinant M^pro^

a ±Standard deviation (SD), *n* = 3.

**Figure 4:**
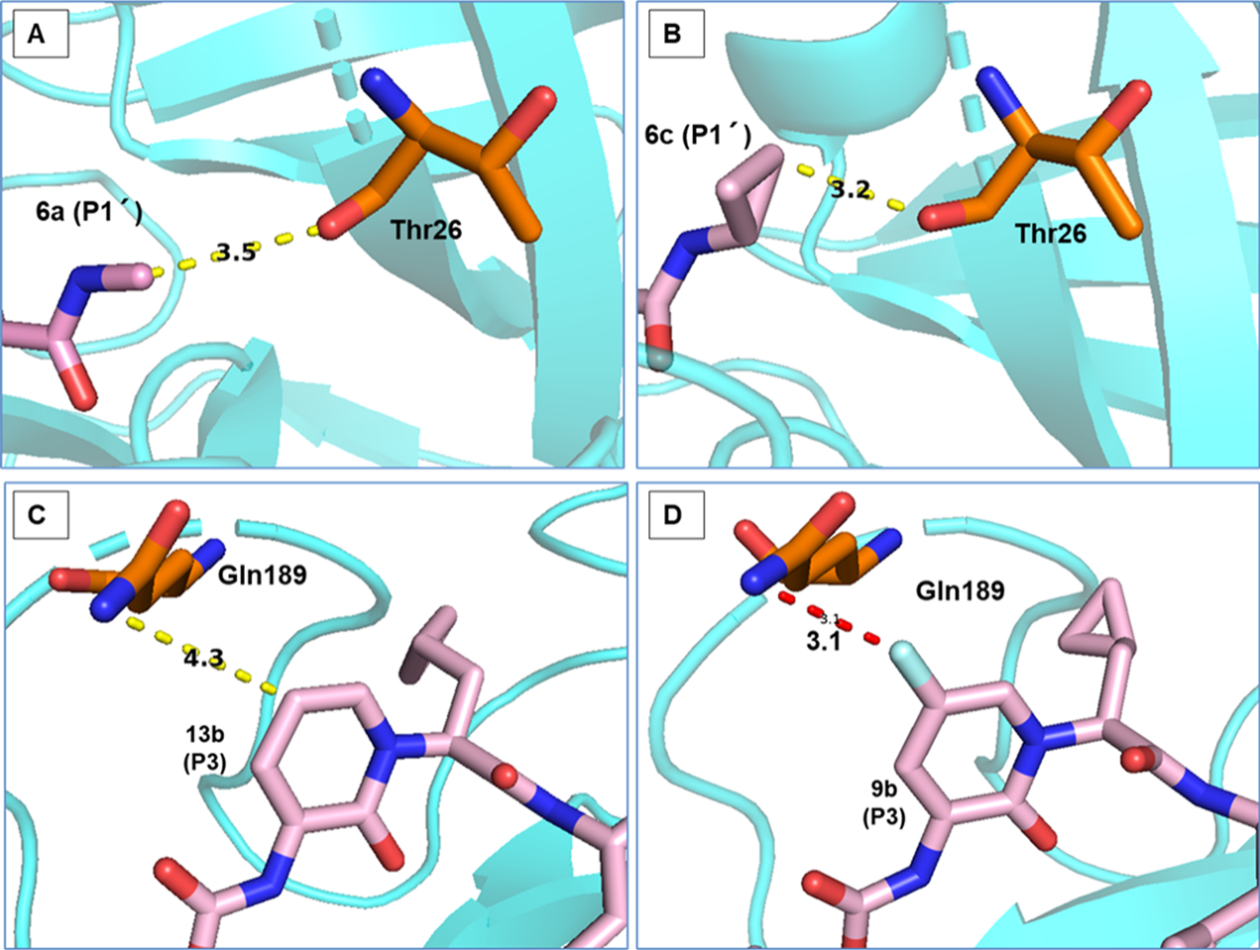
Crystal structures of M^pro^ in complex with **6a** (A, PDB code: 8AIV) and **6c** (B, PDB code: 8AIU). The P1′ cyclopropyl derivative **6c** shows close contact with Thr26. Crystal structure of M^pro^ in complex with **13b**-**K** (C, PDB code: 6Y2G) and **9b** (D, PDB code: 9F2V).

In the complex with **6a**, the closest distance between the P1′ *N*-methyl group of the inhibitor and the M^pro^ (Thr26 O) was 3.5 Å (Figures 3 and 4). The P1′ cyclopropyl derivative **6c** featured a close contact (3.2 Å) with Thr26 O, which is probably energetically unfavorable and is the likely cause for the inferior inhibitory activity. The cyclopropyl moiety marks the maximum ligand size that can be accommodated in the small S1′ pocket; the branched isopropyl moiety is too large to bind to this site. Nevertheless, many M^pro^ inhibitors feature larger P1′ moieties, such as benzyl as in compound **13b-K**. However, the X-ray structure of the M^pro^ in complex with **13b-K** (Figure 3) shows that the bulk of the inhibitor, i.e., the phenyl group, is not embedded within the S1′ pocket.^19^ Having an inhibitor with a size larger than the S1′ pocket is not necessarily a disadvantage; on the contrary, it introduces some rotational freedom and is therefore favorable for entropic reasons, compared to inhibitors that are fully accommodated in the binding pocket.

The pyrimidone **9c** and the pyrazinone **9d** inhibited M^pro^ with IC_50_ values of 210 nM and 380 nM, respectively, and thus were nearly equipotent to the pyridone **13b** that displayed an IC_50_ value of 380 nM. In contrast, the 5-fluoropyridone **9b** had a more than 5-fold increased potency, inhibiting the enzyme activity with an IC_50_ of 61 nM. A plausible reason for the increased affinity was provided by the crystal structure of **9b** in complex with M^pro^: The *meta*-fluoro substituent of the pyridone ring formed a hydrogen bond to Gln189 Nε2, as shown by a distance of 3.1 Å between the heteroatoms in chain A (Figure 4) (chain B could not be measured due to the low electron density of the Gln189 side chain). Thus, our initial design hypothesis that an additional interaction to the S3 pocket is gained by a 5-substitution of the pyridone could be confirmed experimentally.

### Synthesis of N-Terminally Modified α-Ketoamide Inhibitors

**Scheme 4:**
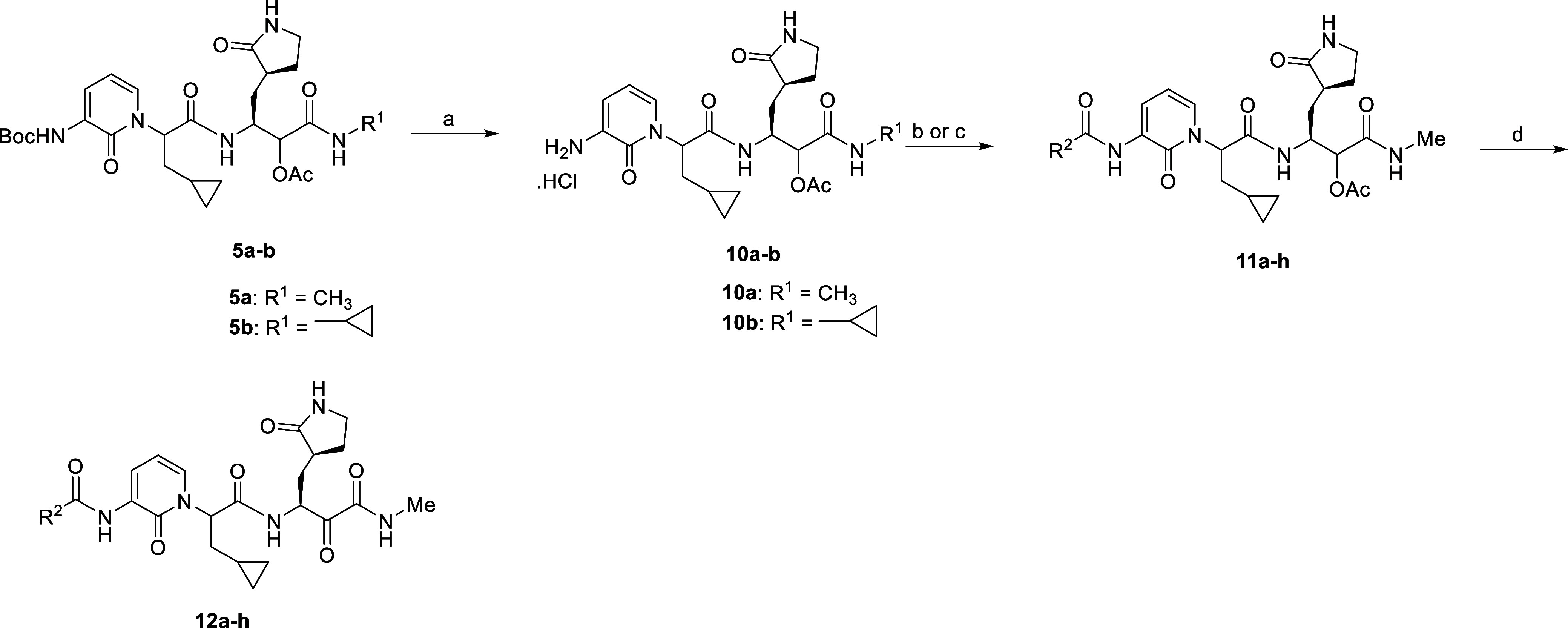
Synthesis of Acylated α-Ketoamides **12a–h** Reagents and conditions: (a) 4 M HCl, CH_2_Cl_2_, 0 °C—rt, 6 h, 84–98%; (b) R_2_-COOH, HATU, TEA, DMF, 0 °C, 10 h, 65–95%; (c) for **11e**: PhCH_2_OCOCl, NaHCO_3_, aq. THF, 0 °C—rt, 16 h, 83%; (d) (i) LiOH·H_2_O, MeOH, H_2_O, 0 °C—rt, 1.5 h; (ii) DMP, CH_2_Cl_2_, rt, 2 h, 47–68%.

A third series of compounds aimed at modifying the *N*-terminal capping unit of **13b**. Because strong binding interactions with residues in the S4 pocket were not observed, this part of the molecule was expected to allow fine-tuning of ADME or pharmacokinetic properties. In two subseries, the carbamate moiety of the *N*-methyl α-ketoamide **6a** was replaced by acyl residues and by urea moieties. All compounds were prepared starting from the late-stage intermediate **10a**, except **17**, which carried a cyclopropyl group as S1′ residue and was obtained from the corresponding intermediate **10b** (Scheme 4). Compound **10a**, prepared by Boc deprotection of the α-acyloxy amide **5a**, was acylated using HATU and trimethylamine to give **11a–h** in yields of 65–95%. These were further converted to nine acyl-capped α-ketoamides **12a–h** in two steps (yields: 47–68%), i.e., LiOH hydrolysis in aqueous MeOH followed by DMP oxidation at room temperature (Scheme 4 and Table 1).

**Scheme 5:**
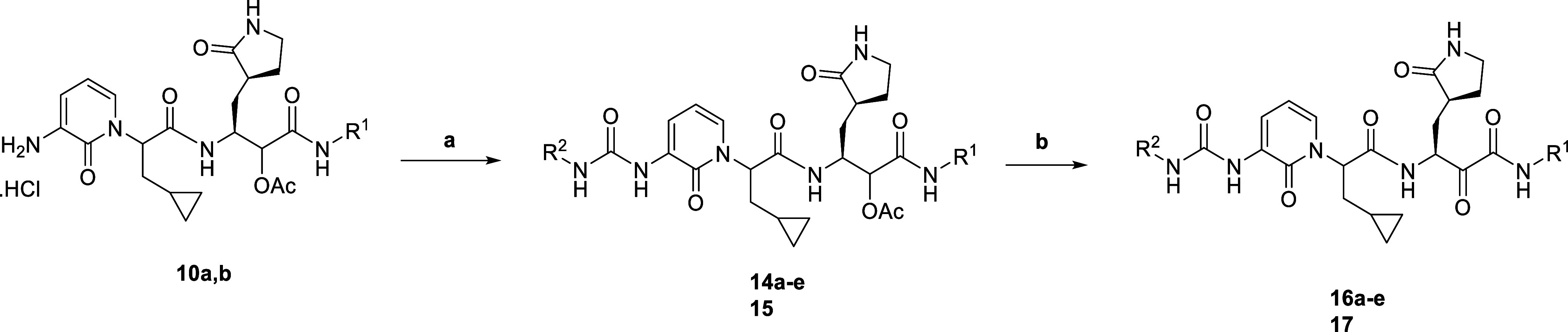
Synthesis of Urea α-Ketoamides **16a–e** and **17** Reagents and Conditions: (a) alkyl/aryl isocyanates, CH_2_Cl_2_, DMF, 0 °C—rt, 24 h, 38–68%; (b) (i) LiOH·H_2_O, MeOH, H_2_O, 0 °C—rt, 1.5 h; (ii) DMP, CH_2_Cl_2_, rt, 2 h, 36–68% over two steps.

Because the urea group is a bioisostere for a carbamate with enhanced stability, we prepared a series of ureas comprising **16a–e** and **17**. For this purpose, the intermediates **10a** and **10b** were reacted with different isocyanates using reported methods^26^ to give **14a–e** and **15** (Scheme 5). The α-acetoxy amides **14a–e** and **15** were further hydrolyzed and oxidized to the corresponding final α-ketoamides **16a–e** and **17** as reported for the previous subseries.

### Inhibitory Activities of N-Terminally Modified α-Ketoamides against M^pro^

For both subseries with N-terminal capping groups, the inhibitory potency toward recombinant M^pro^ was tested. Substitutions of the Boc group by acyl moieties were generally allowed and favorable because 7 out of 8 analogues were more potent than the reference **6a** with the corresponding *C*-terminal *N*-Me-α-ketoamide (IC_50_ = 400 nM). While an isopropyl group attached to the carbonyl group was slightly more potent than **6a**, the conformationally fixed cyclohexyl group led in **12b** to a loss of activity (IC_50_ = 540 nM). A benzyl residue as in **12c** led to an increase in potency (IC_50_ = 80 nM) that could be further enhanced by a two-carbon spacer as in **12d** (IC_50_ = 40 nM). The beneficial role of the phenyl ring was further illustrated by carbamate **12e** that was 5 times more potent than carbamate **6a**. On the other hand, the replacement of the phenyl ring of **12d** by thiophen as in **12g** or pyridine as in **12f** decreased activity. Analogues with substituted urea capping groups showed similar trends: The introduction of phenyl or benzyl groups attached to the urea as in **16e** and **16d**, respectively, led to an increase of potency with IC_50_s of 43 and 49 nM. This was rather due to the phenyl group and not due to the O → NH replacement because the latter alone had little impact on activity, as shown by the comparison of **6a** and **16b**. As demonstrated by **17** (compared to **6c**), the beneficial effect of an *N*-terminal phenyl residue was retained upon exchanging the *C*-terminal rest for cyclopropyl, implying additive SAR in vitro. In summary, the modifications of residues addressing the P1′ and P4 pockets led to up to 9.5-fold increase of potency compared to the starting point **13b**, as demonstrated by an IC_50_ of 40 nM for the α-ketoamide **12d** with a small *N*-Me group for P1′ and a 3-phenylpropanoyl unit for P4.

Most newly prepared compounds contain 50% (*R*,*S*,*S*)-configured diastereomer, that is, predicted to be far less active than the (*S*,*S*,*S*)-configured diastereomer based on the findings for **13b**.^19^ To verify this, we selected **6d**, which had an overall favorable profile of properties (see below), and prepared its corresponding (*S*,*S*,*S*)-diastereomer **6d-K** and the (*R*,*S*,*S*) diastereomer **6d-H**. This was achieved by a HPLC separation of hydroxyamide obtained after deacetylation of **5d**, followed by their oxidation to **6d-K** and **6d-H** (Supporting Information S13). As expected, **6d-K** showed an improved enzymatic activity of 83 ± 15 nM, compared to 110 ± 20 nM for the diastereomeric mixture **6d**. Correspondingly, the activity of **6d-H** was worse (IC_50_ = 1230 ± 220 nM).

### Melting Temperatures (*T*_m_’s) of M^pro^-Inhibitor Complexes

Melting temperature (*T*_m_) assays were performed in order to examine the stability of M^pro^ complexes, providing information that is complementary to the functional inhibition data. For all α-ketoamides, the melting temperatures of the complex with M^pro^ were determined by measuring the fluorescence signal at 350 nm. As can be seen from the melting curves of **13b** and **13b-K** in complex with M^pro^, the *T*_m_ value for the mixture of diastereomers **13b** was significantly lower than for the pure diastereomer **13b-K** (Figure S3). For the former, the *T*_m_ peak appears broader than for the latter, which might be indicative of **13b** containing two diastereomers. Compounds with a free P1′ amino group and/or modified P2/P3 substituents such as **6d** or **9a–d** tended to show higher melting temperatures over 71 °C (Table 1). With 73.0 and 73.4 °C, **16e** and **6d-K** showed the highest *T*_m_ values across the series, at IC_50_s of 49 and 83 nM, respectively. Only nirmatrelvir showed a higher melting temperature of 76 °C, at an IC_50_ of 15 nM. Overall, an inverse correlation between *T*_m_ and IC_50_ values was observed (*R*^2^ = 0.58).

### Inhibition of Cathepsin L

Cathepsin L is a cysteine protease of the host cell that is involved in intracellular protein catabolism; it is also implicated in SARS-CoV-2 entry.^27^ To assess the extent of inhibition of human cathepsin L by the α-ketoamides, we used a fluorogenic assay with the substrate Z-Phe-Arg-MCA and an inhibitor concentration of 10 μM. In this assay, the activity of the enzyme was inhibited by compound **13b** to the extent of 87.6% (Table 2). The only α-ketoamide displaying a similar inhibiting activity (82.3%) was compound **9b**, which carries an additional fluorine atom in the 5-position of the P2/P3-pyridone. A relatively high cathepsin L inhibition activity of 59.3% was also observed for **9a**, which features an unsubstituted P1’ amino group, similar to **9b**. All other α-ketoamides in this series displayed cathepsin L inhibition rates of lower than 43.0%, with a median value of 33.9%. For comparison, the approved HCV NS3-4A inhibitor boceprevir showed an inhibition rate of 19.9% in our assay. The lowest cathepsin L inhibition activity of 23.2% was displayed by compound **6a**, the P1′ *N*-methyl derivative of **13b**.

**Table 2:** Cathepsin L Inhibition by α-Ketoamides^a^

code	CTSL inhibition [%]
**6a**	23.2 ± 1.2
**6c**	34.3 ± 5.8
**6d**	39.4 ± 0.6
**6d-K**	35.8 ± 1.9
**6d-H**	0
**9a**	59.3 ± 1.8
**9b**	82.3 ± 1.8
**12a**	43.0 ± 2.5
**12c**	33.7 ± 7.4
**12d**	26.7 ± 5.3
**16e**	27.5 ± 5.6
**13b**	87.6 ± 0.8
**13b-K**	81.9 ± 1.7
**Nirmatrelvir**	0
**Boceprevir**	19.9 ± 4.2
**DMSO**	0

a ±Standard deviation (SD), *n* = 3.

### Structural Basis of Cathepsin Inhibition

In order to rationalize these observations in structural terms, we made use of the crystal structure of inhibitor **13b** in complex with cathepsin L.^21^ The interaction between **13b** and cathepsin L is governed by a total of six hydrogen bonds. The P1′ carbonyl oxygen interacts with the amides of the oxyanion hole of cathepsin L, the side-chain Nε2 of Gln19 (2.7 Å) and the main-chain amide of Cys25 (2.8 Å). Residue Cys25 is the active nucleophile that forms a covalent bond (1.77 Å) with the α-keto group of **13b**. The α-keto group is converted into a thiohemiketal in this reaction. The resulting hydroxyl group donates a 3.0 Å hydrogen bond to the main-chain oxygen of Asp162 and accepts a 2.5 Å hydrogen bond from the Nδ1 atom of His163. The P1 amide of **13b** donates a 2.9 Å hydrogen bond to Asp162, and the carbonyl oxygen of the P1 moiety accepts a 3.3 Å hydrogen bond from Gly68. Of note, the P2/P3 residue of inhibitor **13b** makes no strong interactions with cathepsin L. In addition to the H-bonds mentioned above, the P1′ benzyl group interacts weakly with the side chain of Gln21 and with a DMSO solvent molecule (which in turn also accepts a 2.8 Å hydrogen bond from the P1′ nitrogen of **13b**).

On the basis of the crystal structure of the cathepsin L—**13b** complex,^21^ we constructed models for complexes with the **13b** derivatives described here. All these compounds fit into the ligand binding site of cathepsin L without steric problems. The hydrogen-bonding interactions found in the crystal structure^21^ are all very likely conserved in the complexes with **13b** derivatives. However, since the P1′ *N*-benzyl group of **13b** is missing in the derivatives, the weak interaction with Gln21 of cathepsin L is absent. A more important reason for the reduced cathepsin L inhibition by **13b** derivatives lacking a bulky P1′ group may be the loss of entropy because of the missing rotatable P1′ group.

**Figure 5:**
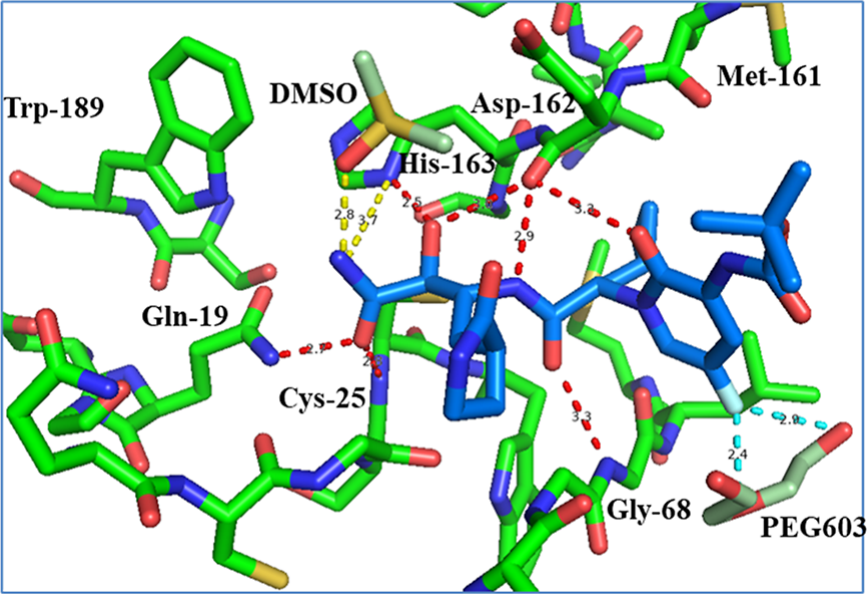
Structural model of cathepsin L in complex with **9b**. Model constructed using electron density map of cathepsin L in complex with **13b** (PDB code: 8PRX): Strong H-bonds are formed between Gln-19 Nε2 and P1́O (2.7 Å) and between Cys25 N and P1́O (2.8 Å); H-bonds are colored in red.

In our model, the extra fluoro group present in the 5-position of the P2/P3 pyridone appears to interact with a polyethylene glycol molecule detected in the electron density map of the **13b** crystal structure.^21^ However, this is of little relevance to our model as polyethylene glycol is an artifact of crystallization. Interestingly, the other 5-fluoro pyridone compound in this series, **6d**, exhibits average cathepsin L inhibition (38.5%). We speculate that this may be due to the P1′ *N*-methyl group as α-ketoamides carrying this moiety display lower cathepsin L inhibition activity. Taking the cathepsin L inhibition results for **6d**, **9a**, and **9b** together, the elevated cathepsin L inhibition potency of compounds **9a** and **9b** (Figure 5) appears to arise from the presence of an unsubstituted amino group in the P1′ position rather than from the fluorine substituent in the 5-position of the pyridone.

### Antiviral Testing

The antiviral activities of selected α-ketoamides were determined against a SARS-CoV-2 wildtype strain in Vero E6 cells as well as A549 cells transfected with ACE2+TMPRSS2,^28^ referred to as A549-AT cells. For A549-AT cells, the ATP content was quantified by a CellTiterGlo kit to detect virus-mediated cytopathic effects and their prevention by the α-ketoamides. The Vero E6 cells constitutively expressed GFP that allowed us to quantify the cytopathogenic effect of the virus as well as its compound-mediated inhibition. Many α-ketoamide analogues exerted an antiviral potency in the Vero E6 cell line that was comparable to the reference compound **13b** with an EC_50_ of 4.3 μM (Table 3). For example, analogue **9b**, carrying optimized residues in the P1′ and P2/P3 positions, had a lower EC_50_ of 2.6 μM, which however did not fully reflect the 6.2-fold gain of in vitro potency with respect to the IC_50_. Surprisingly, the EC_50_ of **9b** was slightly higher in A549-AT cells compared to **13b**, suggesting differences related to the expression of ACE2+TMPRSS2. The same was found for **9a** carrying optimized residues in the P1′ position, which was found to be slightly more active than **13b** in Vero E6 cells but slightly less active in A549-AT cells. Compounds **6c** and **6d**, carrying optimized residues in the P1′ and P2/P3 positions, exhibited slightly improved EC_50_ values over **13b** in both cell lines. Also, the best M^pro^ inhibitor **12d** was merely equipotent to **13b** with an EC_50_ of 4.1 μM in Vero E6 cells. The finding that a good M^pro^ inhibitory activity on the enzyme level was not sufficient to confer antiviral activity was further underlined by **9c**, carrying an electron-deficient pyridone, which was poorly active in the cellular assays with EC_50_’s of 30.3 and >50 μM in Vero E6 and in A549-AT cells, respectively. Data obtained with A549-AT cells were overall consistent with those obtained for Vero E6. However, there was a trend toward slightly higher EC_50_ values in A549-AT cells compared to those in Vero E6 cells. In summary, the optimized pyridone α-ketoamides exerted good antiviral activity, which was however uncorrelated to their in vitro potency. Potential reasons are discussed below.

**Table 3:** Anti-SARS-CoV-2 Activities of α-Ketoamides

code	EC_50_^a^ (A549-AT) [μM]	EC_50_^b^ (Vero E6) [μM]	CC_50_ [μM]^c^
**6a**	2.9 ± 0.5	3.97 ± 1.3	>100
**6c**	2.8 ± 0.3	3.5 ± 0.03	>100
**6d**	1.6 ± 0.1	3.5 ± 2.0	>50
**9a**	5.7 ± 0.7	3.4 ± 0.1	>10
**9b**	4.2 ± 1.1	2.6 ± 1.3	>10
**9c**	>50	30.3 ± 0.1	>100
**12a**	9.5 ± 1.2	5.8 ± 0.2	>50
**12c**	6.3 ± 1.8	5.6 ± 0.5	>100
**12d**	4.7 ± 0.1	4.1 ± 0.1	>10
**12g**	30.6 ± 6.1	18.0 ± 0.2	>50
**12h**	>50	>50	>50
**16e**	6.3 ± 0.03	7.0 ± 2.6	>10
**17**	>50	44.5 ± 5.7	>50
**Nirmatrelvir**	0.07 ± 0.02	0.08	>10
**13b-k**	1.0 ± 0.05	1.0	>10
**13b**	2.5 ± 0.9	4.3 ± 0.4	>100

a Tested on SARS2-ZG/A549^ACE2+TMPRSS2^.

b Tested on SARS2-GHB/VeroE6-GFP with 0.5 μM CP-100356.

c Tested on Vero E6 cells by a tetrazolium salt reduction assay with MTS n.d.: not determined, ± standard deviation (SD).

### In Vitro ADME Studies

The asynchronous enzymatic and cellular activities implied that other factors that impacted inhibition, such as stability or permeability, contributed to the antiviral effects. We therefore determined selected in vitro ADME properties, i.e., plasma protein binding, metabolic stability, and plasma stability, to investigate their variability across α-ketoamide analogues. Moreover, we determined permeability using a commercial parallel artificial membrane permeation assay (PAMPA) kit. As reported before for the reference **13b**, the new analogues did not display any metabolic liabilities in mouse or human microsomes (Table 4). Similarly, most of the compounds were stable in mouse and human plasma. With half-lives around 2 h, **6c**, **12d**, and **12g** exhibited reduced stability in mouse plasma; however, liabilities were not observed in human plasma. With respect to plasma protein binding, the acyl-modified analogues **12b–d** and **12g** had the lowest values in the range of 50–70%. The Boc-capped analogues (**6a**, **6c**, **6d**) and selected ureas (**16c**, **16e**) had a binding between 70 and 90%, similar to **13b** which had a plasma protein binding of ca. 90%.^6^ Except for **6c**, which had a high permeability and was the most lipophilic compound within the series, all tested α-ketoamides exhibited a moderate permeability across membranes using a PAMPA assay. That was also true for **13b-K** (3.6 × 10^–6^ cm/s) Overall, the in vitro ADME properties were favorable and relatively constant across the investigated α-ketoamides.

**Table 4:** In Vitro ADME Properties of Selected Pyridone α-Ketoamides^a^

code	metabolic stability [min] (Clearance [μL/min/mg protein])		plasma stability: *t*_1/2_ in [min]		plasma protein binding [%]		permeability [cm/s]
	mouse	human	mouse	human	mouse	human	
**6a**	>60 (<23)	>60 (<23)	>240	>240	79.7 ± 3.2	81.1 ± 2.6	1.7 × 10^–6^
**6c**	>60 (<23)	>60 (<23)	143.2	>240	72.7 ± 9.0	84.5 ± 2.2	10.0 × 10^–6^
**6d**	>60 (<23)	>60 (<23)	231.0	>240	89.9 ± 3.5	83.9 ± 0.5	n.d.
**9a**	>60 (<23)	n.d.	>240	>240	73.4 ± 5.1	59.6 ± 2.0	1.8 × 10^–6^
**9b**	>60 (<23)	>60 (<23)	231.0	>240	80.2 ± 4.4	84.8 ± 1.4	n.d.
**12a**	>60 (<23)	n.d.	>240	>240	49.5 ± 4.1	29.1 ± 6.1	n.d.
**12b**	>60 (<23)	>60 (<23)	>240	>240	74.0 ± 5.1	65.5 ± 14.3	n.d.
**12c**	>60 (<23)	>60 (<23)	>240	>240	54.4 ± 15.2	34.2 ± 21.6	1.5 × 10^–6^
**12d**	>60 (<23)	>60 (<23)	138.6	>240	72.3	67.0 ± 11.1	3.8 × 10^–6^
**12g**	>60 (<23)	>60 (<23)	138.6	231.0	51.7 ± 6.2	71.5 ± 9.6	3.9 × 10^–6^
**16c**	>60 (<23)	>60 (<23)	>240	>240	83.5 ± 2.1	72.7 ± 6.5	n.d.
**16d**	>60 (<23)	>60 (<23)	>240	>240	69.0 ± 9.1	67.5 ± 9.0	n.d.
**16e**	>60 (<23)	>60 (<23)	231.0	>240	90.2 ± 2.7	95.1 ± 0.3	n.d.

a n.d.: not determined, ± standard deviation (SD).

### Pharmacokinetic Studies

With encouraging in vitro ADME properties, we selected ten compounds for cassette pharmacokinetic (PK) studies at 1 mg/kg IV, namely, **6a**, **6c**, **6d**, **9a**, **9b**, **12b–d**, **16c**, and **16e**. The selection was based on activity as well as on the ADME properties. Moreover, we aimed to see whether compounds with a higher unbound fraction had a higher renal clearance, to get information for future structure–PK relationships. Among the tested compounds, only the ureas **16c** and **16e** had half-lives > 0.5 h, whereas all other compounds, irrespective of high or medium plasma-protein binding, had half-lives < 0.4 h (Table 5; Figure 7). Besides **16e**, several compounds from the 9 and 12 series (**9a**, **9b**, **12b**, and **12c**) had a high initial concentration C0, but overall, a low exposure, expressed as AUC, was observed. Plasma clearance was high, except for **16e**, for which a moderate plasma clearance was observed. Compounds **6c**, **12b**, **12d**, **16c**, and **16e** exhibited a higher volume of distribution, suggesting good tissue distribution, which would be in line with the observed gut permeability results.

**Table 5:** Pharmacokinetic Parameters^a^

code	*t*_1/2_ [h]	*C*_0_ [ng/mL]	AUC_0–*t*_ [ng/mL·h]	MRT [h]	Vz_obs [L/kg]	Cl_obs [mL/min/kg]
**6a**	0.14 ± 0.0	355.23 ± 135.6	113.0 ± 14.4	0.23 ± 0.1	1.76 ± 0.6	147.4 ± 17.6
**6c**	0.35 ± 0.1	257.02 ± 134.6	87.01 ± 11.6	0.43 ± 0.2	5.14 ± 1.6	166.91 ± 6.7
**6d**	n.d.	238.75 ± 115.8	75.05 ± 17.7	n.d.	n.d.	n.d.
**9a**	0.15	417.24 ± 108.9	118.68 ± 3.6	0.25	1.78	135.9
**9b**	0.31 ± 0.0	360.23 ± 120.8	139.12 ± 16.2	0.41 ± 0.1	2.92 ± 0.5	108.11 ± 9.3
**12b**	0.18 ± 0.1	358.84 ± 321.3	88.01 ± 34.6	0.24 ± 0.2	3.35 ± 2.8	196.90 ± 69.2
**12c**	0.24 ± 0.1	445.74 ± 341.7	132.93 ± 32.1	0.31 ± 0.2	2.61 ± 1.2	121.10 ± 22.9
**12d**	0.31 ± 0.0	166.02 ± 89.3	62.33 ± 1.9	0.39 ± 0.1	6.48 ± 0.0	241.61 ± 13.7
**16c**	0.77 ± 0.1	201.50 ± 141.0	72.27 ± 6.0	0.70 ± 0.1	14.39 ± 0.4	217.61 ± 18.6
**16e**	0.51 ± 0.0	437.02 ± 81.3	241.75 ± 50.7	0.51 ± 0.1	3.07 ± 0.8	69.31 ± 14.4

a *t*_1/2_: half-life, *C*_max_: maximal concentration, AUC_0–*t*_: area under the curve from time point 0 until *t*, MRT: mean residence time, *V*_z_: volume of distribution, Cl: clearance, ± standard deviation (SD).

**Figure 6:**
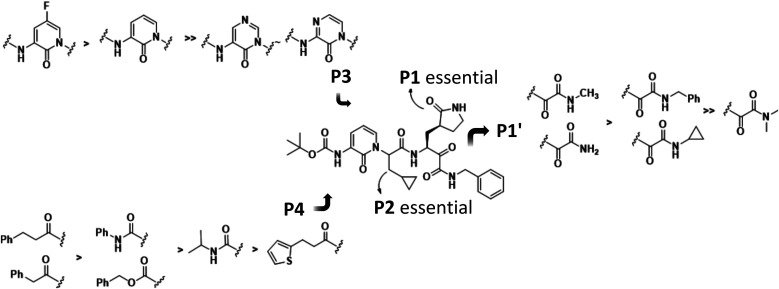
Summary of the structure–activity relationship of pyridone α-ketoamides derived in this study.

**Figure 7:**
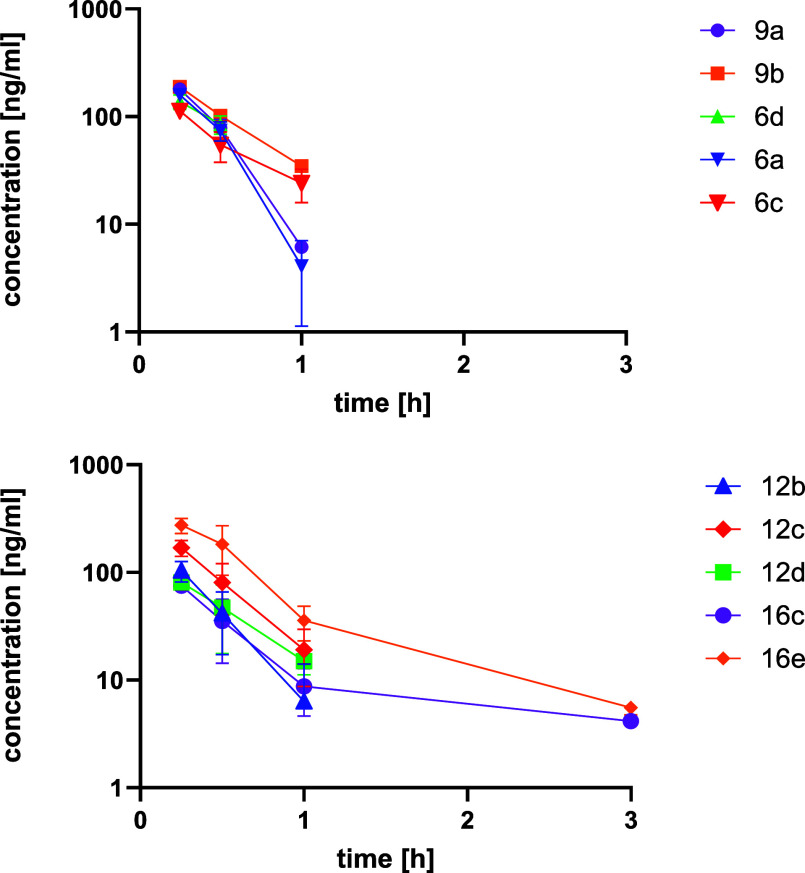
Pharmacokinetic studies of pyridone α-ketoamides in mice. Plasma concentrations are displayed after cassette dosing of 1 mg/kg IV for **6a**, **6c**, **6d**, **9a**, and **9b** (upper panel) and for **12b**, **12c**, **12d**, **16c**, and **16e** (lower panel). *n* = 2 animals were used per study.

## Discussion

Therapies targeting various stages of the viral life cycle have demonstrated significant efficacy in combating COVID-19.^5^ The combination of nirmatrelvir and molnupiravir, for example, has shown substantial antiviral efficacy in mice compared to monotherapy with either compound.^29^ Preclinical studies of host protease TMPRSS2 inhibitors such as nafamostat and camostat in combination with approved SARS-CoV-2 drugs have also demonstrated effectiveness in combating the disease.^30^ Dual inhibition strategies with two drugs may offer advantages, but they also present challenges related to pharmacokinetics, drug–drug interaction potential, pharmacodynamics, toxicology, and safety in clinical applications. Notably, SARS-CoV-2 Omicron variants such as the BA.5 strain utilize the cathepsin L entry pathway instead of the TMPRSS2 pathway observed in earlier variants.^27^ This finding triggered the optimization of single compounds with dual activities against the coronavirus protease and the host protease cathepsin L, most of them featuring an aldehyde warhead. The α-ketoamide warhead also has a high nucleophilicity, and indeed, compound **13b**(21) and its derivatives exhibit activity against cathepsin L. As shown above, the α-ketoamides prepared here cover a broad range of cathepsin L inhibition activities from high (87.6% for **13b**, 82.3% for the 5-fluoro pyridone **9b**) over intermediate (59.3% for **9a**) to low (mean of 32.4% for all other α-ketoamides discussed here), all measured at concentrations of 10 μM. Thus, the α-ketoamides allow us to either dial in or avoid the inhibition of cathepsin L. Due to its importance for host cell processes, cathepsin L has long been regarded as an undesired off-target. However, this view has changed recently, because of the attractive perspective to inhibit different steps of the SARS-CoV-2 life cycle with a single drug.^21^ Moreover, the approved HCV NS3/4A drug boceprevir, inhibiting cathepsin L by approx. 20% in our assay, demonstrates that the inhibition of this target in the course of viral hepatitis therapy is obviously acceptable.

The primary molecular target in this study was the SARS-CoV-2 main protease. The structure of M^pro^ with the first generation inhibitor **13b**, reported by us in early 2020,^6^ allowed a rational design of inhibitors. The impact of structural knowledge on the optimization program is best illustrated by three findings as depicted in Figure 1: We observed that the distance of the pyridone residue in **13b** was too large for a hydrogen bond to Gln189 of the S3 site. However, the insertion of a hydrogen bond acceptor such as fluorine at the 5-position of the pyridone was predicted to shorten the proximity to Gln189 and to enable hydrogen bonding. Compounds **6d** and **9b** were thus synthesized and indeed found to form the aforementioned hydrogen bond. Moreover, this translated into improved IC_50_ values of 110 and 61 nM in the M^pro^ inhibition assay, respectively, thereby validating the structure-based design hypothesis. The second observation from the **13b** cocrystal structure concerned the *N*-benzyl amide group of the compound, which was too large to fill the S1′ site. We therefore prepared the unsubstituted α-ketoamide **9a** and then grew the P1′ substituents to methyl, cyclopropyl, or isopropyl. As predicted, these analogues reached into the S1′ site, but due to unfavorable contacts with Thr26 O, cyclopropyl was the largest residue that could be accommodated. In line with this, the simple primary α-ketoamide **9a** exhibited a superior IC_50_ value (110 nM) compared to **13b** (380 nM), while a further increase of the substituent as in **6a–c** led to a decrease in activity (Figure 6). Third, the cocrystal structures demonstrated few interactions of the S4 site with the Boc-group that capped the amino group of the pyridone. We therefore expected that this part of the molecule was amenable to larger structural variations and prepared the two subseries **12a–12h** and **16a–16e**. This yielded the most potent M^pro^ inhibitors **12d**, **16d**, or **16e** in the **13b** series of α-ketoamides that all exhibited IC_50_ values < 50 nM. Moreover, the capping group allowed a fine-tuning of physicochemical and pharmacokinetic properties. For example, compound **16e** was stable in in vitro assays and exhibited a clearance in vivo that was lower than that of **13b**(6) or of other analogues synthesized here. Compound **16e** also has a superior *T*_m_ value (73 °C) among all the analogs.

Structure–activity relationships were not only established with the SARS-CoV-2 M^pro^ enzyme assay but also with cellular antiviral assays. We found that enzymatic and cellular data were often not congruent: As mentioned above, the enzymatic inhibition of N-substituted analogues **6a–c** decreased with the increasing size of the substituent. However, in cell-based antiviral assays, compounds **6a** and **6c** had slightly better EC_50_ values compared to **9a** in A549-AT cells. Overall, the antiviral potency in the human cell line decreased in the order **6c** (2.8 μM) ≈ **6a** (2.9 μM) > **9a** (5.7 μM), which is the opposite of the order observed in the M^pro^ enzyme inhibition assay. The cyclopropyl analogue **6c** is the most lipophilic of the series, and we hypothesize that it is best suited for crossing the cell wall as we observed high permeability in the PAMPA.

Antiviral activity requires a high inhibitory activity to the target combined with high intracellular concentrations; the latter is determined by permeability, metabolism, and competing cellular interactions. This study illustrates that optimization of permeability and activity may even go in the opposite direction, as discussed for the P1′ residues. While the larger P4 residues as in **12d** enhance inhibitory activity, they are not beneficial for intracellular exposure. In fact, discrepancies between in vitro activity and cellular activity caused by small structural changes have often been observed for SARS-CoV-2 antivirals. For example, the boceprevir analogues MG-78, reported by us^25^ and other groups,^15^ and Pfizer’s nirmatrelvir^9^ are structurally highly similar, and both had superb inhibitory activities against M^pro^ of 15 nM and 13 nM, respectively. However, the small structural changes, in particular the use of the trifluoroacetyl group on the P4 position, led to a >100fold improvement in antiviral activity for nirmatrelvir when compared to MG-78.^15^ These discrepancies call for a more systematic inclusion of intracellular exposure data by either experimental methods and/or improved in silico prediction tools.

A correlation between antiviral activity and cathepsin L inhibition could not be seen. Potentially, a contribution of cathepsin L inhibition to an antiviral effect might have been more pronounced in Omicron strains which were not tested in this study. However, the data from the enzymatic assays show that cathepsin L was inhibited only to a minor degree at concentrations inducing an antiviral effect with the wildtype SARS-CoV-2 strain. Thus, a much higher cathepsin L inhibitory activity in the nM range would be required for a dual mechanism of action.

Although the in vitro ADME properties of the α-ketoamides were promising, our in vivo PK data revealed a high clearance, which was only improved for the urea derivative **16e**. The urea derivative **16e** also exhibited the highest plasma protein binding, and it is conceivable that this property is important for protecting against clearance. According to the extended clearance concept proposed by El-Kattan^31^ and Varma,^32^ hepatic uptake or renal clearance might influence clearance. The in vivo clearance mechanisms and the role of protein binding need to be investigated further to guide medicinal chemistry efforts.

## Conclusions

This study reports a structure-guided optimization of the early SARS-CoV-2 M^pro^ inhibitor **13b** on three key positions. This led to compounds that address cathepsin L as a secondary target and that possess up to 9.5-fold higher potency against M^pro^. Because the gain in antiviral activity was less pronounced compared to **13b**, further optimization of these α-ketoamides will focus on enhancing their cellular uptake and on understanding parameters that determine in vivo exposure. The improved synthetic access to the intermediate **4a** facilitates such efforts. We also described the synthetic method for a potent diastereomer of **6d** with it is improved enzymatic potency. Overall, the study shows the power of inhibitor design based on high-resolution cocrystal structures and contributes to the arsenal of coronavirus inhibitors that is much needed to prepare for future pandemics.

## Experimental Section

### General Experimental Information

Commercial reagents were used as received, and all other reagents were prepared using known literature procedures. All solvents used for reactions, workups, and purifications had the HPLC purity grade. Dried solvents were purchased in water-free form (99.5%, extra dry, absolute, AcroSeal, ACROS Organics) and used as received. Reactions were either monitored by liquid chromatography-coupled mass spectrometry (LCMS) analysis or thin-layer chromatography (TLC) on “TLC Silica gel 60 F254” plates (Merck) and visualized by staining with aqueous basic KMnO_4_. LCMS was conducted with an Agilent 1260 HPLC System with a DAD detector and an Agilent 6130 quadrupole mass detector with electrospray ionization (ESI) (MeCN/H_2_O + 0.1% HCOOH). Flash chromatography was performed on silica gel 60 (technical grade, pore size 60 Å, 40–63 μm, 230–400 mesh, Supelco). Reverse-phase high-pressure liquid chromatography was performed with a Phenomenex Luna C18 RP-column 00G-4252-P0-AX, 5 μm, 100 Å, 250 × 21.2 mm (flow rate 10 mL/min, max. loading 100 mg crude) coupled to a Thermo Fisher Scientific Dionex Ultimate 3000 HPLC-System using a ternary solvent mixture (MeCN/H_2_O + 0.1% CF_3_ COOH). Product-containing fractions were identified by LCMS and lyophilized to dryness. The purity of final compounds was measured on SHIMADZU HPLC (model number: CBM-20A) with Gemini 3 μm NX-C18-110 Å, LC-column 50 × 2 m.m (flow rate 1 mL/min). For nuclear magnetic resonance (NMR) spectroscopic analysis, Bruker AVANCE III or Bruker AVANCE III HD spectrometers were employed and ^1^H NMR spectra were recorded at 500 MHz or 700 MHz, respectively, and 126 and 176 MHz for ^13^C NMR spectra. Chemical shifts are reported in parts per million (ppm) using the residual non-deuterated solvent resonance for proton measurements and the solvent resonance for carbon measurements as internal standards (D_3_COD: ^1^H = 3.31 ppm and ^13^C = 49.00 ppm; DMSO-*d*_6_: ^1^H = 2.50 ppm and ^13^C = 39.52 ppm, CD_3_CN: ^1^H = 1.94 ppm and ^13^C = 1.32, 118.26 ppm). Data are reported as follows: chemical shift (multiplicity (s = singlet, d = doublet, t = triplet, q = quartet, m = multiplet), coupling constant(s) in Hz, integration). High-resolution mass spectrometry was performed using a Dionex Ultimate 3000 HPLC system equipped with a DAD detector and a Bruker maXis HD QTOF mass detector with electrospray ionization (ESI). Samples were injected via an Ultimate 3000RS autosampler (Thermo Fisher Scientific), and data are reported in the form of mass-to-charge ratio (*m*/*z*). SFC purity analyses were conducted using a Waters ACQUITY UPC2 system with Binary Solvent Manager (K17C2B854M), Sample Manager (L17C2S782M), Convergence Manager (K17C2M838M), Column Manager (E18AZ3268M), and PDA Detector (M17C2P349A). Data were collected and processed using Empower 3 Build 3471 software. All the final compounds used for biological studies were purified using prep-HPLC. All compounds are >95% pure by HPLC analysis or supercritical fluid chromatography (SFC) analysis (exception: **16c** and **9b** had >94% purity).

#### Synthesis of Compounds

##### Synthesis of (3*S*)-3-((*S*)-2-(3-((*tert*-Butoxycarbonyl)amino)-2-oxopyridin-1(2*H*)-yl)-3-cyclopropylpropanamido)-1-(methyl amino)-1-oxo-4-((*S*)-2-oxopyrrolidin-3-yl)butan-2-yl acetate (**5a**)

To a stirred solution of *tert*-butyl (1-((S)-3-cyclopropyl-1-oxo-1-(((S)-1-oxo-3-((S)-2-oxopyrrolidin-3-yl)propan-2-yl)amino)propan-2-yl)-2-oxo-1,2-dihydropyridin-3-yl)carbamate (**4a**) (600 mg, 1.304 mmol) and acetic acid (156 mg, 2.6 mmol) in CH_2_Cl_2_ (30 mL) at ambient temperature was added methyl isocyanide (82 mg, 1.96 mmol) and the reaction mixture was stirred for 22 h at room temperature (rt) before being concentrated under reduced pressure. Purification by flash column chromatography, eluting with 3–5% of CH_3_OH/CH_2_Cl_2_, afforded the title compound as an off-white solid. Yield: 651 mg, 89%, ^1^H NMR (700 MHz, CD_3_CN, 298 K): δ 7.97–7.84 (m, 1H), 7.67–7.52 (m, 1H), 7.46–6.94 (m, 2H), 6.89–6.62 (m, 1H), 6.39–6.19 (m, 1H), 6.16–5.93 (m, 1H), 5.55–5.27 (m, 1H), 5.24–4.85 (m, 1H), 4.50 (tdd, *J* = 9.4, 7.5, 3.6 Hz, 1H), 4.41–4.25 (m, 1H), 3.35–3.01 (m, 2H), 2.73–2.43 (m, 4H), 2.16–1.96 (m, 4H), 1.90–1.53 (m, 3H), 1.53–1.46 (s, 9H), 1.45–1.23 (m, 2H), 0.59–0.52 (m, 1H), 0.44–0.30 (m, 2H), 0.16 to −0.07 (m, 2H). ESI-HRMS: calcd for C_27_H_40_N_5_O_8_ [M + H]^+^, *m*/*z* = 562.2876 Da; found, *m*/*z* = 562.2876 Da.

##### (3*S*)-3-((*S*)-2-(3-((*tert*-Butoxycarbonyl)amino)-2-oxopyridin-1(2*H*)-yl)-3-cyclopropylpropanamido)-1-(isopropylamino)-1-oxo-4-((*S*)-2-oxopyrrolidin-3-yl) Butan-2-yl Acetate (**5b**)

Following the procedure described for the preparation of compound **5a** with a slight modification of conducting the reaction with acetic acid (13.2 μL, 0.23 mmol) and isopropyl isocyanide (15.8 mg, 0.23 mmol) at rt afforded the acetoxy amide **5b** (63 mg, 92%) as a white solid. ^1^H NMR (500 MHz, CDCl_3_, 298 K): δ 8.06–7.92 (m, 1H), 7.69–7.57 (m, 1H), 7.19–7.08 (m, 1H), 6.48–6.09 (m, 2H), 5.82–5.59 (m, 1H), 5.57–5.04 (m, 2H), 4.52–4.29 (m, 1H), 4.14–3.95 (m, 1H), 3.39–3.08 (m, 2H), 2.63–2.26 (m, 2H), 2.22–1.73 (m, 7H), 1.51 (m, 9H), 1.47–1.02 (m, 7H), 0.64–0.51 (m, 1H), 0.47–0.37 (m, 2H), 0.18–0.09 (m, 1H), 0.08 to −0.06 (m, 1H). ESI-MS (*m*/*z*): 590 Da [M + H]^+^.

##### (3*S*)-3-((*S*)-2-(3-((*tert*-Butoxycarbonyl)amino)-2-oxopyridin-1(2*H*)-yl)-3-cyclopropylpropanamido)-1-(cyclopropylamino)-1-oxo-4-((*S*)-2-oxopyrrolidin-3-yl) Butan-2-yl Acetate (**5c**)

Following the procedure described for the preparation of compound **5a** with a slight modification of conducting the reaction with acetic acid (17.4 μL, 0.305 mmol) and cyclopropyl isocyanide (20.4 mg, 0.304 mmol) at rt afforded the acetoxy amide **5c** (79.4 mg, 89%) as a white solid.^1^H NMR (500 MHz, CDCl_3_, 298 K): δ 8.04–7.98 (m, 1H), 7.83–7.53 (m, 2H), 7.25–7.12 (m, 1H), 7.05–6.55 (m, 2H), 6.35–6.27 (m, 1H), 5.55–5.36 (m, 1H), 5.31–5.02 (m, 1H), 4.44–4.33 (m, 1H), 3.49–3.29 (m, 2H), 2.79–2.28 (m, 3H), 2.21–1.59 (m, 7H), 1.52 (m, 9H), 0.86–0.37 (m, 7H), 0.21–0.1 (m, 1H), 0.081 to −0.07 (m, 1H). ESI-MS (*m*/*z*): 588 Da [M + H]^+^.

##### (3*S*)-3-((*S*)-2-(3-((*tert*-Butoxycarbonyl)amino)-5-fluoro-2-oxopyridin-1(2*H*)-yl)-3-cyclopropylpropanamido)-1-(methylamino)-1-oxo-4-((**S**)-2-oxopyrrolidin-3-yl)butan-2-yl Acetate (**5d**)

Following the procedure described for the preparation of compound **5a** with a slight modification of conducting the reaction with **4b** and acetic acid (24.8 μL, 0.434 mmol) and methyl isocyanide (17.36 mg, 0.434 mmol) at rt afforded the acetoxy amide **5d** (103 mg, 85%) as a white solid. ^1^H NMR (700 MHz, CD_3_CN, 298 K): δ 8.24–8.07 (m, 1H), 7.89–7.41 (m, 2H), 7.23–7.02 (m, 1H), 6.76–6.35 (m, 1H), 6.06–5.82 (m, 1H), 5.35–4.88 (m, 2H), 4.45–4.22 (m, 1H), 3.25–2.99 (m, 2H), 2.77–2.44 (m, 3H), 2.30–1.99 (m, 3H), 1.94–1.59 (m, 5H), 1.59–1.46 (m, 9H), 1.47–1.18 (m, 2H), 0.93–0.79 (m, 1H), 0.54–0.35 (m, 2H), 0.22–0.06 (m, 2H). ESI-MS (*m*/*z*): 580 Da [M + H]^+^.

##### *tert*-Butyl (1-((*S*)-3-Cyclopropyl-1-(((*S*)-4-(methylamino)-3,4-dioxo-1-((*S*)-2-oxopyrrolidin-3-yl)butan-2-yl)amino)-1-oxopropan-2-yl)-2-oxo-1,2-dihydropyridin-3-yl)carbamate (**6a**)

To the stirred solution of compound **5a** (33 mg, 0.055 mmol) in aq CH_3_OH, LiOH·H_2_O (4.67 mg, 0.111 mmol) was added and stirred at rt for 1.5 h. The progress of the reaction was checked on TLC and LCMS. After completion of the reaction, it was concentrated, diluted with 10 mL of CH_2_Cl_2_, and washed with 10% NaHCO_3_. The resulting organic was evaporated to dryness which gave (24.5 mg, 80%) of a hydroxyl product as a white solid. ESI-MS (*m*/*z*): 520 Da [M + H]^+^. Dess–Martin periodinane (DMP) (37.7 mg, 0.0898 mmol) was added portion-wise to a solution of the above hydroxy amide compound (24.5 mg, 0.0445 mmol) in CH_2_Cl_2_ (5 mL). The mixture was stirred at rt for 2 h. After completion of the reaction, it was treated with few drops of a Na_2_S_2_O_3_ solution and concentrated. The product was further purified on reverse-phase preparative HPLC which afforded α-ketoamide **6a** (14 mg, 61%) as a white solid. d.r. ∼ 1:1 (*S*,*S*,*S*): (*R*,*S*,*S*), HPLC purity: 98.8%, ^1^H NMR (500 MHz, CD_3_CN, 298 K): δ 8.57, 8.40 (d, *J* = 5.3 Hz, 0.5H; d, *J* = 5.0 Hz, 0.5H), 7.95–7.85 (m, 1H), 7.55 (d, *J* = 5.4 Hz, 1H), 7.27 (d, *J* = 21.9 Hz, 1H), 7.16 (td, *J* = 6.9, 1.7 Hz, 1H), 6.30–6.22 (m, 1H), 6.13 (d, *J* = 32.4 Hz, 1H), 5.58, 5.48 (dd, *J* = 9.0, 6.6 Hz, 0.5H; dd, *J* = 9.3, 6.1 Hz, 0.5H), 5.11–5.04 (m, 1H), 3.22 (ddd, *J* = 15.4, 9.5, 5.4 Hz, 2H), 2.77–2.69 (m, 3H), 2.53–2.46 (m, 1H), 2.41–2.22 (m, 2H), 2.10–1.68 (m, 5H), 1.49 (s, 9H), 0.62–0.52 (m, 1H), 0.41–0.30 (m, 2H), 0.09 (ddd, *J* = 16.2, 9.8, 5.0 Hz, 1H), −0.01 (ddd, *J* = 12.7, 8.8, 4.6 Hz, 1H). ^13^C NMR (126 MHz, CD_3_CN, 298 K): δ 196.40, 180.28, 170.16, 161.43, 153.51, 130.05, 128.54, 119.77, 106.65, 81.27, 59.12, 54.56, 40.98, 40.93, 39.74, 39.46, 36.33, 36.02, 32.07, 29.38, 28.32, 25.84, 8.26, 4.87, 4.35. ESI-HRMS: calcd for C_25_H_36_N_5_O_7_ [M + H]^+^, *m*/*z* = 518.2614 Da; found, *m*/*z* = 518.2610 Da.

##### *tert*-Butyl (1-((*S*)-3-Cyclopropyl-1-(((*S*)-4-(isopropylamino)-3,4-dioxo-1-((*S*)-2-oxopyrrolidin-3-yl)butan-2-yl)amino)-1-oxopropan-2-yl)-2-oxo-1,2-dihydropyridin-3-yl)carbamate (**6b**)

Compound **6b** was prepared in two steps in a 58% yield by deacylation of acetoxy amide **5b** (60 mg, 0.1018 mmol) followed by oxidation with DMP (38.7 mg, 0.091 mmol) utilizing the same procedure as described for compound **6a**. d.r. ∼ 1:1 (*S*,*S*,*S*): (*R*,*S*,*S*), HPLC purity: 95.9%, ^1^H NMR (500 MHz, CD_3_CN, 298 K): δ 8.53–8.46 (m, 1H), 7.87 (d, *J* = 7.2 Hz, 1H), 7.55 (s, 1H), 7.20–7.06 (m, 2H), 6.25 (td, *J* = 7.2, 2.1 Hz, 1H), 6.12 (d, *J* = 24.1 Hz, 1H), 5.61–5.43 (m, 1H), 5.24–5.03 (m, 1H), 3.95 (ddq, *J* = 18.2, 15.0, 6.6 Hz, 1H), 3.27–3.16 (m, 2H), 2.53–2.45 (m, 1H), 2.38–2.2 (m, 2H), 2.18–1.67 (m, 5H), 1.49 (s, 9H), 1.16–1.06 (m, 6H), 0.61–0.52 (m, 1H), 0.42–0.32 (m, 2H), 0.13 (dd, *J* = 8.0, 4.0 Hz, 1H), 0.02 to −0.04 (m, 1H)·^13^C NMR (176 MHz, CD_3_CN, 298 K): δ 196.19, 179.31, 169.22, 159.19, 152.59, 129.01, 127.60, 105.73, 80.37, 55.25, 53.67, 53.04, 41.45, 40.04, 38.79, 38.48, 37.68, 35.41, 31.27, 30.47, 27.41, 21.26, 7.35, 3.96, 3.43. ESI-HRMS: calcd for C_27_H_40_N_5_O_7_ [M + H]^+^, *m*/*z* = 546.2927 Da; found, *m*/*z* = 546.2927 Da.

##### *tert*-Butyl (1-((*S*)-3-Cyclopropyl-1-(((*S*)-4-(cyclopropylamino)-3,4-dioxo-1-((*S*)-2-oxopyrrolidin-3-yl)butan-2-yl)amino)-1-oxopropan-2-yl)-2-oxo-1,2-dihydropyridin-3-yl)carbamate (**6c**)

Compound **6c** was prepared in two steps in a 63% yield by deacylation of acetoxy amide **5c** (60 mg, 0.1022 mmol) followed by oxidation with DMP (56 mg, 0.132 mmol) utilizing the same procedure as described for compound **6a**. d.r. ∼ 1:1 (*S*,*S*,*S*): (*R*,*S*,*S*), HPLC purity: 99.3%, ^1^H NMR (700 MHz, CD_3_CN, 298 K): δ 8.43–8.20 (d, *J* = 5.1 Hz, 0.5H, d, *J* = 5.1 Hz, 0.5H), 7.78 (s, 1H), 7.46 (d, *J* = 6.3 Hz, 1H), 7.20 (d, *J* = 34.2 Hz, 1H), 7.07 (ddd, *J* = 10.3, 7.1, 1.7 Hz, 1H), 6.17 (dd, *J* = 16.7, 9.4 Hz, 1H), 6.07–6.00 (m, 1H), 5.49–5.38 (dd, *J* = 8.9, 6.6 Hz; dd, *J* = 9.1, 6.2 Hz, 0.5H), 4.98 (ddd, *J* = 10.2, 6.6, 4.2 Hz, 1H), 3.18–3.07 (m, 2H), 2.64–2.57 (m, 1H), 2.42–2.11 (m, 4H), 1.93–1.61 (m, 4H), 1.40 (s, 9H), 0.69–0.58 (m, 2H), 0.55–0.42 (m, 2H), 0.33–0.22 (m, 2H), 0.03 to −0.13 (m, 2H). ^13^C NMR (176 MHz, CD_3_CN, 298 K): δ 195.91, 179.90, 169.83, 161.86, 157.79, 153.17, 129.72, 127.92, 119.48, 106.32, 80.94, 58.82, 54.13, 40.61, 39.33, 35.98, 31.82, 28.98, 28.88, 27.97, 22.77, 7.91, 5.84, 4.52, 4.01. ESI-HRMS: calcd for C_27_H_37_N_5_O_7_ [M + H]^+^, *m*/*z* = 544.2771 Da; found, *m*/*z* = 544.2765 Da.

##### *tert*-Butyl (1-((*S*)-3-Cyclopropyl-1-(((*S*)-4-(methylamino)-3,4-dioxo-1-((*S*)-2-oxopyrrolidin-3-yl)butan-2-yl)amino)-1-oxopropan-2-yl)-5-fluoro-2-oxo-1,2-dihydropyridin-3-yl)carbamate (**6d**)

Compound **6d** was prepared in two steps in a 63% yield by deacylation of acetoxy amide **5d** (140 mg, 0.2417 mmol) followed by oxidation with DMP (128 mg, 0.301 mmol) utilizing the same procedure as described for compound **6a**. d.r. ∼ 1:1 (*S*,*S*,*S*): (*R*,*S*,*S*), HPLC purity: 99.1%, ^1^H NMR (500 MHz, DMSO-*d*_6_, 298 K): δ 9.03–8.93 (m, 1H), 8.69–8.58 (m, 1H), 8.00 (t, *J* = 5.2 Hz, 1H), 7.86–7.80 (m, 1H), 7.75–7.67 (m, 1H), 7.57–7.49 (m, 1H), 5.68 (dd, *J* = 9.8, 5.3 Hz, 0.4H), 5.62–5.55 (m, 0.6H), 5.14–5.08 (m, 0.4H), 5.02–4.95 (m, 0.7H), 3.23–2.95 (m, 3H), 2.64 (dt, *J* = 10.1, 4.9 Hz, 2H), 2.41–2.10 (m, 3H), 2.07–1.57 (m, 5H), 1.48 (s, 9H), 0.56–0.44 (m, 1H), 0.38–0.28 (m, 2H), 0.17–0.10 (m, 1H), 0.02 to −0.05 (m, 1H). ^13^C NMR (176 MHz, DMSO-*d*_6_, 298 K): δ 196.59, 178.37, 169.52, 161.69, 155.40, 152.47, 147.10, 145.93, 128.95, 114.43, 111.79, 81.26, 58.84, 53.09, 39.99, 35.73, 31.58, 28.29, 27.60, 25.90, 7.95, 5.09, 4.90, 3.98, ESI-HRMS: calcd for C_25_H_35_FN_5_O_7_ [M + H]^+^, *m*/*z* = 536.2520 Da; found, *m*/*z* = 536.2515 Da.

##### *tert*-Butyl (1-((*S*)-3-Cyclopropyl-1-(((*S*)-4-(methylamino)-3,4-dioxo-1-((*S*)-2-oxopyrrolidin-3-yl)butan-2-yl)amino)-1-oxopropan-2-yl)-5-fluoro-2-oxo-1,2-dihydropyridin-3-yl)carbamate (**6d-K** and **6d-H**)

Compounds **6d-K** and **6d-H** were prepared in two steps in a 49% yield by deacylation of acetoxy amide **5d** (130 mg, 0.224 mmol) followed by separation using prep-HPLC with the method described above. The isolated fractions were oxidized individually with DMP utilizing the same procedure as described for compound **6a** (Supporting Information S13).

**6d-K**^1^H NMR (700 MHz, DMSO-*d*_6_, 298 K): δ 8.94 (d, *J* = 7.0 Hz, 1H), 8.63 (d, *J* = 4.9 Hz, 1H), 8.00 (s, 1H), 7.81 (dd, *J* = 14.5, 5.2 Hz, 1H), 7.68 (s, 1H), 7.52 (d, *J* = 34.8 Hz, 1H), 5.63–5.56 (m, 1H), 5.01–4.94 (m, 1H), 3.22–3.05 (m, 2H), 2.69–2.55 (m, 3H), 2.34–2.25 (m, 1H), 2.14 (dd, *J* = 18.3, 9.4 Hz, 1H), 2.00–1.76 (m, 3H), 1.65 (dt, *J* = 24.4, 11.4 Hz, 2H), 1.46 (s, 9H), 0.57–0.46 (m, 1H), 0.37–0.29 (m, 2H), 0.17–0.11 (m, 1H), −0.00 (t, *J* = 10.9 Hz, 1H). ^13^C NMR (176 MHz, DMSO-*d*_6_, 298 K): 196.12, 177.90, 169.02, 160.94, 154.90, 152.00, 146.68, 145.41, 128.47, 113.74, 111.35, 80.76, 58.37, 52.62, 40.02, 37.84, 34.99, 30.85, 27.82, 27.08, 25.43, 7.45, 4.62, 3.51. ESI-HRMS: calcd for C_25_H_35_FN_5_O_7_ [M + H]^+^, *m*/*z* = 536.2520 Da; found, *m*/*z* = 536.2515 Da.

**6d-H**^1^H NMR (700 MHz, DMSO-*d*_6_, 298 K): δ 8.99 (d, *J* = 6.8 Hz, 1H), 8.61 (t, *J* = 7.2 Hz, 1H), 8.03–7.95 (m, 1H), 7.86–7.77 (m, 1H), 7.68 (s, 1H), 7.57–7.47 (m, 1H), 5.73–5.64 (m, 1H), 5.04–4.96 (m, 1H), 3.22–3.10 (m, 2H), 2.70–2.58 (m, 3H), 2.35 (t, *J* = 21.6 Hz, 1H), 2.23–2.13 (m, 1H), 2.03–1.95 (m, 1H), 1.95–1.84 (m, 1H), 1.78–1.59 (m, 3H), 1.47 (s, 9H), 0.49 (t, *J* = 18.4 Hz, 1H), 0.35–0.27 (m, 2H), 0.13 (dd, *J* = 19.5, 11.9 Hz, 1H), 0.02 to −0.05 (m, 1H). ^13^C NMR (151 MHz, DMSO-*d*_6_, 298 K): δ 195.98, 177.75, 168.78, 160.98, 154.88, 146.73, 145.24, 128.48, 113.90, 111.11, 80.78, 57.80, 52.47, 37.83, 35.26, 31.11, 27.82, 27.13, 25.42, 7.48, 4.43, 3.45. ESI-HRMS: calcd for C_25_H_35_FN_5_O_7_ [M + H]^+^, *m*/*z* = 536.2520 Da; found, *m*/*z* = 536.2514 Da.

##### *tert*-Butyl (1-(1-(((2*S*)-1-Cyano-1-hydroxy-3-((*S*)-2-oxopyrrolidin-3-yl)propan-2-yl)amino)-3-cyclopropyl-1-oxopropan-2-yl)-2-oxo-1,2-dihydropyridin-3-yl)carbamate (**7a**)

To a stirred solution of *tert*-butyl (1-(3-cyclopropyl-1-oxo-1-(((*S*)-1-oxo-3-((S)-2-oxopyrrolidin-3-yl)propan-2-yl)amino)propan-2-yl)-2-oxo-1,2-dihydropyridin-3-yl)carbamate (**4a**) (117 mg, 0.254 mmol) in CH_2_Cl_2_ (5 mL) at 0 °C were added acetone cyanohydrin (33 mg, 0.381 mmol) and triethylamine (52 mg, 0.514 mmol), and the resulting mixture was stirred for 2 h at ambient temperature. The reaction mixture was diluted with 10 mL of CH_2_Cl_2_ and was washed with water and brine solution. The resulting organic solution of CH_2_Cl_2_ was dried over anhydrous sodium sulfate, concentrated, and purified over flash column chromatography, eluting with 2–5% of MeOH/CH_2_Cl_2_, affording the title compound as a white solid (101 mg, 82%). ^1^H NMR (700 MHz, DMSO-*d*_6_, 298 K): δ 8.67–8.28 (m, 1H), 7.86–7.70 (m, 2H), 7.66–7.54 (m, 1H), 7.46–7.33 (m, 1H), 6.80–6.63 (m, 1H), 6.29 (tt, *J* = 7.5, 5.6 Hz, 1H), 5.68–5.50 (m, 1H), 4.63–4.34 (m, 1H), 4.19–3.93 (m, 1H), 3.24–2.97 (m, 2H), 2.30–1.73 (m, 5H), 1.71–1.33 (m, 2H), 1.46 (d, *J* = 2.0 Hz, 9H), 0.55–0.43 (m, 1H), 0.37–0.26 (m, 2H), 0.19–0.08 (m, 1H), 0.03 to −0.07 (m, 1H). ESI-MS (*m*/*z*): 488 Da [M + H]^+^.

##### *tert*-Butyl (1-(1-(((2*S*)-1-Cyano-1-hydroxy-3-((*S*)-2-oxopyrrolidin-3-yl) Propan-2-yl)amino)-3-cyclopropyl-1-oxopropan-2-yl)-5-fluoro-2-oxo-1,2-dihydropyridin-3-yl)carbamate (**7b**)

To a stirred solution of *tert*-butyl (1-(3-cyclopropyl-1-oxo-1-(((*S*)-1-oxo-3-((*S*)-2-oxopyrrolidin-3-yl)propan-2-yl)amino)propan-2-yl)-5-fluoro-2-oxo-1,2-dihydropyridin-3-yl)carbamate **4b** (1.97 g, 4.12 mmol) in methanol (7.8 mL) at 0 °C was added an ice-cooled solution of sodium bisulfite (0.45 g, 4.28 mmol) in water (11.7 mL), and the resulting mixture was stirred for 2 h. After this time, an ice-cooled solution of sodium cyanide (0.32 g, 6.52 mmol) in a mixture of water (7.8 mL) and ethyl acetate (23.4 mL) was added, and stirring was maintained at 0 °C for 2 h before warming to ambient temperature with stirring overnight. The organic phase was separated, and the aqueous component was extracted with ethyl acetate (2 × 20 mL). The combined organic extracts were washed with brine (20 mL), dried over anhydrous magnesium sulfate, and concentrated under reduced pressure, affording the title compound as an off-white solid (1.86 g, 89%) which was used directly in the next step without further purification. ^1^H NMR (400 MHz DMSO-*d*_6_, 298 K): δ 8.65–8.42 (m, 1H), 8.02–7.96 (m, 1H), 7.86–7.79 (m, 1H), 7.66–7.51 (m, 2H), 6.76–6.66 (m, 1H), 5.66–5.47 (m, 1H), 4.61–4.34 (m, 1H), 4.02–3.89 (m, 1H), 3.20–2.99 (m, 2H), 2.24–1.69 (m, 5H), 1.64–1.40 (m, 1H), 1.47 (s, 9H), 0.59–0.42 (m, 1H), 0.39–0.25 (m, 2H), 0.20–0.09 (m, 1H), 0.04 to −0.05 (m, 1H). ESI^+^-MS (*m*/*z*): 528.3 Da [M + Na^+^]^+^.

##### *tert*-Butyl (1-(1-(((2*S*)-1-Cyano-1-hydroxy-3-((*S*)-2-oxopyrrolidin-3-yl) Propan-2-yl) amino)-3-cyclopropyl-1-oxopropan-2-yl)-6-oxo-1,6-dihydropyrimidin-5-yl)carbamate (**7c**)

Following the procedure described for the preparation of compound **7b** with a slight modification of conducting the reaction with compound **4c** (0.45 g, 0.98 mmol) and sodium cyanide (0.08 g, 1.55 mmol) afforded compound **7c** (0.42 g, 88%) as an off-white solid. ^1^H NMR (400 MHz, DMSO-*d*_6_, 298 K): δ 8.65 (d, *J* = 8.4 Hz, 1H), 8.32–8.23 (m, 2H), 8.02–7.93 (m, 1H), 7.67–7.55 (m, 1H), 6.77–6.66 (m, 1H), 5.54–5.39 (m, 1H), 4.61–4.39 (m, 1H), 3.99 (m, 1H), 3.13 (m, 1H), 2.27–1.81 (m, 5H), 1.45 (s, 9H), 0.60–0.45 (m, 1H), 0.37–0.28 (m, 2H), 0.19–0.10 (m, 1H), 0.02 to −0.06 (m, 1H). ESI^+^-MS (*m*/*z*): 511.3 Da [M + Na^+^]^+^: 489.3 Da [M + H]^+^.

##### *tert*-Butyl (4-(1-(((2S)-1-Cyano-1-hydroxy-3-((S)-2-oxopyrrolidin-3-yl) Propan-2-yl) amino)-3-cyclopropyl-1-oxopropan-2-yl)-3-oxo-3,4-dihydropyrazin-2-yl)carbamate (**7d**)

Following the procedure described for the preparation of compound **7b** with a slight modification of conducting the reaction with compound **4d** (1.00 g, 2.17 mmol) and sodium cyanide (0.17 g, 3.42 mmol) afforded compound **7d** (0.97 g, 92%) as an off-white solid. ^1^H NMR (400 MHz, DMSO-*d*_6_, 298 K): δ 8.69–8.59 and 8.54–8.46 (2m, 2H), 7.66–7.52 (m, 1H), 7.39–7.33 (m, 1H), 7.07–7.02 (m, 1H), 6.75–6.67 (m, 1H), 5.55–5.37 (m, 1H), 4.61–4.54 and 4.43–4.35 (2m, 1H), 4.01–3.92 (m, 1H), 3.19–3.02 (m, 2H), 2.25–1.70 (m, 5H), 1.61–1.41 (m, 11H), 0.60–0.43 (m, 1H), 0.40–0.26 (m, 2H), 0.19–0.10 (m, 1H), 0.05–0.03 (m, 1H). ESI^+^-MS (*m*/*z*): 389.3 Da [M + H-Boc]^+^.

##### *tert*-Butyl (1-(1-(((2*S*)-4-Amino-3-hydroxy-4-oxo-1-((*S*)-2-oxopyrrolidin-3-yl)-butan-2-yl)amino)-3-cyclopropyl-1-oxopropan-2-yl)-2-oxo-1,2-dihydropyridin-3-yl)¬carb-amate (**8a**)

To a stirred solution of *tert*-butyl (1-(1-(((2*S*)-1-cyano-1-hydroxy-3-((*S*)-2-oxopyrrolidin-3-yl)propan-2-yl)amino)-3-cyclopropyl-1-oxopropan-2-yl)-2-oxo-1,2-dihydropyridin-3-yl)carbamate **7a** (100 mg, 0.205 mmol) in methanol (3 mL) at 0 °C were added 30% hydrogen peroxide solution (0.2 mL, 2.05 mmol) and lithium hydroxide monohydrate (17.3 mg, 0.412 mmol), and the resulting mixture was stirred for 3.5 h. After this time, the reaction mixture was treated with a 5% aqueous sodium meta-bisulfite solution (2 mL), and after stirring for a further 10 min, CH_2_Cl_2_ (10 mL) was added and the organic phase was separated. The aqueous component was extracted with CH_2_Cl_2_ (6 × 5 mL), and the combined organic extracts were dried over anhydrous sodium sulfate and concentrated under reduced pressure. Purification by flash column chromatography, eluting with 3–7% MeOH/CH_2_Cl_2_, afforded the title compound as a white solid (70 mg, 68%). ^1^H NMR (700 MHz, DMSO-*d*_6_, 298 K): δ 8.31–8.09 (m, 1H), 7.89–7.80 (m, 1H), 7.77–7.51 (m, 1H), 7.51–7.33 (m, 1H), 7.31–7.14 (m, 2H), 6.28 (tt, *J* = 8.5, 4.3 Hz, 1H), 5.73–5.56 (m, 2H), 4.27–4.07 (m, 1H), 3.90–3.75 (m, 1H), 3.20–2.97 (m, 2H), 2.20–1.90 (m, 3H), 1.86–1.52 (m, 2H), 1.50–1.43 (m, 9H), 1.42–1.33 (m, 1H), 1.08–0.91 (m, 1H), 0.89–0.69 (m, 1H), 0.55–0.39 (m, 1H), 0.39–0.24 (m, 2H), 0.20–0.10 (m, 1H), −0.03 (qd, *J* = 10.4, 5.6 Hz, 1H). ESI-MS (*m*/*z*): 506 Da [M + H]^+^.

##### *tert*-Butyl (1-(1-(((2*S*)-4-amino-3-hydroxy-4-oxo-1-((*S*)-2-oxopyrrolidin-3-yl) Butan-2-yl)amino)-3-cyclopropyl-1-oxopropan-2-yl)-5-fluoro-2-oxo-1,2-dihydropyridin-3-yl)carbamate (**8b**)

Following the procedure described for the preparation of compound **8a** with a slight modification of conducting the reaction with compound **7b** (0.90 g, 1.78 mmol), 30% hydrogen peroxide solution (1.8 mL, 17.80 mmol), and lithium hydroxide monohydrate (0.11 g, 2.67 mmol) afforded the hydroxy amide **8b** (0.44 g, 48%) as a white solid. ^1^H NMR (400 MHz, DMSO-*d*_6_, 298 K): δ 7.99 (s, 1H), 7.82 (dd, *J*_1_ = 10.0 Hz, *J*_2_ = 2.8 Hz, 1H), 7.56 (m, 1H), 7.52 (m, 1H), 7.22 (s, 1H), 5.71–5.55 (m, 2H), 4.09 (m, 1H), 3.88–3.76 (m, 1H), 3.17–2.96 (m, 2H), 2.16–1.88 (m, 4H), 1.83–1.70 (m, 1H), 1.64–0.98 (m, 2H), 1.47 (s, 9H), 0.55–0.41 (m, 1H), 0.37–0.24 (m, 2H), 0.20–0.11 (m, 1H), 0.03 to −0.06 (m, 1H). ESI^–^-MS (*m*/*z*): 522.1 Da [M – H]^−^.

##### *tert*-Butyl (1-(1-(((2*S*)-4-Amino-3-hydroxy-4-oxo-1-((*S*)-2-oxopyrrolidin-3-yl) Butan-2-yl) amino)-3-cyclopropyl-1-oxopropan-2-yl)-6-oxo-1,6-dihydropyrimidin-5-yl)carbamate (**8c**)

Following the procedure described for the preparation of compound **8a** with a slight modification of conducting the reaction with compound **7c** (0.41 g, 0.84 mmol), a 30% hydrogen peroxide solution (0.86 mL, 8.39 mmol), and lithium hydroxide monohydrate (0.05 g, 1.26 mmol) afforded the hydroxy amide **8c** (0.14 g, 33%) as a white solid. ^1^H NMR (400 MHz, DMSO-*d*_6_, 298 K): δ 8.38–7.91 (m, 4H), 7.59–7.48 (m, 1H), 7.26–7.01 (m, 2H), 5.73–5.47 (m, 2H), 4.10 (m, 1H), 3.90–3.77 (m, 1H), 3.18–2.98 (m, 2H), 2.22–1.81 (m, 5H), 1.66–0.97 (m, 2H), 1.45 (s, 9H), 0.56–0.43 (m, 1H), 0.37–0.25 (m, 2H), 0.20–0.12 (m, 1H), 0.02 to −0.07 (m, 1H). ESI^+^-MS (*m*/*z*): 507.3 Da [M + H]^+^.

##### *tert*-Butyl (4-(1-(((2*S*)-4-Amino-3-hydroxy-4-oxo-1-((*S*)-2-oxopyrrolidin-3-yl)butan-2-yl)amino)-3-cyclopropyl-1-oxopropan-2-yl)-3-oxo-3,4-dihydropyrazin-2-yl)carbamate (**8d**)

Following the procedure described for the preparation of compound **8a** with a slight modification of conducting the reaction with compound **7d** (0.97 g, 1.99 mmol), a 30% hydrogen peroxide solution (2.03 mL, 19.9 mmol), and lithium hydroxide monohydrate (0.13 g, 2.98 mmol) afforded the hydroxy amide **8d** (0.34 g, 34%) as a white solid. ^1^H NMR (400 MHz, DMSO-*d*_6_, 298 K): δ 8.68–8.60 (m, 1H), 8.34–8.15 and 8.00–7.94 (2m, 1H), 7.65–7.47 (m, 1H), 7.44–7.32 (m, 1H), 7.25–7.14 (m, 1H), 7.06–7.00 (m, 1H), 5.75–5.43 (m, 2H), 4.25–4.05 (m, 1H), 3.90–3.78 (m, 1H), 3.19–2.99 (m, 2H), 2.22–1.70 (m, 5H), 1.65–0.95 (m, 11H), 0.56–0.42 (m, 1H), 0.38–0.25 (m, 2H), 0.19–0.11 (m, 1H), 0.04 to −0.05 (m, 1H). ESI^–^-MS (*m*/*z*): 505.3 Da [M – H]^−^.

##### *tert*-Butyl (1-((*S*)-1-(((*S*)-4-Amino-3,4-dioxo-1-((*S*)-2-oxopyrrolidin-3-yl)butan-2-yl)amino)-3-cyclopropyl-1-oxopropan-2-yl)-2-oxo-1,2-dihydropyridin-3-yl)carbamate (**9a**)

The α-hydroxy amide **8a** (65 mg, 0.128 mmol) was dissolved in DMF (3 mL), and DMP (110 mg, 0.259 mmol) was added. The mixture was stirred at ambient temperature for 3 h. After completion of the reaction, it was treated with few drops of a Na_2_S_2_O_3_ solution and concentrated. Purification by reverse-phase prep HPLC afforded the title compound as a white solid (20 mg, 31%). d.r. ∼ 1:1 (*S*,*S*,*S*): (*R*,*S*,*S*), HPLC purity: 96.8%, ^1^H NMR (700 MHz, DMSO-*d*_6_, 298 K): δ 8.93 (ddd, *J* = 23.9, 15.9, 7.0 Hz, 1H), 8.01 (dd, *J* = 18.6, 13.4 Hz, 1H), 7.84–7.66 (m, 3H), 7.39–7.34 (m, 1H), 6.31–6.25 (m, 1H), 5.74–5.60 (m, 1H), 5.15–4.97 (m, 1H), 3.12 (tdt, *J* = 16.1, 9.3, 8.0 Hz, 2H), 2.38–2.07 (m, 3H), 2.01–1.52 (m, 5H), 1.46 (s, 9H), 0.48 (dtd, *J* = 20.0, 12.6, 7.3 Hz, 1H), 0.39–0.26 (m, 2H), 0.17–0.09 (m, 1H), 0.03 to −0.06 (m, 1H). ^13^C NMR (176 MHz, DMSO-*d*_6,_ 298 K): δ 197.02, 178.38, 169.67, 163.36, 158.66, 157.22, 152.65, 129.29, 128.37, 120.37, 105.33, 80.56, 57.83, 53.42, 38.58, 36.19, 31.60, 28.59, 27.65, 8.02, 5.10, 4.89, 4.05. ESI-HRMS: calcd for C_24_H_34_N_5_O_7_ [M + H]^+^, *m*/*z* = 504.2458 Da; found, *m*/*z* = 504.2451 Da.

##### *tert*-Butyl (1-(1-(((*S*)-4-Amino-3,4-dioxo-1-((*S*)-2-oxopyrrolidin-3-yl)butan-2-yl)amino)-3-cyclopropyl-1-oxopropan-2-yl)-5-fluoro-2-oxo-1,2-dihydropyridin-3-yl)carbamate (**9b**)

A stirred solution of *tert*-butyl (1-(1-(((2*S*)-4-amino-3-hydroxy-4-oxo-1-((*S*)-2-oxopyrrolidin-3-yl)butan-2-yl)amino)-3-cyclopropyl-1-oxopropan-2-yl)-5-fluoro-2-oxo-1,2-dihydropyridin-3-yl)carbamate **8b** (0.40 g, 0.76 mmol) in acetone (25 mL) was treated with IBX (0.64 g, 2.29 mmol) and then heated at reflux for 5 h. After cooling to ambient temperature, the suspended solid was filtered and washed with acetone. The filtrate was concentrated under reduced pressure and redissolved in CH_2_Cl_2_ (5 mL). A small amount of undissolved solid was filtered, and the filtrate again concentrated under reduced pressure. Purification by flash column chromatography, elution with 6% MeOH/CH_2_Cl_2_, followed by lyophilization from acetonitrile/water (1:1) afforded the title compound as a white solid (0.27 g, 69%). d.r. ∼ 2:1 (*S*,*S*,*S*): (*R*,*S*,*S*), SFC purity (sum of two diastereomers): 94.6%, HPLC purity: 98.1% ^1^H NMR (400 MHz, DMSO-*d*_6_, 120 °C): δ 8.55 (2*d* (1:2 ratio diastereomer pair), *J* = 6.3 Hz, 1H), 7.79 (dd, *J* = 9.4, 3.1 Hz, 1H), 7.75 (br s, 1H), 7.42–7.36 (m, 3H), 7.16 (br s, 1H), 5.61 (2 x ddd (1:2 ratio diastereomer pair), *J* = 9.1, 6.0, 1.2 Hz, 1H), 5.16–5.10 and 5.06–4.98 (2*m*, 1H), 3.22–3.10 (m, 2H), 2.42–2.10 (m, 2H), 2.05–1.60 (m, 5H), 1.51 (2*s* (1:2 ratio diastereomer pair), 9H), 0.67–0.56 (m, 1H), 0.41–0.35 (m, 2H), 0.17–0.11 (m, 1H), 0.06–0.00 (m, 1H)·^13^C NMR (101 MHz, DMSO-*d*_6_, ambient temperature): δ 196.66, 178.68, 169.05, 162.68, 154.95, 152.00, 147.22, 144.98, 128.55, 114.12, 111.42, 94.02, 80.76, 58.40 (=N–CH-CO), 52.40, 37.81, 35.32, 31.12, 30.87, 27.81, 27.10, 7.47, 4.62, 4.44, 3.50. ESI-HRMS: calcd for C_24_H_33_FN_5_O_7_ [M + H]^+^, *m*/*z* = 522.2364 Da; found, *m*/*z* = 522.2360 Da.

##### *tert*-Butyl (1-(1-(((*S*)-4-Amino-3,4-dioxo-1-((*S*)-2-oxopyrrolidin-3-yl)butan-2-yl)amino)-3-cyclopropyl-1-oxopropan-2-yl)-6-oxo-1,6-dihydropyrimidin-5-yl)carbamate (**9c**)

Following the procedure described for the preparation of compound **9b** with a slight modification of conducting the reaction with compound **8c** (0.13 g, 0.26 mmol) and IBX (0.22 g, 0.77 mmol) afforded the α-ketoamide **9c** (80 mg, 58%) as a white solid. d.r. ∼ 1:1 (*S*,*S*,*S*): (*R*,*S*,*S*), SFC purity (sum of two diastereomers) > 95%, ^1^H NMR (400 MHz, DMSO-*d*_6_, 120 °C): δ 8.67 (dd, *J* = 14.8, 6.7, 1H), 8.29 (br s, 1H), 8.14 (d, *J* = 1.5 Hz, 1H), 7.49 (br s, 1H), 7.39 (br s, 2H), 7.20 (br m, 1H), 5.52 (2 x dd, *J* = 9.1, 6.2 Hz, 1H), 5.17–5.12 and 5.07–5.00 (m, 1H), 3.25–3.11 (m, 2H), 2.43–2.15 (m, 2H), 2.10–1.90 (m, 3H), 1.81–1.64 (m, 2H), 1.50 (2*s*, 9H), 0.70–0.67 (m, 1H), 0.44–0.35 (m, 2H), 0.17–0.11 (m, 1H), 0.06–0.00 (m, 1H). ^13^C NMR (101 MHz, DMSO-*d*_6_, ambient temperature): δ 196.66, 177.89, 174.05, 168.78, 162.67, 156.70, 152.40, 144.53, 136.51, 125.74, 94.07, 80.24, 53.01 (=N–CH-CO), 52.39 (=N–CH-CO), 35.32, 31.19, 27.89, 27.53, 27.07, 7.51, 4.75, 4.50, 3.31, 3.14. ESI-HRMS: calcd for C_23_H_33_N_6_O_7_ [M + H]^+^, *m*/*z* = 505.2411 Da; found, *m*/*z* = 505.2408 Da.

##### *tert*-Butyl (4-(1-(((*S*)-4-Amino-3,4-dioxo-1-((*S*)-2-oxopyrrolidin-3-yl)butan-2-yl)amino)-3-cyclopropyl-1-oxopropan-2-yl)-3-oxo-3,4-dihydropyrazin-2-yl)carbamate (**9d**)

Following the procedure described for the preparation of compound **9b** with a slight modification of conducting the reaction with compound **8d** (0.34 g, 0.64 mmol) and IBX (0.54 g, 1.91 mmol) afforded the α-ketoamide **9d** (210 mg, 59%) as a white solid. d.r. ∼ 2:1 (*S*,*S*,*S*): (*R*,*S*,*S*), SFC purity (sum of two diastereomers): 94.6%, ^1^H NMR (400 MHz, DMSO-*d*_6_, 100 °C): δ 8.68 (2*d* (2:1 ratio diastereomer pair), *J* = 6.8 Hz, 1H), 8.34 (br s, 1H), 7.50 (br s, 2H), 7.33–7.23 (m, 2H), 7.02 (2*d*, *J* = 4.8 Hz, 1H), 5.43 (2 x dd, *J* = 9.0, 6.3 Hz, 1H), 5.15–5.10 and 5.05–4.98 (2*m*, 1H), 3.23–3.10 (m, 2H), 2.41–2.12 (m, 2H), 2.01–1.62 (m, 5H), 1.41 (2*s*, 9H), 0.66–0.55 (m, 1H), 0.41–0.34 (m, 2H), 0.17–0.11 (m, 1H), 0.06–0.00 (m, 1H). ^13^C NMR (101 MHz, DMSO-*d*_6_, ambient temperature): δ 196.65, 177.89, 168.66, 162.90, 151.06, 149.94, 145.42, 122.15, 119.91, 80.18, 57.98, 52.43, 37.81, 35.05, 34.83, 31.14, 27.81, 27.16, 7.44, 4.68, 4.49, 3.51. ESI-HRMS: calcd for C_23_H_33_N_6_O_7_ [M + H]^+^, *m*/*z* = 505.2411 Da; found, *m*/*z* = 505.2406 Da.

##### 1-((2*S*)-1-(((2*S*)-3-Acetoxy-4-(methylamino)-4-oxo-1-((*S*)-2-oxopyrrolidin-3-yl) Butan-2-yl)amino)-3-cyclopropyl-1-oxopropan-2-yl)-2-oxo-1,2-dihydropyridin-3-aminium Chloride (**10a**)

To the stirred solution of compound **5a** (710 mg, 1.268 mmol) in CH_2_Cl_2_, 4 M HCl in dioxin (3.77 mL, 12.68 mmol) was added at ice-cold temperature. The reaction mixture was stirred for 6 h. After confirming the completion of the reaction based on LCMS, it was concentrated to dryness and coevaporated with methanol. This resulting solid was used for further reaction without purification (620 mg, 98%). Part of the compound was treated with a trimethylamine solution and purified again to get free amine. ^1^H NMR (700 MHz, CD_3_CN, 298 K): δ 7.87–7.77 (m, 1H), 7.54–7.43 (m, 1H), 7.19–6.80 (m, 2H), 6.82–6.56 (m, 1H), 6.26–6.17 (m, 1H), 6.05–5.83 (m, 1H), 5.43–5.21 (m, 1H), 5.14–4.80 (m, 1H), 4.46–4.20 (m, 1H), 3.20–3.07 (m, 2H), 2.62–2.40 (4Xs, 3H), 2.35–2.08 (m, 3H), 2.09–1.90 (m, 4H), 1.85–1.69 (m, 2H), 1.68–1.45 (m, 2H), 1.39–1.15 (m, 2H), 0.51–0.48 (m, 1H), 0.32–0.26 (m, 2H), ESI-MS Da (*m*/*z*): 462 Da [M + H]^+^.

##### (3*S*)-3-((*S*)-2-(3-Amino-2-oxopyridin-1(2*H*)-yl)-3-cyclopropylpropanamido)-1-(cyclopropylamino)-1-oxo-4-((S)-2-oxopyrrolidin-3-yl) Butan-2-yl Acetate Hydrochloride (**10b**)

Following the procedure described for the preparation of compound **10a** with a slight modification of conducting the reaction with Boc-protected amine **5b** (149 mg, 0.2538 mmol) at rt afforded compound **10b** (115 mg, 84%) as a white solid. ^1^H NMR (500 MHz, CD_3_CN, 298 K): δ 7.29–6.81 (m, 3H), 6.75–6.47 (m, 1H), 6.17–5.89 (m, 2H), 5.46–5.14 (m, 1H), 4.98–4.74 (m, 1H), 4.51–4.21 (m, 1H), 4.34–4.22 (m, 1H), 3.29–3.01 (m, 2H), 2.65–2.42 (m, 1H), 2.36–2.04 (m, 4H), 1.99–1.77 (m, 3H), 1.75–1.31 (m, 2H), 1.77–1.30 (m, 3H), 0.70–0.26 (m, 5H), 0.16 to −0.10 (m, 2H). ESI-MS (*m*/*z*): 488 Da [M + H]^+^.

##### (3*S*)-3-((*S*)-3-Cyclopropyl-2-(3-isobutyramido-2-oxopyridin-1(2*H*)-yl) propanamido)-1-(methylamino)-1-oxo-4-((*S*)-2-oxopyrrolidin-3-yl)butan-2-yl Acetate (**11a**)

To a solution of isobutyric acid (21.24 mg, 0.241 mmol) in DMF, HATU (91.7 mg, 0.241 mmol) was added at 0 °C. The reaction mixture was stirred at 0 °C under a nitrogen atmosphere, and after few minutes, a prestirred solution of amine salt (60 mg, 0.1207 mmol) and triethylamine (73.14 mg, 0.723 mmol) in DMF at 0 °C was added. The reaction was continued for 10 h at 0 °C. Progress of the reaction was monitored based on TLC and LCMS. The reaction mixture was concentrated, diluted with CH_2_Cl_2_ (5 mL), and washed with water. The resulting organic layer was washed with brine solution and dried over Na_2_SO_4_. The resulting organic was evaporated to dryness and purified on a flash column over a C18 column (25–45% CH_3_CN/H_2_O). The product was isolated with a 75% yield (48 mg). ^1^H NMR (500 MHz, CD_3_CN, 298 K): δ 8.50–8.24 (m, 2H), 7.35–6.96 (m, 2H), 6.84–6.58 (m, 1H), 6.34–6.24 (m, 1H), 6.14–5.91 (m, 1H), 5.47–5.32 (m, 1H), 5.25–4.87 (m, 1H), 4.53–4.28 (m, 1H), 3.26–3.02 (m, 2H), 2.72–2.60 (m, 3H), 2.56–2.47 (m, 1H), 2.26–2.04 (m, 4H), 1.98–1.79 (m, 3H), 1.77–1.20 (m, 3H), 1.20–1.08 (m, 6H), 0.65–0.50 (m, 1H), 0.43–0.30 (m, 2H), 0.18 to −0.03 (m, 2H). ESI-MS (*m*/*z*): 432 Da [M + H]^+^.

##### (3*S*)-3-((*S*)-2-(3-(Cyclohexanecarboxamido)-2-oxopyridin-1(2*H*)-yl)-3-cyclopropylpropanamido)-1-(methylamino)-1-oxo-4-((*S*)-2-oxopyrrolidin-3-yl)butan-2-yl Acetate (**11b**)

Following the procedure described for the preparation of compound **11a** with a slight modification of conducting the reaction with compound **10a** (50 mg, 0.100 mmol) and cyclohexane carboxylic acid (23.4 mg, 0.20 mmol) afforded compound **11b** (41 mg, 72%) as a white solid. ^1^H NMR (500 MHz, CDCl_3_, 298 K): δ 8.46–8.36 (m, 1H), 8.34–7.88 (m, 1H), 7.68–7.39 (m, 1H), 7.25–7.13 (m, 1H), 6.38–6.28 (m, 1H), 6.26–6.07 (m, 1H), 5.98–5.71 (m, 1H), 5.52–5.12 (m, 2H), 4.46–4.30 (m, 1H), 3.80–3.68 (m, 2H), 3.41–3.14 (m, 3H), 2.98–2.62 (4Xs, 3H), 2.50–2.25 (m, 2H), 2.20–2.11 (m, 3H), 2.08–1.60 (m, 4H), 1.56–1.39 (m, 7H), 1.39–1.20 (m, 2H), 0.65–0.53 (m, 1H), 0.48–0.37 (m, 2H), 0.19–0.10 (m, 1H), 0.09–0.01 (m, 1H). ESI-MS (*m*/*z*): 572 Da [M + H]^+^.

##### (3*S*)-3-((*S*)-3-Cyclopropyl-2-(2-oxo-3-(2-phenylacetamido)pyridin-1(2*H*)-yl)propanamido)-1-(methylamino)-1-oxo-4-((*S*)-2-oxopyrrolidin-3-yl)butan-2-yl Acetate (**11c**)

Following the procedure described for the preparation of compound **11a** with a slight modification of conducting the reaction with compound **10a** (40 mg, 0.0805 mmol) and 2-phenylacetic acid (22 mg, 0.161 mmol) afforded compound **11c** (33 mg, 71%) as a white solid. ^1^H NMR (500 MHz, CDCl_3_, 298 K): δ 8.69–8.33 (m, 2H), 7.70 (d, *J* = 8.6 Hz, 1H), 7.44–7.17 (m, 5H), 6.94–6.38 (m, 1H), 6.35–6.24 (m, 1H), 6.14–5.79 (m, 1H), 5.48–5.07 (m, 2H), 4.44–4.27 (m, 1H), 3.80–3.69 (m, 2H), 3.40–3.13 (m, 3H), 2.83–2.47 (4xs, 3H), 2.46–2.22 (m, 1H), 2.19–2.04 (m, 2H), 2.02–1.72 (m, 4H), 1.65–1.39 (m, 3H), 0.64–0.49 (m, 1H), 0.46–0.35 (m, 2H), 0.11 (td, *J* = 9.3, 4.5 Hz, 1H), 0.07 to −0.02 (m, 1H). ESI-MS (*m*/*z*): 580 Da [M + H]^+^.

##### (3*S*)-3-((*S*)-3-Cyclopropyl-2-(2-oxo-3-(3-phenylpropanamido)pyridin-1(2*H*)-yl)propanamido)-1-(methylamino)-1-oxo-4-((*S*)-2-oxopyrrolidin-3-yl)butan-2-yl Acetate (**11d**)

Following the procedure described for the preparation of compound **11a** with a slight modification of conducting the reaction with compound **10a** (35 mg, 0.0704 mmol) and 3-phenylpropanoic acid (21.2 mg, 0.141 mmol) afforded compound **11d** (38.8 mg, 93%) as a white solid. ^1^H NMR (500 MHz, CDCl_3_, 298 K): δ 8.69–8.01 (m, 3H), 8.10–7.51 (m, 1H), 7.39–7.15 (m, 5H), 6.79–6.44 (m, 1H), 6.44–5.99 (m, 2H), 5.60–5.11 (m, 2H), 4.52–4.30 (m, 1H), 3.79–3.66 (m, 1H), 3.43–3.15 (m, 3H), 3.09–3.00 (m, 2H), 2.82–2.54 (m, 4H), 2.49–2.12 (m, 2H), 2.10–1.67 (m, 6H), 1.63–1.49 (m, 1H), 0.59–0.51 (m, 1H), 0.46–0.30 (m, 2H), 0.16–0.07 (m, 1H), 0.05 to −0.04 (m, 1H). ESI-MS (*m*/*z*): 594 Da [M + H]^+^.

##### (3*S*)-3-((*S*)-2-(3-(((Benzyloxy)carbonyl)amino)-2-oxopyridin-1(2*H*)-yl)-3-cyclopropylpropanamido)-1-(methylamino)-1-oxo-4-((*S*)-2-oxopyrrolidin-3-yl)butan-2-yl acetate(**11e**)

To the stirred solution of **10a** (25 mg, 0.051 mmol) in aq. THF (1:3, 2 mL), NaHCO_3_ (13 mg, 0.152 mmol) was added at 0 °C. The reaction mixture was stirred at 0 °C for few minutes, and a predissolved solution of benzyl chloroformate (26 mg, 0.152 mmol, in 0.5 mL THF) was added slowly. The reaction was continued for 30 min at a 0 °C temperature and was slowly raised to rt. Progress of the reaction was monitored based on TLC and LCMS. After 16 h, the reaction mixture was concentrated, diluted with CH_2_Cl_2_ (5 mL), and washed with water. The resulting organic layer was washed with a brine solution and dried over Na_2_SO_4_. The resulting organic was evaporated to dryness and purified on a flash column at 3–8% CH_3_OH in CH_2_Cl_2_ which gave the product with an 83% yield (25.3 mg). ^1^H NMR (500 MHz, CDCl_3_, 298 K): δ 8.06 (m, 1H), 7.89–7.80 (m, 1H), 7.71–7.55 (m, 1H), 7.44–7.32 (m, 4H), 7.22–7.11 (m, 1H), 6.53–6.18 (m, 2H), 5.84–5.55 (m, 1H), 5.51–5.10 (m, 3H), 4.47–4.28 (m, 1H), 3.41–3.15 (m, 3H), 2.86–2.54 (m, 3H), 2.50–2.26 (m, 2H), 2.24–2.07 (m, 3H), 2.04–1.80 (m, 4H), 1.77–1.66 (m, 2H), 0.64–0.53 (m, 1H), 0.49–0.38 (m, 2H), 0.20–0.01 (m, 2H). ESI-MS (*m*/*z*): 596 Da [M + H]^+^.

##### (3*S*)-3-((*S*)-3-Cyclopropyl-2-(2-oxo-3-(3-(pyridin-3-yl)propanamido)pyridin-1(2*H*)-yl)propanamido)-1-(methylamino)-1-oxo-4-((*S*)-2-oxopyrrolidin-3-yl)butan-2-yl Acetate (**11f**)

Following the procedure described for the preparation of compound **11a** with a slight modification of conducting the reaction with compound **10a** (28 mg, 0.056 mmol) and 3-(pyridin-3-yl)propanoic acid (17 mg, 0.1126 mmol) afforded compound **11f** (31.5 mg, 95%) as a white solid. ^1^H NMR (500 MHz, CD_3_OD, 298 K): δ 8.85–8.63 (m, 1H), 8.52–7.97 (m, 5H), 7.80 (t, *J* = 7.7 Hz, 1H), 7.44 (m, 3H), 6.41–6.30 (m, 1H), 5.61–5.46 (m, 1H), 5.28–4.93 (m, 1H), 4.52–4.34 (m, 1H), 3.80–3.67 (m, 1H), 3.17–2.77 (m, 6H), 2.75–2.48 (m, 3H), 2.43–1.60 (m, 7H), 1.52–1.18 (m, 2H), 0.65–0.57 (m, 1H), 0.41 (dd, *J* = 10.6, 4.2 Hz, 2H), 0.21–0.12 (m, 1H), 0.05 (dd, *J* = 15.0, 8.3 Hz, 1H). ESI-MS (*m*/*z*): 595 Da [M + H]^+^.

##### (3*S*)-3-((*S*)-3-Cyclopropyl-2-(2-oxo-3-(3-(thiophen-2-yl)propanamido)pyridin-1(2*H*)-yl)propanamido)-1-(methylamino)-1-oxo-4-((*S*)-2-oxopyrrolidin-3-yl)butan-2-yl Acetate (**11g**)

Following the procedure described for the preparation of compound **11a** with a slight modification of conducting the reaction with compound **10a** (30 mg, 0.0603 mmol) and 3-(thiophen-2-yl)propanoic acid (18.2 mg, 0.121 mmol) afforded compound **11g** (30 mg, 83%) as a white solid. ^1^H NMR (500 MHz, CD_3_CN, 298 K): δ 8.55–8.37 (m, 1H), 8.39–8.24 (m, 1H), 7.46–7.11(m, 3H), 7.05–6.85 (m, 2H), 6.81–6.56 (br. d, 1H), 6.35–6.22 (m, 1H), 6.13–5.95 (m, 1H), 5.47–5.24 (m, 1H), 5.20–4.86 (m, 1H), 4.56–4.26 (m, 1H), 3.26–2.99 (m, 4H), 2.79 (d, *J* = 5.2 Hz, 2H), 2.68–2.45 (m, 3H), 2.36–2.21 (m, 2H), 2.15–2.04 (m, 4H), 2.04–1.21 (m, 6H), 0.58 (m, 1H), 0.43–0.31 (m, 2H), 0.154–0.062 (m, 1H), 0.038 to −0.01 (m,1H). ESI-MS (*m*/*z*): 600 Da [M + H]^+^.

##### (3*S*)-3-((*S*)-3-Cyclopropyl-2-(3-(nicotinamido)-2-oxopyridin-1(2*H*)-yl)propanamido)-1-(methylamino)-1-oxo-4-((*S*)-2-oxopyrrolidin-3-yl)butan-2-yl Acetate (**11h**)

Following the procedure described for the preparation of compound **11a** with a slight modification of conducting the reaction with compound **10a** (50 mg, 0.100 mmol) and nicotinic acid (25 mg, 0.201 mmol) afforded compound **11h** (36.8 mg, 65%) as a white solid. ^1^H NMR (500 MHz, CD_3_CN, 298 K): δ 9.35–9.04 (m, 2H), 8.84–8.72 (m, 1H), 8.51–8.39 (m, 1H), 8.34–8.16 (m, 1H), 7.55–7.33 (m, 2H), 7.28–6.99 (m, 1H), 6.84–6.59 (m, 1H), 6.46–6.27 (m, 1H), 6.18–5.94 (m, 1H), 5.53–5.31 (m, 1H), 5.26–4.91 (m, 1H), 4.60–4.32 (m, 1H), 3.32–3.01 (m, 2H), 2.69–2.43 (m, 3H), 2.37–2.19 (m, 1H), 2.15–1.96 (m, 4H), 1.91–1.80 (m, 2H), 1.78–1.24 (m, 3H), 0.66–0.52 (m, 1H), 0.46–0.28 (m, 2H), 0.19 to −0.04 (m, 2H). ESI-MS (*m*/*z*): 567 Da [M + H]^+^.

##### (*S*)-3-((*S*)-3-Cyclopropyl-2-(3-isobutyramido-2-oxopyridin-1(2*H*)-yl)propanamido)-*N*-methyl-2-oxo-4-((*S*)-2-oxopyrrolidin-3-yl)butanamide (**12a**)

Compound **12a** was prepared in two steps in a 64% yield by deacylation of acetoxy amide **11a** (53 mg, 0.100 mmol) followed by oxidation with DMP (52 mg, 0.122 mmol) utilizing the same procedure as described for compound **6a**. d.r. ∼ 1:1 (*S*,*S*,*S*): (*R*,*S*,*S*), HPLC purity: 99.2%, ^1^H NMR (700 MHz, CD_3_CN, 298 K): δ 8.63–8.38 (m, 2H), 8.36–8.25 (m, 1H), 7.39–7.20 (m, 2H), 6.32–6.24 (m, 1H), 6.21–6.08 (m, 1H), 5.64–5.46 (dd, *J* = 9.1, 6.3 Hz, 1H), 5.27–5.05 (m, 1H), 3.28–3.07 (m, 2H), 2.81–2.60 (m, 4H), 2.54–2.21 (m, 2H), 2.06–1.67 (m, 6H), 1.20–1.10 (m, 6H), 0.64–0.54 (m, 1H), 0.41–0.33 (m, 2H), 0.14–0.08 (m, 1H), 0.04 to −0.03 (m, 1H). ^13^C NMR (176 MHz, CD_3_CN, 298 K): δ 195.47, 179.26, 175.96, 169.26, 160.54, 157.41, 129.06, 128.54, 121.12, 105.70, 59.15, 53.64, 40.07, 38.55, 35.99, 35.13, 31.04, 28.33, 24.93, 18.83, 7.39, 3.98, 3.48. ESI-HRMS: calcd for C_24_H_34_N_5_O_6_ [M + H]^+^: *m*/*z* = 488.2509 Da; found, *m*/*z* = 488.2505 Da.

##### *N*-(1-((*S*)-3-Cyclopropyl-1-(((*S*)-4-(methylamino)-3,4-dioxo-1-((*S*)-2-oxopyrrolidin-3-yl)butan-2-yl)amino)-1-oxopropan-2-yl)-2-oxo-1,2-dihydropyridin-3-yl)cyclohexanecarboxamide (**12b**)

Compound **12b** was prepared in two steps in a 59% yield by deacylation of acetoxy amide **11b** (51 mg, 0.089 mmol) followed by oxidation with DMP (52 mg, 0.133 mmol) utilizing the same procedure as described for compound **6a**. d.r. ∼ 1:1 (*S*,*S*,*S*): (*R*,*S*,*S*), HPLC purity: 96.9%, ^1^H NMR (500 MHz, CD_3_CN, 298 K): δ 8.63–8.40 (m, 2H), 8.36–8.20 (m, 1H), 7.22 (ddd, *J* = 7.1, 2.6, 1.8 Hz, 2H), 6.25 (t, *J* = 7.2 Hz, 2H), 5.61 (dd, *J* = 8.9, 6.7 Hz, 0.5H), 5.51 (dd, *J* = 9.1, 6.3 Hz, 0.5H), 5.08 (dt, *J* = 7.3, 5.3 Hz, 1H), 3.30–3.15 (m, 2H), 2.73 (dd, *J* = 13.4, 5.1 Hz, 3H), 2.56–2.46 (m, 1H), 2.45–2.29 (m, 2H), 2.24–2.17 (m, 2H), 2.01–1.61 (m, 9H), 1.42 (ddd, *J* = 12.1, 7.8, 3.5 Hz, 2H), 1.37–1.17 (m, 3H), 0.64–0.50 (m, 1H), 0.42–0.30 (m, 2H), 0.15–0.05 (m, 1H), 0.04 to −0.05 (m, 1H). ^13^C NMR (176 MHz, CD_3_CN, 298 K): δ 196.46, 180.38, 176.04, 170.22, 161.51, 158.36, 129.90, 129.16, 122.08, 106.73, 60.04 (=N–CH-CO), 59.16 (=N–CH-CO), 54.54, 46.57, 41.04, 39.78, 36.42, 32.14, 30.35, 29.32, 26.44, 25.88, 8.31, 4.93, 4.80, 4.44. ESI-HRMS: calcd for C_27_H_38_N_5_O_6_ [M + H]^+^, *m*/*z* = 528.2822 Da; found, *m*/*z* = 528.2824 Da.

##### (*S*)-3-((*S*)-3-Cyclopropyl-2-(2-oxo-3-(2-phenylacetamido)pyridin-1(2*H*)-yl)propanamido)-*N*-methyl-2-oxo-4-((S)-2-oxopyrrolidin-3-yl)butanamide (**12c**)

Compound **12c** was prepared in two steps in a 62% yield by deacylation of acetoxy amide **11c** (33 mg, 0.0569 mmol) followed by oxidation with DMP (24 mg, 0.0566 mmol) utilizing the same procedure as described for compound **6a**. d.r. ∼ 1:1 (*S*,*S*,*S*): (*R*,*S*,*S*), HPLC purity: 98.2%, ^1^H NMR (500 MHz, CD_3_CN, 298 K): δ 8.61–8.41 (m, 0.5H; ddd, *J* = 7.7, 6.1, 1.8 Hz, 1.5H), 8.25 (m, 1H), 7.44–7.18 (m, 6H), 6.28–6.09 (m, 2H), 5.55 (dd, *J* = 8.9, 6.7 Hz, 0.5H), 5.45 (dd, *J* = 9.1, 6.3 Hz, 0.5H), 5.11–5.01 (m, 1H), 3.74 (s, 2H), 3.29–3.13 (m, 2H), 2.72 (dd, *J* = 13.8, 5.1 Hz, 3H), 2.52–2.08 (m, 4H), 1.90–1.68 (m, 4H), 0.64–0.48 (m, 1H), 0.42–0.28 (m, 2H), 0.13–0.04 (m, 1H), −0.01 (d, *J* = 5.0 Hz, 1H). ^13^C NMR (176 MHz, CD_3_CN, 298 K): δ 195.47, 179.37, 169.83, 169.20, 160.53, 157.24, 135.34, 129.51, 128.78, 127.15, 121.21, 105.60, 59.04 (=N–CH-CO), 58.13 (=N–CH-CO), 53.62, 43.95, 40.07, 38.84, 35.49, 31.02, 28.43, 24.92, 7.34, 3.96, 3.83, 3.48. ESI-HRMS: calcd for C_28_H_34_N_5_O_6_ [M + H]^+^, *m*/*z* = 536.2509 Da; found, *m*/*z* = 536.2509 Da.

##### (*S*)-3-((*S*)-3-Cyclopropyl-2-(2-oxo-3-(3-phenylpropanamido)pyridin-1(2*H*)-yl)propanamido)-*N*-methyl-2-oxo-4-((*S*)-2-oxopyrrolidin-3-yl)butanamide (**12d**)

Compound **12d** was prepared in two steps in a 57% yield by deacylation of acetoxy amide **11d** (38 mg, 0.0689 mmol) followed by oxidation with DMP (37 mg, 0.087 mmol) utilizing the same procedure as described for compound **6a**. d.r. ∼ 1:1 (*S*,*S*,*S*): (*R*,*S*,*S*), HPLC purity: 97.6%, ^1^H NMR (500 MHz, CD_3_CN, 298 K): δ 8.60–8.40 (d, *J* = 5 Hz, 0.5H; m, 1.5H), 8.29–8.24 (m, 1H), 7.33–7.16 (m, 6H), 6.24 (t, *J* = 7 Hz, 1H), 6.19–6.12 (br, 1H), 5.60–5.56, 5.50–5.47 (br, 1H), 5.1–5.06 (m, 1H), 3.26–3.19 (m, 2H), 2.98–2.94 (m, 2H), 2.76–2.69 (m, 5H), 2.53–2.49 (m,1H), 2.45–2.05 (m, 3H), 1.95–1.77 (m, 6H), 0.61–0.51 (m,1H), 0.39–0.30 (m, 2H), 0.12–0.06 (m,1H), −0.02 to −0.04 (m, 1H). ^13^C NMR (126 MHz, CD_3_CN, 298 K): δ 196.40, 180.54, 172.26, 170.38, 161.66, 158.32, 142.21, 129.81, 129.55, 127.27, 122.38, 106.66, 60.15 (=N–CH-CO), 59.20 (=N–CH-CO), 54.63, 41.01, 39.90, 39.58, 36.56, 32.23, 31.86, 29.58, 25.92, 8.46, 5.02, 4.84, 4.51. ESI-HRMS: calcd for C_29_H_36_N_5_O_6_ [M + H]^+^, *m*/*z* = 550.2665 Da; found, *m*/*z* = 550.2662 Da.

##### Benzyl (1-((*S*)-3-Cyclopropyl-1-(((*S*)-4-(methylamino)-3,4-dioxo-1-((*S*)-2-oxopyrrolidin-3-yl)butan-2-yl)amino)-1-oxopropan-2-yl)-2-oxo-1,2-dihydropyridin-3-yl)carbamate (**12e**)

Compound **12e** was prepared in two steps in a 61% yield by deacylation of acetoxy amide **11e** (21 mg, 0.0352 mmol) followed by oxidation with DMP (27.6 mg, 0.065 mmol) utilizing the same procedure as described for compound **6a**. d.r. ∼ 1:1 (*S*,*S*,*S*): (*R*,*S*,*S*), HPLC purity: 98.9%, ^1^H NMR (500 MHz, CD_3_CN, 298 K): δ 8.61–8.41 (m, 1H), 7.94 (t, *J* = 6.5 Hz, 1H), 7.86 (d, *J* = 8.6 Hz, 1H), 7.48–7.18 (m, 7H), 6.27 (td, *J* = 7.3, 1.3 Hz, 1H), 6.21–6.09 (br, 1H), 5.64–5.46 (m, 1H), 5.25–5.15 (s, 2H), 5.08 (tt, *J* = 10.5, 5.2 Hz, 1H), 3.30–3.14 (m, 2H), 2.75 (dd, *J* = 9.3, 6.7 Hz, 3H), 2.53–2.20 (m, 2H), 2.19–1.89 (m, 2H), 1.88–1.70 (m, 4H), 0.65–0.53 (m, 1H), 0.42–0.32 (m, 2H), 0.14–0.06 (m, 1H), 0.04 to −0.04 (m, 1H). ^13^C NMR (176 MHz, CD_3_CN, 298 K): δ 195.48, 179.38, 169.23, 160.54, 157.16, 153.28, 136.64, 128.78, 128.21, 127.94, 119.62, 105.61, 66.62, 58.20 (=N–CH-CO), 53.67, 40.06, 38.82, 38.54, 35.48, 31.16, 30.98, 28.42, 24.92, 7.34, 3.96, 3.45. ESI-HRMS: calcd for C_28_H_34_N_5_O_7_ [M + H]^+^, *m*/*z* = 552.2458 Da; found, *m*/*z* = 552.2457 Da.

##### (*S*)-3-((*S*)-3-Cyclopropyl-2-(2-oxo-3-(3-(pyridin-3-yl)propanamido)pyridin-1(2*H*)-yl)propanamido)-*N*-methyl-2-oxo-4-((*S*)-2-oxopyrrolidin-3-yl)butanamide (**12f**)

Compound **12f** was prepared in two steps in a 53% yield by deacylation of acetoxy amide **11f** (35 mg, 0.059 mmol) followed by oxidation with DMP (43 mg, 0.101 mmol) utilizing the same procedure as described for compound **6a**. d.r. ∼ 1:1 (*S*,*S*,*S*): (*R*,*S*,*S*), LCMS purity: >95%, ^1^H NMR (700 MHz, CD_3_CN, 298 K): δ 8.67–8.43 (m, 3H), 8.39 (d, *J* = 4.7 Hz, 1H), 8.26 (ddd, *J* = 12.3, 7.4, 1.6 Hz, 1H), 7.63 (ddd, *J* = 7.3, 4.3, 2.0 Hz, 1H), 7.33–7.22 (m, 2H), 7.21–7.16 (m, 1H), 6.35–6.21 (m, 2H), 5.61–5.46 (m, 1H), 5.27–5.05 (m, 1H), 3.27–3.12 (m, 2H), 3.04–2.90 (m, 2H), 2.83–2.69 (m, 4H), 2.54–2.45 (m, 1H), 2.41–2.19 (m, 2H), 2.05–1.67 (m, 6H), 0.61–0.53 (m, 1H), 0.39–0.30 (m, 2H), 0.11–0.06 (m, 1H), −0.02 (m, 1H). ^13^C NMR (176 MHz, CD_3_CN, 298 K): δ 195.46, 179.45, 170.87, 169.26, 160.54, 157.29, 149.86, 147.48, 136.54, 135.88, 128.89, 123.35, 121.12, 105.66, 59.01 (=N–CH-CO), 58.26 (=N–CH-CO), 53.71, 40.10, 38.95, 38.64, 35.36, 31.15, 28.52, 24.93, 7.34, 3.95, 3.45. ESI-HRMS: calcd for C_28_H_35_N_6_O_6_ [M + H]^+^, *m*/*z* = 551.2618 Da; found, *m*/*z* = 551.2617 Da.

##### (*S*)-3-((*S*)-3-Cyclopropyl-2-(2-oxo-3-(3-(thiophen-2-yl)propanamido)pyridin-1(2*H*)-yl)propanamido)-*N*-methyl-2-oxo-4-((*S*)-2-oxopyrrolidin-3-yl)butanamide (**12g**)

Compound **12g** was prepared in two steps in a 68% yield by deacylation of acetoxy amide **11g** (21 mg, 0.035 mmol) followed by oxidation with DMP (29 mg, 0.0687 mmol) utilizing the same procedure as described for compound **6a**. d.r. ∼ 1:1 (*S*,*S*,*S*): (*R*,*S*,*S*), HPLC purity: 99.29%, ^1^H NMR (500 MHz, CD_3_CN, 298 K): δ 8.77–8.48 (m, 2H), 8.34–8.22 (m, 1H), 7.39–7.12 (m, 3H), 6.96–6.80 (m, 2H), 6.28 (ddd, *J* = 14.5, 13.9, 7.7 Hz, 2H), 5.65–5.47 (m, 1H), 5.27–5.02 (m, 1H), 3.34–3.08 (m, 4H), 2.90–2.65 (m, 5H), 2.57–2.05 (m, 3H), 1.97–1.64 (m, 5H), 0.61–0.50 (m, 1H), 0.45–0.26 (m, 2H), 0.17–0.04 (m, 1H), 0.03 to −0.08 (m, 1H). ^13^C NMR (176 MHz, CD_3_CN, 298 K): δ 195.94, 179.35, 170.73, 160.52, 157.19, 143.71, 128.79, 126.96, 124.83, 123.53, 121.41, 105.56, 59.04 (=N-CH-CO), 58.23 (=N–CH–CO), 53.66, 52.94, 40.13, 38.52, 37.59, 35.42, 31.17, 30.23, 28.44, 24.96, 7.35, 3.97, 3.48. ESI-HRMS: calcd for C_27_H_34_N_5_O_6_S [M + H]^+^, *m*/*z* = 556.2229 Da; found, *m*/*z* = 556.2229 Da.

##### *N*-(1-((*S*)-3-Cyclopropyl-1-(((*S*)-4-(methylamino)-3,4-dioxo-1-((*S*)-2-oxopyrrolidin-3-yl)butan-2-yl)amino)-1-oxopropan-2-yl)-2-oxo-1,2-dihydropyridin-3-yl)nicotinamide (**12h**)

Compound **12h** was prepared in two steps in a 39% yield by deacylation of acetoxy amide **11h** (33 mg, 0.070 mmol) followed by oxidation with DMP (26 mg, 0.061 mmol) utilizing the same procedure as described for compound **6a**. d.r. ∼ 1:1 (*S*,*S*,*S*): (*R*,*S*,*S*), HPLC purity: 99.5%, ^1^H NMR (700 MHz, DMSO-*d*_6_, 298 K): δ 9.60 (s, 1H), 9.11–8.94 (m, 2H), 8.76 (s, 1H), 8.67–8.56 (m, 1H), 8.31–8.20 (m, 2H), 7.75–7.65 (m, 1H), 7.54 (dd, *J* = 32.4, 13.1 Hz, 2H), 6.42–6.32 (m, 1H), 5.72 (dt, *J* = 23.5, 22.0 Hz, 1H), 5.14 (d, *J* = 12.5 Hz, 0.4H), 5.00 (t, *J* = 16.0 Hz, 0.6H), 3.22–3.06 (m, 2H), 2.70–2.53 (m, 3H), 2.39–2.05 (m, 2H), 2.04–1.77 (m, 3H), 1.74–1.50 (m, 3H), 0.54 (t, *J* = 30.5 Hz, 1H), 0.36 (dd, *J* = 32.9, 12.7 Hz, 2H), 0.16 (dd, *J* = 35.0, 14.4 Hz, 1H), 0.03 (dd, *J* = 28.5, 15.7 Hz, 1H)·^13^C NMR (176 MHz, DMSO-*d*_6_, 298 K): δ 196.63, 178.34, 169.88, 164.34, 161.50, 157.83, 152.77, 148.84, 135.69, 130.28, 127.94, 125.22, 105.09, 58.34 (=N–CH-CO), 57.95 (=N–CH-CO), 53.35, 40.91, 38.35, 35.95, 31.44, 27.55, 25.93, 8.06, 5.16, 4.06. ESI-HRMS: calcd for C_26_H_31_N_6_O_6_ [M + H]^+^, *m*/*z* = 523.2305 Da; found, *m*/*z* = 523.2300 Da.

##### (3*S*)-3-((*S*)-3-Cyclopropyl-2-(3-(3-isopropylureido)-2-oxopyridin-1(2*H*)-yl)propanamido)-1-(methylamino)-1-oxo-4-((*S*)-2-oxopyrrolidin-3-yl)butan-2-yl Acetate (**14a**)

To a solution of amine salt **10a** (50 mg, 0.1 mmol) in dry CH_2_Cl_2_, *N*,*N*-diisopropyl amine (70 μL, 0.402 mmol) was added at 0 °C. After stirring for few minutes, isopropyl isocyanate (17 mg, 0.2 mmol) was added at the same temperature and stirred at rt under inert conditions. The reaction was continued for 24 h, and the progress of the reaction was monitored based on TLC and LCMS. After completion of the reaction, it was quenched with 0.1 mL of methanol and evaporated to dryness. The resulting crude was purified based on a flash column over silica gel (5–10% MeOH/CH_2_Cl_2_). The product was isolated with a 45.7% yield (25 mg). ^1^H NMR (700 MHz, CD_3_CN, 298 K): δ 8.11–7.99 (m, 1H), 7.62 (dd, *J* = 16.0, 9.6 Hz, 1H), 7.40–6.93 (m, 2H), 6.86–6.65 (m, 1H), 6.31–6.19 (m, 1H), 6.11–5.90 (m, 1H), 5.74–5.62 (m, 2H), 5.52–4,85 (m, 1H), 4.54–4.27 (m, 1H), 3.87–3.79 (m, 1H), 3.27–3.04 (m, 2H), 2.73–2.49 (m, 3H), 2.43–2.19 (m, 2H), 2.13–1.98 (m, 3H), 1.92–1.69 (m, 3H), 1.69–1.23 (m, 3H), 1.21–0.99 (m, 6H), 0.59–0.52 (m, 1H), 0.36 (ddd, *J* = 22.5, 11.4, 7.3 Hz, 2H), 0.14–0.07 (m, 1H), 0.04 to −0.03 (m, 1H). ESI-MS (*m*/*z*): 547 Da [M + H]^+^.

##### (3*S*)-3-((*S*)-2-(3-(3-(*tert*-Butyl)ureido)-2-oxopyridin-1(2*H*)-yl)-3-cyclopropylpropanamido)-1-(methylamino)-1-oxo-4-((*S*)-2-oxopyrrolidin-3-yl)butan-2-yl Acetate (**14b**)

Following the procedure described for the preparation of compound **14a** with a slight modification of conducting the reaction with compound **10a** (49 mg, 0.0985 mmol) and 2-isocyanato-2-methylpropane (22.3 μL, 0.197 mmol) afforded compound **14b** (21 mg, 38%) as a white solid. ^1^H NMR (500 MHz, CDCl_3_, 298 K): δ 8.28–7.92 (m, 2H), 7.90–7.65 (m, 2H), 7.54–7.10 (m, 1H), 6.92–6.39 (m, 1H), 6.35–6.26 (m, 1H), 6.22–6.05 (m, 1H), 5.81–5.64 (m, 1H), 5.64–5.47 (m, 1H), 5.35–5.19 (m, 1H), 4.60–4.28 (m, 1H), 3.43–3.15 (m, 2H), 2.83–2.60 (m, 3H), 2.49–1.99 (m, 5H), 1.98–1.79 (m, 2H), 1.77–1.50 (m, 3H), 1.43–1.33 (4xs, 9H), 0.53 (dd, *J* = 13.4, 6.1 Hz, 1H), 0.40–0.30 (m, 2H), 0.16 to −0.02 (m, 2H). ESI-MS (*m*/*z*): 561 Da [M + H]^+^.

##### (3*S*)-3-((*S*)-2-(3-(3-Cyclohexylureido)-2-oxopyridin-1(2*H*)-yl)-3-cyclopropylpropanamido)-1-(methylamino)-1-oxo-4-((*S*)-2-oxopyrrolidin-3-yl)butan-2-yl Acetate (**14c**)

Following the procedure described for the preparation of compound **14a** with a slight modification of conducting the reaction with compound **10a** (50 mg, 0.100 mmol) and isocyanato cyclohexane (25.2 mg, 0.201 mmol) afforded compound **14c** (39.8 mg, 68%) as a white solid. ^1^H NMR (500 MHz, CDCl_3_, 298 K): δ 8.25–7.70 (m, 3H), 7.37–7.36 (m, 2H), 7.33–7.28 (m, 1H), 7.24–7.12 (m, 1H), 7.04–6.55 (m, 1H), 6.53–6.25 (m, 1H), 6.20–5.49 (m, 2H), 5.34–5.17 (m, 1H), 4.60–4.29 (m, 1H), 3.69–3.58 (m, 1H), 3.41–3.12 (m, 2H), 2.88–2.60 (m, 3H), 2.49–1.98 (m, 7H), 1.98–1.78 (m, 4H), 1.76–1.49 (m, 3H), 1.43–1.09 (m, 4H), 0.53 (dt, *J* = 19.6, 9.8 Hz, 1H), 0.40–0.29 (m, 2H), 0.16 to −0.03 (m, 2H). ESI-MS (*m*/*z*): 587 Da [M + H]^+^.

##### (3*S*)-3-((*S*)-2-(3-(3-Benzylureido)-2-oxopyridin-1(2*H*)-yl)-3-cyclopropylpropanamido)-1-(methylamino)-1-oxo-4-((*S*)-2-oxopyrrolidin-3-yl) Butan-2-yl Acetate (**14d**)

Following the procedure described for the preparation of compound **14a** with a slight modification of conducting the reaction with compound **10a** (25 mg, 0.05 mmol) and (isocyanatomethyl)benzene (12.5 μL, 0.101 mmol) afforded compound **14d** (18 mg, 61%) as a white solid. ^1^H NMR (500 MHz, CDCl_3_, 298 K): δ 8.33–7.93 (m, 3H), 7.38–7.28 (m, 4H), 7.26–7.21 (m, 1H), 7.17–6.59 (m, 1H), 6.57–6.12 (m, 2H), 5.69–5.47 (m, 1H), 5.34–5.15 (m, 1H), 4.69–4.23 (m, 3H), 3.38–3.05 (m, 3H), 2.72–2.61 (m, 2H), 2.40–1.92 (m, 7H), 1.87–1.46 (m, 5H), 0.59–0.24 (m, 3H), 0.05 to −0.12 (m, 2H). ESI-MS (*m*/*z*): 595 Da [M + H]^+^.

##### (3*S*)-3-((*S*)-3-Cyclopropyl-2-(2-oxo-3-(3-phenylureido) Pyridin-1(2*H*)-yl)propanamido)-1-(methylamino)-1-oxo-4-((*S*)-2-oxopyrrolidin-3-yl)butan-2-yl Acetate (**14e**)

Following the procedure described for the preparation of compound **14a** with a slight modification of conducting the reaction with compound **10a** (32 mg, 0.0693 mmol) and isocyanato benzene (16.5 mg, 0.138 mmol) afforded compound **14e** (27 mg, 67%) as a white solid. ^1^H NMR (500 MHz, CDCl_3_, 298 K): δ 8.78–8.10 (m, 4H), 7.54–7.39 (m, 2H), 7.36–7.10 (m, 5H), 7.09–6.99 (m, 1H), 6.97–6.22 (m, 2H), 5.93–5.12 (m, 2H), 4.72–4.33 (m, 1H), 3.44–3.13 (m, 2H), 2.90–2.66 (m, 3H), 2.53–2.19 (m, 3H), 2.18–1.94 (m, 5H), 1.87–1.53 (m, 2H), 0.56 (dd, *J* = 15.1, 7.6 Hz, 1H), 0.43–0.28 (m, 2H), 0.17 to −0.03 (m, 2H). ESI-MS (*m*/*z*): 581 Da [M + H]^+^.

##### (3*S*)-3-((*S*)-3-Cyclopropyl-2-(2-oxo-3-(3-phenylureido) Pyridin-1(2*H*)-yl)propanamido)-1-(cyclopropylamino)-1-oxo-4-((*S*)-2-oxopyrrolidin-3-yl)butan-2-yl Acetate (**15**)

Following the procedure described for the preparation of compound **14a** with a slight modification of conducting the reaction with compound **10b** (30 mg, 0.05524 mmol) and isocyanato benzene (13.1 mg, 0.110 mmol) afforded compound **15** (27 mg, 68%) as a white solid. ^1^H NMR (500 MHz, CD_3_CN, 298 K): δ 8.35–7.99 (m, 3H), 7.58–7.14 (m, 5H), 7.05–6.80 (m, 2H), 6.38–6.24 (m, 1H), 6.12 (br. d, 1H), 5.75–5.40 (m, 1H), 5.14–4.81 (m, 1H), 4.59–4.27 (m, 1H), 3.32–3.02 (m, 2H), 2.67–2.42 (m, 1H), 2.40–1.97 (m, 6H), 1.92–1.54 (m, 5H), 1.47–1.39 (m, 1H), 0.74–0.25 (m, 4H), 0.17–0.07 (m, 1H), 0.05 to −0.09 (m, 1H). ESI-MS (*m*/*z*): 607 Da [M + H]^+^.

##### (*S*)-3-((*S*)-3-Cyclopropyl-2-(3-(3-isopropylureido)-2-oxopyridin-1(2*H*)-yl) propanamido)-*N*-methyl-2-oxo-4-((*S*)-2-oxopyrrolidin-3-yl) Butanamide (**16a**)

Compound **16a** was prepared in two steps in a 64% yield by deacylation of acetoxy amide **14a** (20 mg, 0.036 mmol) followed by oxidation with DMP (31 mg, 0.073 mmol) utilizing the same procedure as described for compound **6a**. d.r. ∼ 1:1 (*S*,*S*,*S*): (*R*,*S*,*S*), HPLC purity: 98.9%, ^1^H NMR (700 MHz, CD_3_CN, 298 K): δ 8.50–8.27 (m, 1H), 8.01 (t, *J* = 11.9 Hz, 1H), 7.64 (d, *J* = 11.7 Hz, 1H), 7.27 (br, 1H), 7.09 (t, *J* = 9.7 Hz, 1H), 6.27–6.15 (m, 2H), 5.72–5.46 (m, 2H), 5.27–5.04 (m, 1H), 3.83 (t, *J* = 9.7 Hz, 1H), 3.27–3.16 (m, 2H), 2.76–2.70 (m, 3H), 2.48–2.18 (m, 3H), 1.99–1.65 (m, 5H), 1.11 (d, *J* = 8.9 Hz, 6H), 0.56 (t, *J* = 18.5 Hz, 1H), 0.36 (m, 2H), 0.09 (m, 1H), 0.04 to −0.04 (m, 1H)·^13^C NMR (176 MHz, CD_3_CN, 298 K): δ 196.84,180.23, 170.34, 161.64, 158.34, 155.35, 131.57, 126.69, 118.91, 107.05, 60.22 (=N–CH-CO), 59.28 (=N–CH-CO), 54.59, 42.57, 40.95, 39.69, 36.38, 32.23, 32.00, 29.30, 25.86, 23.16, 8.32, 4.90, 4.30. ESI-HRMS: calcd for C_24_H_35_N_6_O_6_ [M + H]^+^, *m*/*z* = 503.2618 Da; found, *m*/*z* = 503.2619 Da.

##### (*S*)-3-((*S*)-2-(3-(3-(*tert*-Butyl) Ureido)-2-oxopyridin-1(2*H*)-yl)-3-cyclopropylpropanamido)-*N*-methyl-2-oxo-4-((*S*)-2-oxopyrrolidin-3-yl) Butanamide (**16b**)

Compound **16b** was prepared in two steps in a 45% yield by deacylation of acetoxy amide **14b** (19 mg, 0.0338 mmol) followed by oxidation with DMP (26.2 mg, 0.061 mmol) utilizing the same procedure as described for compound **6a**. d.r. ∼ 1:1 (*S*,*S*,*S*): (*R*,*S*,*S*), HPLC purity: 99.3%, ^1^H NMR (500 MHz, CD_3_CN, 298 K): δ 8.46–8.28 (d, *J* = 5.5 Hz; d, *J* = 5.4 Hz, 1H), 8.00 (td, *J* = 7.1, 1.7 Hz, 1H), 7.63 (s, 1H), 7.26 (br, 1H), 7.06 (dt, *J* = 7.1, 1.5 Hz, 1H), 6.24–6.11 (m, 2H), 5.70 (s, 1H), 5.57–5.45 (dd, *J* = 8.6, 6.8 Hz; m, 1H), 5.11–5.03 (m, 1H), 3.22 (ddd, *J* = 15.5, 9.2, 5.2 Hz, 2H), 2.72 (m, 3H), 2.49 (ddd, *J* = 15.1, 10.5, 6.5 Hz, 1H), 2.38–2.18 (m, 3H), 1.99–1.67 (m, 4H), 1.31 (s, 9H), 0.61–0.51 (m, 1H), 0.39–0.31 (m, 2H), 0.09 (tdd, *J* = 7.4, 4.8, 2.5 Hz, 1H), 0.02 to −0.05 (m, 1H). ^13^C NMR (176 MHz, CD_3_CN, 298 K): δ 195.37, 179.37, 169.42, 160.54, 157.33, 154.31, 130.65, 125.64, 106.16, 59.06 (=N–CH-CO), 58.34 (=N–CH-CO), 53.57, 50.06, 40.04, 38.75, 35.23, 31.26, 31.05, 28.37, 28.29, 24.93, 7.36, 3.94, 3.47. ESI-HRMS: calcd for C_25_H_37_N_6_O_6_ [M + H]^+^, *m*/*z* = 517.2774 Da; found, *m*/*z* = 517.2774 Da.

##### (*S*)-3-((*S*)-2-(3-(3-Cyclohexylureido)-2-oxopyridin-1(2*H*)-yl)-3-cyclopropylpropanamido)-*N*-methyl-2-oxo-4-((*S*)-2-oxopyrrolidin-3-yl) Butanamide (**16c**)

Compound **16c** was prepared in two steps in a 39% yield by deacylation of acetoxy amide **14c** (40 mg, 0.0682 mmol) followed by oxidation with DMP (42 mg, 0.099 mmol) utilizing the same procedure as described for compound **6a**. d.r. ∼ 1:1 (*S*,*S*,*S*): (*R*,*S*,*S*), HPLC purity: 93.5%, LC–MS purity: >95%, ^1^H NMR (700 MHz, CD_3_CN, 298 K): δ 8.40–8.22 (d, *J* = 5.1 Hz; *J* = 5.5 Hz, 1H), 7.94 (ddd, *J* = 11.5, 7.4, 1.7 Hz, 1H), 7.59 (d, *J* = 11.6 Hz, 1H), 7.19 (br, 1H), 7.00 (ddd, *J* = 6.0, 4.3, 1.7 Hz, 1H), 6.18–6.05 (m, 2H), 5.64 (d, *J* = 5.7 Hz, 1H), 5.51–5.38 (dd, *J* = 8.7, 6.7 Hz; m, 1H), 5.00 (dt, *J* = 9.1, 5.5 Hz, 1H), 3.50–3.41 (m, 1H), 3.20–3.07 (m, 2H), 2.65 (ddd, *J* = 18.9, 5.0, 2.8 Hz, 3H), 2.46–2.11 (m, 2H), 1.84–1.68 (m, 6H), 1.67–1.45 (m, 4H), 1.32–1.23 (m, 2H), 1.13 (ddd, *J* = 21.6, 15.6, 7.1 Hz, 3H), 0.55–0.42 (m, 1H), 0.34–0.22 (m, 2H), 0.06 to −0.03 (m, 1H), −0.04 to −0.14 (m, 1H). ^13^C NMR (176 MHz, CD_3_CN, 298 K): δ 196.34, 180.23, 170.34, 161.52, 158.30, 155.26, 131.57, 126.74, 118.86, 107.13, 60.14 (=N–CH-CO), 59.29 (=N–CH-CO), 54.55, 49.36, 40.87, 39.35, 35.96, 33.97, 32.23, 29.15, 26.36, 25.86, 8.27, 4.78, 4.42. ESI-HRMS: calcd for C_27_H_39_N_6_O_6_ [M + H]^+^, *m*/*z* = 543.2931 Da; found, *m*/*z* = 543.2928 Da.

##### (*S*)-3-((*S*)-2-(3-(3-Benzylureido)-2-oxopyridin-1(2*H*)-yl)-3-cyclopropylpropanamido)-*N*-methyl-2-oxo-4-((*S*)-2-oxopyrrolidin-3-yl) Butanamide (**16d**)

Compound **16d** was prepared in two steps in a 48% yield by deacylation of acetoxy amide **14d** (18 mg, 0.036 mmol) followed by oxidation with DMP (24.5 mg, 0.028 mmol) utilizing the same procedure as described for compound **6a**. d.r. ∼ 1:1 (*S*,*S*,*S*): (*R*,*S*,*S*), HPLC purity: 97.6%, ^1^H NMR (500 MHz, CD_3_CN, 298 K): δ 8.53–8.34 (ss, 1H), 8.12–8.00 (m, 1H), 7.82 (dd, *J* = 26.4, 12.9 Hz, 1H), 7.37–7.22 (m, 5H), 7.14–7.05 (m, 1H), 6.34–6.10 (m, 3H), 5.57–5.47 (dd, m, *J* = 8.9, 6.6 Hz, 1H), 5.12–5.03 (m, 1H), 4.42–4.26 (m, 2H), 3.28–3.14 (m, 2H), 2.80–2.65 (m, 3H), 2.54–2.20 (m, 3H), 2.12–2.03 (m, 1H), 1.91–1.68 (m, 4H), 0.63–0.50 (m, 1H), 0.41–0.29 (m, 2H), 0.12–0.04 (m, 1H), 0.03 to −0.06 (m, 1H). ^13^C NMR (126 MHz, CD_3_CN, 298 K): δ 195.73, 179.57, 169.32, 160.87, 157.31, 155.07, 140.03, 128.41, 127.17, 126.89, 126.01, 118.25, 106.06, 59.07 (=N–CH-CO), 58.28 (=N–CH-CO), 53.63, 43.15, 40.07, 39.98, 35.20, 31.10, 28.38, 24.90, 7.30, 3.91, 3.44. ESI-HRMS: calcd for C_28_H_35_N_6_O_6_ [M + H]^+^, *m*/*z* = 551.2618 Da; found, *m*/*z* = 551.2613 Da.

##### (*S*)-3-((*S*)-3-Cyclopropyl-2-(2-oxo-3-(3-phenylureido) Pyridin-1(2*H*)-yl)propanamido)-*N*-methyl-2-oxo-4-((*S*)-2-oxopyrrolidin-3-yl) Butanamide (**16e**)

Compound **16e** was prepared in two steps in a 40% yield by deacylation of acetoxy amide **14e** (27 mg, 0.046 mmol) followed by oxidation with DMP (39.4 mg, 0.0929 mmol) utilizing the same procedure as described for compound **6a**. d.r. ∼ 1:1 (*S*,*S*,*S*): (*R*,*S*,*S*), HPLC purity: 96.4%, ^1^H NMR (500 MHz, CD_3_CN, 298 K): δ 8.48–8.30 (dd, *J* = 5.2 Hz, *J* = 4.9 Hz, 1H), 8.06–7.82 (m, 3H), 7.35 (dd, *J* = 8.1, 2.5 Hz, 2H), 7.19 (dt, *J* = 26.9, 10.1 Hz, 3H), 7.09–7.01 (m, 1H), 6.92 (td, *J* = 7.4, 1.1 Hz, 1H), 6.20–6.04 (m, 2H), 5.50–5.40 (dd, *J* = 8.8, 6.7 Hz; dd, *J* = 8.8, 6.6 Hz, 1H), 4.99 (dt, *J* = 10.5, 5.6 Hz, 1H), 3.19–2.98 (m, 2H), 2.77–2.55 (m, 3H), 2.51–2.11 (m, 3H), 2.01–1.94 (m, 1H), 1.93–1.57 (m, 4H), 0.55–0.44 (m, 1H), 0.34–0.18 (m, 2H), 0.00 (dd, *J* = 9.0, 4.4 Hz, 1H), −0.04 to −0.14 (m, 1H). ^13^C NMR (126 MHz, CD_3_CN, 298 K): δ 179.58, 169.96, 161.15, 153.05, 140.10, 130.77, 129.38, 127.46, 123.08, 119.17, 106.53, 59.81 (=N–CH-CO), 58.95 (=N–CH-CO), 54.23, 44.60, 40.65, 39.46, 35.87, 35.61, 31.63, 28.91, 25.41, 7.91, 4.59, 4.10. ESI-HRMS: calcd for C_27_H_33_N_6_O_6_ [M + H]^+^, *m*/*z* = 537.2461 Da; found, *m*/*z* = 537.2456 Da.

##### (*S*)-*N*-Cyclopropyl-3-((*S*)-3-cyclopropyl-2-(2-oxo-3-(3-phenylureido)pyridin-1(2*H*)-yl)propanamido)-2-oxo-4-((*S*)-2-oxopyrrolidin-3-yl) Butanamide (**17**)

Compound **17** was prepared in two steps in a 35.9% yield by deacylation of acetoxy amide **15** (30 mg, 0.0495 mmol) followed by oxidation with DMP (37.6 mg, 0.0886 mmol) utilizing the same procedure as described for compound **6a**. d.r. ∼ 1:1 (*S*,*S*,*S*): (*R*,*S*,*S*), HPLC purity: 93.5%, LC–MS purity: >95% ^1^H NMR (700 MHz, CD_3_CN, 298 K): δ 8.62–8.37 (m, 2H), 8.19–8.04 (m, 2H), 7.53–7.39 (m, 2H), 7.36–7.24 (m, 2H), 7.19–7.11 (m, 1H), 7.08–6.99 (m, 1H), 6.32–6.16 (m, 2H), 5.64–5.48 (m, 2H), 5.26–5.04 (m, 1H), 3.32–3.15 (m, 2H), 2.76–2.64 (m, 1H), 2.55–2.23 (m, 3H), 2.05–1.95 (m, 1H), 1.89–1.64 (m, 4H), 0.75–0.68 (m, 2H), 0.63–0.52 (m, 2H), 0.41–0.34 (m, 2H), 0.15–0.07 (m, 1H), 0.04 to −0.04 (m, 1H). ^13^C NMR (176 MHz, CD_3_CN, 298 K): δ 195.39, 179.42, 169.26, 161.30, 157.35, 152.47, 139.45, 128.82, 126.56, 122.51, 118.88, 106.12, 59.18 (=N–CH–CO), 58.24 (=N–CH-CO), 53.72, 40.13, 38.59, 37.68, 35.28, 31.05, 28.28, 22.21, 9.95, 7.35, 5.26, 3.94, 3.83, 3.47. ESI-HRMS: calcd for C_29_H_35_N_6_O_6_ [M + H]^+^, *m*/*z* = 563.2618 Da; found, *m*/*z* = 563.2613 Da.

#### SARS-CoV-2 M^pro^ Inhibition Assay

The inhibitory activities versus the SARS-CoV-2 M^pro^ of all compounds were determined in a reaction buffer containing 20 mM HEPES, 120 mM NaCl, 0.4 mM EDTA, 20% glycerol, 4 mM DTT (freshly added before the measurements), pH 7.0, at 37 °C. A fluorescent substrate with the cleavage site (indicated by ↓) of SARS-CoV-2 M^pro^ (Dabcyl-KTSAVLQ↓SGFRKM-E(Edans)-NH_2_; Biosyntan) was used, and the fluorescence signal of the cleaved substrate was monitored using a Tecan Spark fluorescence spectrophotometer at an emission/excitation wavelength of 460/360 nm. Initially, 10 μL (per well) of the SARS-CoV-2 M^pro^ was pipetted into a 96-well plate with the corresponding drops containing 59 μL of the reaction buffer (final enzyme concentration: 50 nM). Subsequently, the inhibitors were added to give different final concentrations (0, 0.1, 0.4, 1.2, 3.7, 11, 33, and 100 μM). The inhibitor-M^pro^ mixture was incubated at 37 °C for 10 min. Finally, the reaction was initiated by adding 30 μL of the substrate dissolved in the reaction buffer with the corresponding concentrations described above.

All reactions were monitored at 37 °C for 15 min. Measurements of enzymatic activity were performed in triplicate, and the value was presented as mean ± standard deviation (SD). The initial velocity of the enzymatic reaction with compounds was calculated by linear regression for the first minute of the progression curve. Inhibition in % was obtained as (1 – (*V*_max_/*V*_0_)) × 100%. *V*_0_ is the velocity obtained for the DMSO control (inhibitor concentration of 0 μM). The log[% inhibition] was plotted against different concentrations of inhibitors (dose–response-inhibition). The IC_50_ values were calculated by nonlinear regression (Curve fit: Y: Bottom + (Top-Bottom)/(1 + 10̂ ((X-lg IC_50_))) using GraphPad Prism 9.0.

#### Melting Temperature (*T*_m_) Determination

To quantify the protein stability in complex with the α-ketoamide compounds, thermal unfolding was monitored using the nanoDSF (nanodifferential scanning fluorimetry) of the Prometheus NT.48 from NanoTemper Technologies. Ten μM SARS-CoV-2 M^pro^ was preincubated for 10 min at room temperature with 100 μM compound in a buffer containing 20 mM HEPES, 120 mM NaCl, 0.4 mM EDTA, 20% glycerol, pH 7.0, and freshly added 4 mM DTT. After loading the standard capillaries, the intrinsic fluorescence of the tryptophan and tyrosine residues was monitored at an emission wavelength of 350 nm while the temperature was increased by 0.5 °C/min. Measurements were performed using the PR.ThermControl software. The first derivative of the fluorescence signal at 350 nm was plotted as a function of temperature (Figure S3). The *T*_m_ ± SD was calculated by the PR. Stability Analysis software (Table 1). All measurements were performed in triplicate.

#### Cathepsin L Inhibition Assay

Z-Phe-Arg-MCA (methyl cumaryl amide) was purchased from Peptide Institute (3095-v). Cathepsin L (human liver) was purchased from Enzo Life Sciences (BML-SE201, UniProt ID P07711). All chemicals were of analytical grade. In the presence of α-ketoamides, cathepsin L activity was measured with Z-Phe-Arg-MCA as a substrate. Enzymes and compounds, at a final concentration of 25 nM and 10 μM, respectively, were preincubated for 10 min at 37 °C in 20 mM sodium acetate buffer, pH 5.5, containing 1 mM EDTA and 2.5 mM DTT. The final DMSO concentration in the assay was 1%. The reaction was started by the addition of Z-Phe-Arg-MCA to a final concentration of 10 μM, and fluorescence was followed at an excitation wavelength of 360 nm and an emission wavelength of 460 nm, using a Synergy H1 (Agilent BioTek) fluorescence spectrometer. Data collection and evaluation were carried out in a kinetic reading mode using the Gen5 data analysis software. The inhibition rates [%] were measured in triplicate and are presented as the mean ± SD.

#### ADME In Vitro Studies

The plasma stability assay, the metabolic stability assay, and the plasma protein binding assay were conducted as described previously.^19,33,34^ For the parallel artificial membrane permeation assay (PAMPA), the PAMP-096 kit from BioAssay Systems was used. Theophylline, chloramphenicol, and diclofenac were used as low-, high-, and medium-permeability controls, respectively. The assay was conducted as described in the manufacturer’s protocol. For all compounds, 10 mM DMSO stocks were used. Compound concentrations were determined using HPLC-MS/MS. For the HPLC-MS measurements, samples were analyzed using an Agilent 1290 Infinity II HPLC system coupled to an AB Sciex QTrap 6500plus. LC conditions were as described previously.^19^ MS/MS transitions can be found in Table S1.

#### Pharmacokinetic Studies

For pharmacokinetic experiments, outbred male CD-1 mice (Charles River, Germany), 4 weeks old, were used. The animal studies were conducted in accordance with the recommendations of the European Community (Directive 2010/63/EU, first January 2013). All animal procedures were performed in strict accordance with the German regulations of the Society for Laboratory Animal Science (GV-SOLAS) and the European Health Law of the Federation of Laboratory Animal Science Associations (FELASA). Animals were excluded from further analysis if sacrifice was necessary according to the human end points established by the ethical board. All experiments were approved by the ethical board of the Niedersächsisches Landesamt für Verbraucherschutz and Lebensmittelsicherheit, Oldenburg, Germany. Compounds **6a**, **6c**, **6d**, **9a**, **9b**, **12b-d**, **16c**, and **16e** were administered in cassette PK studies with a maximum of 5 compounds per study at 1 mg/kg intravenously (IV). Up to 25 μL of blood was collected from the lateral tail vein (*n* = 2 mice per study) at time points *t* = 0.25, 0.5, 1, and 3 h. At *t* = 5 h, animals were euthanized to collect blood. Whole blood was collected into Eppendorf tubes coated with 0.5 M EDTA and immediately spun down at 15,870 g for 10 min at 4 °C. Then, plasma was transferred into a new Eppendorf tube and stored at −80 °C until analysis. Bioanalytics were performed as described previously.^6^ PK parameters were determined using a noncompartmental analysis with PKSolver.^34^

#### Antiviral Activity Assays against SARS-CoV-2—Zagreb on A549^ACE2+TMPRSS2^ Cells

The SARS-CoV-2 Zagreb isolate (SARS-CoV2/ZG/297-20, University Hospital for Infectious Diseases, Zagreb, Croatia, GISAID database ID: EPI_ISL_451934),^35^ was grown on Vero E6 cells, and a titer of 7.25 × 10^5^ pfu/mL was determined for the virus stock by plaque assays. A549^ACE2+TMPRSS2^ cells were obtained from Goethe-University^28^ Frankfurt and cultured in Dulbecco’s modified Eagle medium (DMEM, Gibco REF 61965-026) supplemented with 10% fetal bovine serum (Gibco REF A5256801) and 1% l-glutamine (100×, Gibco REF A29168-01). For antiviral testing, cells were seeded in DMEM + 2% FBS at a density of 1.5 × 10^4^ cells/well in a 96-well flat-bottom cell culture plate (white, Greiner REF 655098) two days before the infection and incubated at 37 °C and 5% CO_2_. On the following day, the compounds were serially diluted in DMEM + 2% FBS and added to the cells. The compounds were added at a final volume of 100 μL in 10 different concentrations starting at either 50, 30, or 10 μM and diluted 2-fold. The cells were infected at the BSL-3 level with the SARS-CoV-2 Zagreb strain diluted in DMEM + 2% FBS at an MOI of 0.005 and incubated at 37 °C and 5% CO_2_. After 1 h, the medium containing the virus was removed and the medium containing the same compound concentrations as before was added back. On day three post-infection, the cell viability was analyzed by measuring the luminescence at a gain of 100 in a BioTek Synergy H4 Hybrid Microplate Reader. For that, 100 μL of the cell titer glo reagent (CellTiter-Glo Substrate (lyophilized, #G755A, Promega) and CellTiter-Glo Buffer (#G756A, Promega)) was added to each well and incubated for 10 min at room temperature. The measured luminescence was normalized as follows: [(*V*_sample_ – *V*_untreated_)/(*V*_uninfected_ – *V*_untreated_) × 100%], and EC_50_ values were calculated in GraphPad Prism version 9.4.1 using a nonlinear regression–exponential decay [[Inhibitor] vs response–Variable slope (four parameters)].

#### Antiviral Activity Assays against SARS-CoV-2-GHB on Vero E6-GFP Cells

The Vero E6 cell line engineered to constitutively express the enhanced green fluorescent protein (eGFP) was provided by Dr. Marnix Van Loock (Janssen Pharmaceutica, Beerse, Belgium) and was maintained in DMEM (Gibco cat. no. 41965-039) with 10% v/v heat-inactivated FCS and 500 μg/mL G418 (Gibco 10131-0275). The SARS-CoV-2 isolate used in this study was BetaCov/Belgium/GHB-03021/2020 (EPI ISL407976|2020-02-03), which was isolated from a Belgian patient returning from Wuhan in February 2020. The isolate was passaged 7 times on Vero E6 cells which introduced two series of amino acid deletions in the spike protein.^36^ The infectious content of the virus stock was determined by titration on Vero E6 cells.

The SARS-CoV-2 antiviral assay was derived from the previously established SARS-CoV assay^37^ and was performed in the certified, high-containment biosafety level-3 facilities of the Rega Institute at KU Leuven. In this assay, the fluorescence of Vero E6-eGFP cells declines after infection with SARS-CoV-2 due to the cytopathogenic effect of the virus. In the presence of an antiviral compound, the cytopathogenicity is inhibited and the fluorescence signal is maintained. On day 1, the test compounds were serially diluted in an assay medium (DMEM supplemented with 2% v/v FCS and 0.5 μM CP-100356). The plates were incubated (37 °C, 5% CO_2_, and 95% relative humidity) overnight. On day 0, the diluted compounds were then mixed with SARS-CoV-2 at 20 TCID50/well and Vero E6-eGFP cells corresponding to a final density of 25,000 cells/well in 96-well black-view plates (Greiner Bio-One, Vilvoorde, Belgium; Catalog 655090). The plates were incubated in a humidified incubator at 37 °C and 5% CO_2_. At 4 days p.i., the wells were examined for eGFP expression using an argon laser-scanning microscope. The microscope settings were excitation at 488 nm and emission at 510 nm, and the fluorescence images of the wells were converted into signal values. The results were expressed as EC_50_ values defined as the concentration of compound achieving 50% inhibition of the virus-reduced eGFP signals as compared to the untreated virus-infected control cells. The toxicity of compounds in the absence of a virus was evaluated in a standard MTS assay as reported previously.^38^

#### Crystallization and Diffraction Data Collection of M^pro^ in Complex with **6a**, **6c**, **6d**, **9a**, **9b**, **9c**, **12a**, **12d**, **12c**, and **16e**

Purified SARS-CoV-2 M^pro^ protein was concentrated to 15 mg/mL and then coincubated overnight with a 5-fold molar excess of different inhibitors at 4 °C. After being centrifuged at 13,000 rpm (rotor 1789 A), to remove precipitate, the protein–compound mixture was then subjected to cocrystallization screening with commercially available kits (PACT premierTM HT-96 and LFS Screen from Molecular Dimensions), using the vapor-diffusion sitting-drop method at 20 °C.

Diffraction data were collected from crystals grown under different conditions: **6a** (0.02 M sodium/potassium phosphate, 0.1 M bis-Tris propane, pH 6.5, 20% w/v PEG 3350), **6c** (0.1 M bis-Tris propane, pH 6.5, 2 M sodium formate, 20% w/v PEG 3350, 10% v/v ethylene glycol), **6d** (0.2 M sodium bromide, 0.1 M bis-Tris propane, pH 8.5, 20% w/v PEG 3350), **9a** (0.02 M sodium/potassium phosphate, 0.1 M bis-tris propane, pH 8.5, 20% w/v PEG 3350), **9b** (0.1 M bis-Tris propane, pH 7.5, 0.2 M sodium nitrate, 20% w/v PEG 3350, 10% v/v ethylene glycol), **9c** (0.1 M HEPES, pH 7, 0.1 M calcium chloride dihydrate, 20% w/v PEG 6000, 10% v/v ethylene glycol), **12a** (0.1 M MMT (dl-malic acid, MES monohydrate, Tris), pH 9.0, 25% w/v PEG 1500), **12c** (0.002 M zinc chloride, 0.1 M Tris, pH 8.0, 20% w/v PEG 6000), **12d** (0.1 M MES, pH 6, 0.2 M sodium chloride, 20% w/v PEG 6000, 10% v/v ethylene glycol), **16e** (0.1 M PCTP (sodium propionate, sodium cacodylate trihydrate, bis-Tris propane), pH 7.0, 25% w/v PEG 1500), and **9d** (0.1 M HEPES, pH 7.0, 0.2 M sodium chloride, 20% w/v PEG 6000, 10% v/v ethylene glycol). Fished crystals were flash-cooled using liquid nitrogen, and diffraction data were collected at 100 K at DESY PETRA III beamline P11 using a Pilatus 6 M detector (Dectris) and with synchrotron radiation of a wavelength of 1.0332 Å.

#### Diffraction Data Processing, Structure Solution, Refinement, and Modeling

XDSapp,^39^ Pointless,^40,41^ and Scala^40,41^ were used for data processing. Molrep^41,42^ was applied for solving the phase problem via the molecular replacement method using the SARS-CoV-2 M^pro^ free enzyme structure^43^ as a search model (PDB code: 6Y2E). All compounds were built into Fo–Fc difference density using the Coot software,^44^ and structures were refined using the Refmac5 program.^45^ Models of cathepsin L in complex with **6a**, **6c**, **6d**, **9a**, **9b**, **9c**, **12a**, **12c**, **12d**, **16e**, and **9d** were constructed using the electron density map of cathepsin L in complex with **13b** (PDB code: 8PRX) and refined using Refmac5. Pictures were made using Pymol.^46^ Diffraction data and model-refinement statistics are displayed in Tables S2 and S3.

## Supporting Information Available

Structures of synthesized final compounds; intermediate aldehyde (**4a-d**) synthesis; synthesis of ketoamides **6d-K** and **6d-H**; X-ray crystallographic figures of important final compounds; melting curves of α-ketoamides; mass transitions of the internal standard, caffeine, and of the inhibitors; diffraction data and model refinement statistics; ^1^H NMR and ^13^C NMR spectra of compounds; and HPLC and SFC traces (PDF)
Molecular formula strings (CSV)

## Abbreviations

Boc_2_O: Boc anhydride, ditert-butyl dicarbonate
DEAD: diethyl azodicarboxylate
DIPEA: diisopropylethylamine
DMP: Dess–Martin periodinane
EDC·HCl: *N*-(3-dimethylaminopropyl)-*N*′-ethylcarbodiimide hydrochloride
FRET: Förster resonance energy transfer
HATU: 1-[bis-(dimethylamino)methylene]-1*H*-1,2,3-triazolo[4,5-b]-pyridinium 3-oxide hexafluorophosphate
HEPES: 2-[4-(2-hydroxyethyl)piperazin-1-yl]ethanesulfonic acid
HOBt: 1-hydroxybenzotriazole hydrate
MCA: 7-methoxycoumarin-4-acetic acid
MES monohydrate: 2-(*N*-morpholino)ethanesulfonic acid
MMT buffer: sodium malonate/imidazole/boric acid solution
TBME: *tert*-butyl methyl ether
TEA: triethylamine
Tf_2_O: trifluoromethanesulfonic acid anhydride
TIPSCl: triisopropylsilyl chloride
TMSCl: trimethylsilyl chloride
