# Supplementary material for: Structure-Based Optimization of Pyridone α-Ketoamides as Inhibitors of the SARS-CoV-2 Main Protease
Source: J Med Chem. 2025 Jan 16;68(3):2920–41. doi: 10.1021/acs.jmedchem.4c02172 (PMC11831675; doi:10.1021/acs.jmedchem.4c02172)
Supplement: Supplementary file 1 — jm4c02172_si_001.pdf [file jm4c02172_si_001.pdf]

Supporting information for

Structure-based optimization of pyridone α-ketoamides as inhibitors of the SARS-CoV-2 main protease

Ravi Kumar Akula[1,2¶], Haifa El Kilani[2¶], Alina Metzen[1,3], Judith Röske[2], Kaixuan Zhang[2], Matthias Göhl[1], Nanaji Arisetti[1], Graham P. Marsh[5], Hannah J. Maple[5], Mark S. Cooper[5], Burhan Karadogan[5], Dirk Jochmans[6], Johan Neyts[6], Katharina Rox[1,3], Rolf Hilgenfeld[2,4]*, Mark Brönstrup[1,3,7]*

1 Department of Chemical Biology, Helmholtz Centre for Infection Research, Inhoffenstr. 7, 38124 Braunschweig, Germany

2 Institute of Molecular Medicine, University of Lübeck, Ratzeburger Allee 160, 23562 Lübeck, Germany

3 German Center for Infection Research (DZIF), Hannover-Braunschweig Site, 38124 Braunschweig, Germany

4 German Center for Infection Research (DZIF), Hamburg-Lübeck-Borstel-Riems Site, 23562 Lübeck, Germany

5 Bio-Techne (Tocris), Bristol BS11 9QD, U.K.

6 Department of Microbiology, Immunology and Transplantation, Rega Institute, KU Leuven, 3000 Leuven, Belgium

7 Institute of Organic Chemistry and Biomolecular Drug Research Centre (BMWZ), Leibniz University Hannover, Schneiderberg 1B, 30167, Hannover, Germany

¶ R. K. A. and H. E. K. contributed equally to this work

*Authors for correspondence

Contents	Pages
**Figure S1.** Structures of synthesized final compounds.	**S2**
Intermediate aldehyde (**4a**-**d**) synthesis	**S3-S13**
Synthesis of α-ketoamides **6d**-**K** & **6d**-**H**	**S13**
**Figure S2.** X-ray crystallographic figures of important final compounds.	**S14**
**Figure S3.** Melting curves of α-ketoamides	**S15**
**Table S1:** Mass transitions of the internal standard, caffeine, and of the inhibitors	**S16-S17**
**Table S2-S3.** Diffraction data and model refinement statistics	**S18- S22**
^{1}H NMR and ^{13}CNMR spectra of compounds	**S23-S48**
HPLC & SFC Traces	**S49-S60**
References	**S61**

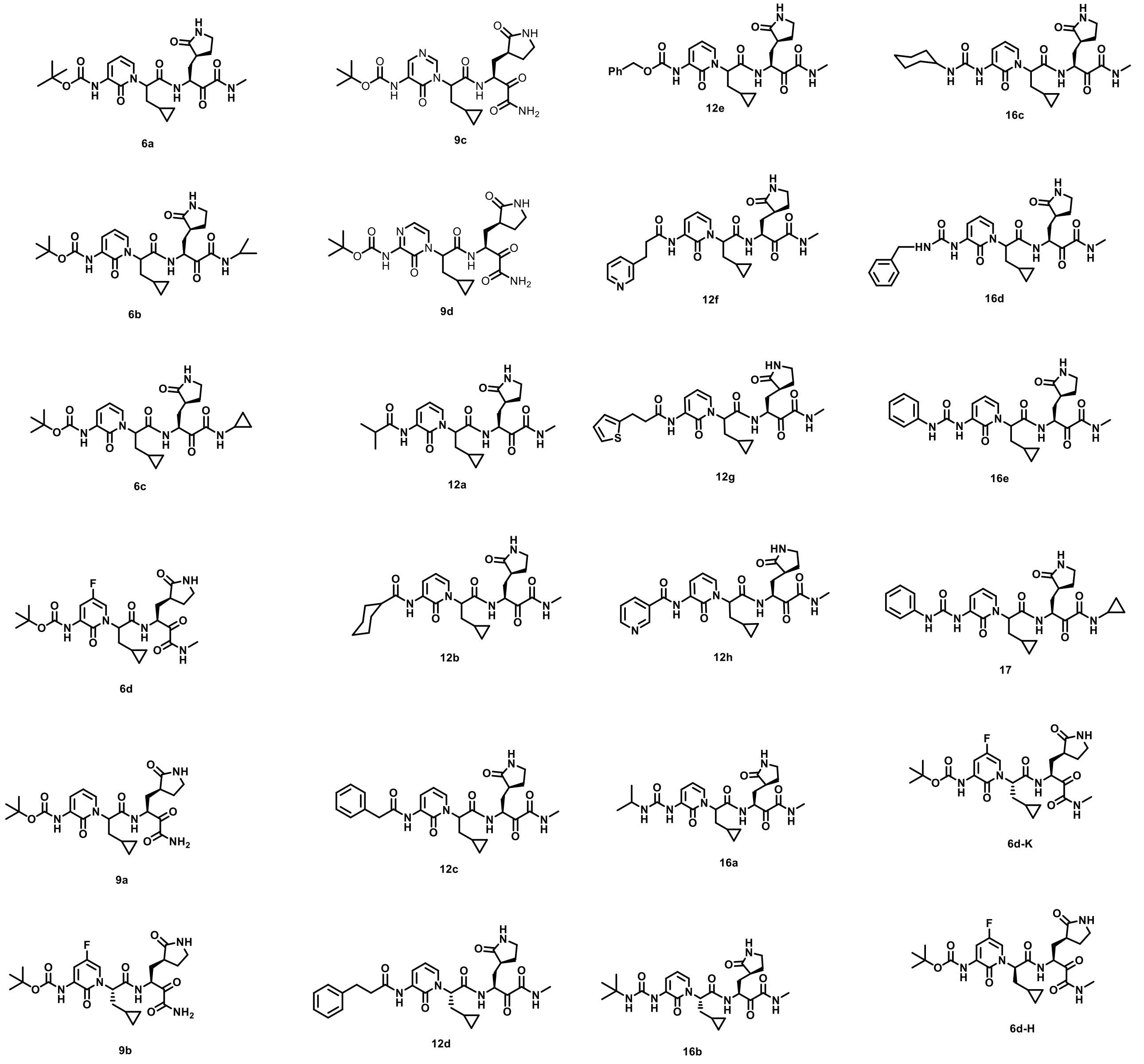

Figure S1. Structures of synthesized α-ketoamide compounds.

Intermediate aldehyde (4a-d) synthesis

*Synthesis of tert-butyl (1-((S)-3-cyclopropyl-1-oxo-1-(((S)-1-oxo-3-((S)-2-oxopyrrolidin-3-yl)propan-2-yl)amino)propan-2-yl)-2-oxo-1,2-dihydropyridin-3-yl)carbamate **4a**.*

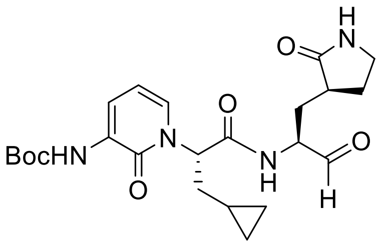

4a

*Methyl (R)-2-bromo-3-cyclopropylpropanoate(**1**).*

The (R)-2-amino-3-cyclopropylpropanoic acid (9.6 g, 0.074 mmol) and KBr (35.4 g, 0.296 mmol) was dissolved and stirred in 1M H_2SO_4 (160 mL) at 0 °C. A solution of $NaNO_2$ (30.6 g, 0.444 mmol) in H_2O (50 mL) was added dropwise over the course of 1.5 hours. The reaction mixture was stirred for further 3 hours at 0 °C and then allowed to stir at rt for further 16 hours. The mixture was extracted with ethyl acetate (3 x 100 mL) and the combined organic extracts were dried over Na_2SO_4 and evaporated to dryness. The title compound was obtained as a colorless liquid (12.98 g, 90%, m/z (ESI): 194 Da $[M+H^+]^-$), which was used directly without further manipulation.

To a solution of (R)-2-bromo-3-cyclopropylpropanoic acid (12.9 g, 0.0668 mol) in dry CH_3OH (160 mL) was added $SOCl_2$ (31.8 g, 0.267 mol) at 0 °C and stirred at ambient temperature for 16 hours. The solvent was removed in vacuo to provide (S)-methyl 2-bromo-3-cyclopropylpropanoate (**1**) as a colorless liquid (13.8 g, 90%, overall, two steps) used directly in the next reaction. ^{1}H NMR (700 MHz, DMSO-d_6, 298 K): δ 4.56 (t, J = 7.3 Hz, 1H), 3.72 (d, J = 3.7 Hz, 3H), 1.92 – 1.83 (m, 2H), 0.79 – 0.72 (m, 1H), 0.47 – 0.38 (m, 2H), 0.24 – 0.15 (m, 1H), 0.11 (td, J = 9.0, 5.0 Hz, 1H), ESI-MS (m/z): 206 Da $[M+H]^+$.

tert-Butyl (2-oxo-1,2-dihydropyridin-3-yl)carbamate was prepared based on the literature.[1]

*Methyl 2-(3-((tert-butoxycarbonyl)amino)-2-oxopyridin-1(2H)-yl)-3-cyclopropylpropanoate (**2a**).*

A mixture of (S)-methyl 2-bromo-3-cyclopropylpropanoate **1** (2.31 g, 0.011 mol), tert-butyl (2-oxo-1,2-dihydropyridin-3-yl)carbamate (2 g, 0.0074 mol) and Cs_2CO_3 (7.21 g, 0.022 mol) in CH_3CN (50 mL) was stirred at 50 °C overnight. The mixture was filtered and washed with MeCN (100

mL). The filtrate was concentrated in vacuo and the residue was purified by silica gel column (petroleum ether: EtOAc 2:1) to provide ethyl (S)-2-(3-((tert-butoxycarbonyl)amino)-2-oxopyridin-1(2H)-yl)-3-cyclopropylpropanoate (**2a**) as a yellow solid (1.74 g, 70%). ^{1}H NMR (700 MHz, DMSO-d_6, 298 K): δ 7.85 (dd, J_1 = 7.5 Hz, J_2 = 1.1 Hz, 1H), 7.80 (s, 1H), 7.40 (dd, J_1 = 7.0 Hz, J_2 = 1.7 Hz, 1H), 6.34 (t, J = 7.2 Hz, 1H), 5.23 (dd, J_1 = 9.8 Hz, J_2 = 5.2 Hz, 1H), 3.64 (s, 3H), 2.08 – 1.92 (m, 2H), 1.47 (s, 9H), 0.57 – 0.43 (m, 1H), 0.39 – 0.23 (m, 2H), 0.11 – 0.04 (m, 1H), −0.08 to −1.15 (m, 1H). ESI-MS (m/z): 337 Da [M+H]$^+$.

*Methyl (S)-2-(2-(3-((tert-Butoxycarbonyl) amino)-2-oxopyridin-1(2H)-yl)-3-cyclopropylpropanamido)-3-((S)-2-oxopyrrolidin-3-yl)-propanoate (**3a**).*

A stirred solution of methyl 2-(3-((tert-butoxycarbonyl)amino)-2-oxopyridin-1(2H)-yl)-3-cyclopropylpropanoate **2a (**1.74 g, 0.00517 mol) in methanol (24 mL) and water (6 mL) was treated with lithium hydroxide monohydrate (0.435 g, 0.0103mol) and the resulting solution was stirred at ambient temperature for 1.5 hours. The reaction mixture was concentrated and acidified to pH 5-6 with 1M HCl. The resulting solution was concentrated and extracted with CH_2Cl_2 (3 × 50 mL). The combined organic extracts were washed with brine (10 mL) dried over anhydrous magnesium sulfate and concentrated under reduced pressure to afford the title compound (S)-2-(3-((tert-butoxycarbonyl)amino)-2-oxopyridin-1(2H)-yl)-3-cyclopropylpropanoic acid as a white foam (1.63 g, 98%). A solution of (S)-2-(3-((tert-butoxycarbonyl)amino)-2-oxopyridin-1(2H)-yl)-3-cyclopropylpropanoic acid (905 mg, 2.81 mmol) in DMF (5 mL), HATU (1.922 g, 5.057 mmol) was added and stirred at 0 °C under dry conditions for 5 min. Reaction mixture was added to pre-stirred solution of methyl (S)-2-amino-3-((S)-2-oxopyrrolidin-3-yl)propanoate hydrochloride (1.06 g, 4.77 mmol) with triethyl amine (2.4 mL, 16.86 mmol) in DMF, CH_2Cl_2 at 0 °C. Reaction was continued for 6 hours in the same temp. Progress of the reaction was monitored based on TLC and LCMS. After completion of reaction, the mixture was added to ice cold water and extracted in CH_2Cl_2 (3 X 50mL). Resulted organic layer was washed with brine solution and dried over Na_2SO_4. Resulted organic was evaporated to dryness and purified on Flash Chromatography on C18 column (45% H_2O/ACN). Product **3a** was isolated with 1.3 g, 92% of yield over two steps. ^{1}H NMR (700 MHz, DMSO-d_6, 298 K): δ 8.88 (dd, *J*1 = 19.2 Hz, *J*2 = 7.3 Hz, 1H), 7.79 (d, J = 7.4 Hz, 1H), 7.75 (d, J = 2.4 Hz, 1H), 7.66 (d, J = 10.1 Hz, 1H), 6.29 (t, J = 7.1 Hz, 1H), 5.72 – 5.56 (m, 1H), 4.34 – 4.24 (m, 1H), 3.61 (d, J = 10 Hz, 3H), 3.19 – 3.03 (m, 2H), 2.30 – 2.17 (m, 1H), 2.16 – 1.90 (m, 3H), 1.85 – 1.71 (m, 1H), 1.67 – 1.53 (m, 2H), 1.46 (s, 9H), 0.57 – 0.41 (m, 1H), 0.36 – 0.27 (m, 2H), 0.17 – 0.09 (m, 1H), 0.03 to -0.05 (m, 1H). ESI-MS (m/z): 513 Da [M + Na+]$^+$.

*tert-Butyl (1-(3-Cyclopropyl-1-oxo-1-(((S)-1-oxo-3-((S)-2-oxopyrrolidin-3-yl)propan-2-yl)amino)propan-2-yl)-2-oxo-1,2-dihydropyridin- 3-yl)carbamate (**4a**).*

Methyl (S)-2-(2-(3-((tert-Butoxycarbonyl)amino)-2-oxopyridin-1(2H)-yl)-3-cyclopropylpropanamido)-3-((S)-2-oxopyrrolidin-3-yl)-propanoate (**3a**) (1.295 g, 3.42 mol) was dissolved in CH_2Cl_2 (20 mL). $LiBH_4$ solution (2M in THF, 1.97 mL, 3.95 mmol) was added to the above solution at 0 °C. The reaction mixture was warmed to rt and stirring was continued for 1.5 hours. The reaction was quenched with adding saturated NH_4Cl solution (10 mL). All volatiles were removed under reduced pressure and the aqueous residue was extracted with CH_2Cl_2 (3 x 50 mL). The combined organic layer was concentrated under reduced pressure and it was washed with brine solution and dried over Na_2SO_4. Crude product (1.21 g, quantitative.) was used for next reaction without purification. ESI-MS (m/z): 463 Da $[M + H]^+$.

To a stirred solution of tert-butyl (1-(3-cyclopropyl-1-(((S)-1-hydroxy-3-((S)-2-oxopyrrolidin-3-yl)propan-2-yl)amino)-1-oxopropan-2-yl)-2-oxo-1,2-dihydropyridin-3-yl)carbamate (1.58 g, 3.41 mmol) in CH_2Cl_2 (30 mL) at ambient temperature was added $NaHCO_3$ (0.029 g, 0.3419 mmol) and DMP (2.18 g, 5.14 mmol) and the solution was stirred for 2 hours. The reaction mixture was treated with 10% aqueous solution $Na_2S_2O_3$ (10 mL), saturated $NaHCO_3$ (aq. 5 mL). The organic phase was separated, and the aqueous component was extracted with CH_2Cl_2 (2 × 50 mL). The combined organic extracts was washed with saturated $NaHCO_3$ solution and was dried over anhydrous Na_2SO_4 and concentrated under reduced pressure to a white solid. Purification by flash column chromatography, eluting with 4% MeOH/CH_2Cl_2, afforded the title compound **4a** as a white solid (1.51 g, 96%).^{1}H NMR (700 MHz, DMSO-d_6, 298 K): δ 9.40 (d, J = 10.1 Hz, 1H), 8.81 (dd, J_1 = 12.9 Hz, J_2 = 7.3 Hz, 1H), 7.86 – 7.67 (m, 2H), 7.67 – 7.50 (m, 1H), 7.43 – 7.37 (m, 1H), 6.33 – 6.25 (m, 1H), 5.68 – 5.54 (m, 1H), 4.37 – 4.13 (m, 1H), 3.24 – 2.96 (m, 3H), 2.31 – 1.28 (m, 6H), 1.46 (s, 9H), 0.57 – 0.39 (m, 1H), 0.37 – 0.23 (m, 2H), 0.20 – 0.07 (m, 1H), 0.04 – 0.08 (m, 1H). ESI-MS (m/z): 461 Da $[M+H]^+$.

Synthesis of intermediate aldehyde 4b-d.

Scheme-S2: Synthesis of intermediate aldehyde (4b-d)[a].

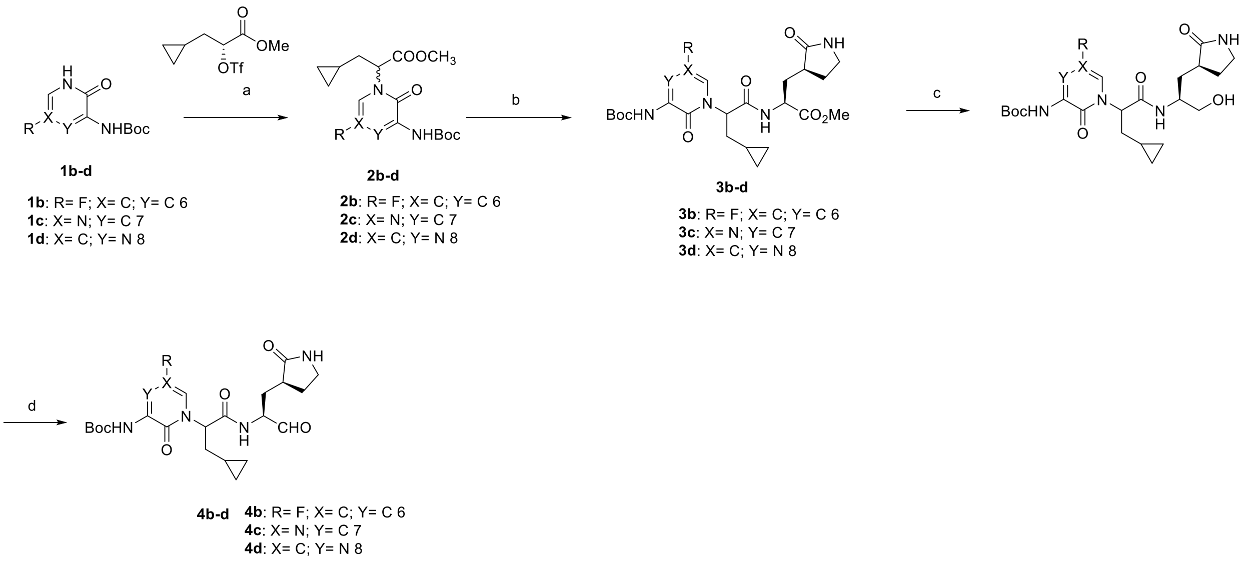

[a]**Reagent & conditions:** a) NaH, THF, 0 °C - rt, 31-54%; b) (i) LiOH.H_2O, MeOH, H_2O; (ii) methyl (*S*)-2-amino-3-((*S*)-2-oxopyrrolidin-3-yl)propanoate hydrochloride, EDC.HCl, HOBt, TEA, CH_2Cl_2, 18-64% over two steps; c) $NaBH_4$, MeOH, rt; d) IBX, acetone, reflux, 61-97% over two steps.

*tert-Butyl (5-fluoro-2-hydroxypyridin-3-yl)carbamate (***1b***).*

To a stirred solution of 3-amino-5-fluoropyridin-2-ol (5.30 g, 41.37 mmol) in THF (150 mL) was added di-*tert*-butyl dicarbonate (13.54 g, 62.06 mmol), and the reaction was heated to reflux. After 22 hours at reflux the reaction had only progressed to a small extent. Reflux was continued with two additional portions of di-*tert*-butyl dicarbonate (1 eq.) being added, the reaction was eventually stopped after 94 hours at reflux and a total of 3.5 eq., di-*tert*-butyl dicarbonate having been added. The reaction mixture was concentrated under reduced pressure to give a dark brown oil. Purification by dry-flash chromatography, eluting with DCM and then 2% MeOH/DCM, afforded the title compound as a light brown solid (3.11 g, 33%). ^{1}H NMR (400 MHz, DMSO-d_6, ambient temperature): δ 11.91 (br s, 1H), 7.89 (br s, 1H), 7.79 (dd, *J* = 9.7, 3.1, 1H), 7.21 – 7.19 (m, 1H), 1.46 (s, 9H).

*Synthesis of tert-Butyl (6-oxo-1,6-dihydropyrimidin-5-yl)carbamate (***1c***)[a].*

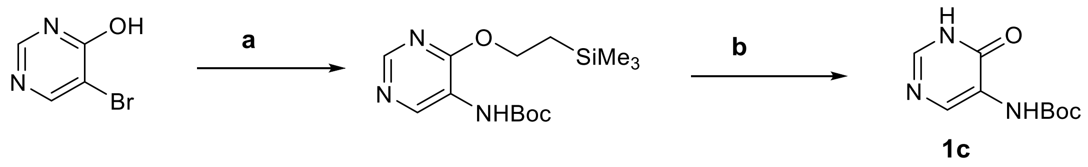

[a]**Reagent & conditions:** (a) (i) $Me_3SiCH_2CH_2OH$, PPh_3, DIAD, 1,4-dioxane, 62%; (ii) *t*-butylcarbamate, Cs_2CO_3, $Pd(dba)_2$, 1,4-dioxane, reflux, 70%; (b) TBAF, THF, 94%.

To a stirred suspension of 5-bromopyrimidin-4-ol (8.00 g, 45.72 mmol) in 1,4-dioxane (320 mL) at ambient temperature was added triphenylphosphine (43.17 g, 164.59 mmol) and 2-trimethylsilylethanol (19.46 g, 164.59 mmol). The reaction mixture was cooled to 5 °C and DIAD (32.41 mL, 164.59 mmol) was added dropwise over 10 minutes at such a rate so as to keep the temperature below 25 °C. The reaction mixture was stirred at ambient temperature for 4 hours and then was treated with water (1.2 L) before being extracted with DCM (2 x 800 mL). The combined organic extracts were washed with water (2 x 500 mL), dried over anhydrous magnesium sulfate, and concentrated under reduced pressure. The resulting residue was treated with petroleum ether (40:60) (400 mL), and the solid was filtered off, being washed further with petrol (400 mL), and was discarded. The filtrate was concentrated under reduced pressure to a pale yellow oil. Purification by flash column chromatography, eluting with 2% EtOAc/petroleum ether, afforded the title compound as a colorless oil (7.82 g, 62%). ^{1}H NMR (400 MHz, DMSO-d_6, ambient temperature): δ 8.64 (s, 1H), 8.56 (s, 1H), 4.58-4.54 (m, 2H), 1.23-1.17 (m, 2H), 0.10 (s, 9H).

A stirring mixture of 5-Bromo-4-(2-(trimethylsilyl)ethoxy)pyrimidine (7.70 g, 19.58 mmol), *tert*-butylcarbamate (2.52 g, 21.54 mmol), cesium carbonate (15.95 g, 48.96 mmol), $Pd_2(dba)_3$ (0.89 g, 0.98 mmol) and Xantphos (0.57 g, 0.98 mmol) in 1,4-dioxane (77 mL) was degassed and placed under an atmosphere of nitrogen, and then was heated at reflux for 7 hours. After cooling to ambient temperature, the reaction mixture was treated with ethyl acetate (80 mL) and the filtered through celite™, the filter cake being further washed with ethyl acetate (150 mL). The filtrate was washed with water (200 mL) followed by brine (200 mL), dried over anhydrous magnesium sulfate, and concentrated under reduced pressure. Purification by flash column chromatography, eluting with 10% EtOAc/petroleum ether, afforded the title compound as a white solid (4.25 g, 70%). (^{1}H NMR 400 MHz, DMSO-d_6, ambient temperature): δ 9.15 (br s, 1H), 8.44 (s, 1H), 6.75 (s, 1H), 4.55 – 4.50 (m, 2H), 1.52 (s, 9H), 1.22 – 1.16 (m, 2H), 0.09 (s, 9H).

To a stirred solution of *tert*-butyl (4-(2-(trimethylsilyl)ethoxy)pyrimidin-5-yl)carbamate (4.20 g, 13.49 mmol) in THF (70 mL) in an ice bath was dropwise added TBAF (26.97 mL, 26.97 mmol, 1M solution in THF), and the reaction mixture was allowed to warm to ambient temperature and stirred for 18 hours. The reaction mixture was concentrated under reduced pressure, and the residue was partitioned between DCM (100 mL) and water (50 mL). The organic phase was separated, and the aqueous component was extracted with DCM (50 mL). The combined organic extracts were washed with water (2 x 50 mL), dried over anhydrous magnesium sulfate, and concentrated under reduced pressure. Purification by flash column chromatography, eluting with 8% MeOH/DCM, afforded *tert-butyl (6-oxo-1,6-dihydropyrimidin-5-yl)carbamate (**1c**)* as a white solid (2.67 g, 94%). ^{1}H NMR (400 MHz, DMSO-d_6, ambient temperature): δ 12.77 (br s, 1H), 8.28 (s, 1H), 7.93 (s, 1H), 7.86 (s, 1H), 1.45 (s, 9H).

*tert-Butyl (3-oxo-3,4-dihydropyrazin-2-yl)carbamate (**1d**).*

To a stirred solution of 3-aminopyrazin-2-ol (5.00 g, 45.00 mmol) in THF (120 mL) was added di-tert-butyl dicarbonate (12.28 g, 56.29 mmol), and the reaction was heated to reflux. After 18 hours at reflux, the resulting suspension was cooled to ambient temperature and collected by filtration, the solid being washed with THF and dried on the sinter. This afforded an initial crop of product as a pale yellow solid (3.30 g). Concentration of the filtrate and subsequent trituration of the residue with diethyl ether afforded a second crop of product (1.45 g). In total, the title compound was obtained as a pale yellow solid (4.75 g, 42%). ^{1}H NMR (400 MHz, DMSO-d_6, ambient temperature): δ 12.24 (br s, 1H), 8.52 (br s, 1H), 7.05 (d, J = 4.3 Hz, 1H), 6.97 (d, J = 4.3 Hz, 1H), 1.45 (s, 9H). ES$^+$-MS (m/z): 112.2 Da [M+H+-Boc]+.

*Methyl (S)-2-(3-((tert-butoxycarbonyl) amino)-5-fluoro-2-oxopyridin-1(2H)-yl)-3-cyclopropylpropanoate (**2b**).*

Sodium hydride (0.42 g, 10.52 mmol, 60% dispersion in mineral oil) was added portion-wise to a stirred solution of *tert*-butyl *N*-(5-fluoro-2-oxo-1H-pyridin-3-yl)carbamate (3.00 g, 13.15 mmol) in THF (100 mL) at 0 °C. After stirring at this temperature for 90 mins, a solution of methyl (2*R*)-3-cyclopropyl-2-(trifluoromethylsulfonyloxy)propanoate (5.65 g, 18.40 mmol, 90% purity) in THF (50 mL) was added dropwise over a period of 10 mins maintaining the temperature at 0 °C. Stirring was maintained at this temperature for 4 hours and then the reaction mixture was allowed to warm to ambient temperature overnight. The reaction mixture was treated with water (4 mL), and then concentrated under reduced pressure. The residue was treated with DCM (120 mL) and filtered, the filter cake was washed with DCM, and the filtrate was concentrated under reduced pressure.

Purification by flash column chromatography, eluting with EtOAc/petroleum ether (40:60), afforded the title compound as a viscous pale yellow oil (2.53 g, 54%). ^{1}H NMR (400 MHz, DMSO-d_6, ambient temperature): δ 8.06 (s, 1H), 7.87 (dd, J_1 = 9.6 Hz, J_2 = 3.0 Hz, 1H), 7.65 (dd, *J* = 5.1, 3.1, 1H), 5.22 (dd, *J* = 9.9, 5.2, 1H), 3.63 (s, 1H), 2.10 – 1.90 (m, 2H), 1.46 (s, 9H), 0.57 – 0.47 (m, 1H), 0.38 – 0.24, m, 2H), 0.12 – 0.01 (m, 1H), -0.07 – -0.15 (m, 1H). ES$^+$-MS (m/z): 377.1 Da [M+Na$^+$]$^+$.

*Methyl (S)-2-(5-((tert-butoxycarbonyl)amino)-6-oxopyrimidin-1(6H)-yl)-3-cyclopropyl-propanoate (**2c**).*

Following the procedure described for the preparation of compound **2b** with a slight modification of conducting the reaction with tert-butyl (6-oxo-1,6-dihydropyrimidin-5-yl)carbamate **1c** (2.60 g, 9.85 mmol, 80% purity) and methyl (2R)-3-cyclopropyl-2-(trifluoromethylsulfonyloxy)propanoate (4.53 g, 14.77 mmol, 90% purity) afforded the compound **2c** (1.02 g, 31%). as yellow semi-solid. ^{1}H NMR (400 MHz, DMSO-d_6, ambient temperature): δ 8.34 (s, 1H), 8.28 (s, 1H), 8.11 (s, 1H), 5.23 (dd, *J* = 10.2, 5.1 Hz, 1H), 3.65 (s, 3H), 2.09-1.90 (m, 2H), 1.44 (s, 9H), 0.57 – 0.45 (m, 1H), 0.39 – 0.23 (m, 2H), 0.09 – 0.03 (m, 1H), -0.11 to -0.17 (m, 1H). ES$^+$-MS (m/z): 238.20 Da [M+H$^+$-Boc]$^+$.

*Methyl (S)-2-(3-((tert-butoxycarbonyl)amino)-2-oxopyrazin-1(2H)-yl)-3-cyclopropylpropanoate (**2d**).*

Following the procedure described for the preparation of compound **2b** with a slight modification of conducting the reaction with tert-butyl (3-oxo-3,4-dihydropyrazin-2-yl)carbamate **1d** (4.61 g, 21.83 mmol, 80% purity) and methyl (6.70 g, 21.83 mmol, 90% purity) afforded the compound **2d** (3.66 g, 50%) as colorless semi-solid. ^{1}H NMR (400 MHz, DMSO-d_6, ambient temperature): δ 8.74 (br s, 1H), 7.38 (d, *J* = 4.6 Hz, 1H), 7.08 (d, *J* = 4.6 Hz, 1H), 5.22 (dd, *J* = 10.0, 5.3 Hz, 1H), 3.65 (s, 3H), 2.06 – 1.90 (m, 2H), 1.44 (s, 9H), 0.58 – 0.48 (m, 1H), 0.38 – 0.26 (m, 2H), 0.11 – 0.04 (m, 1H), -0.05 to -0.11 (m, 1H).

*Methyl (S)-2-(2-(3-((tert-butoxycarbonyl)amino)-5-fluoro-2-oxopyridin-1(2H)-yl)-3-cyclopropylpropanamido)-3-((S)-2-oxopyrrolidin-3-yl)propanoate (**3b**).*

To a stirred solution of methyl (2*S*)-2-[3-(*tert*-butoxycarbonylamino)-5-fluoro-2-oxo-1-pyridyl]-3-cyclopropylpropanoic acid **2b** (2.45 g, 6.91 mmol) in methanol (65.3 mL) at ambient temperature was added water (13 mL) followed by lithium hydroxide monohydrate (0.58 g, 13.83 mmol). After stirring for 60 min at ambient temperature, the reaction mixture was acidified to pH 4 by addition

of 1N HCl (aq). The solution was partially concentrated under reduced pressure to remove the bulk of the methanol, and the remaining aqueous mixture was extracted with 10% MeOH/DCM (5 x 30 mL). The combined organic extracts were dried over anhydrous-magnesium sulfate and concentrated under reduced pressure, affording the title compound as a light beige solid (2.42 g, quant.). ^{1}H NMR (400 MHz, DMSO-d_6, ambient temperature): δ 7.95 (s, 1H), 7.80 (dd, J_1 = 9.3 Hz, J_2 = 3.1 Hz, 1H), 7.48 (m, 1H), 5.31 – 5.24 (m, 1H), 1.98 – 1.80 (m, 2H), 1.47 (s, 9H), 0.53 – 0.42 (m, 1H), 0.32 – 0.22, m, 2H), 0.04 – -0.04 (m, 1H), -0.07 to -0.14 (m, 1H). ES^--MS (m/z): 339.20 Da $[M\text{-}H^+]^-$.

To the above stirred solution of (*S*)-2-(3-(*tert*-butoxycarbonylamino)-5-fluoro-2-oxo-1-pyridyl)-3-cyclopropylpropanoic acid (2.35 g, 69.05 mmol) in DCM (60 mL) at 0 °C was added 1-hydroxybenzotriazole hydrate (1.63 g, 9.67 mmol) and EDC hydrochloride (1.85 g, 9.67 mmol) and the reaction mixture was stirred for 60 min. A solution of methyl (*S*)-2-amino-3-((*S*)-2-oxopyrrolidin-3-yl)propanoate hydrochloride (2.65 g, 7.94 mmol, 66.8% purity) and triethylamine (12.03 mL, 8.98 mmol) in DCM (25 mL) was added dropwise. Further triethylamine was added dropwise to adjust the reaction mixture to pH 9, after which point stirring was maintained at 0 °C overnight. The reaction mixture was treated with water (60 mL) and the organic phase was separated. The aqueous component was extracted with DCM (2 x 30 mL). The combined organic extracts were washed with saturated aqueous sodium hydrogen carbonate solution (30 mL) and brine (30 mL) before being dried over anhydrous magnesium sulfate and concentrated under reduced pressure. Purification by flash column chromatography, eluting with 2% MeOH/DCM, and subsequent trituration with petroleum ether (40:60) afforded the title compound as an off-white solid (2.29 g, 65%, *d.r.* approx. 2:1 (*(S,S,S)*:*(R,S,S)*)). ^{1}H NMR (400 MHz, DMSO-d_6, ambient temperature): δ 8.92 – 8.83 (m, 1H), 7.99 (s, 1H), 7.85 – 7.79 (m, 1H), 7.69 – 7.63 (m, 1H), 7.60 – 7.53 (m, 1H), 5.70 – 5.52 (m, 1H), 4.34 – 4.24 (m, 1H), 3.62 and 3.60 (2 x s, 3H), 3.17 – 3.04 (m, 2H), 2.28 – 1.95 (m, 3H), 1.83 – 1.70 (m, 1H), 1.67 – 1.54 (m, 2H), 1.47 and 1.46 (2 x s, 9H), 1.34 – 1.06 (m, 1H), 0.57 – 0.42 (m, 1H), 0.38 – 0.27 (m, 2H), 0.17 – 0.10 (m, 1H), 0.04 to -0.04 (m, 1H). m/z (ES^+): 531.2 Da $[M+Na^+]^+$.

*Methyl (S)-2-(2-(5-((tert-butoxycarbonyl)amino)-6-oxopyrimidin-1(6H)-yl)-3-cyclopropylpropanamido)-3-((S)-2-oxopyrrolidin-3-yl)propanoate (**3c**).*

Compound **3c** was prepared in two steps in 61.4% yield by saponification of methyl ester **2c** (0.98 g, 2.90 mmol) followed by peptide coupling reaction with methyl (S)-2-amino-3-((S)-2-oxopyrrolidin-3-yl)propanoate hydrochloride (0.97 g, 2.94 mmol, 67.5% purity) utilizing the same

procedure as described for compound **3b** afforded white solid Yield: (0.86 g, 74%, *d.r.* approx. 1:1 (*(S,S,S)*:*(R,S,S)*). ^{1}H NMR (400 MHz, DMSO-d_6, ambient temperature): δ 8.94 (dd, J = 18.8, 7.6 Hz, 1H), 8.31 – 8.22 (m, 2H), 7.99 (d, J = 3.4, 1H), 7.67 (d, J = 10.6 Hz, 1H), 5.59 – 5.41 (m, 1H), 4.35 – 4.25 (m, 1H), 3.63 – 3.60 (m, 3H), 3.19 – 3.05 (m, 2H), 2.35 – 1.99 (m, 4H), 1.95 – 1.82 (m, 1H), 1.66 – 1.54 (m, 2H), 1.45 – 1.44 (m, 9H), 0.59 – 0.45 (m, 1H), 0.36 – 0.29 (m, 2H), 0.17 – 0.11 (m, 1H), 0.02 to -0.04 (m, 1H). ES^{+}-MS (m/z): 514.20 [M+Na^{+}]$^{+}$; (ES^{-}): 490.30 Da [M-H^{+}]$^{-}$.

*Methyl (2S)-2-(2-(3-((tert-butoxycarbonyl)amino)-2-oxopyrazin-1(2H)-yl)-3-cyclopropylpropanamido)-3-((S)-2-oxopyrrolidin-3-yl)propanoate (**3d**).*

Compound **3d** was prepared in two steps in 41.7% yield by saponification of methyl ester **2d** (3.75 g, 11.15 mmol) followed by peptide coupling reaction with (S)-2-amino-3-((S)-2-oxopyrrolidin-3-yl)propanoate hydrochloride (4.19 g, 12.42 mmol, 66% purity) utilizing the same procedure as described for compound **3b** afforded pale yellow solid (2.38 g, 43%, *d.r.* approx. 2:1 (*(S,S,S)*:*(R,S,S)*). ^{1}H NMR (400 MHz, DMSO-d_6, ambient temperature): δ 8.94 – 8.86 (m, 1H), 8.67 (br s, 1H), 7.68 – 7.64 (m, 1H), 7.38 – 7.32 (m, 1H), 5.56 – 5.42 (m, 1H), 4.47 – 4.42 and 4.33 – 4.25 (2m, 1H), 3.63 – 3.61 (m, 3H), 3.18 – 3.06 (m, 2H), 2.30 – 1.90 (m, 4H), 1.85 – 1.74 (m, 1H), 1.67 – 1.52 (m, 2H), 1.44 (s, 9H), 0.60 – 0.45 (m 1H), 0.39 – 0.29 (m, 2H), 0.17 – 0.10 (m, 1H), 0.05 to -0.02 (m, 1H). ES^{+}-MS (m/z): 514.30 Da [M+Na^{+}]$^{+}$.

*tert-Butyl (1-(3-cyclopropyl-1-oxo-1-(((S)-1-oxo-3-((S)-2-oxopyrrolidin-3-yl)propan-2-yl)amino)propan-2-yl)-5-fluoro-2-oxo-1,2-dihydropyridin-3-yl)carbamate (**4b**).*

To a stirred solution of methyl (*S*)-2-(2-(3-((tert-butoxycarbonyl)amino)-5-fluoro-2-oxopyridin-1(2H)-yl)-3-cyclopropylpropanamido)-3-((*S*)-2-oxopyrrolidin-3-yl)propanoate **3b** **(**2.23 g, 4.39 mmol) in methanol (44 mL) at ambient temperature was portion-wise added sodium borohydride (0.83 g, 21.93 mmol), and the reaction mixture was stirred for 3 hours. Water (5 mL) was added, and then the pH was adjusted to pH 9 by dropwise addition of 1 M HCl. The reaction mixture was partially concentrated under reduced pressure to approximately 12 mL, and the resulting aqueous suspension was treated with water (10 mL) and DCM (40 mL) and then filtered. The layers of the biphasic filtrate were separated, and the aqueous component was extracted with DCM (2 x 20 mL). The combined organic extracts were dried over anhydrous magnesium sulfate and concentrated under reduced pressure, affording the title compound as a white solid (2.05 g, 97%) which was used in the next step without need for further purification. ^{1}H NMR (400 MHz, DMSO-d_6, ambient temperature): δ 8.16 – 8.08 (m, 1H), 7.98 (s, 1H), 7.73 (s, 1H), 7.82 (dd, J_1 = 9.7 Hz,

J_2 = 3.0 Hz, 1H), 7.62 – 7.51 (m, 2H), 5.60 – 5.48 (m, 1H), 4.69 (m, 1H), 3.75 (m, 1H), 3.39 – 3.30 (m, 1H), 3.29 – 3.20 (m, 1H), 3.18 – 3.00 (m, 1H), 2.20 – 1.95 (m, 3H), 1.85 – 1.68 (m, 2H), 1.64 – 1.30 (m, 2H), 1.47 (s, 9H), 0.55 – 0.41 (m, 1H), 0.38 – 0.25 (m, 2H), 0.19 – 0.08 (m, 1H), 0.04 to -0.04 (m, 1H). ES^--MS (m/z): 479.31 Da $[M-H^+]^-$.

To the above stirred solution of *tert*-butyl (1-(3-cyclopropyl-1-(((*S*)-1-hydroxy-3-((*S*)-2-oxopyrrolidin-3-yl)propan-2-yl)amino)-1-oxopropan-2-yl)-5-fluoro-2-oxo-1,2-dihydropyridin-3-yl)carbamate (2.00 g, 4.16 mmol) and sodium bicarbonate (0.12 g, 1.46 mmol) in DCM (193 mL) at ambient temperature was portion-wise added DMP (2.21 g, 5.20 mmol), and the reaction mixture was stirred for 2 hours. The reaction mixture was treated with saturated aqueous sodium bicarbonate solution (50 mL), and after stirring for 20 min, was filtered through celite™. The organic phase of the filtrate was separated, and the aqueous component was extracted with DCM (2 x 20 mL). The combined organic extracts were washed with brine (40 mL), dried over anhydrous magnesium sulfate and concentrated under reduced pressure. Purification by flash column chromatography, eluting with 4% MeOH/DCM, afforded the title compound as an off-white solid (2.00 g, 93% over two steps, ES^--MS (m/z): 477.31 Da $[M-H^+]^-$), which was used directly without further manipulation.

*tert-Butyl (1-(3-cyclopropyl-1-oxo-1-(((S)-1-oxo-3-((S)-2-oxopyrrolidin-3-yl)propan-2-yl)amino)propan-2-yl)-6-oxo-1,6-dihydropyrimidin-5-yl)carbamate (**4c**).*

Compound **4c** was prepared in two steps in 61% yield by reduction ester **3c** (0.83 g, 1.69 mmol) followed by oxidation with DMP (0.82 g, 1.93 mmol), utilizing the same procedure as described for compound **4b**, afforded it (**4c**) as an off-white solid (0.47 g, 460.3 Da $[M-H^+]^-$) which was used directly without further manipulation.

*tert-Butyl (4-(3-cyclopropyl-1-oxo-1-(((S)-1-oxo-3-((S)-2-oxopyrrolidin-3-yl)propan-2-yl)amino)propan-2-yl)-3-oxo-3,4-dihydropyrazin-2-yl)carbamate (**4d**).*

Compound **4d** was prepared in two steps in 79% yield by reduction ester **3d** (2.83 g, 5.76 mmol) followed by oxidation with DMP (2.75 g, 6.47 mmol), utilizing the same procedure as described for it **4d** was obtained as a white solid (2.40 g), which was used in the next step without need for further purification. Intermediate alcohol-^{1}H NMR (400 MHz, DMSO-d_6, ambient temperature): δ 8.66 – 8.63 (m, 1H), 8.18 – 8.11 (m, 1H), 7.57–7.52 (m, 1H), 7.40 – 7.35 (m, 1H), 7.03 (d, J = 4.3, 1H), 5.47– 5.36 (m, 1H), 4.72 – 4.66 (m, 1H), 3.75 (br s, 1H), 3.37 – 3.22 (m, 2H), 3.16 – 3.02 (m,

2H), 2.18 – 1.92 (m, 3H), 1.83 – 1.71 (m, 2H), 1.64 – 1.32 (m, 11H), 0.56 – 0.43 (m, 1H), 0.37 – 0.27 (m, 2H), 0.17 – 0.10 (m, 1H), 0.05 to -0.02 (m, 1H). ES^--MS (m/z): 462.3 Da $[M-H^+]^-$.

*tert-Butyl (1-((S or R)-3-cyclopropyl-1-(((S)-4-(methylamino)-3,4-dioxo-1-((S)-2-oxopyrrolidin-3-yl)butan-2-yl)amino)-1-oxopropan-2-yl)-5-fluoro-2-oxo-1,2-dihydropyridin-3-yl)carbamate (**6d-K & 6d-H**)*[a].

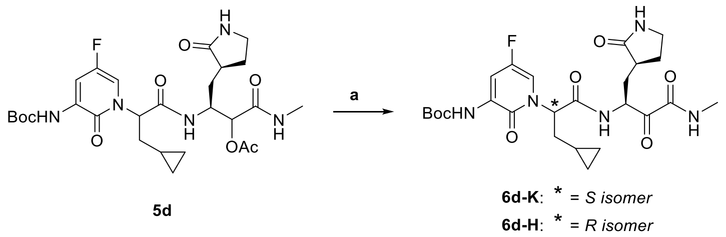

[a]Reagents and Conditions: (a) (i) $LiOH.H_2O$, MeOH, H_2O, 0 °C - rt, 1.5 h; (ii) HPLC diastereomer separation; (iii) DMP, DMF, rt, 3 h, 49% over two steps.

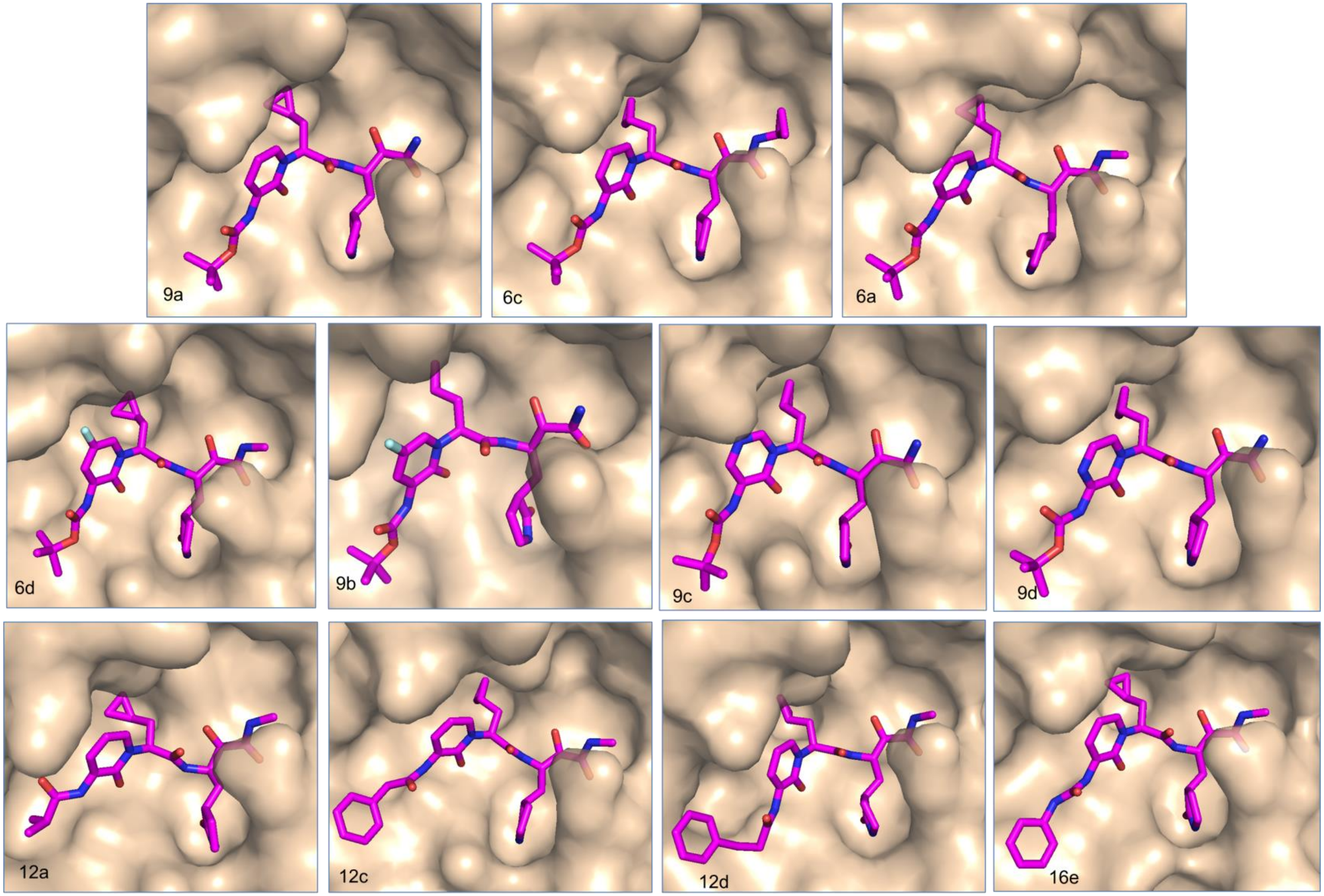

Figure S2. Overall illustration of crystal structures of M^{pro} in complex with **9a** (PDB: 9GMQ), **6c** (PDB: 8AIU), **6a** (PDB: 8AIV), **6d** (PDB: 9F2V), **9b** (PDB: 9F2V), **9c** (PDB: 9FHQ), **9d** (PDB: 9F2X), **12a** (PDB: 9F39), **12c** (PDB: 8AIZ), **12d** (PDB: 8AJ0) and **16e** (PDB: 8AJ1).

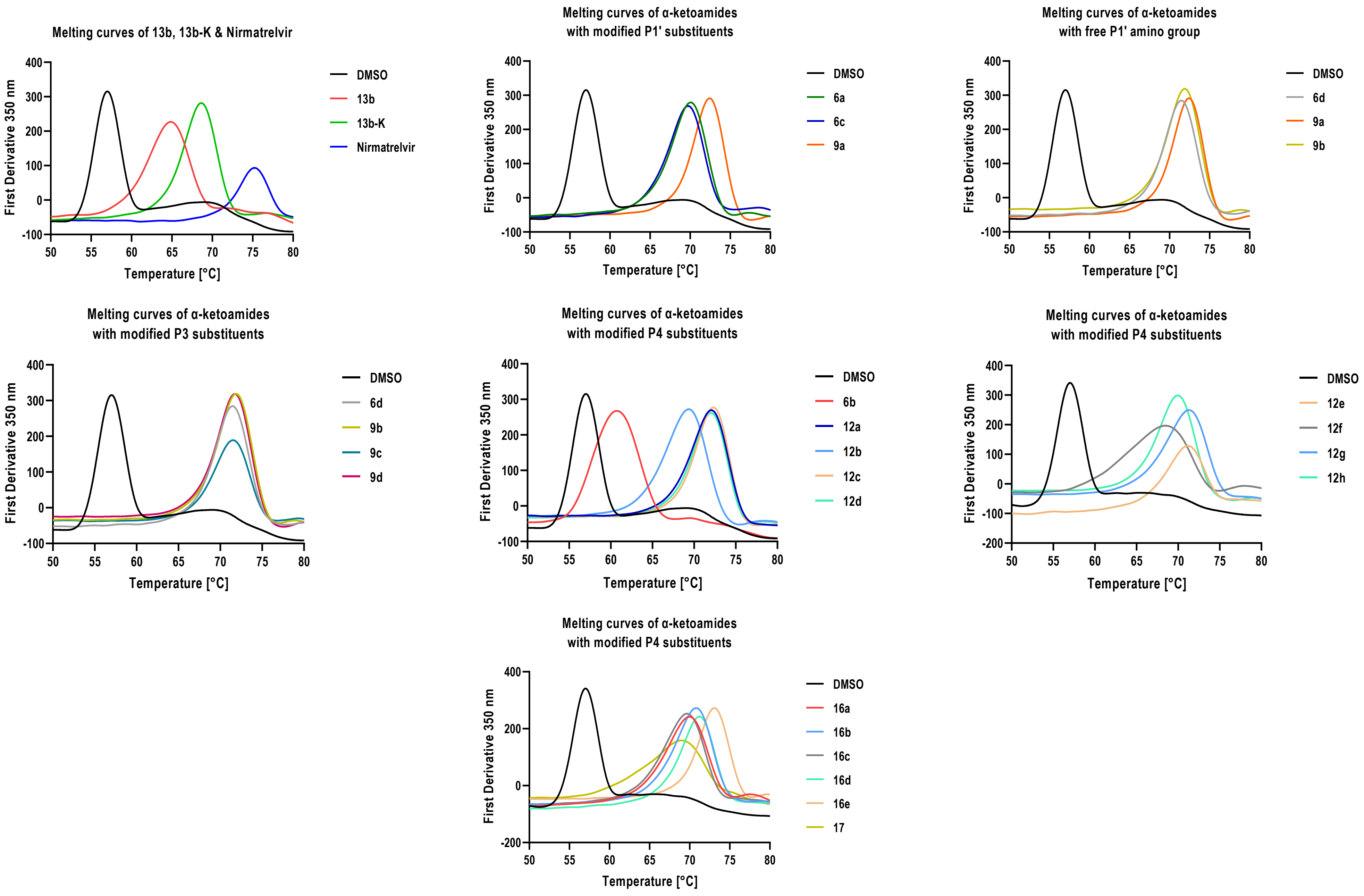

Figure S3: Melting curves of α-ketoamides**.**

Table S1: Mass transitions of the internal standard, caffeine, and of the inhibitors 6a, 6c, 6d, 9a, 9b, 12a-d, 12g, 16c-e.

Code	Q1 mass	Q3 mass	DP [volt]	CE [volts]	CXP [volts]
Caffeine	195.024	138.0	130.0	25.0	14.0
Caffeine	195.024	110.0	130.0	31.0	18.0
6a	518.133	305.1	66.0	17.0	16.0
6a	518.133	249.0	66.0	35.0	20.0
6c	544.199	305.1	101.0	17.0	20.0
6c	544.199	249.0	101.0	33.0	28.0
6d	536.209	267.2	151.0	31.0	22.0
6d	536.209	323.0	151.0	17.0	30.0
9a	504.078	304.9	91.0	17.0	16.0
9a	504.078	248.9	91.0	33.0	36.0
9b	543.968	444.4	131.0	31.0	52.0
9b	543.968	271.1	131.0	47.0	12.0
9b	522.173	323.0	71.0	17.0	20.0
9b	522.173	266.9	71.0	29.0	42.0
12a	487.900	214.2	71.0	15.0	10.0
12a	487.900	177.0	71.0	33.0	4.0
12b	528.203	315.0	66.0	19.0	40.0
12b	528.203	177.1	66.0	45.0	10.0
12c	537.171	324.1	66.0	19.0	20.0
12c	537.171	323.1	66.0	19.0	18.0
12d	550.155	337.1	91.0	17.0	16.0
12d	550.155	177.0	91.0	49.0	20.0
12g	556.137	343.0	116.0	19.0	42.0
12g	556.137	177.1	116.0	45.0	10.0
16c	543.229	330.1	1.0	19.0	18.0
16c	543.229	205.0	1.0	25.0	12.0

16d	551.159	338.0	46.0	19.0	52.0
16d	551.159	230.9	46.0	39.0	24.0
16e	537.143	323.b	101.0	19.0	34.0
16e	537.143	177.0	101.0	45.0	18.0

Table S2. Diffraction data and model refinement statistics.

Protein / Ligand	M^{pro} with 9a	M^{pro} with 6c	M^{pro} with 6a	M^{pro} with 12c	M^{pro} with 12d	M^{pro} with 16e
**PDB entry**	9GMQ	8AIU	8AIV	8AIZ	8AJ0	8AJ1
**Data collection statistics**						
**X-ray source**	DESY P11	DESY P11	DESY P11	DESY P11	DESY P11	DESY P11
**Wavelength [Å]**	1.0332	1.0332	1.0332	1.0332	1.0332	1.0332
**Space group**	$P2_1$	$P2_1$	$P2_1$	$C2$	$P2_1$	$C2$
**Unit cell dimensions [Å]**	*a* = 45.94, *b* = 63.77, *c* = 103.51	*a* = 45.77, *b* = 63.65, *c* = 103.64	*a* = 45.83, *b* = 63.40, *c* = 103.37	*a* = 113.80, *b* = 52.80, *c* = 45.35	*a* = 45.25, *b* = 54.37, *c* = 114.19	*a* = 113.23, *b* = 52.96, *c* = 45.71
**Unit cell dimensions [º]**	α = γ = 90, β = 91.25	α = γ = 90, β = 91.05	α = γ = 90, β = 91.10	α = γ = 90, β = 103.08	α = γ = 90, β = 101.38	α = γ = 90, β = 102.81
**Resolution range [a] [Å]**	45.93 - 2.19 (2.26 - 2.19)	42.16 - 2.00 (2.07 - 2.0)	40.06 - 2.60 (2.69- 2.60)	47.67 - 1.99 (2.06 -1.99)	48.91 - 2.52 (2.61 - 2.52)	47.75 - 2.60 (2.72 - 2.60)
**Number of observations**	210,367 (16,339)	139,149 (10,188)	63,577 (6,670)	121,927 (8,387)	89,995 (10,362)	28,073 (3,642)
**Number of unique reflections**	30,543 (2,906)	38,631 (3,871)	18,139 (1,763)	17,855 (1,281)	18,386 (1,790)	8,126 (1,006)
**Completeness [%]**	98.6 (95.8)	95.7 (95.5)	98.3 (95.3)	98.9 (96.8)	98.6 (98.6)	98.6 (99.4)
**Mean I/σ(I)**	10.9 (3.6)	14.9 (7.1)	8.0 (2.3)	13.1 (3.4)	6.4 (2.0)	7.1 (0.8)
**Multiplicity**	6.9 (6.5)	3.6 (3.6)	3.5 (3.1)	6.8 (6.5)	4.9 (5.1)	3.5 (3.6)
**R_{merge} [b] [%]**	0.133 (0.610)	0.054 (0.159)	0.142 (0.517)	0.081 (0.558)	0.211 (1.025)	0.108 (1.480)
**R_{pim} [c] [%]**	0.055 (0.256)	0.033 (0.097)	0.088 (0.351)	0.033 (0.233)	0.106 (0.505)	0.068 (0.911)

**$CC_{1/2}$ [d]**	0.996 (0.852)	0.997 (0.976)	0.989 (0.721)	0.998 (0.916)	0.988 (0.599)	0.996 (0.508)
**Wilson *B*-factor [Å^2]**	25	19	32	34	32	63
**Refinement statistics**						
**R_{cryst} [e] /R_{free} [f] [%]**	16.9/22.2	16.0/20.5	18.4/26.5	18.2/22.0	18.7/26.4	19.0/27.9
**r.m.s.d. in bond lengths [Å]**	0.014	0.014	0.014	0.013	0.014	0.013
**r.m.s.d. in bond angles [°]**	1.9	1.9	1.9	1.8	2.0	1.8
**Clashscore [g]**	2	4	5	2	6	4
**Average B-factor for protein atoms [Å^2]**	28	21	34	33	33	72
**Average B-factor for ligand atoms [Å^2]**	29	22	34	41	31	71
**Average B-factor for water molecules [Å^2]**	29	29	25	40	26	62
**Number of protein atoms in**	4734	4705	4734	2367	4673	2367
**Number of ligand atoms**	71	79	75	40	82	39
**Number of water molecules**	180	315	54	68	72	42
**Ramachandran plot**						
**Preferred regions [%]**	97	98	93	98	94	90
**Allowed regions [%]**	2	2	6	2	6	10
**Outlier regions [%]**	1	0	1	0	0	0

Table S3. Diffraction data and model refinement statistics

Protein / Ligand	M^{pro} with 12a	M^{pro} with 6d	M^{pro} with 9b	M^{pro} with 9d	M^{pro} with 9c
**PDB entry**	9F39	9F3A	9F2V	9F2X	9FHQ
**Data collection statistics**					
**X-ray source**	DESY P11	DESY P11	DESY P11	DESY P11	DESY P11
**Wavelength [Å]**	1.0332	1.0332	1.0332	1.0332	1.0332
**Space group**	*C*2	$P2_1$	$P2_1$	$P2_1$	$P2_1$
**Unit cell dimensions [Å]**	*a* = 113.70, *b* = 53.19, *c* = 44.85	*a* = 45.25, *b* = 62.87, *c* = 97.49	*a* = 45.84, *b* = 63.68, *c* = 103.50	*a* = 45.78, *b* = 63.55, *c* = 103.31	*a* = 45.86, *b* = 64.08, *c* = 98.48
**Unit cell dimensions [º]**	α = γ = 90, β = 102.44	α = γ = 90, β = 94.08	α = γ = 90, β = 91.17	α = γ = 90, β = 90.88	α = γ = 90, β = 93.74
**Resolution range [a] [Å]**	47.97 - 2.45 (2.538 - 2.45)	48.62 - 2.15 (2.227 - 2.15)	45.84 - 2.19 (2.27 - 2.19)	45.78 - 1.9 (1.968 - 1.9)	38.99 - 1.7 (1.761 - 1.7)
**Number of observations**	67,584 (7,850)	206,597 (17,881)	208,789 (19284)	181,030 (11,580)	426,239 (42,264)
**Number of unique reflections**	9,623 (976)	29,646 (2952)	30,461 (2890)	46,326 (4605)	62,214 (6184)
**Completeness [%]**	98.34 (98.89)	99.18 (99.86)	98.79 (95.47)	98.78 (99.33)	99.11 (99.45)
**Mean I/σ(I)**	6.2 (1.1)	6.3 (1.5)	19.9 (9.7)	9.8 (2.0)	13.8 (2.5)
**Multiplicity**	7.0 (7.2)	7.0 (7.0)	6.9 (6.7)	3.9 (3.9)	6.9 (6.8)
**R_{merge} [b] [%]**	0.250 (2.287)	0.255 (1.566)	0.087 (0.2641)	0.079 (0.609)	0.091 (0.918)
**R_{pim} [c] [%]**	0.101 (0.908)	0.104 (0.633)	0.036 (0.109)	0.045 (0.349)	0.037 (0.375)

**$CC_{1/2}$ [d]**	0.993 (0.585)	0.989 (0.559)	0.997 (0.973)	0.998 (0.758)	0.998 (0.817)
**Wilson *B*-factor [Å^2]**	41	31	21	27	22
**Refinement statistics**					
**R_{cryst} [e] /R_{free} [f] [%]**	22.9/31.1	21.2/27.6	17.6/21.6	18.0/22.6	18.2/21.4
**r.m.s.d. in bond lengths [Å]**	0.007	0.008	0.010	0.009	0.010
**r.m.s.d. in bond angles [°]**	1.3	1.6	1.7	1.6	1.7
**Clashscore [g]**	6	5	3	3	4
**Average B-factor for protein atoms [Å^2]**	53	35	24	30	27
**Average B-factor for ligand atoms [Å^2]**	50	34	24	30	24
**Average B-factor for water molecules [Å^2]**	35	34	26	37	31
**Number of protein atoms in**	2358	4691	4714	4707	4734
**Number of ligand atoms**	35	76	74	72	72
**Number of water molecules**	24	138	111	343	196
**Ramachandran plot**					
**Preferred regions [%]**	89	96	98	97	98
**Allowed regions [%]**	9	4	2	3	2
**Outlier regions [%]**	1	0	0	0	0

[a] The highest resolution shell is shown in parantheses.

[b] $R_{merge} = \sum_{hkl}\sum_{i=1}^{n}\left|I_i(hkl) - \bar{I}(hkl)\right| / \sum_{hkl}\sum_{i=1}^{n} I_i(hkl)$

[c] $R_{pim} = \sum_{hkl}\sqrt{1/(n-1)}\sum_{i=1}^{n}\left|I_i(hkl) - \bar{I}(hkl)\right| / \sum_{hkl}\sum_{i=1}^{n} I_i(hkl)$ [2]

[d] $CC_{1/2}$ is the correlation coefficient determined by two random half data sets [3]

[e] $R_{cryst} = \sum_{hkl}|F_o(hkl) - F_c(hkl)|/\sum_{hkl}|F_o(hkl)|$

[f] R_{free} was calculated for a test set of reflections (5%) omitted from the refinement.

[g] Clashscore is defined as the number of clashes calculated for the model per 1000 atoms (including hydrogens) of the model. Hydrogens were added by MolProbity [4]

^{1}H NMR and ^{13}CNMR spectra compounds

^{1}H-NMR (700 MHz, DMSO-d_6) of Compound 1.

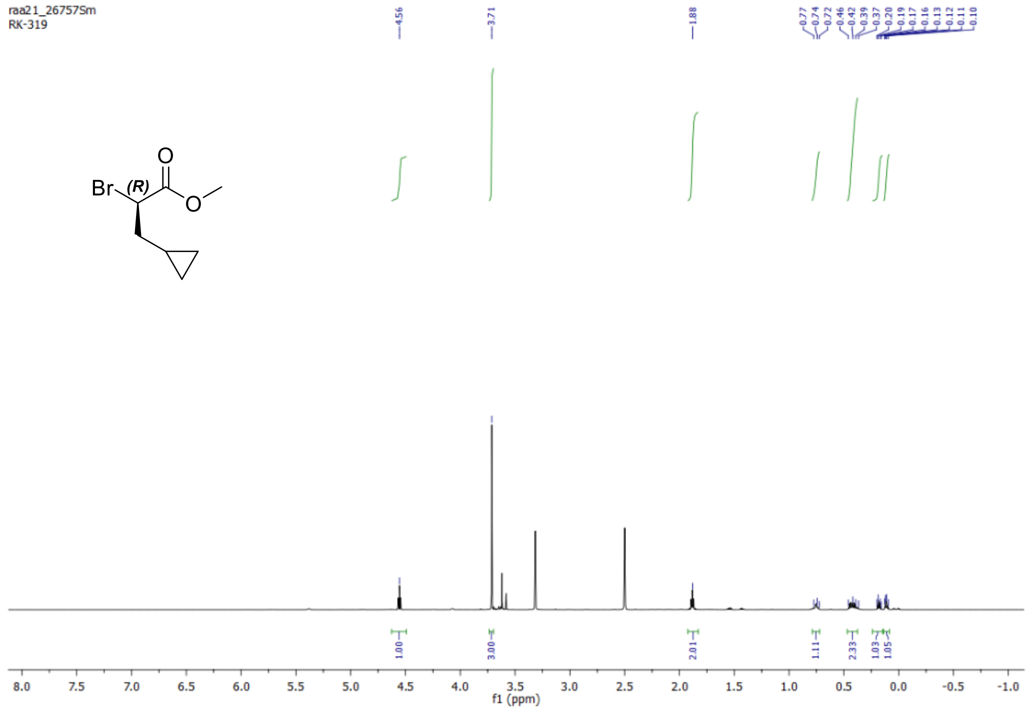

^{1}H-NMR (700 MHz, DMSO-d_6) of Compound 2a.

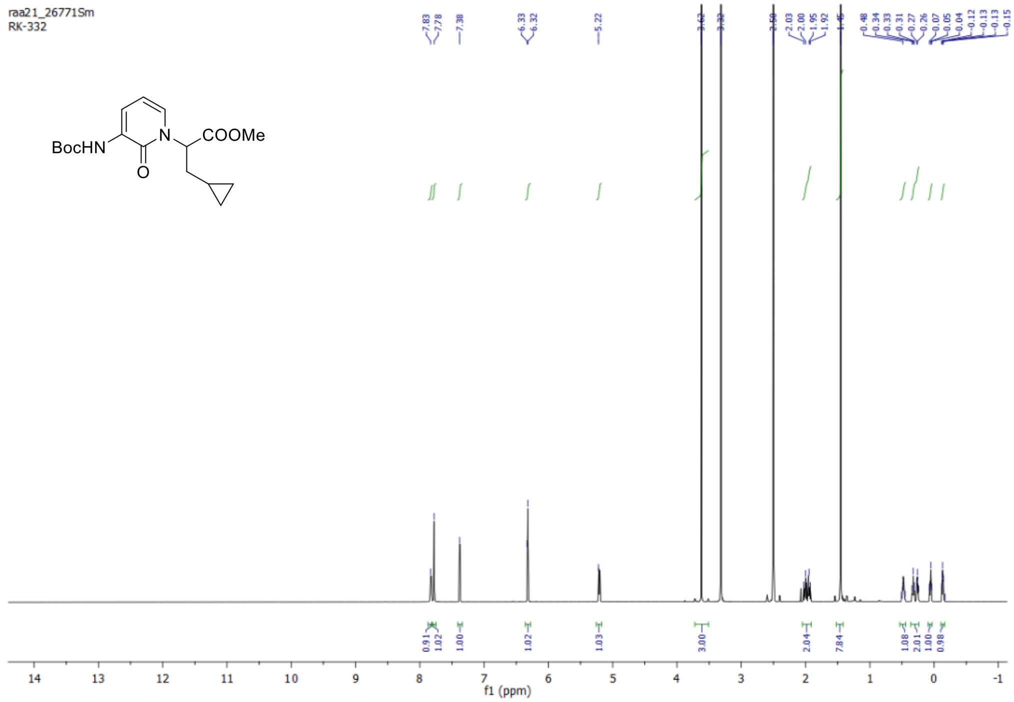

¹H-NMR (700 MHz, DMSO-d_6) of Compound 3a.

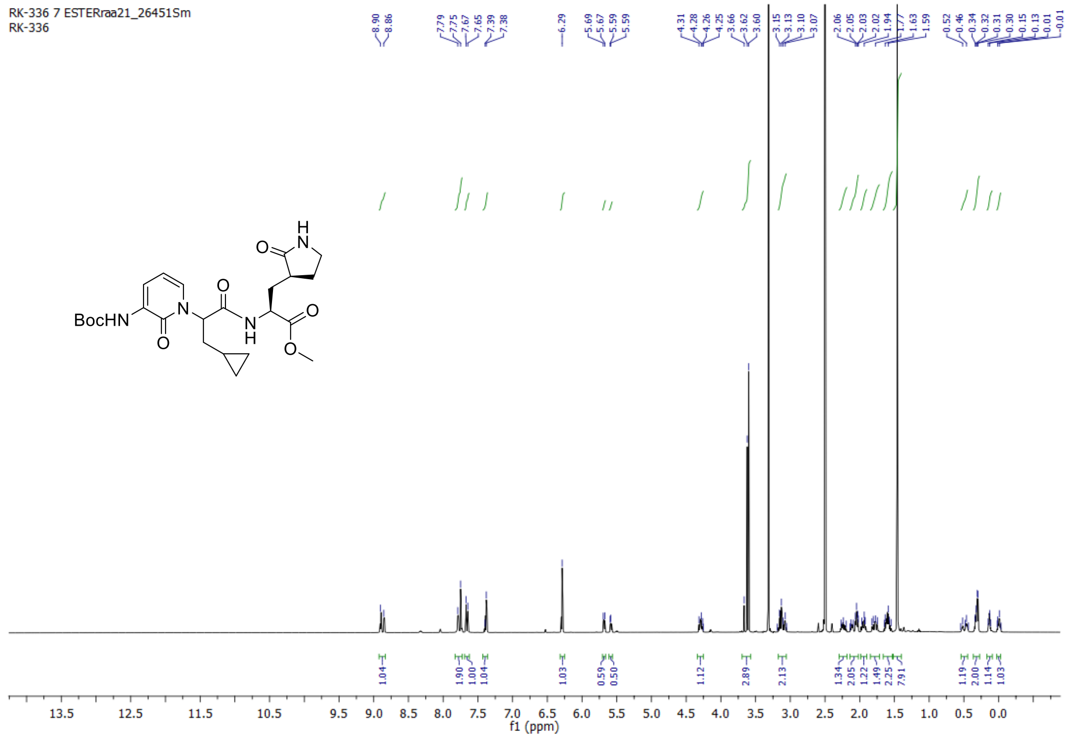

¹H-NMR (700 MHz, DMSO-d_6) of Compound 4a.

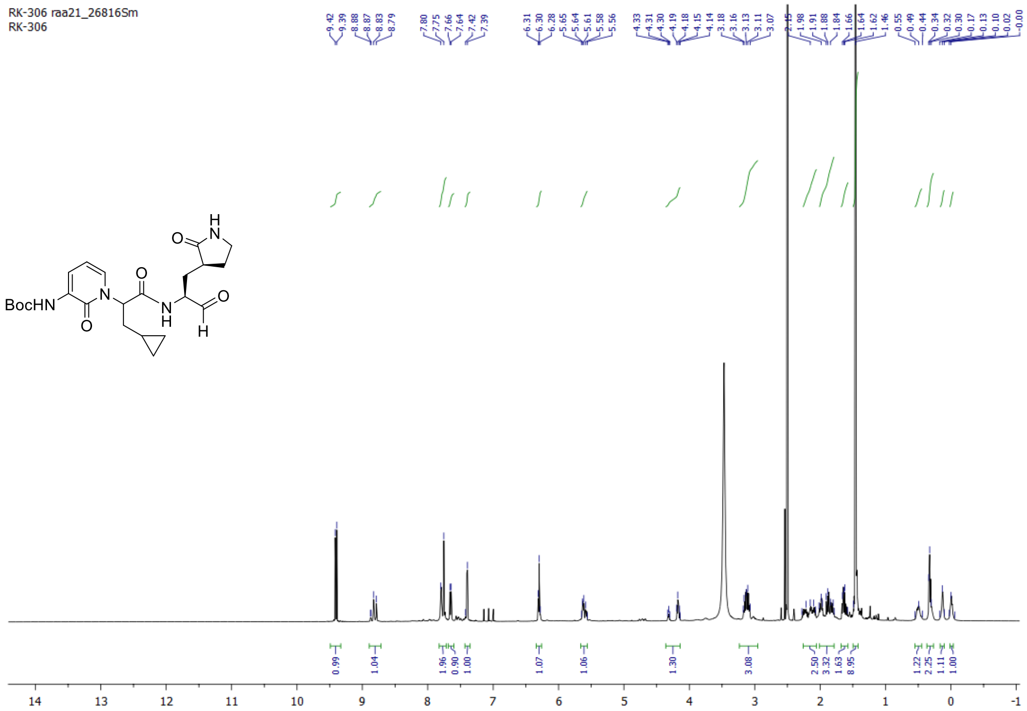

H-NMR (500 MHz, CD_3CN) of 6a.

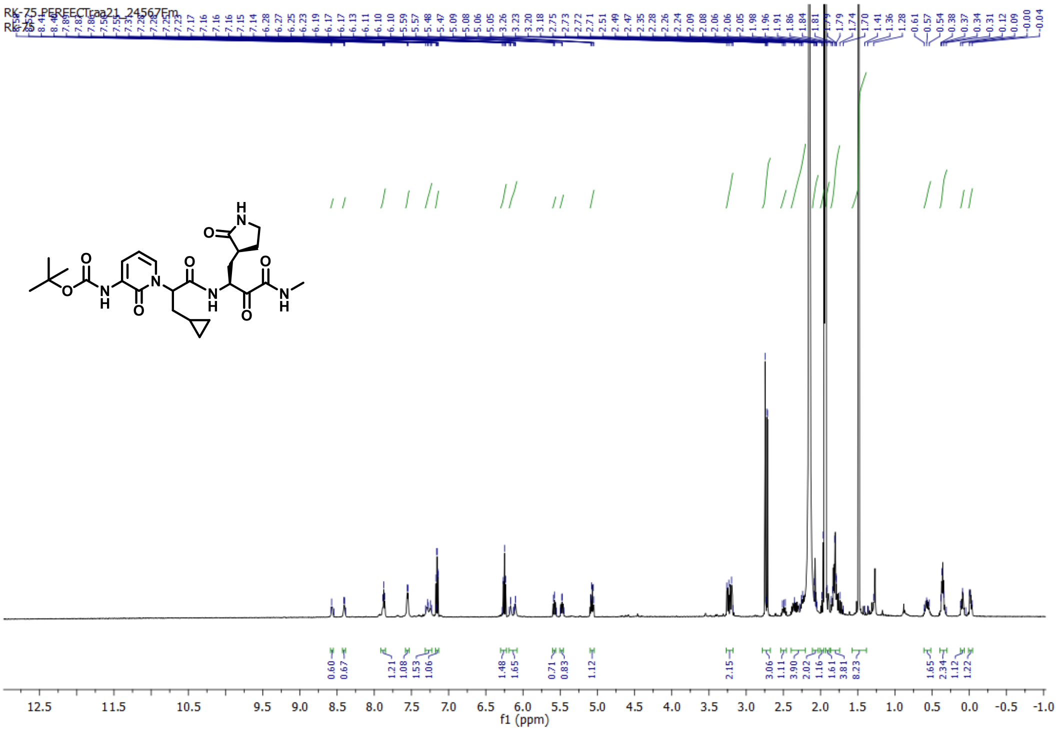

^{13}C-NMR (126 MHz, CD_3CN) of 6a.

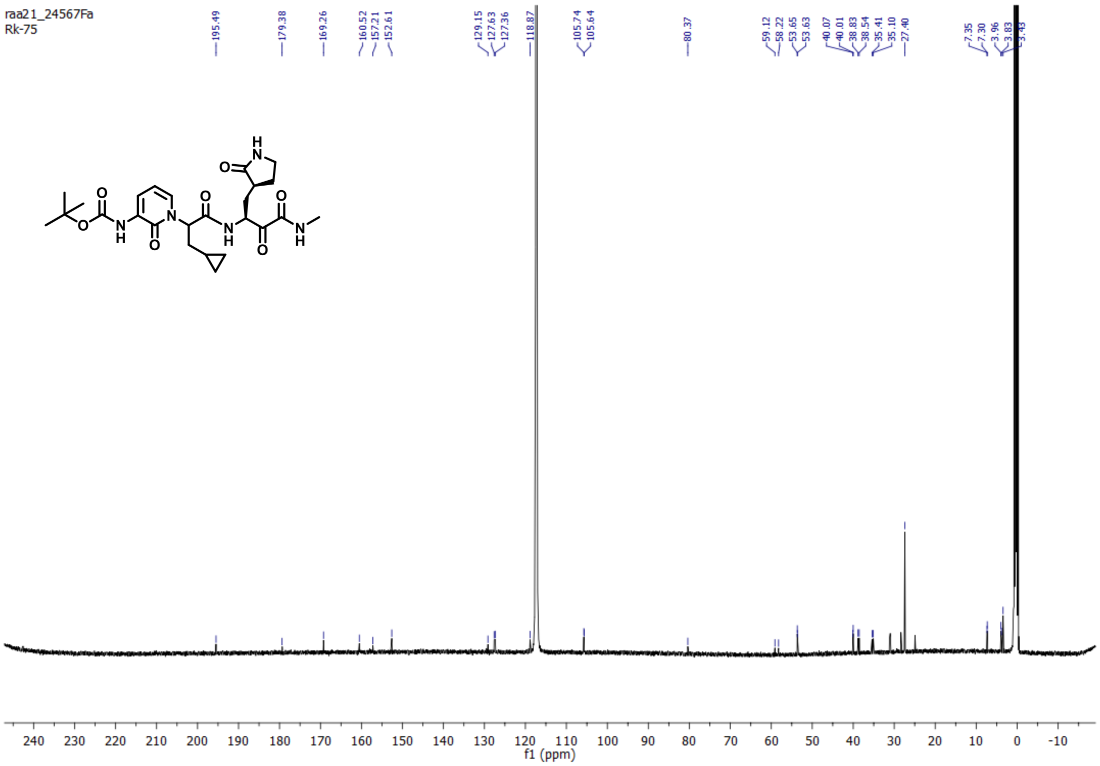

^{1}H-NMR (500 MHz, CD_3CN) of 6b.

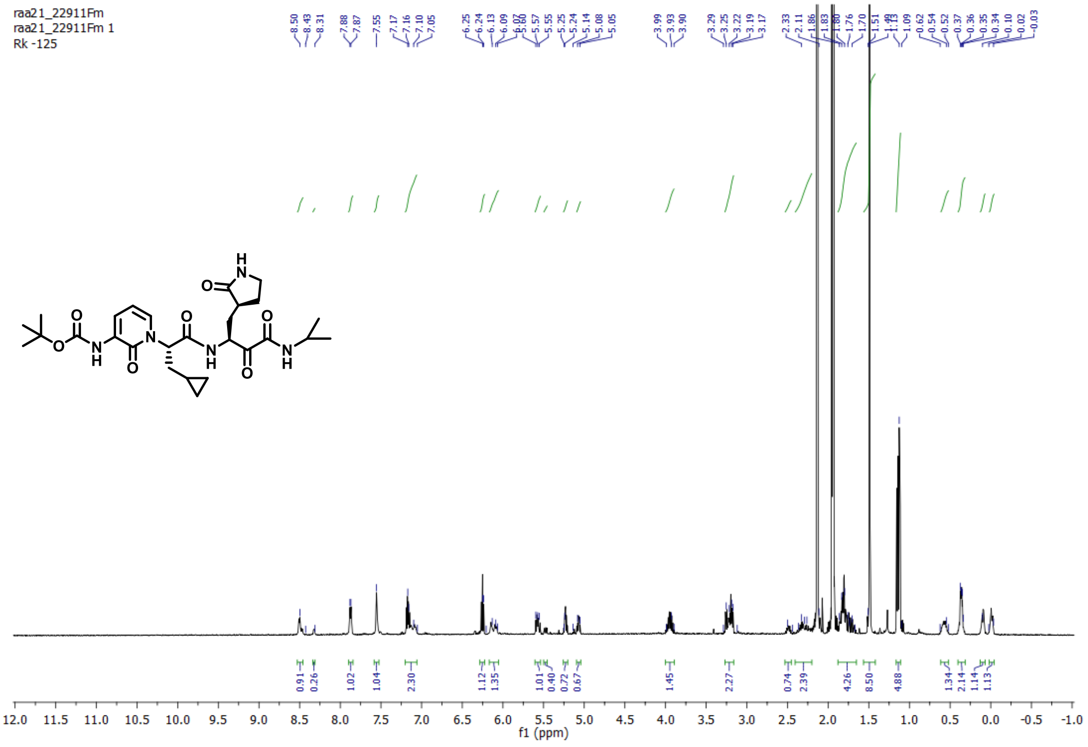

^{13}C-NMR (176 MHz, CD_3CN) of 6b.

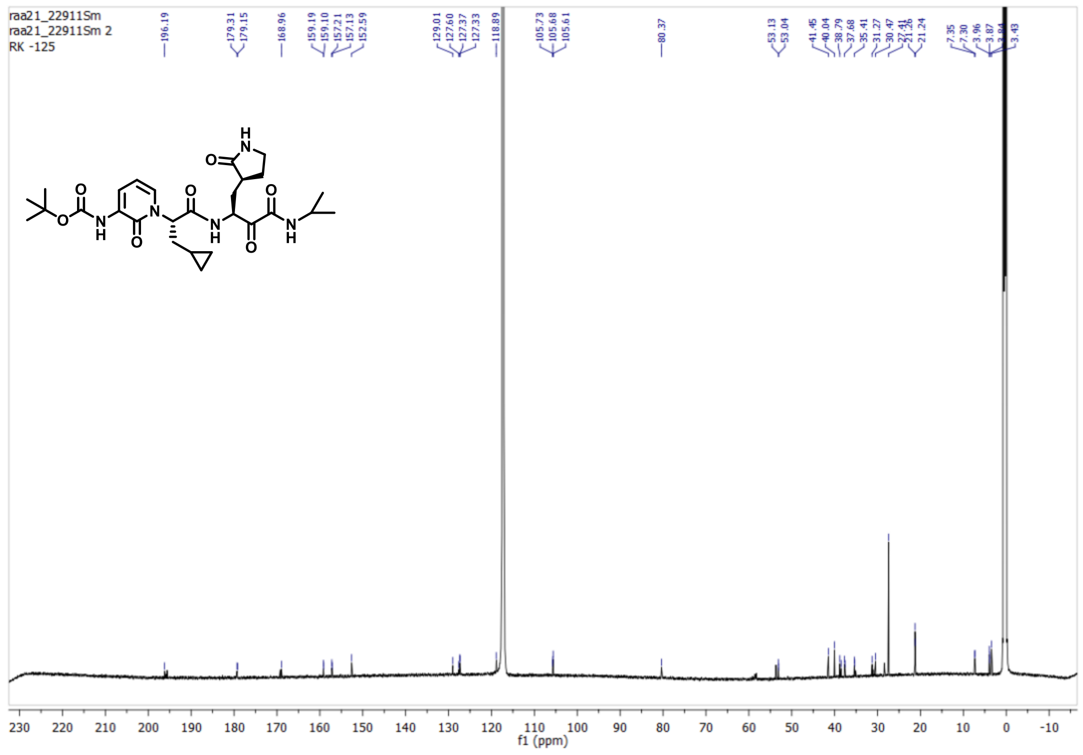

^{1}H-NMR (700 MHz, CD_3CN) of 6c.

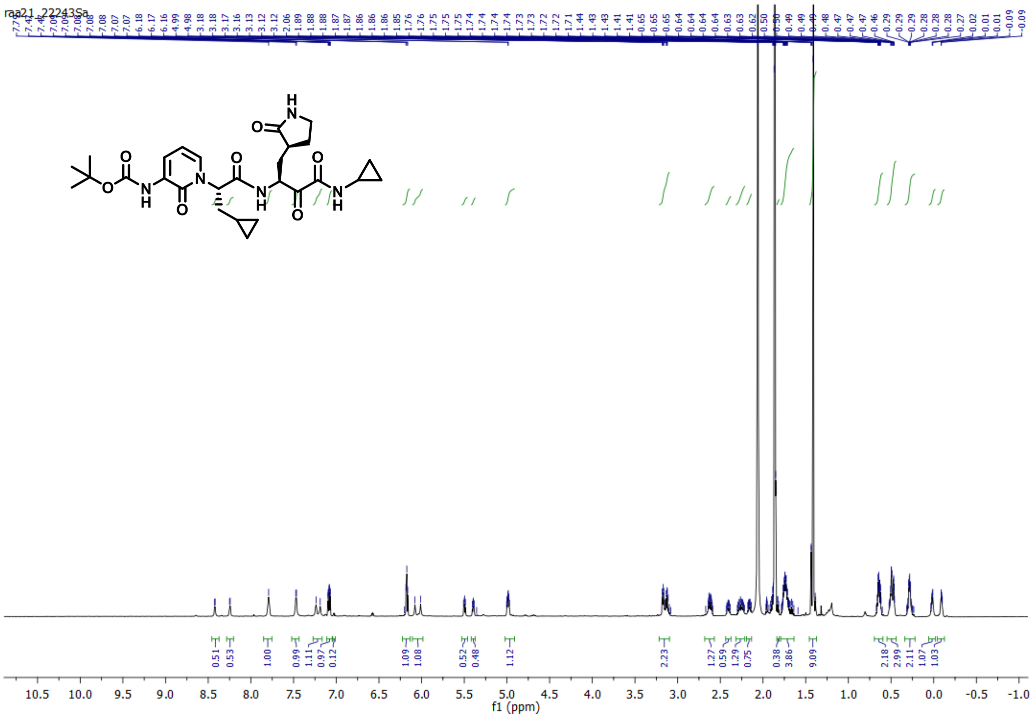

^{13}C-NMR (176 MHz, CD_3CN) of 6c.

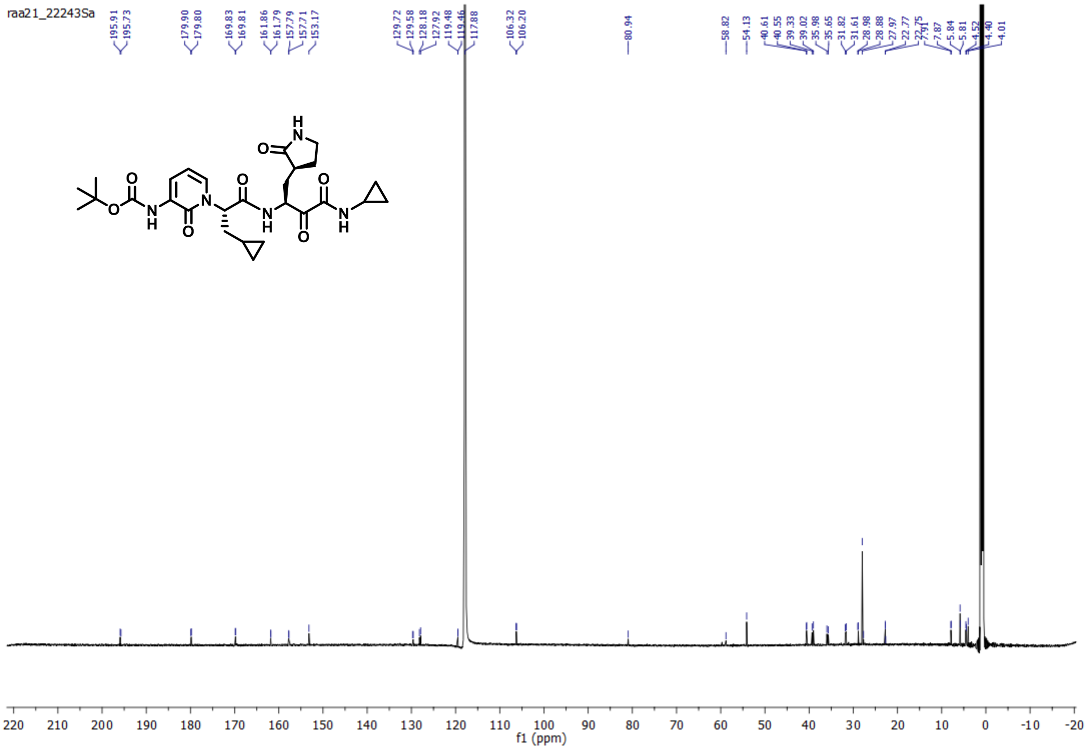

^{1}H-NMR (500 MHz, DMSO-*d*$_6$) of 6d.

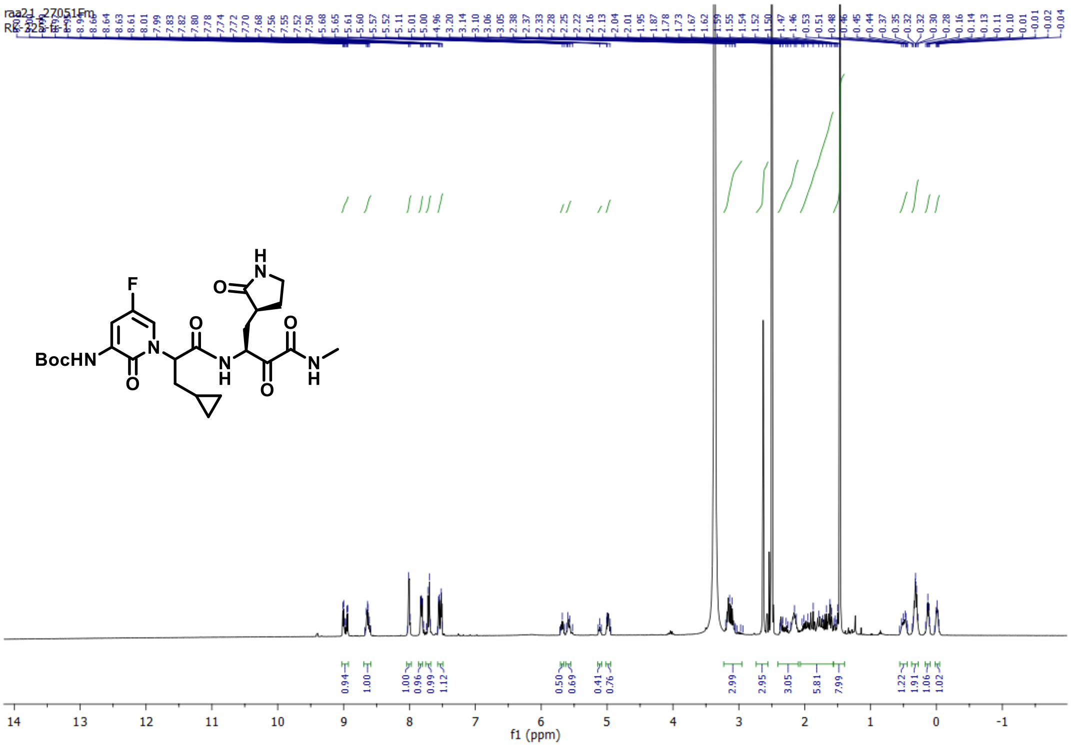

^{13}C-NMR (176 MHz, DMSO-*d*$_6$) of 6d.

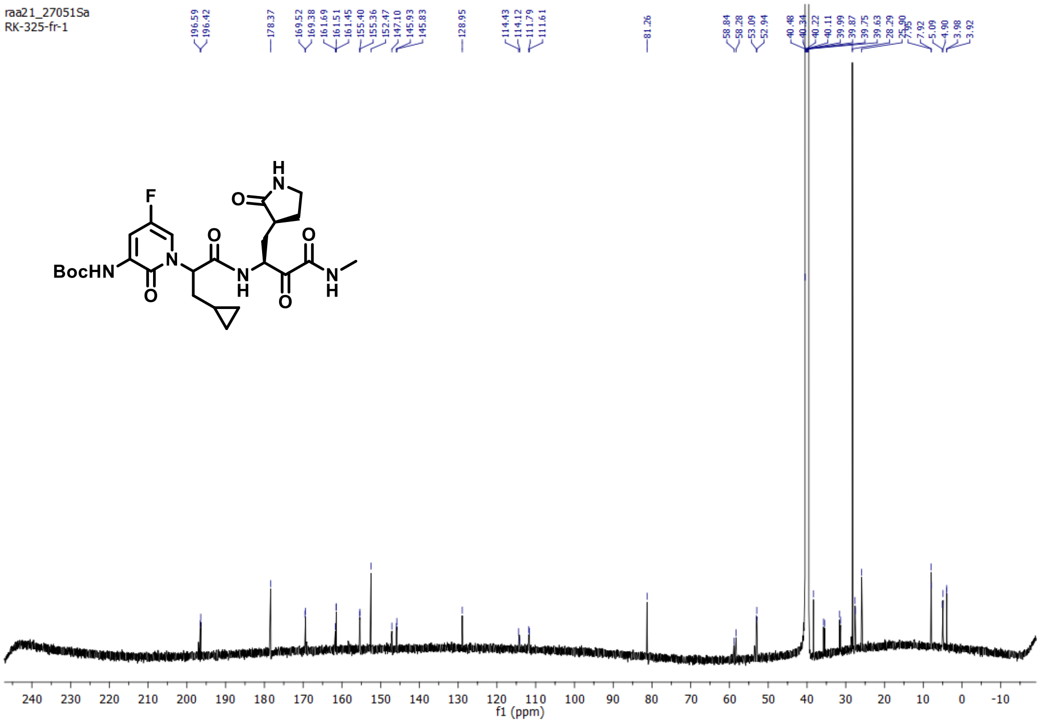

^{1}H-NMR (700 MHz, DMSO-d_6) of 6d-K.

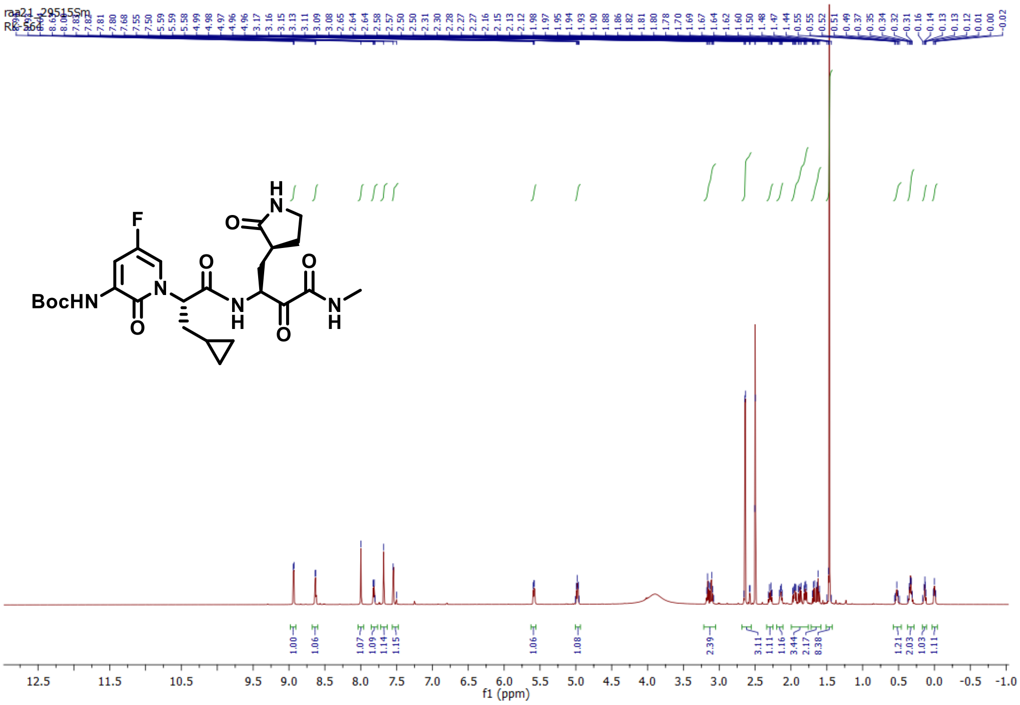

^{13}C-NMR (176 MHz, DMSO-d_6) of 6d-K.

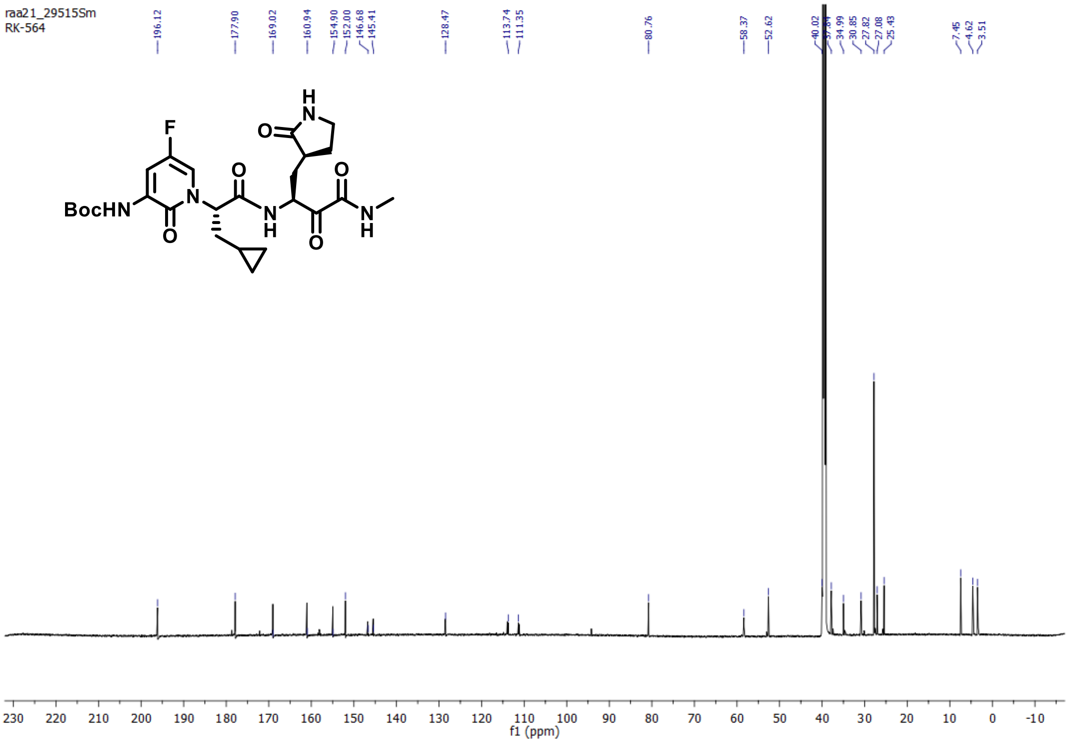

^{1}H-NMR (700 MHz, DMSO-d_6) of 6d-H.

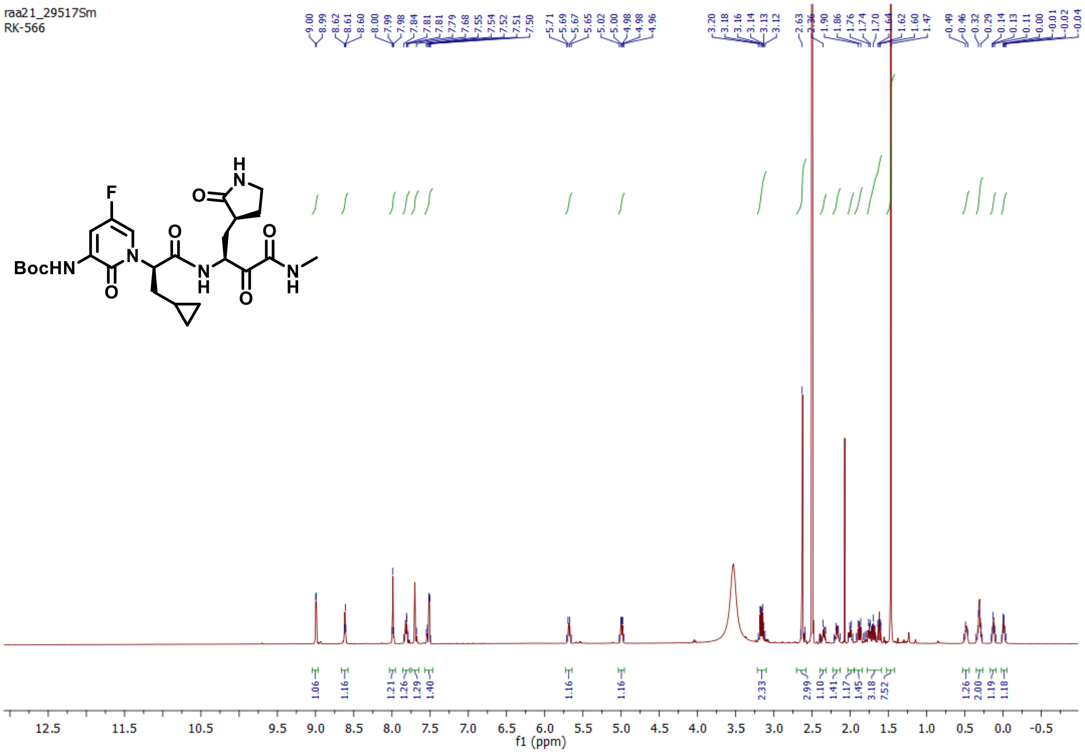

^{13}C-NMR (151 MHz, DMSO-d_6) of 6d-H.

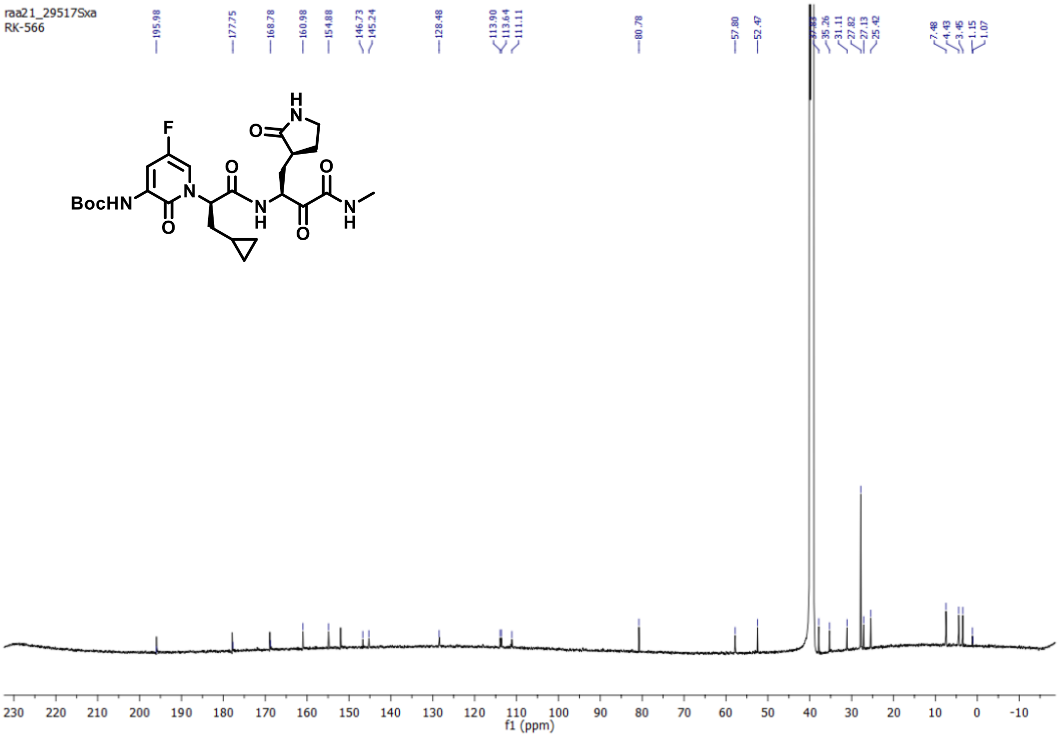

^{1}H-NMR (700 MHz, DMSO-d_6) of 9a.

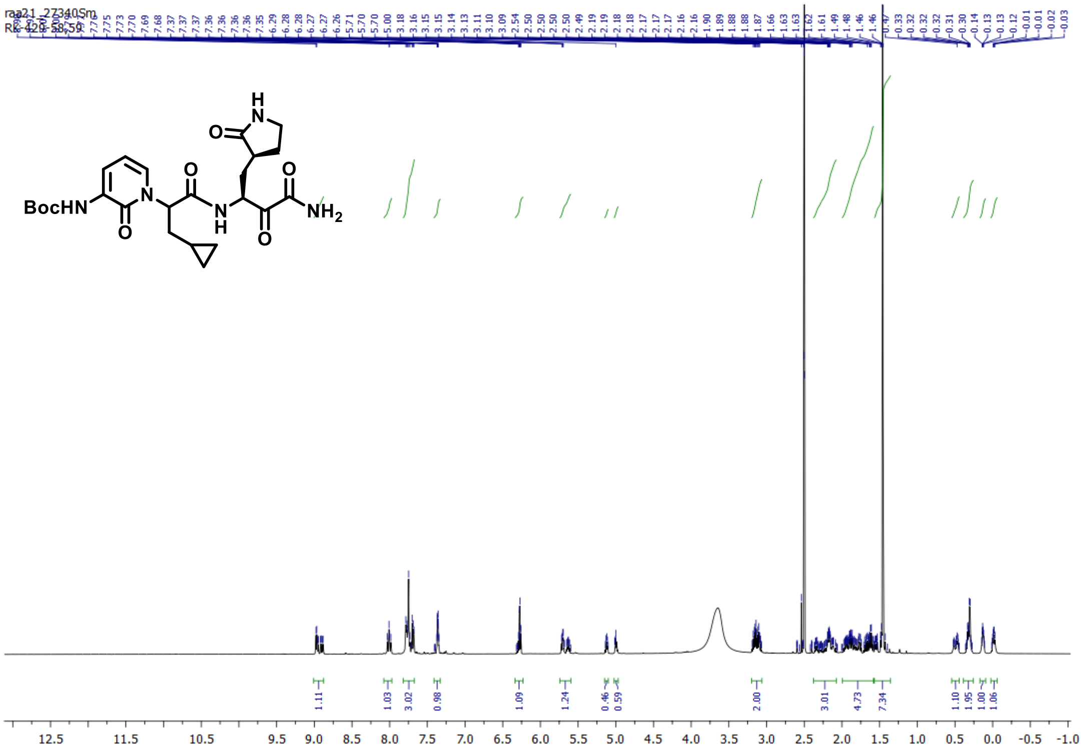

^{13}C-NMR (176 MHz, DMSO-d_6) of 9a.

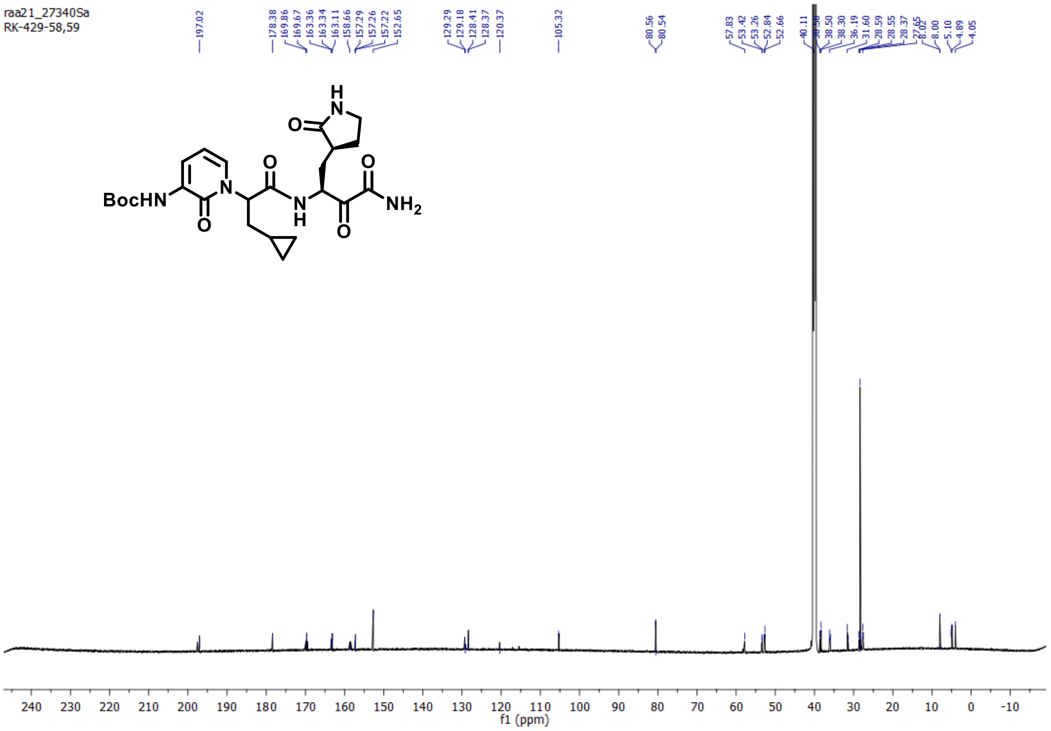

^{1}H-NMR (700 MHz, DMSO-d_6) of 9b.

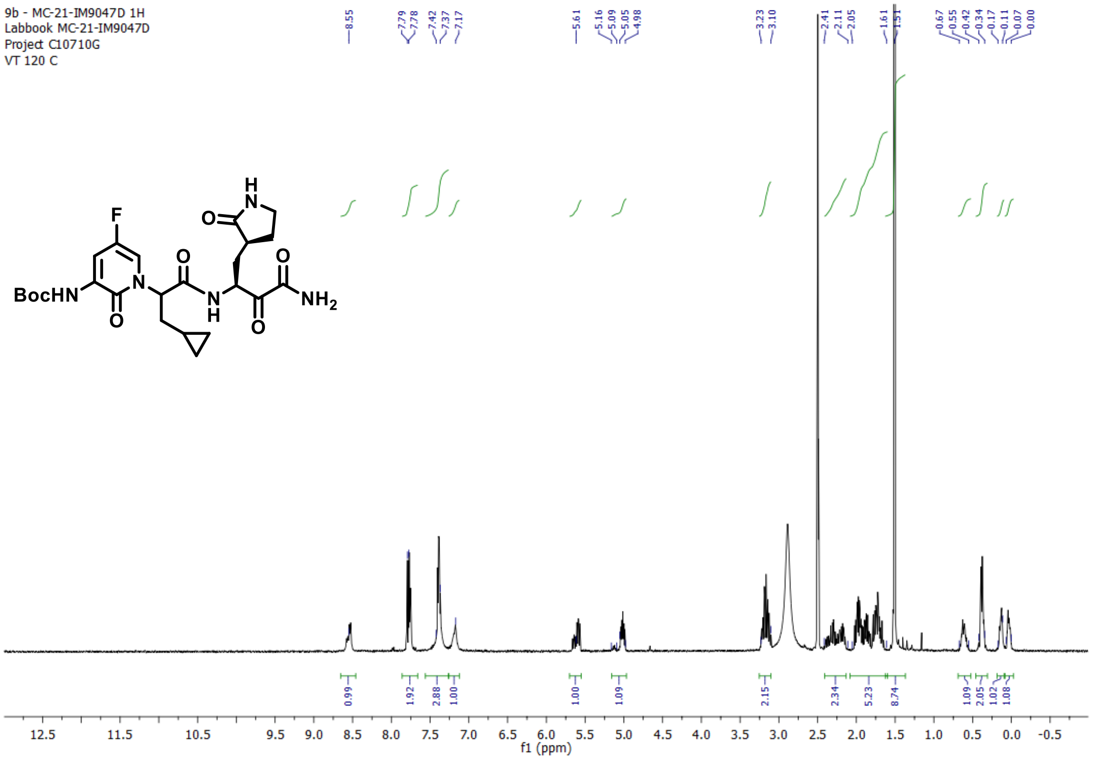

^{13}C-NMR (176 MHz, DMSO-d_6) of 9b.

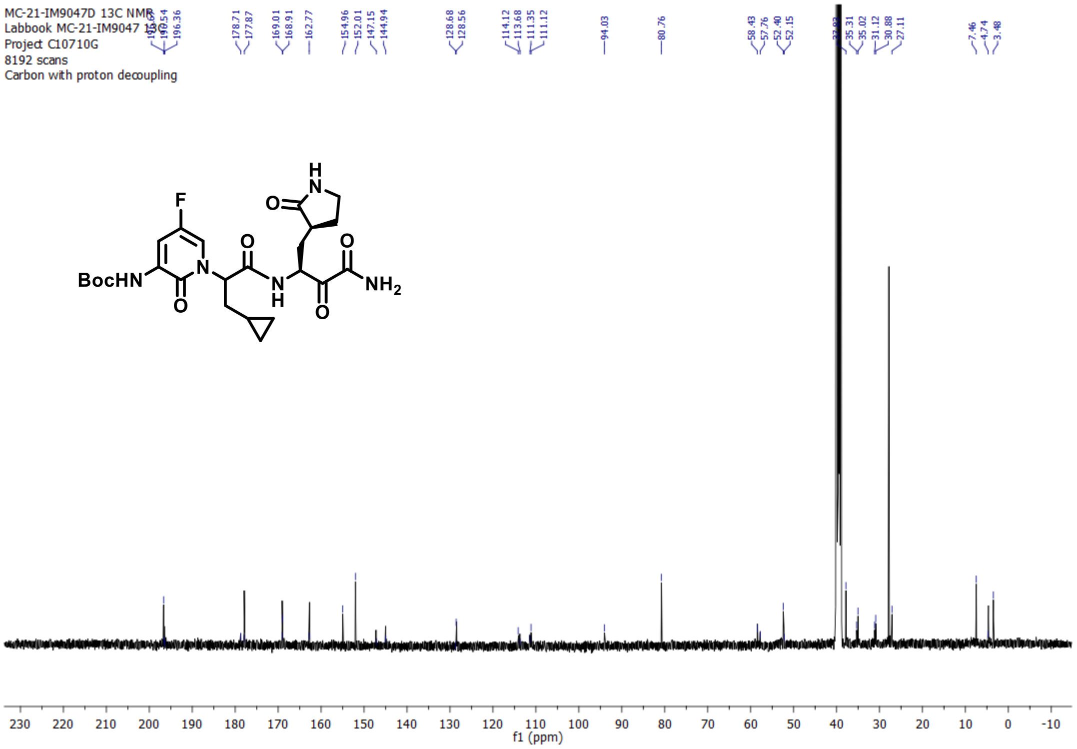

^{1}H-NMR (700 MHz, DMSO-d_6) of 9c.

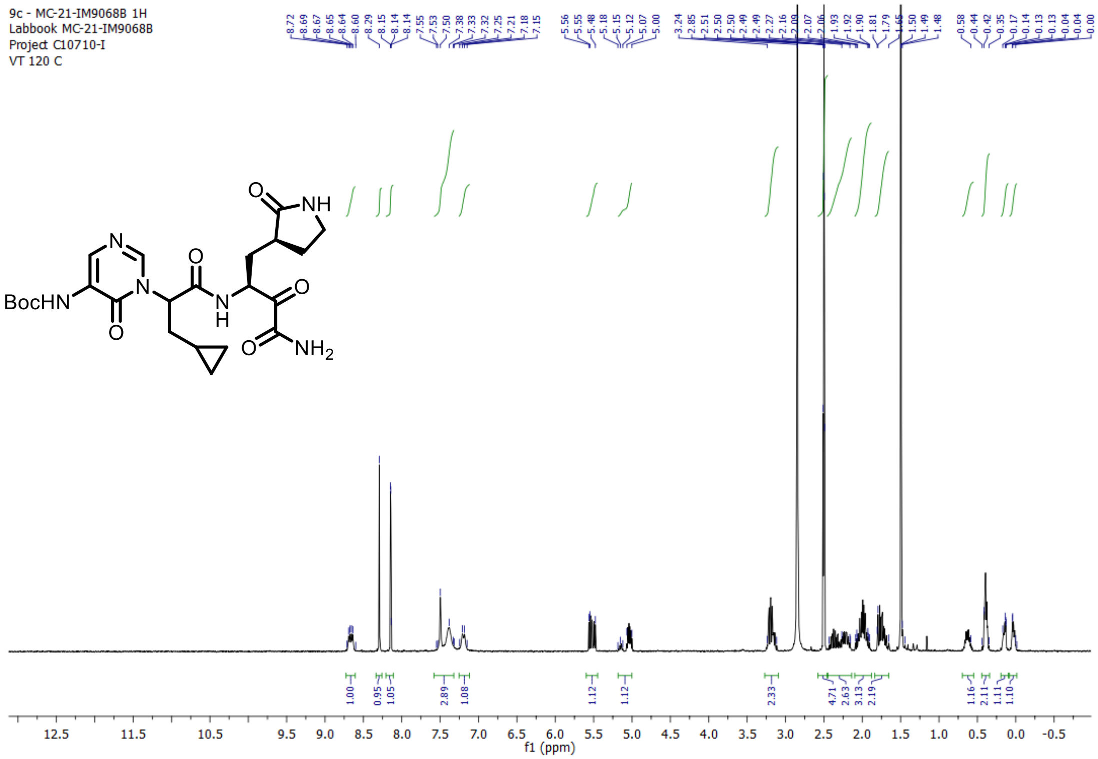

^{13}C-NMR (176 MHz, DMSO-d_6) of 9c.

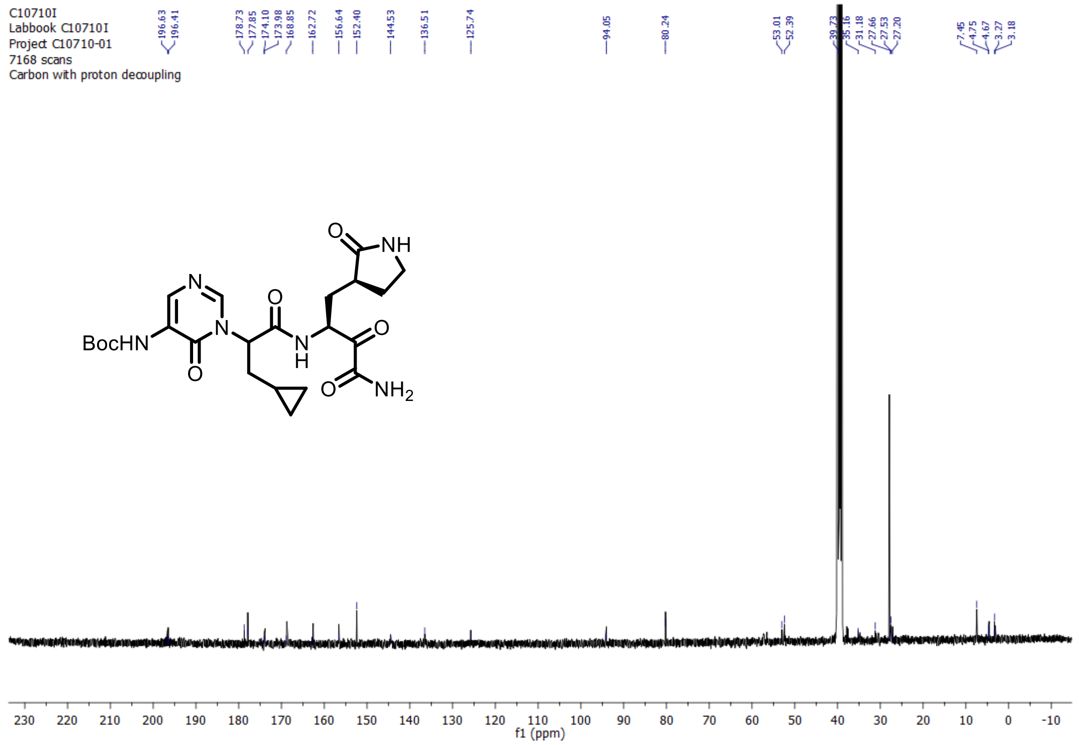

^{1}H-NMR (700 MHz, DMSO-d_6) of 9d.

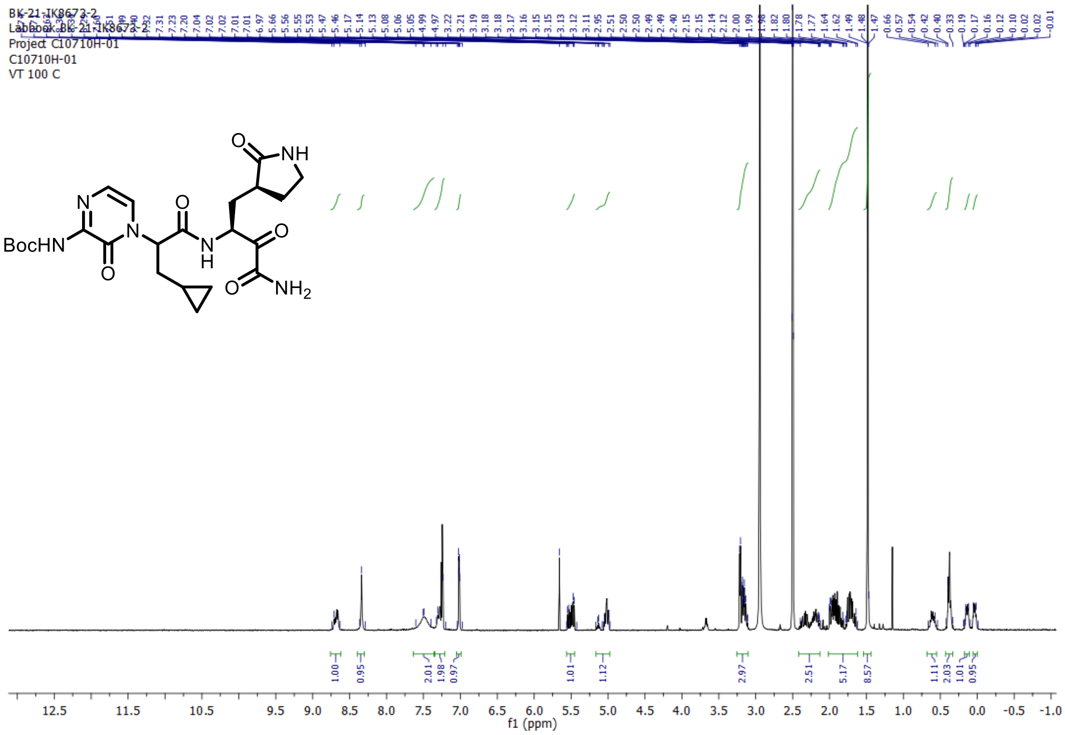

^{13}C-NMR (176 MHz, DMSO-d_6) of 9d.

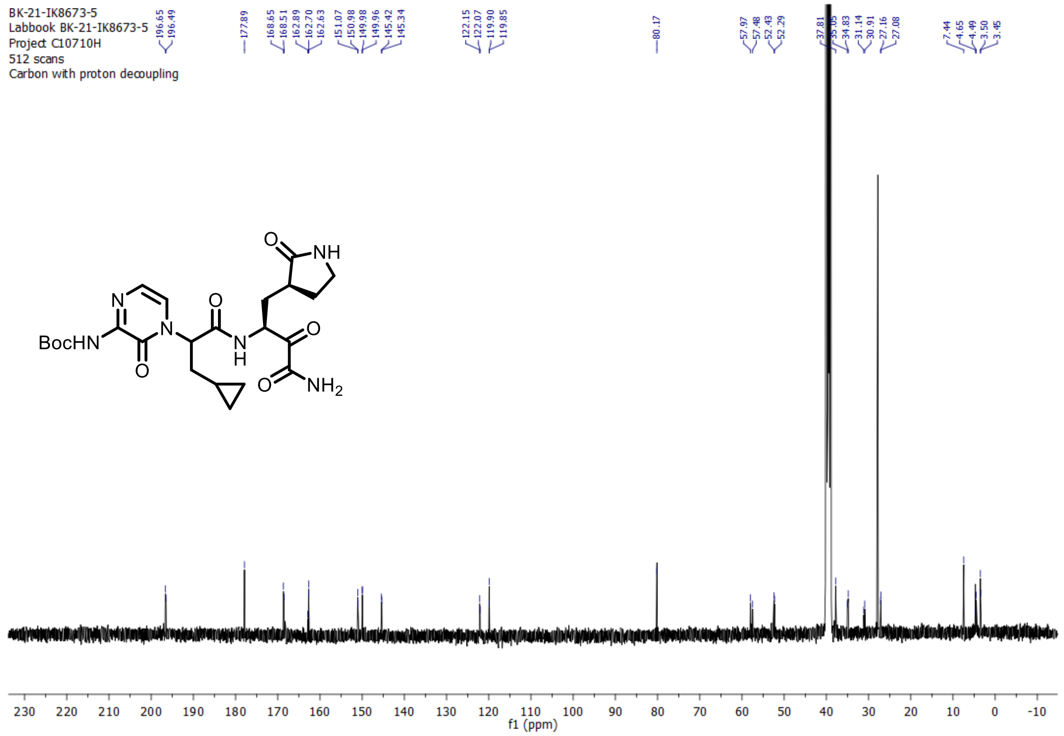

^{1}H-NMR (500 MHz, CD_3CN) of 12a.

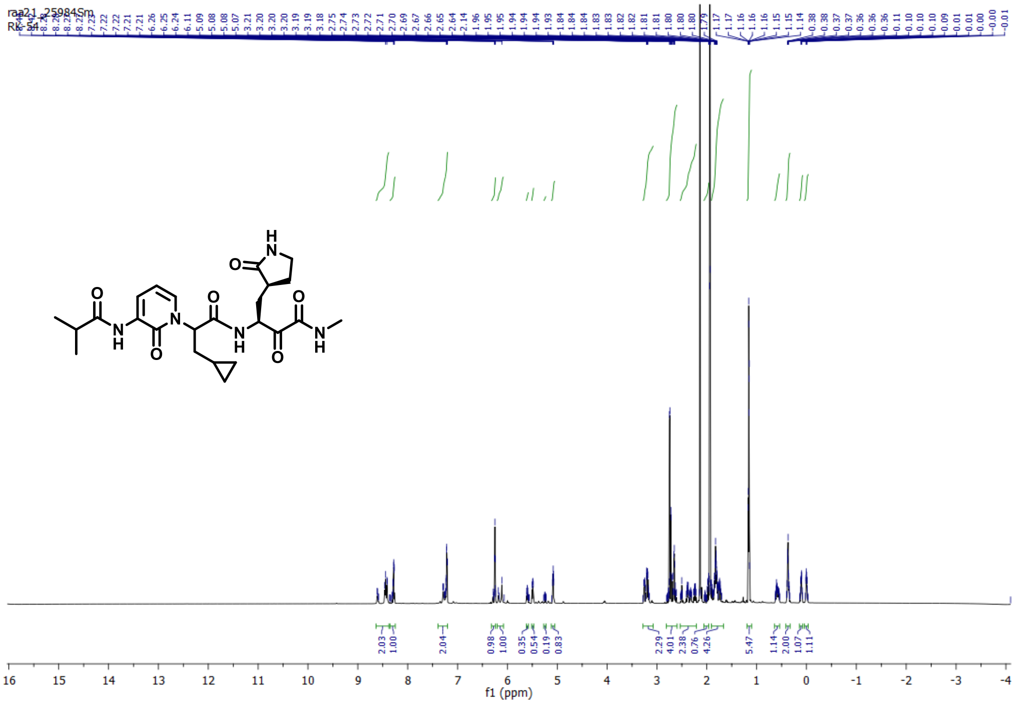

^{13}C-NMR (176 MHz, CD_3CN) of 12a.

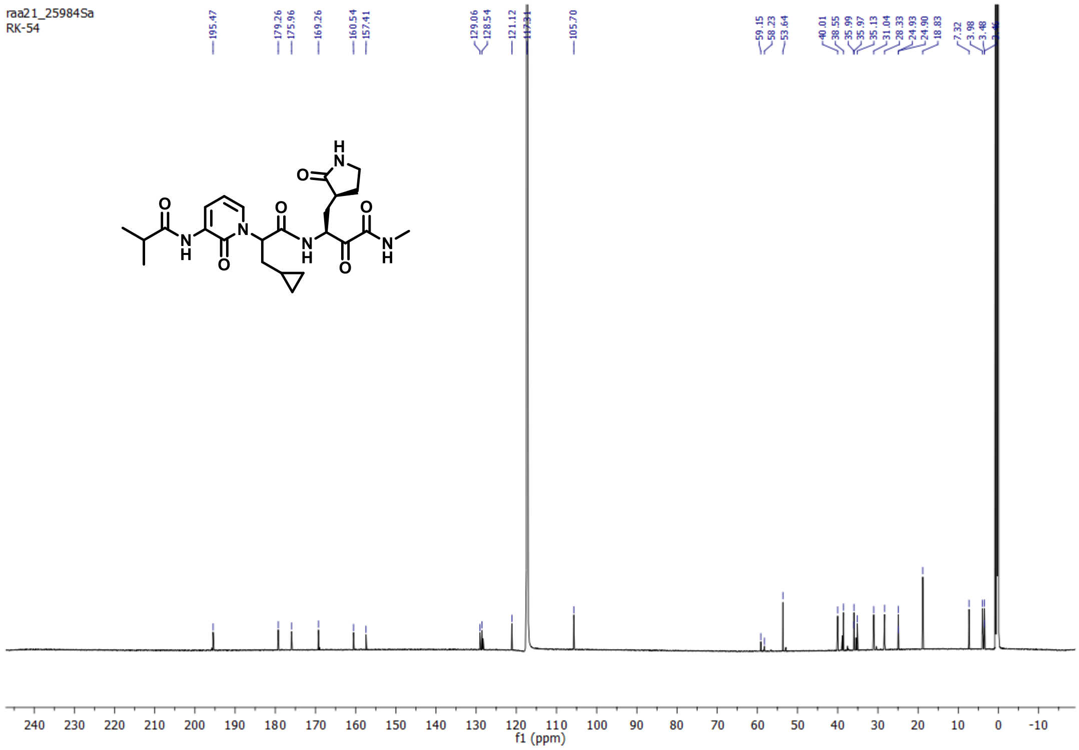

^{1}H-NMR (500 MHz, CD_3CN) of 12b.

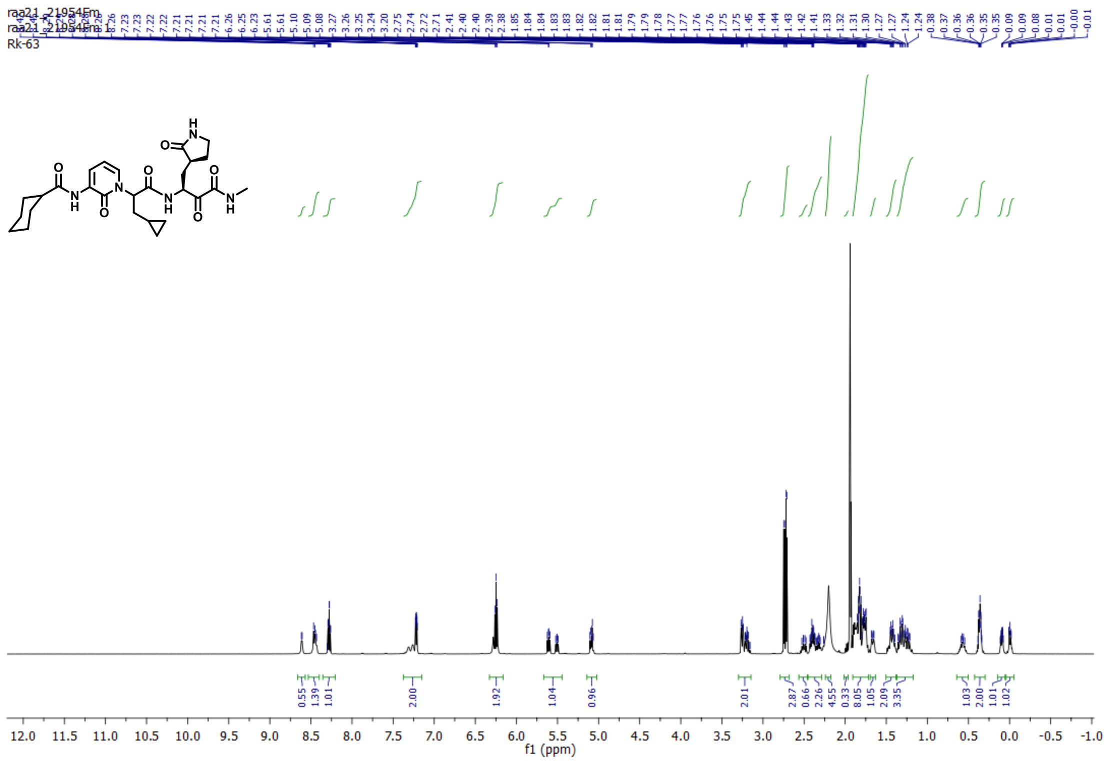

^{13}C-NMR (176 MHz, CD_3CN) of 12b.

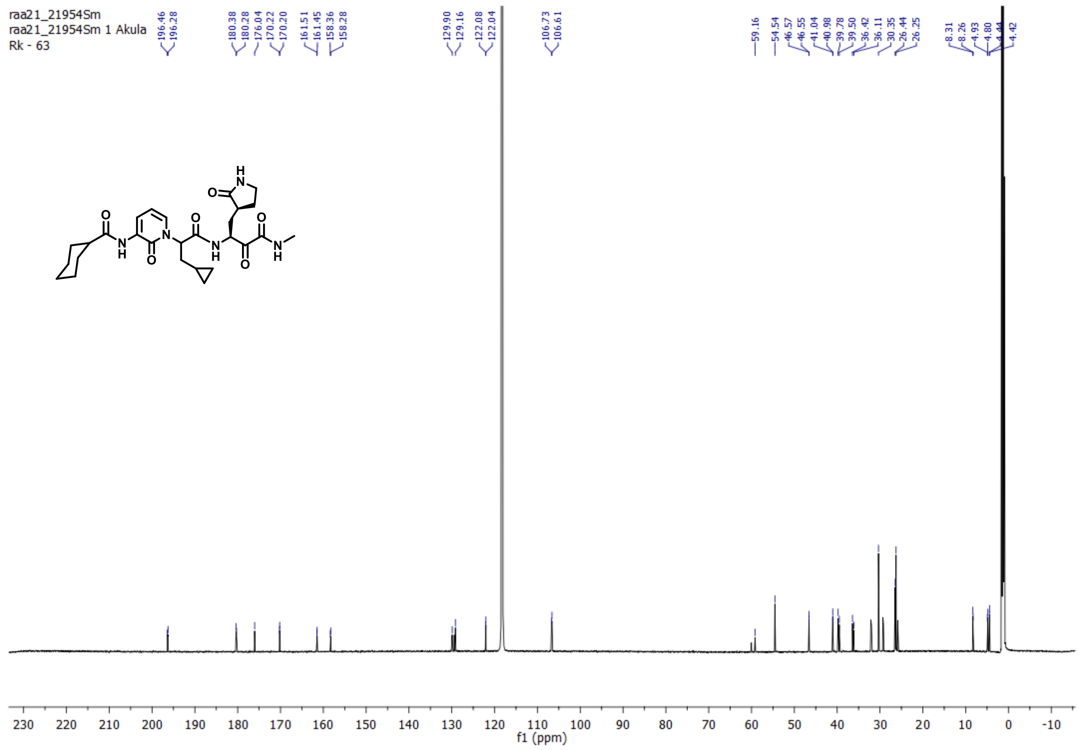

^{1}H-NMR (500 MHz, CD_3CN) of 12c.

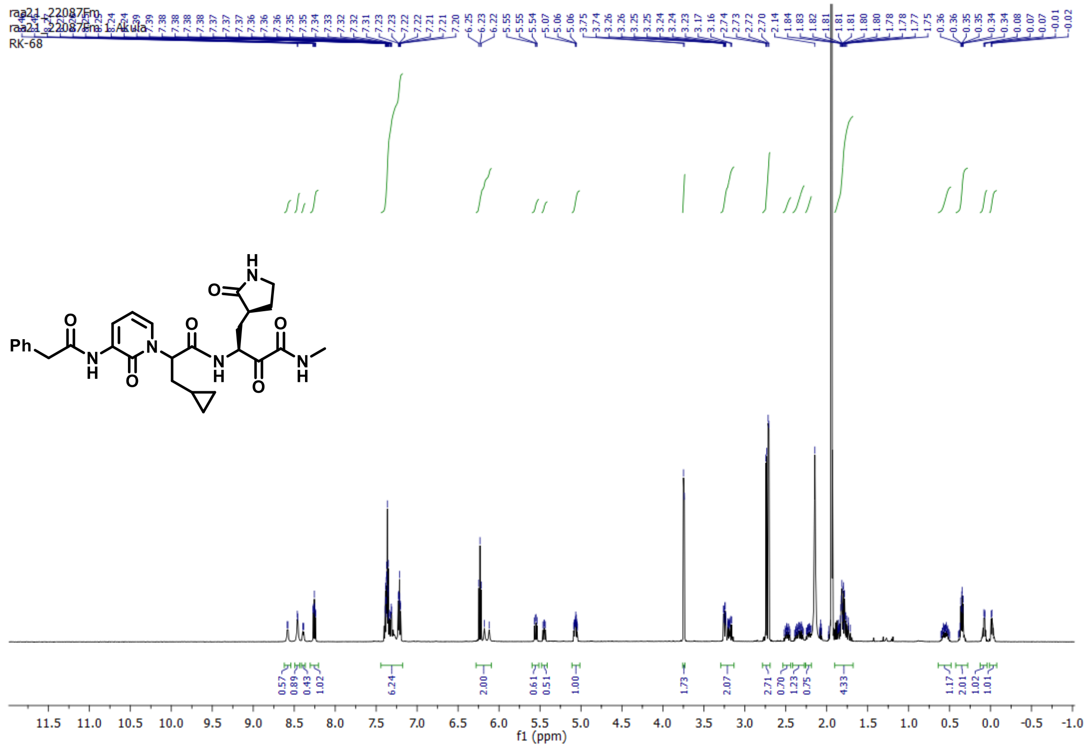

^{13}C-NMR (176 MHz, CD3CN) of 12c.

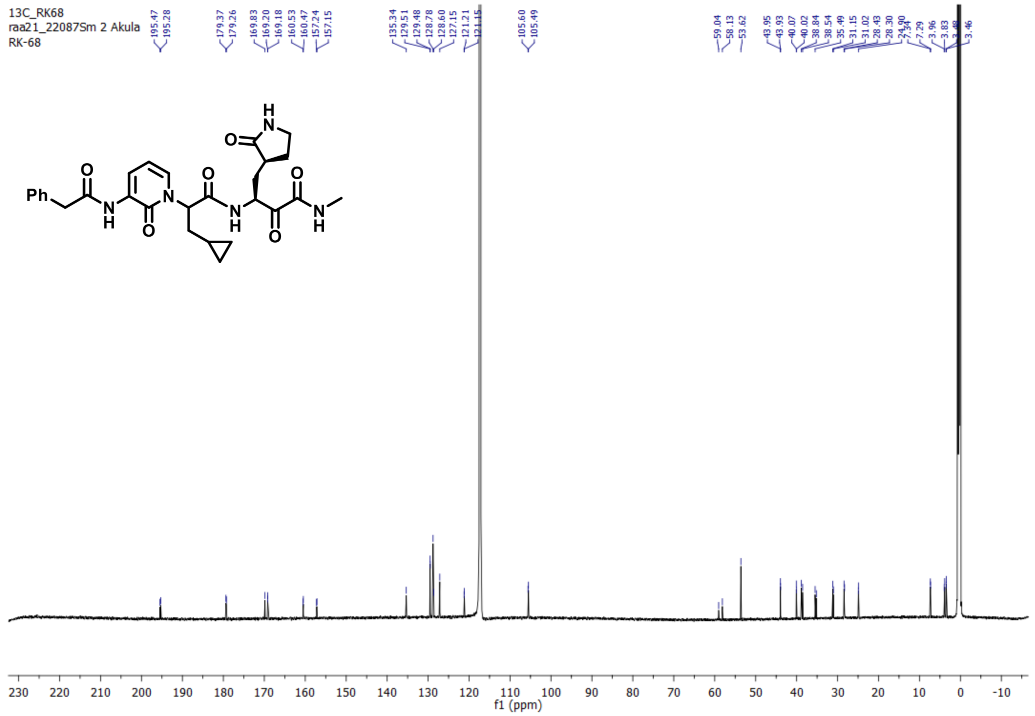

^{1}H-NMR (500 MHz, CD_3CN) of 12d.

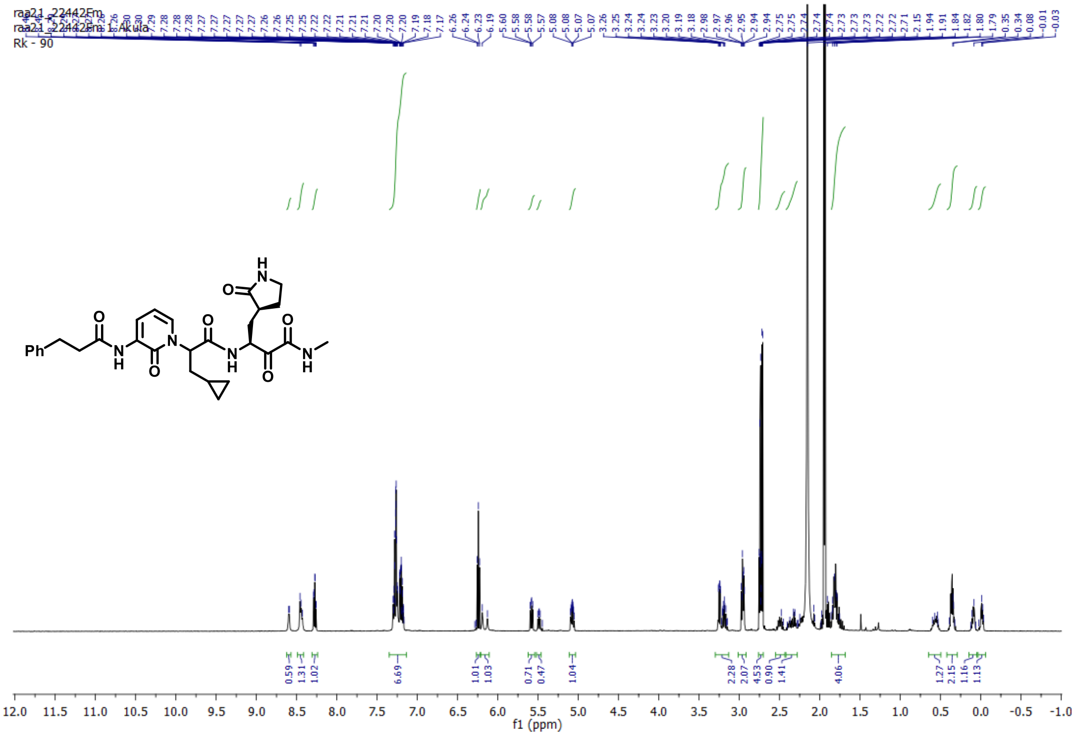

^{13}C-NMR (176 MHz, CD_3CN) of 12d.

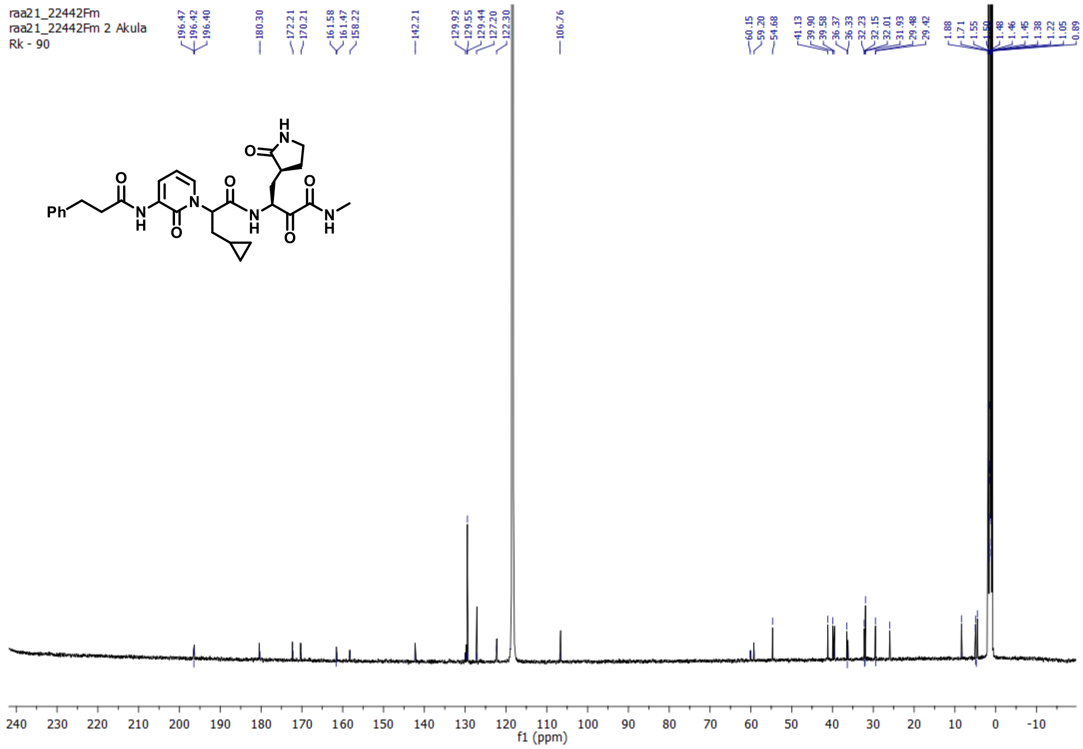

^{1}H-NMR (500 MHz, CD_3CN) of 12e.

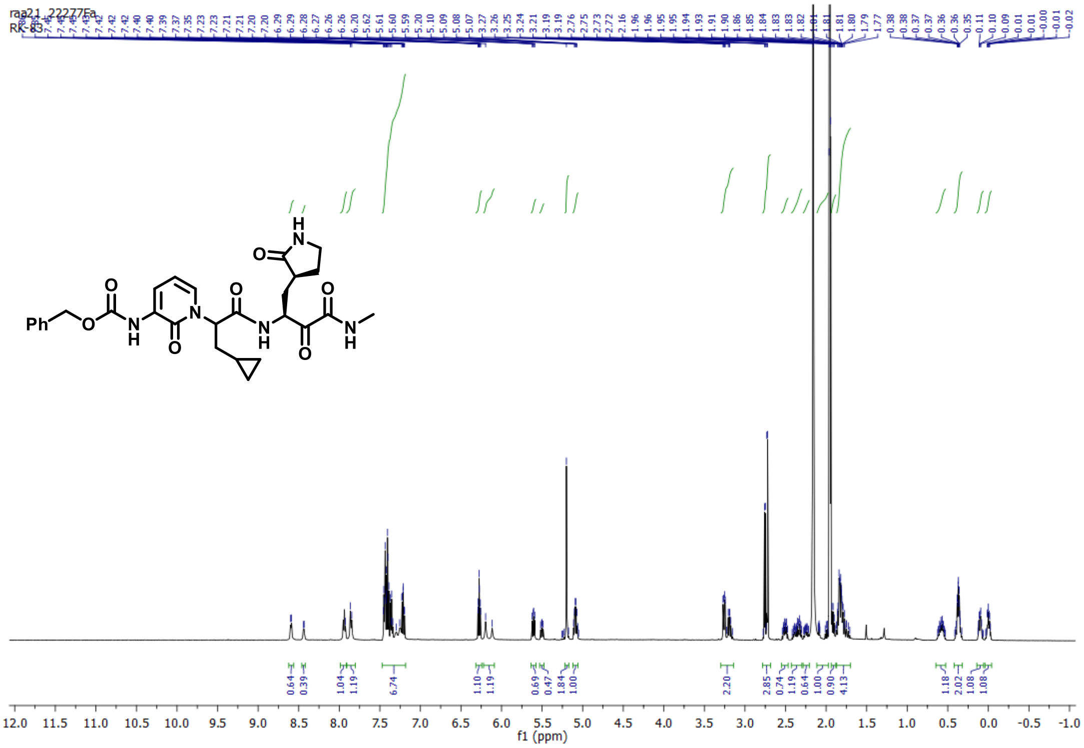

^{13}C-NMR (176 MHz, CD_3CN) of 12e.

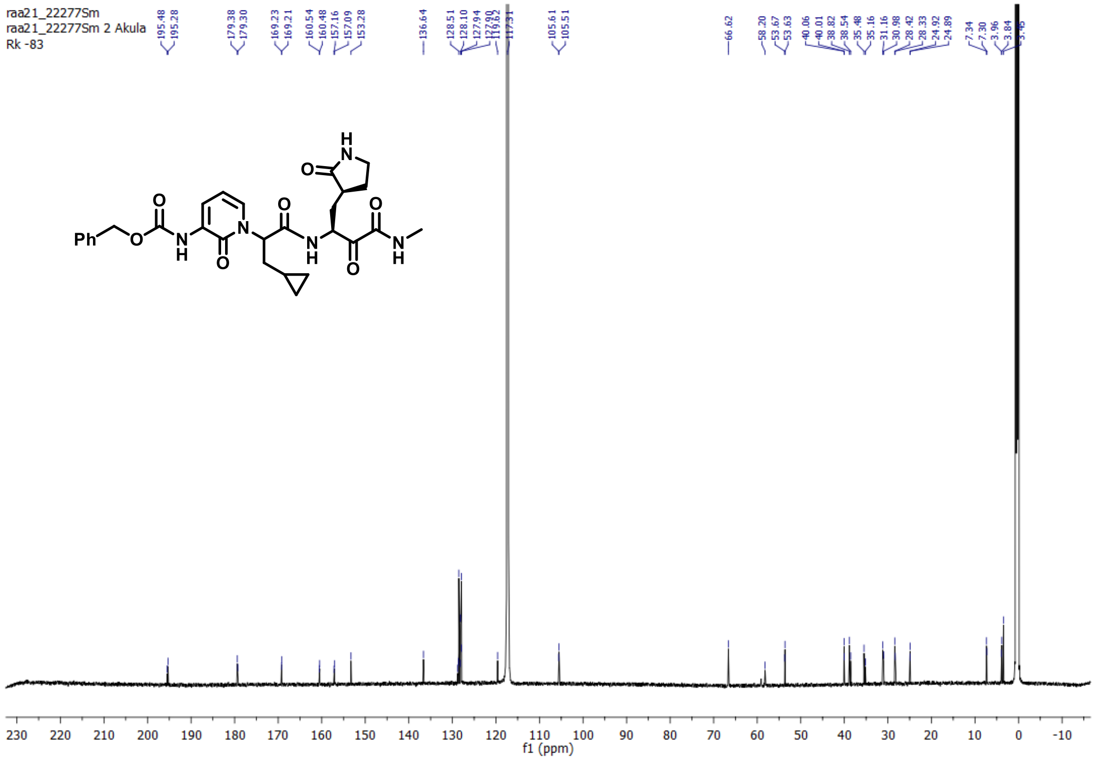

^{1}H-NMR (500 MHz, CD_3CN) of 12f.

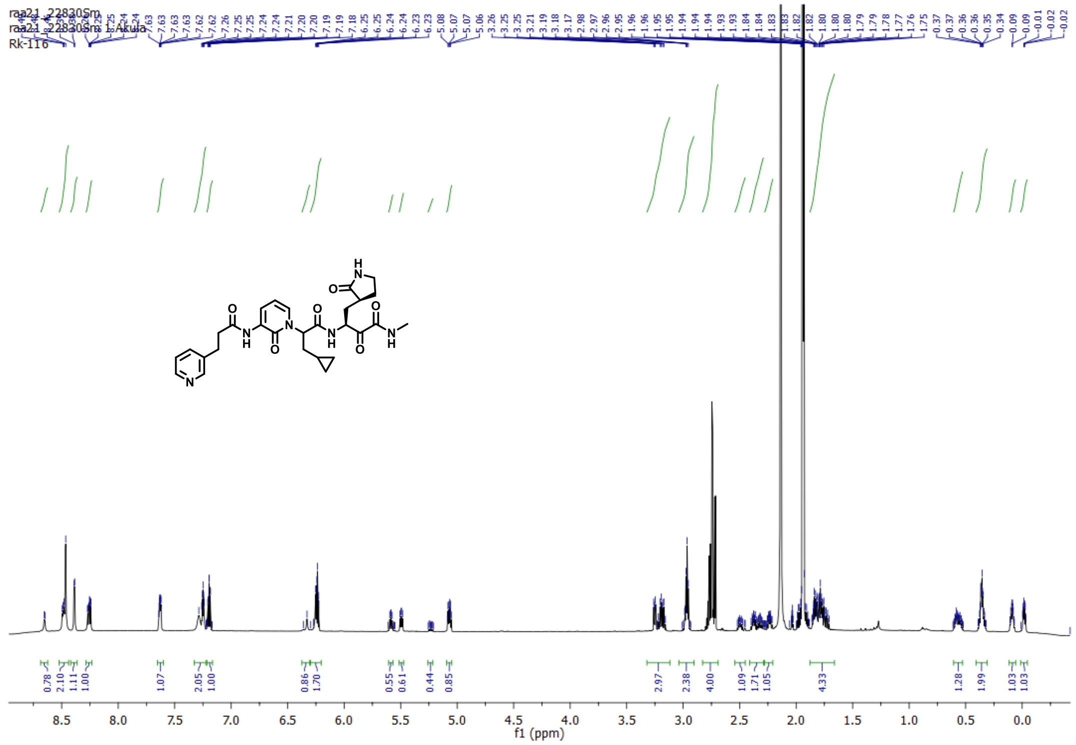

^{13}C-NMR (176 MHz, CD_3CN) of 12f.

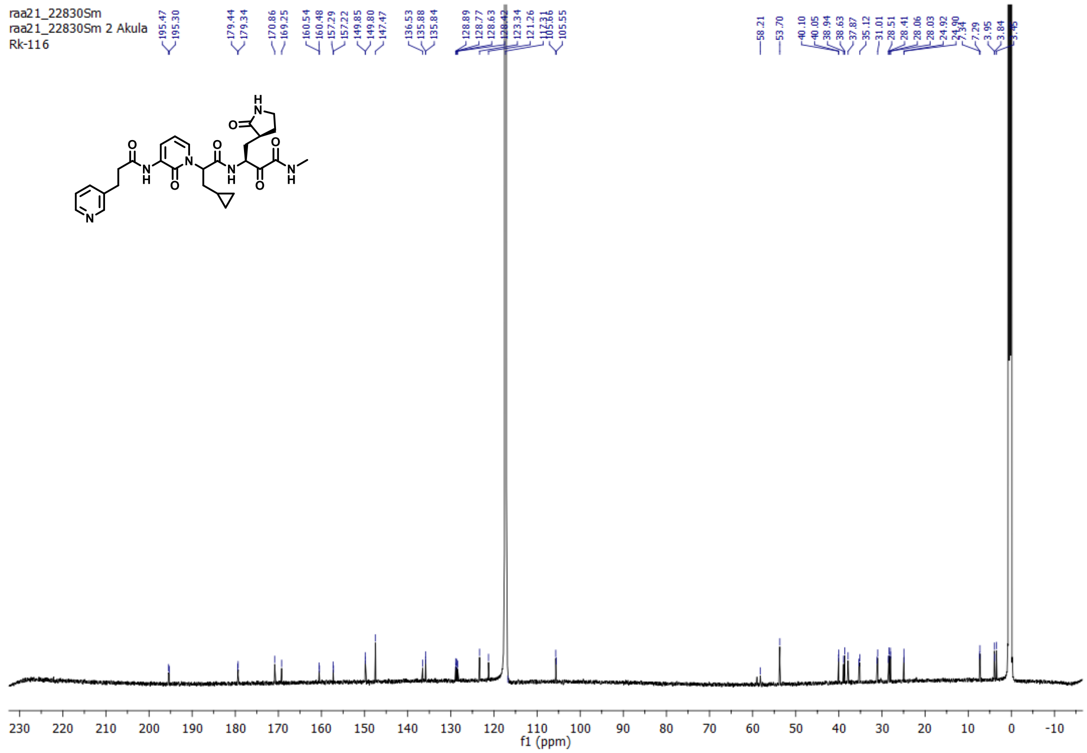

^{1}H-NMR (500 MHz, CD_3CN) of 12g.

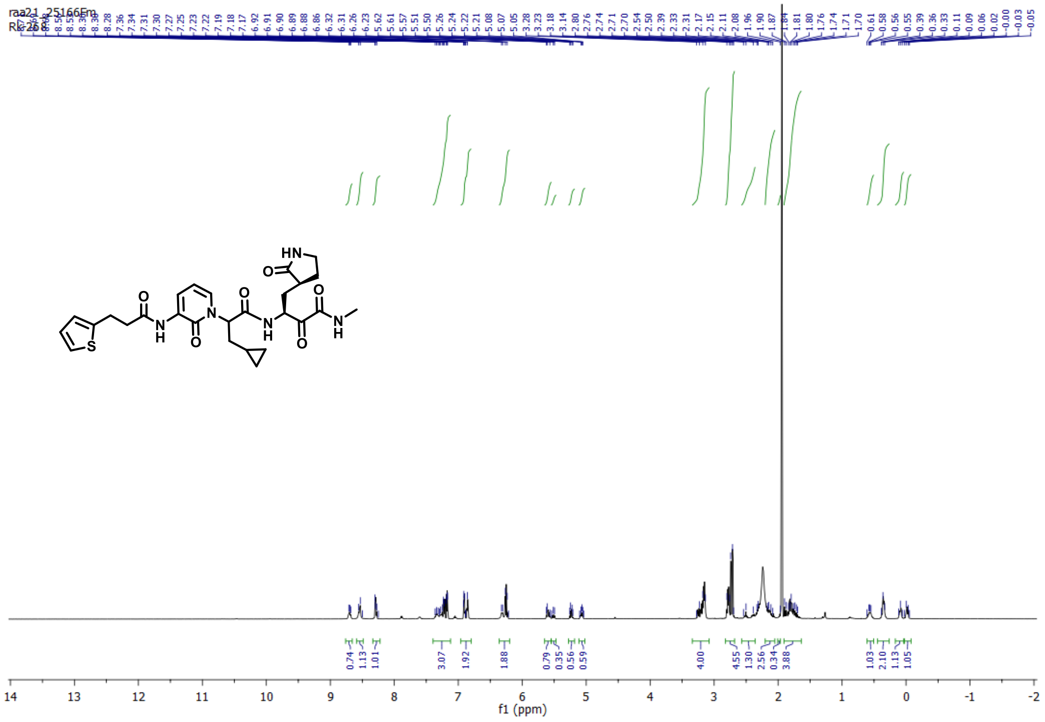

^{13}C-NMR (176 MHz, CD_3CN) of 12g.

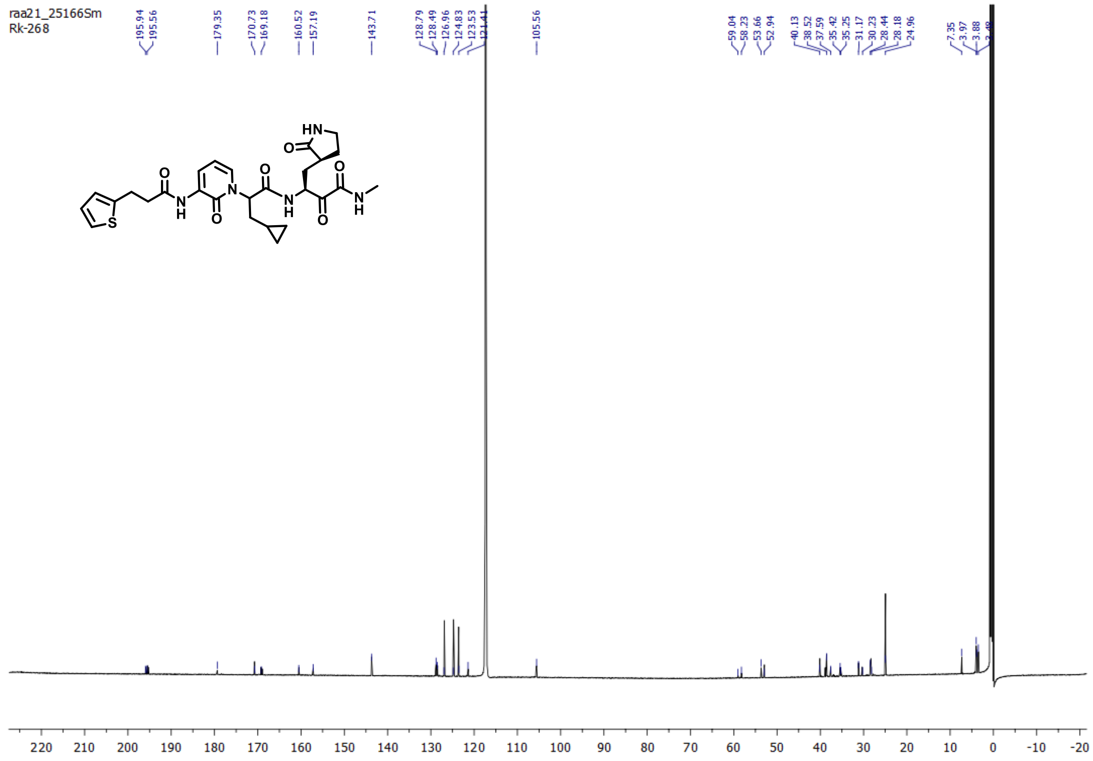

^{1}H-NMR (500 MHz, DMSO-d_6) of 12h.

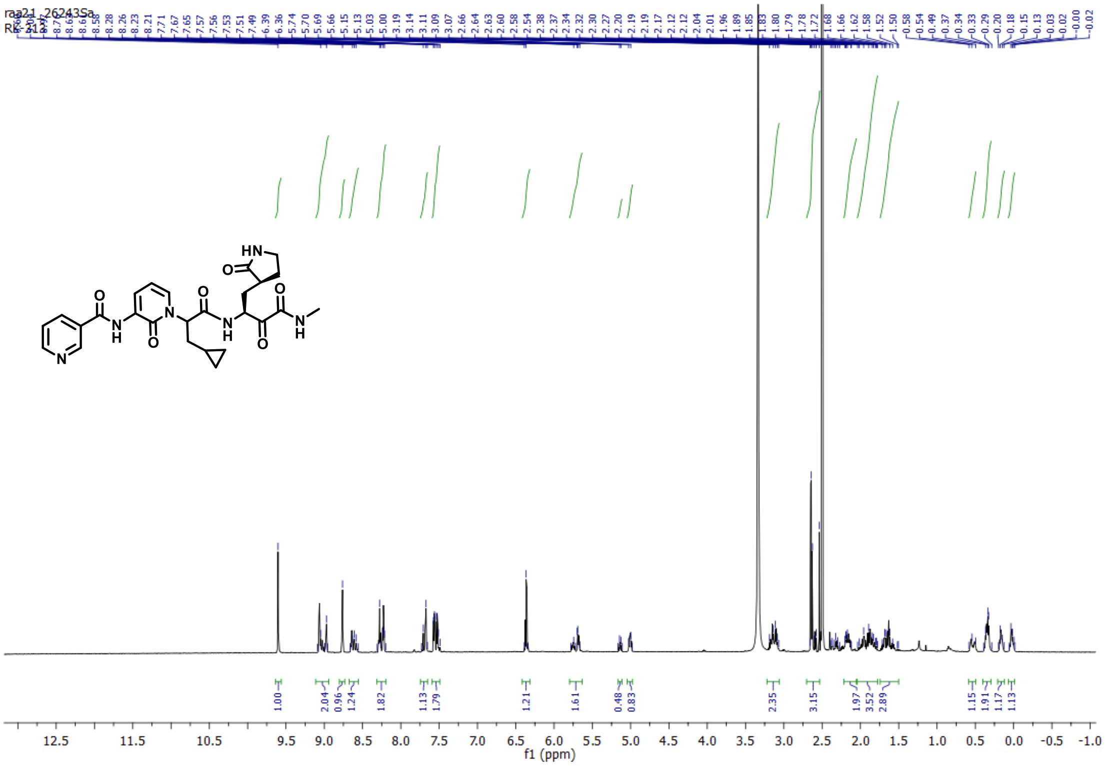

^{13}C-NMR (125 MHz, DMSO-d_6) of 12h.

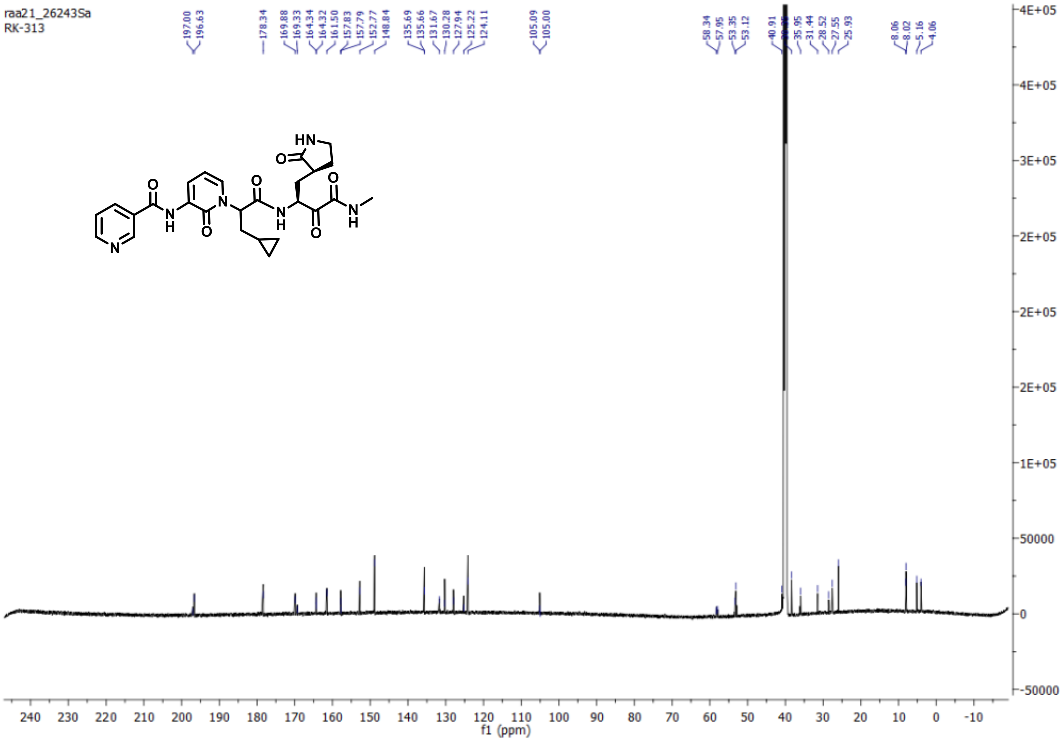

^{1}H-NMR (500 MHz, CD_3CN) of 16a.

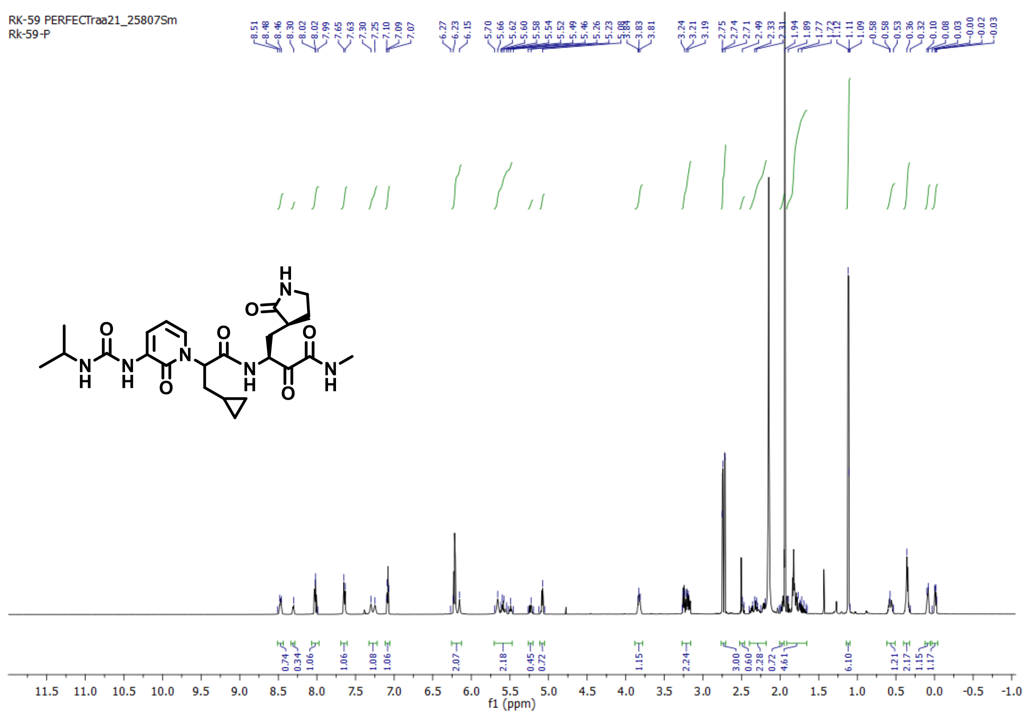

^{13}C-NMR (125 MHz, CD_3CN) of 16a.

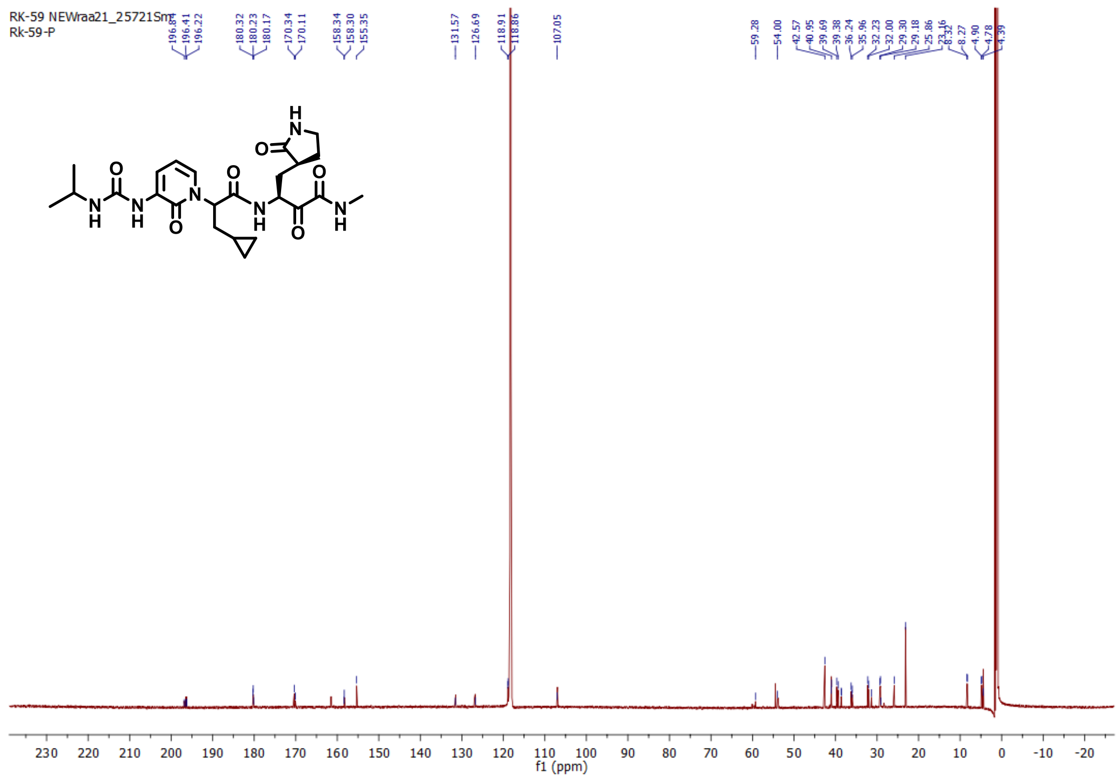

^{1}H-NMR (500 MHz, CD_3CN) of 16b.

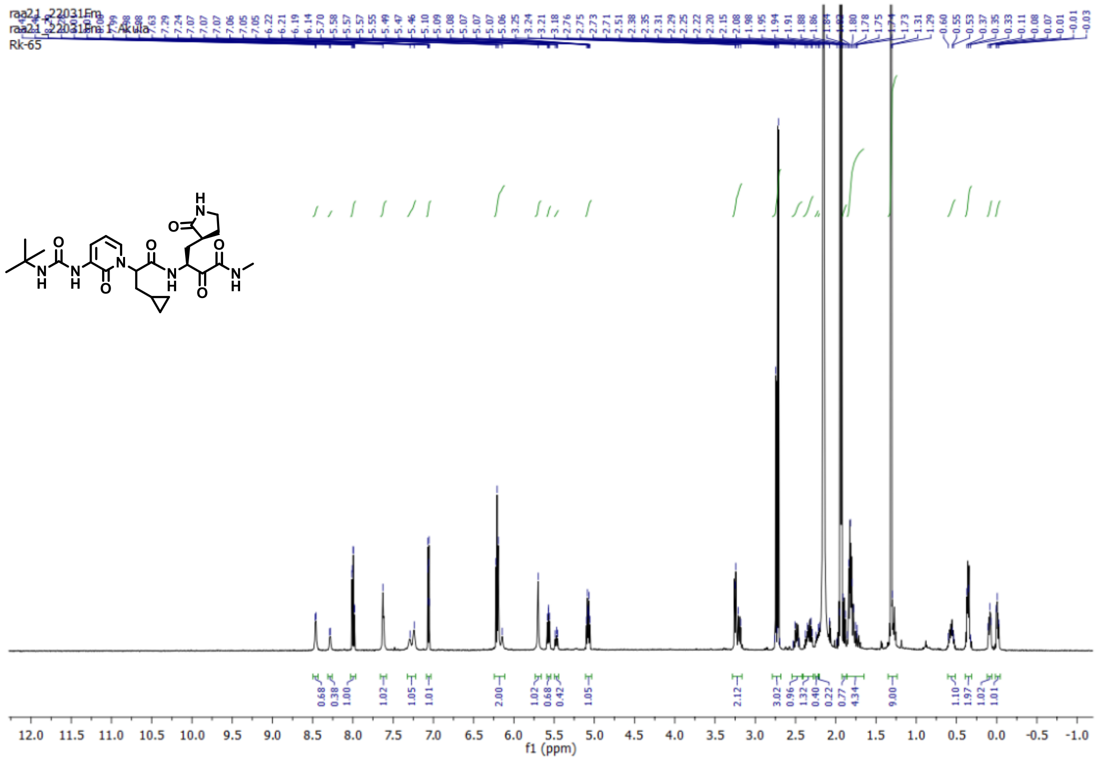

^{13}C-NMR (125 MHz, CD_3CN) of 16b.

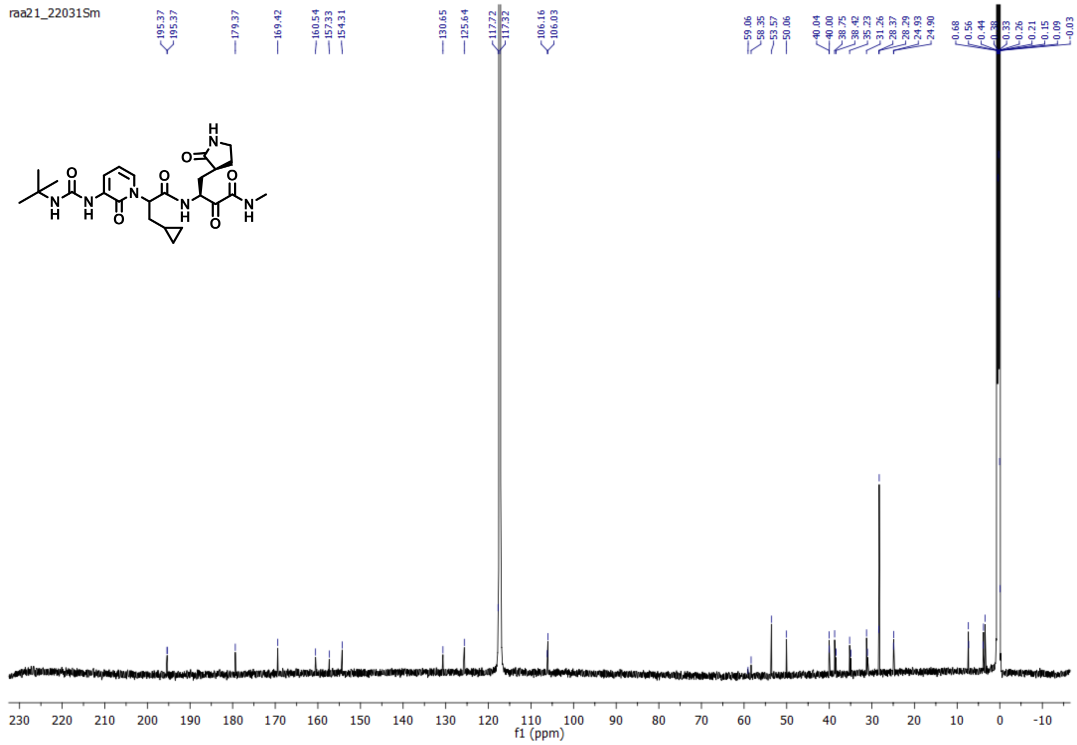

^{1}H-NMR (500 MHz, CD_3CN) of 16c.

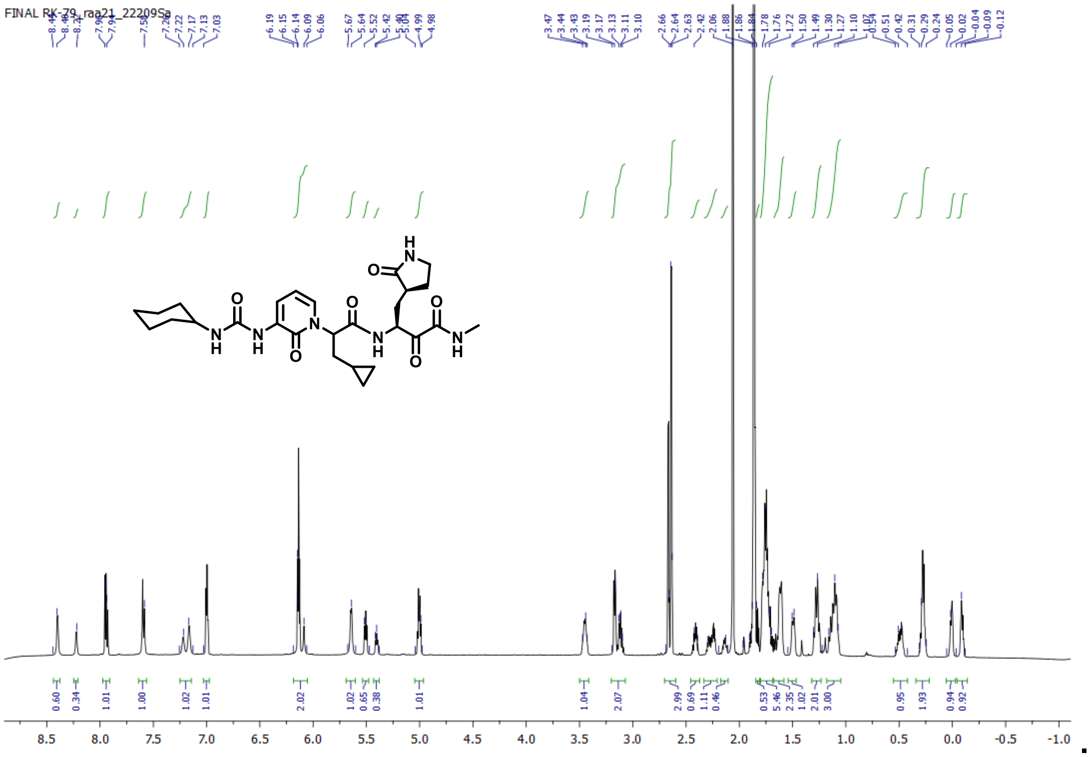

^{13}C-NMR (125 MHz, CD_3CN) of 16c.

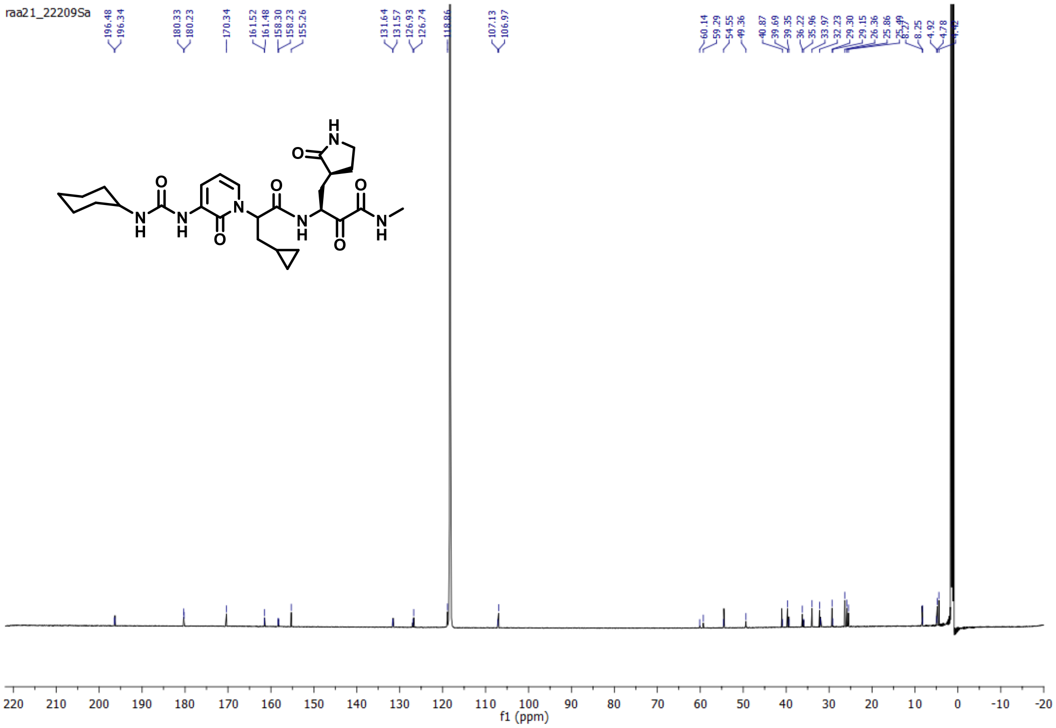

^{1}H-NMR (500 MHz, CD_3CN) of 16d.

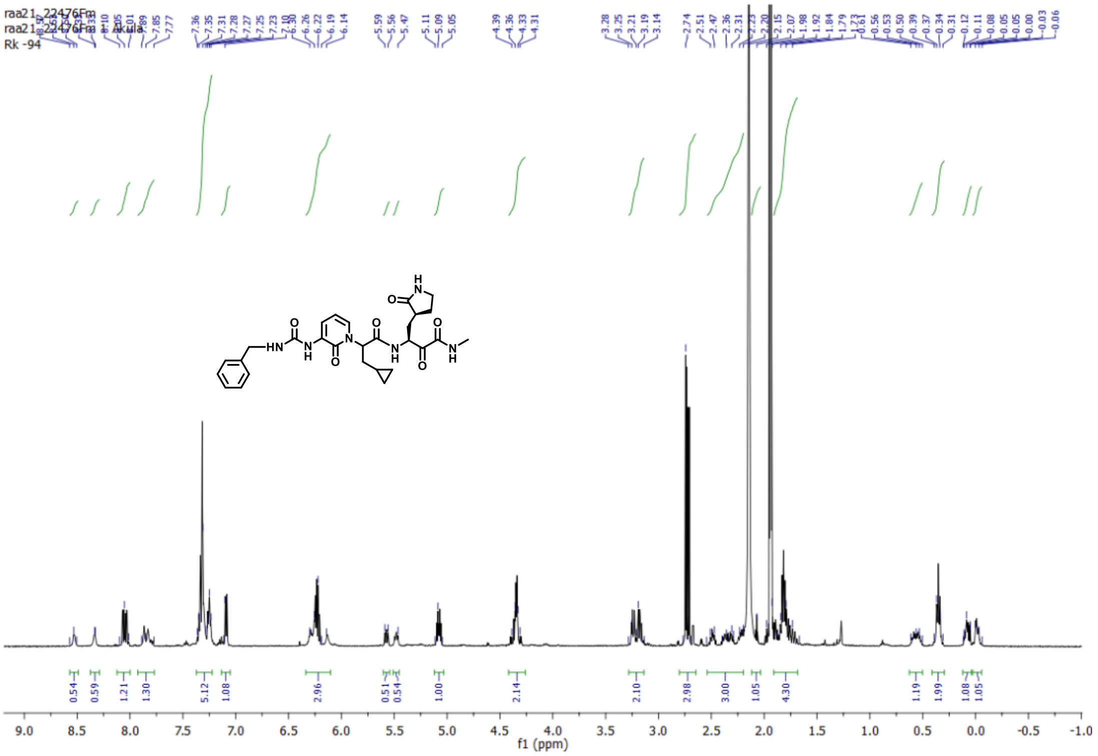

^{13}C-NMR (125 MHz, CD_3CN) of 16d.

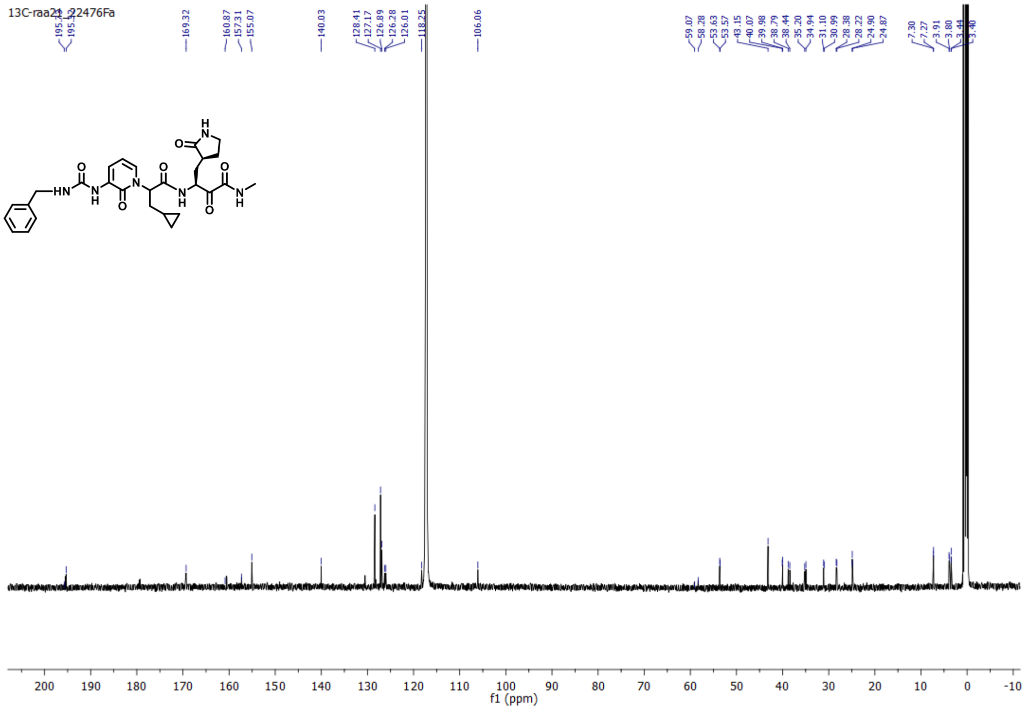

^{1}H-NMR (500 MHz, CD_3CN) of 16e.

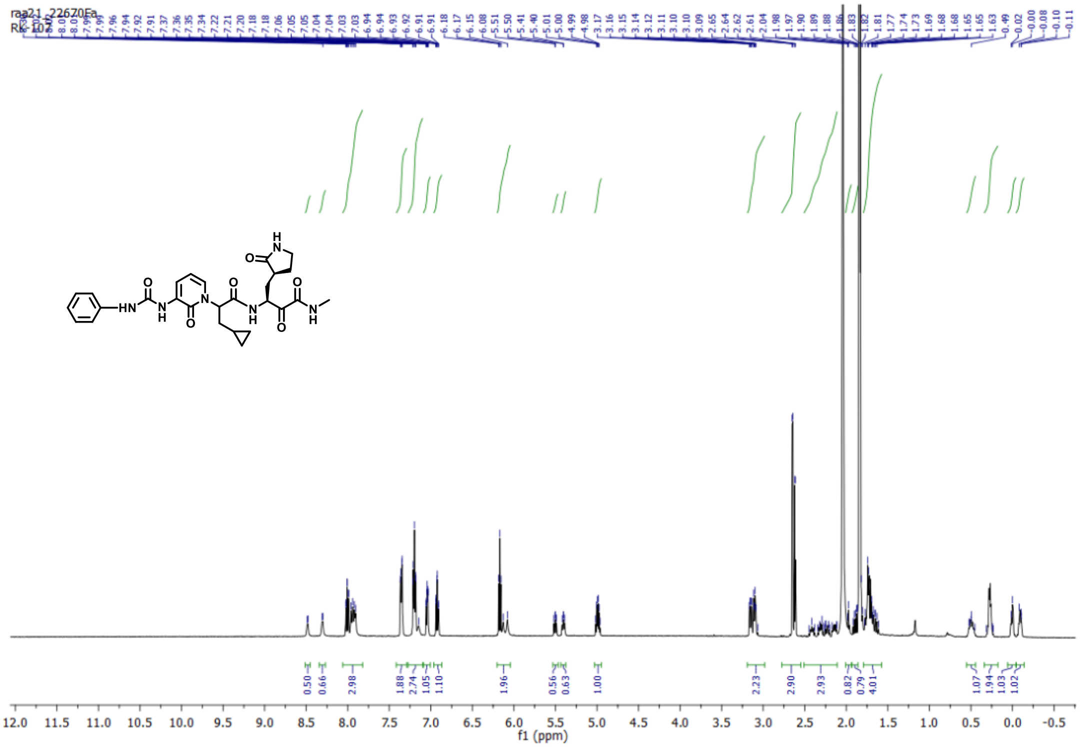

^{13}C-NMR (125 MHz, CD_3CN) of 16e.

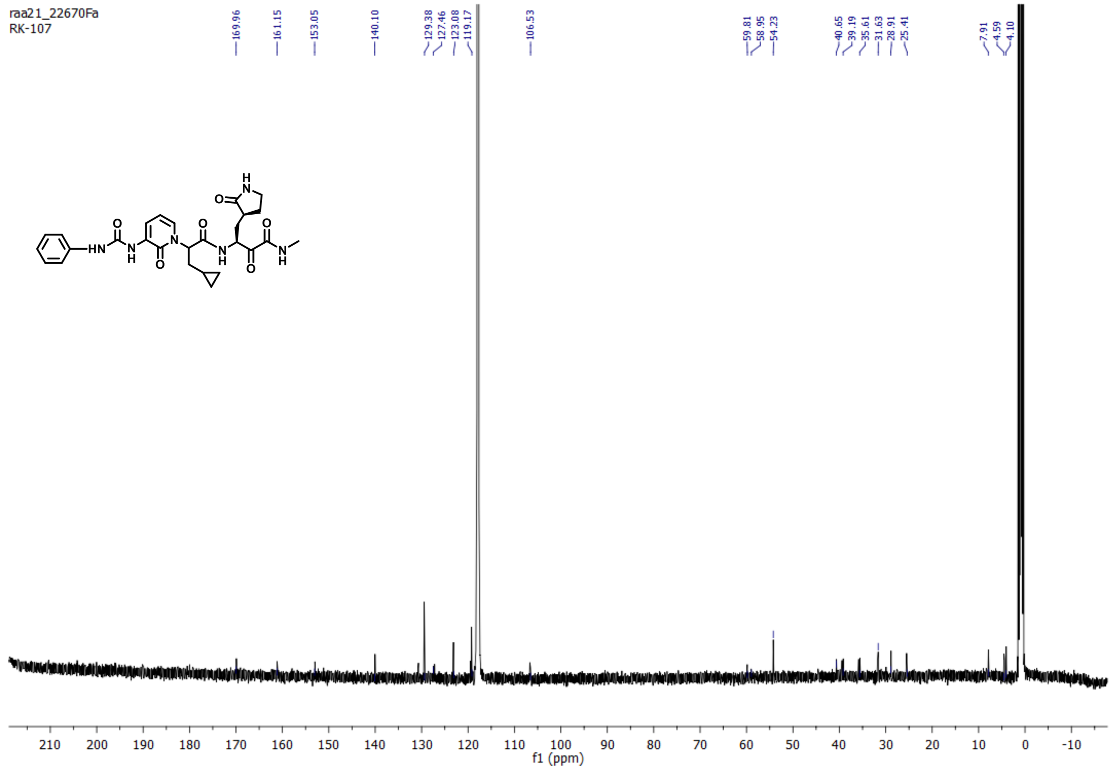

^{1}H-NMR (500 MHz, CD_3CN) of 17.

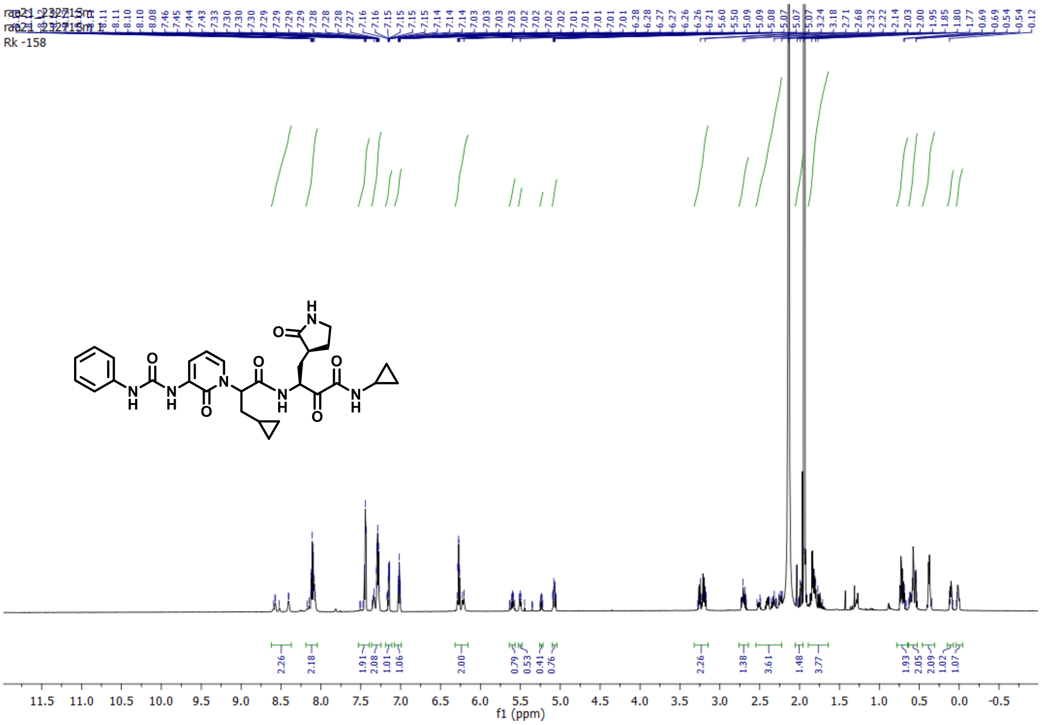

^{13}C-NMR (125 MHz, CD_3CN) of 17.

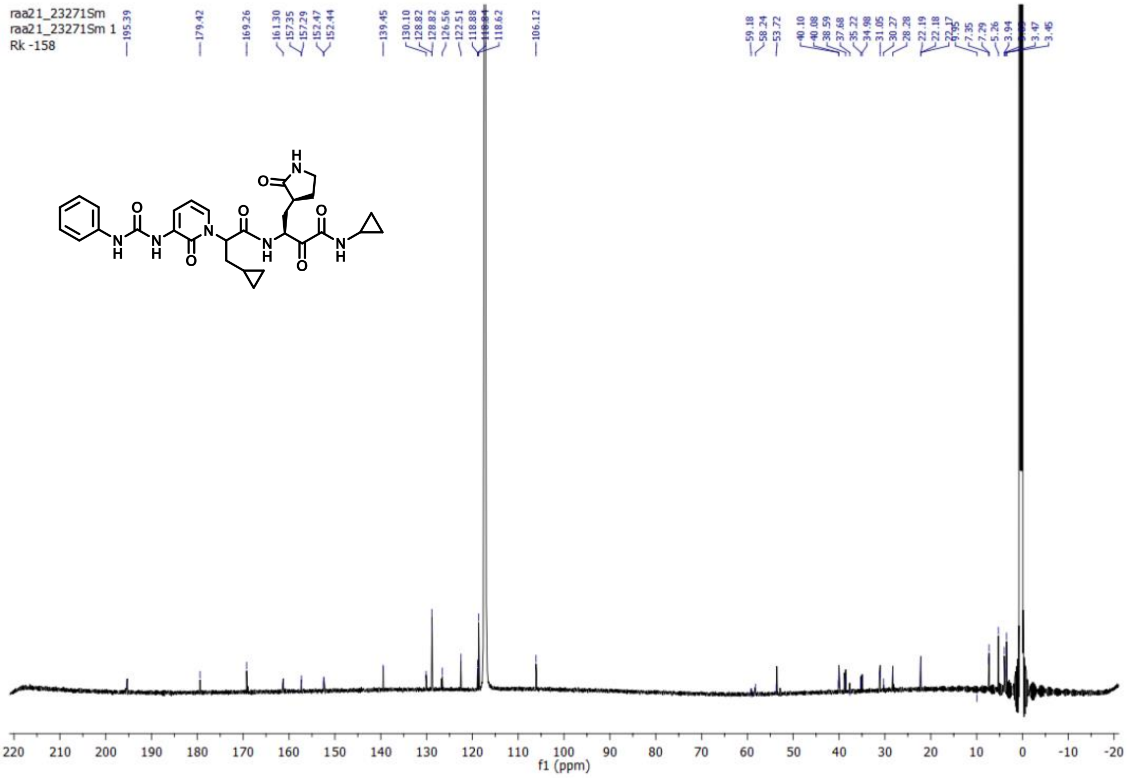

HPLC & SFC Traces

Compound-6a

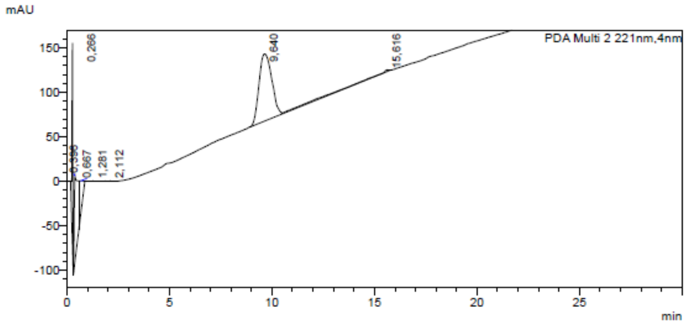

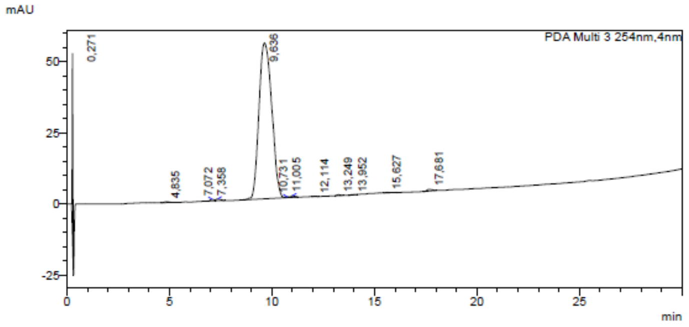

Compound-6b

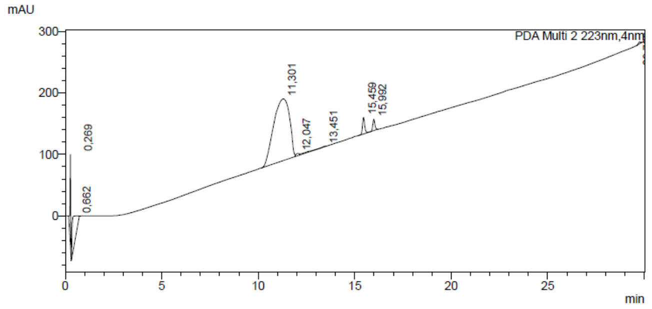

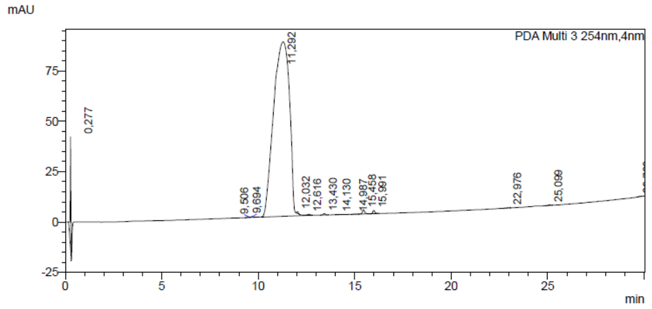

Compound-6c

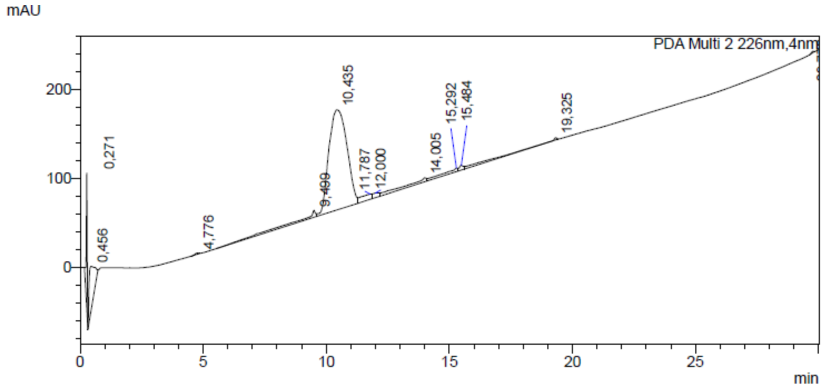

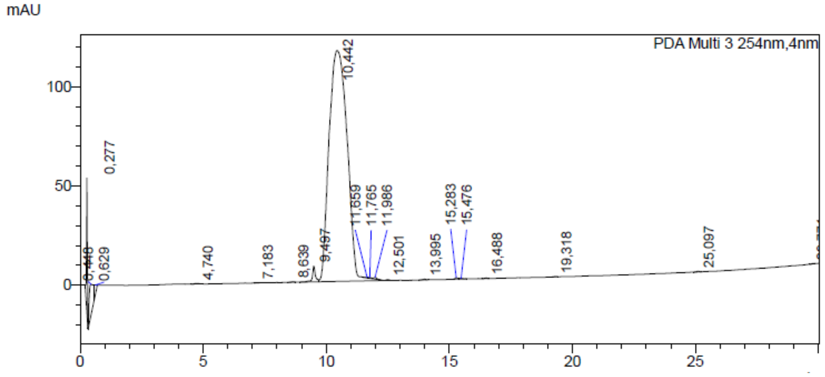

Compound-6d

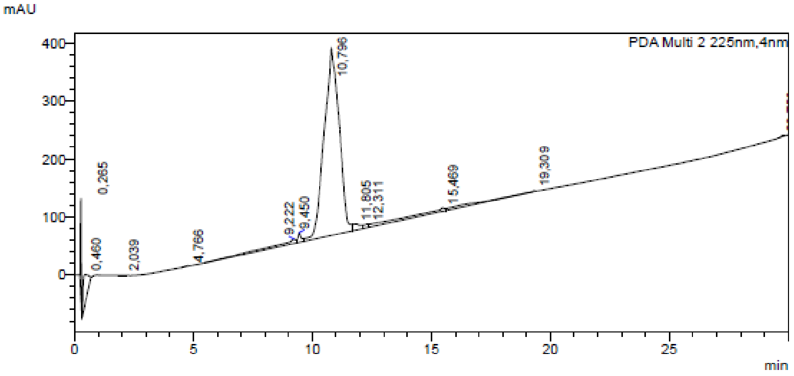

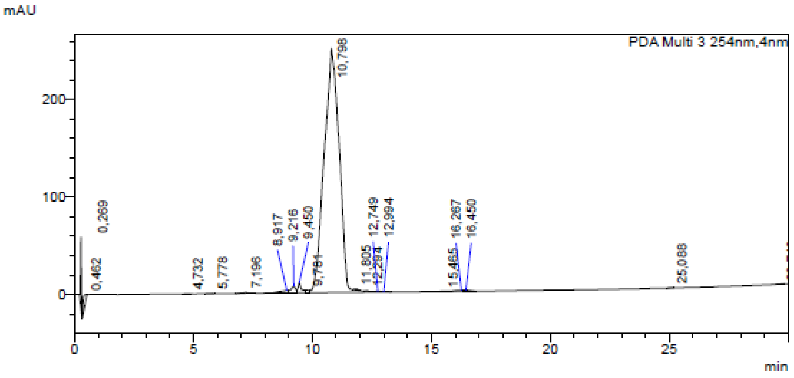

Compound-9a

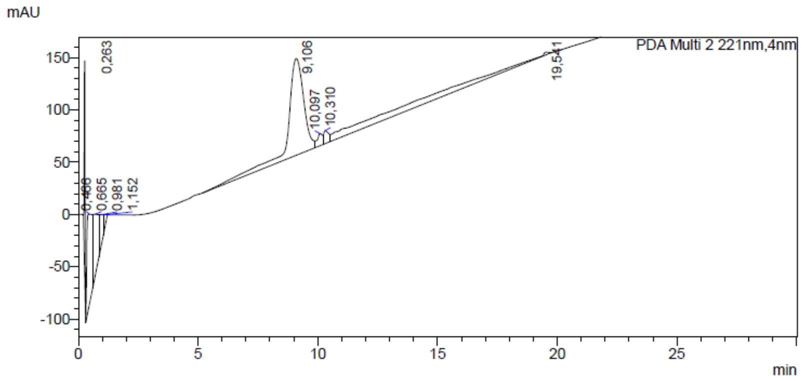

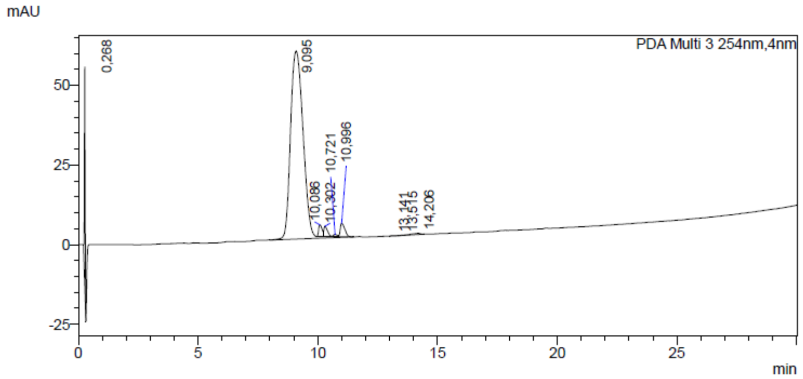

Compound-9b (SFC traces)

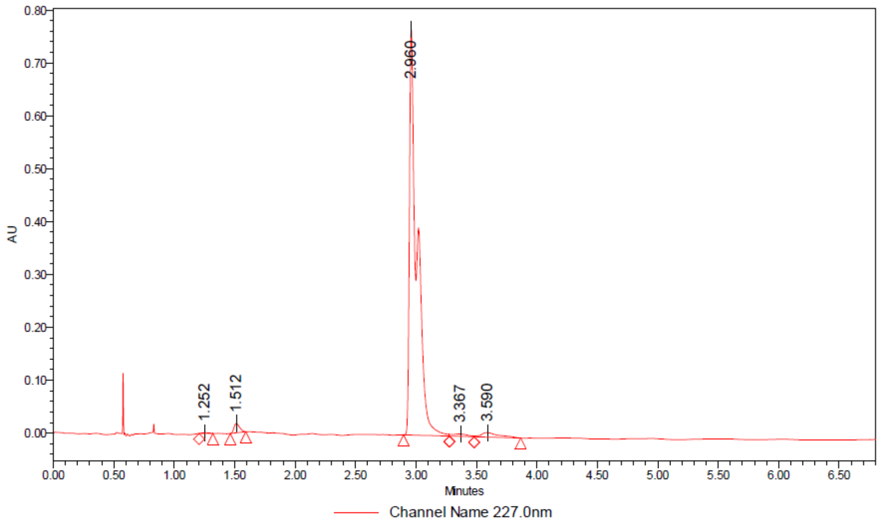

	RT	% Area	Area (µV*sec)
1	1.252	0.19	6538
2	1.512	1.64	57450
3	2.960	94.51	3318682
4	3.367	1.15	40470
5	3.590	2.52	88449

Compound-9b (HPLC traces): 98.1%

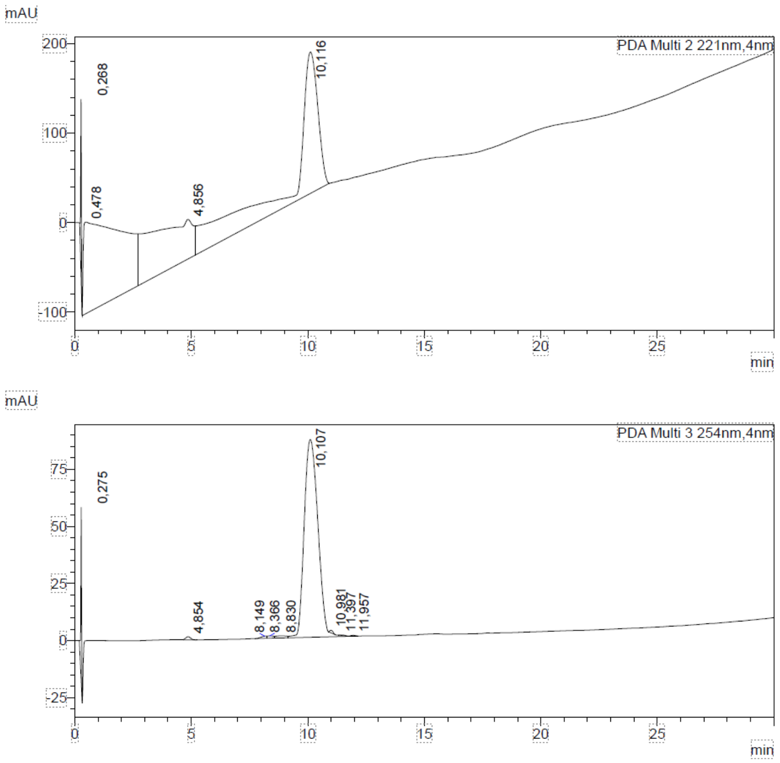

Compound-9c (SFC traces)

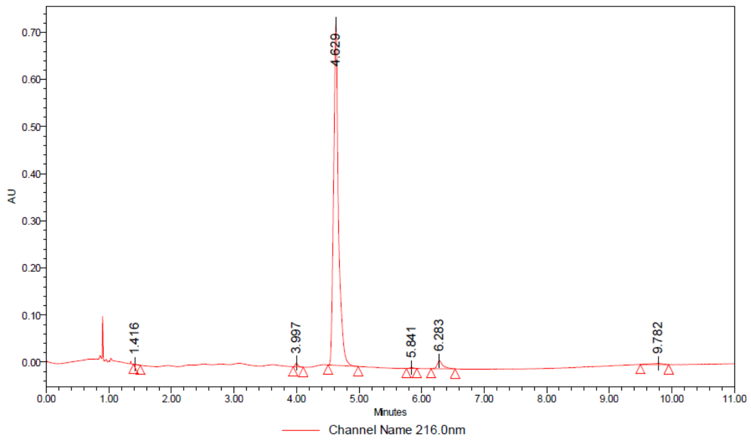

	RT	% Area	Area (µV*sec)
1	1.416	0.06	2576
2	3.997	0.58	23864
3	4.629	95.59	3921472
4	5.841	0.24	9668
5	6.283	2.65	108629
6	9.782	0.89	36374

Compound-9d (SFC traces)

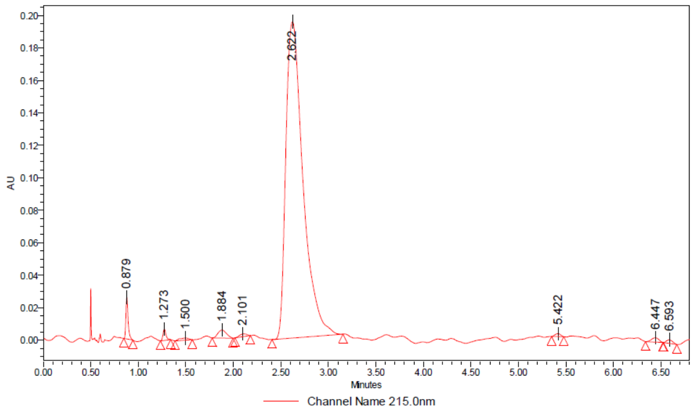

	RT	% Area	Area (μV*sec)
1	0.879	1.62	41787
2	1.273	0.60	15394
3	1.500	0.35	9057
4	1.884	1.21	31367
5	2.101	0.34	8681
6	2.622	94.66	2446980

	RT	% Area	Area (μV*sec)
7	5.422	0.29	7421
8	6.447	0.56	14530
9	6.593	0.38	9893

Compound-12a

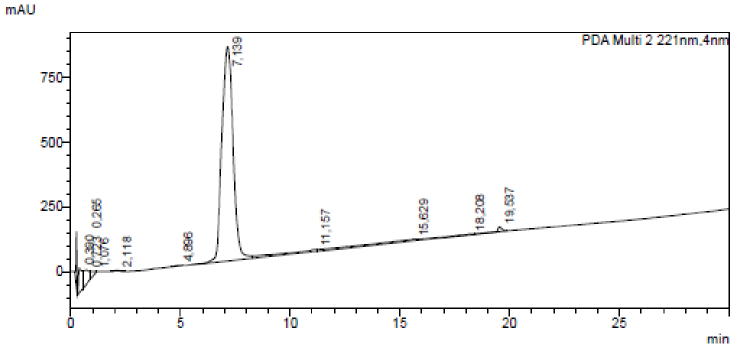

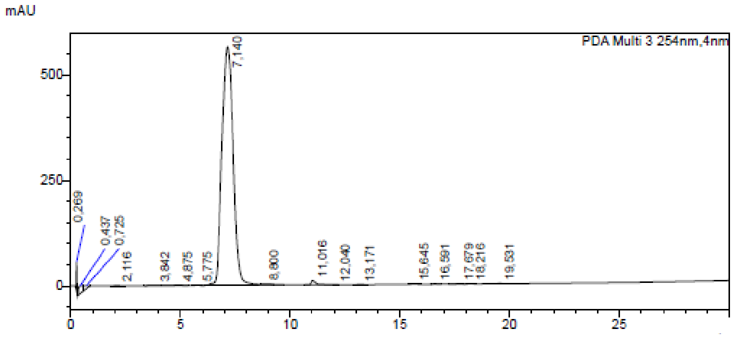

Compound-12b

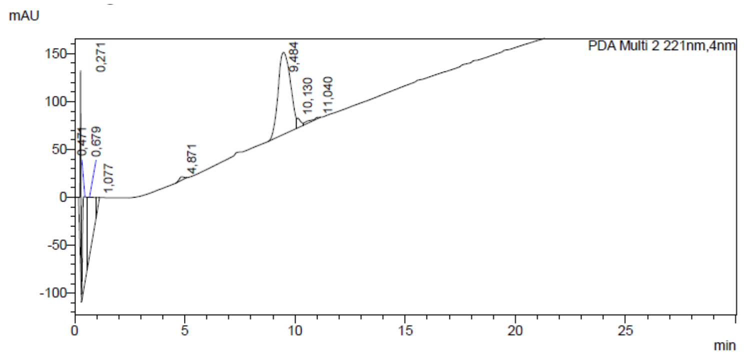

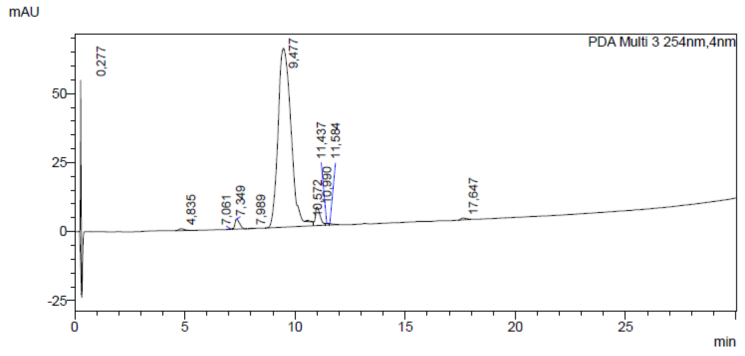

Compound-12c

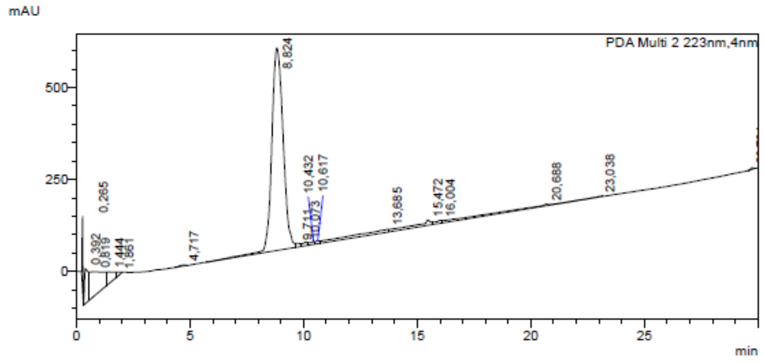

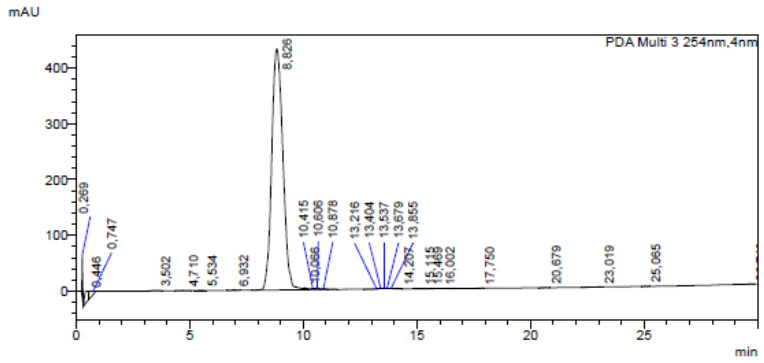

Compound-12d

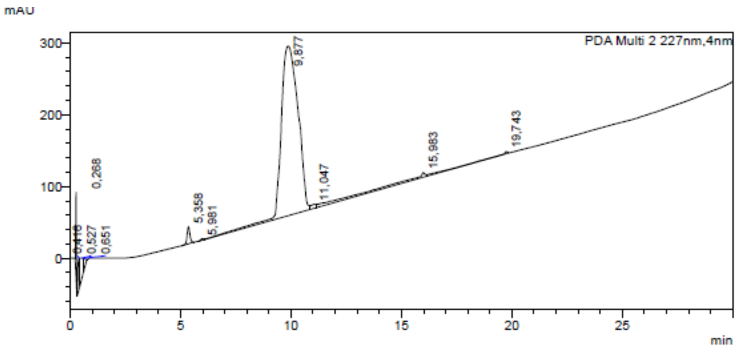

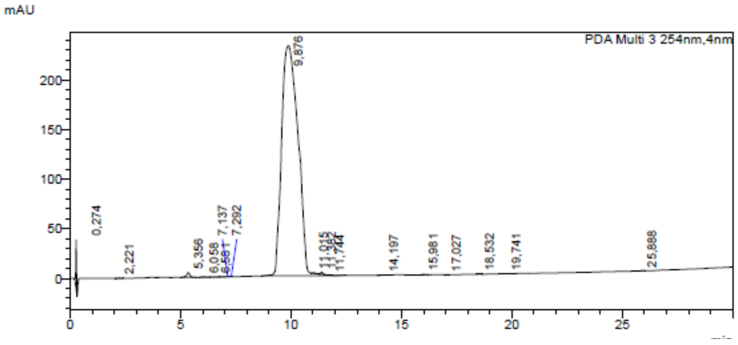

Compound-12e

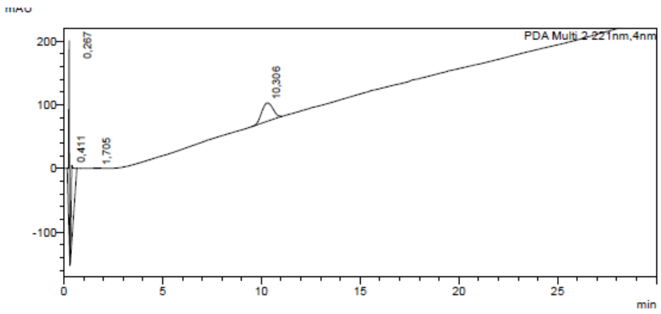

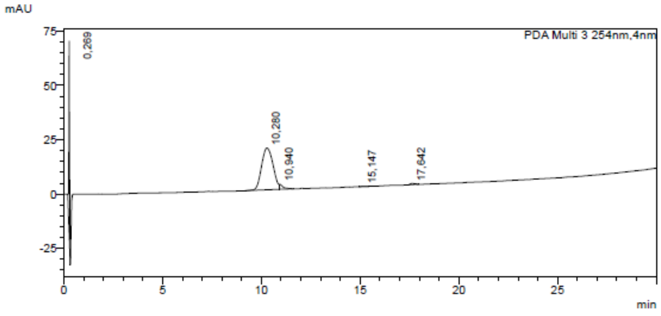

Compound-12f (LCMS Traces)

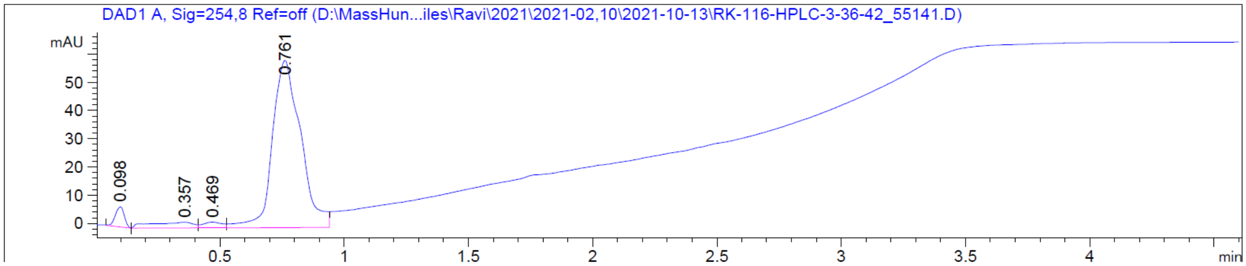

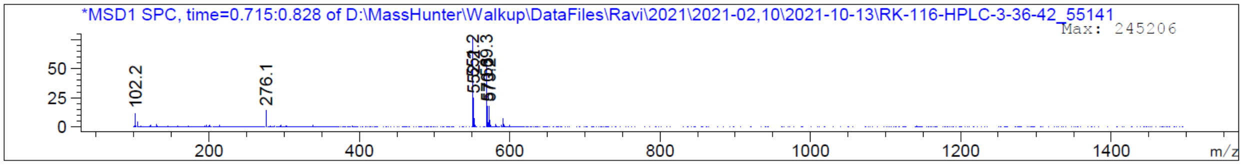

Compound-12g

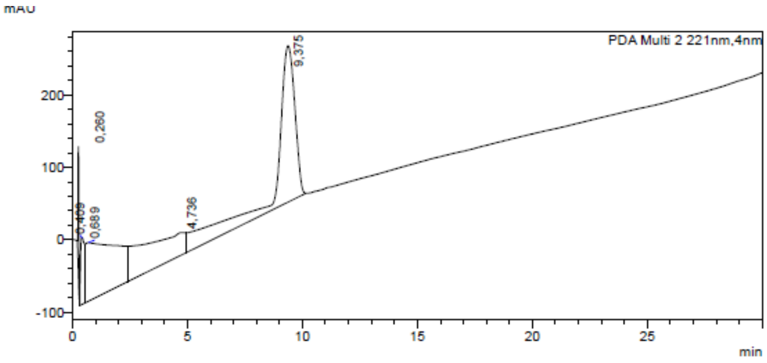

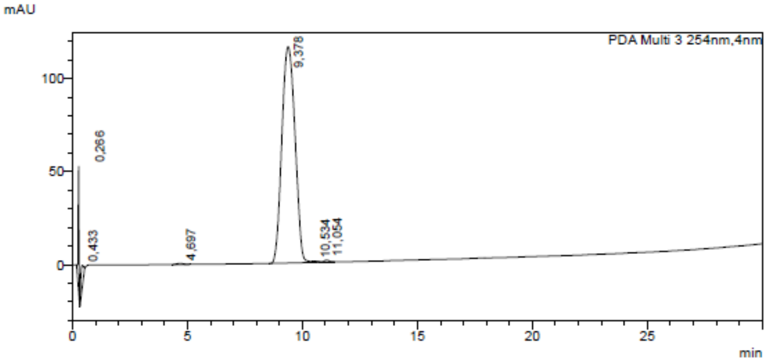

Compound-12h

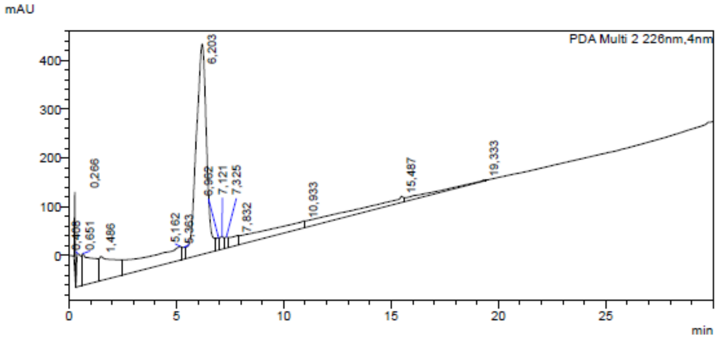

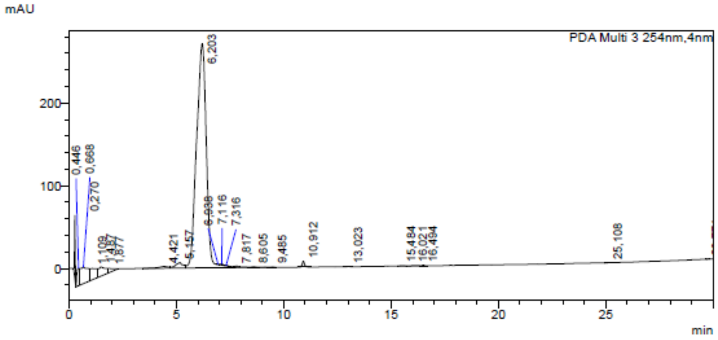

Compound-16a

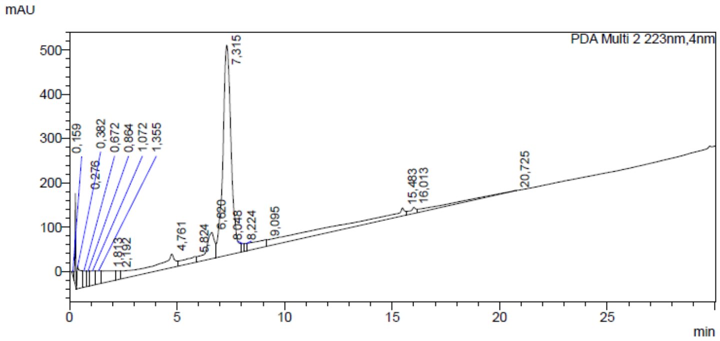

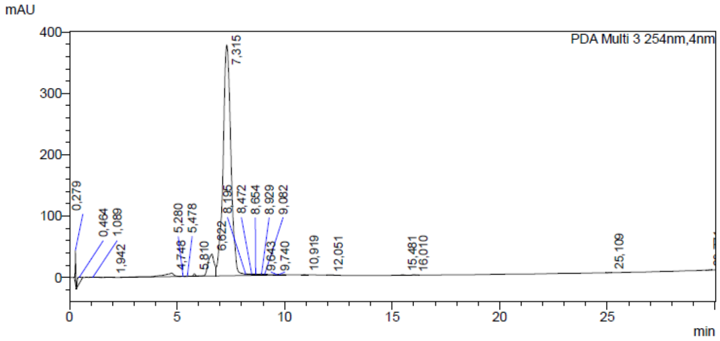

Compound-16b

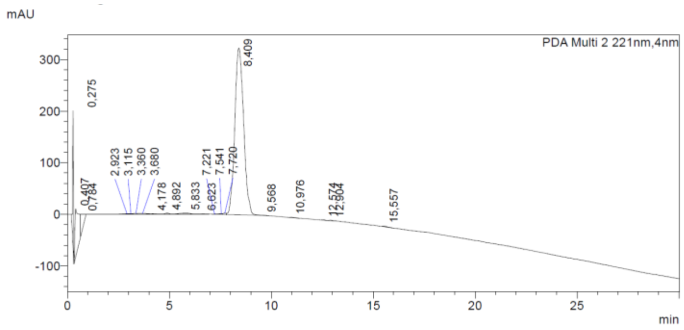

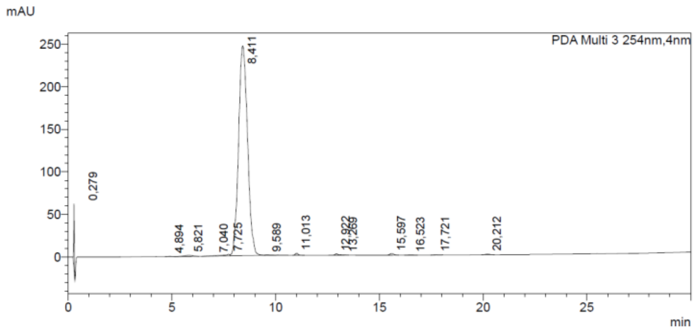

Compound-16c

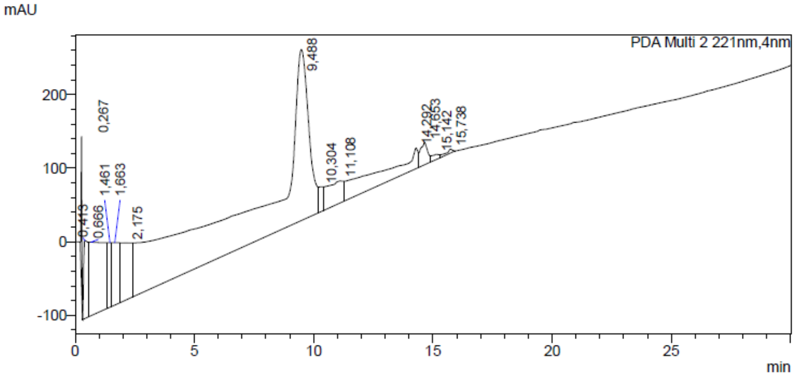

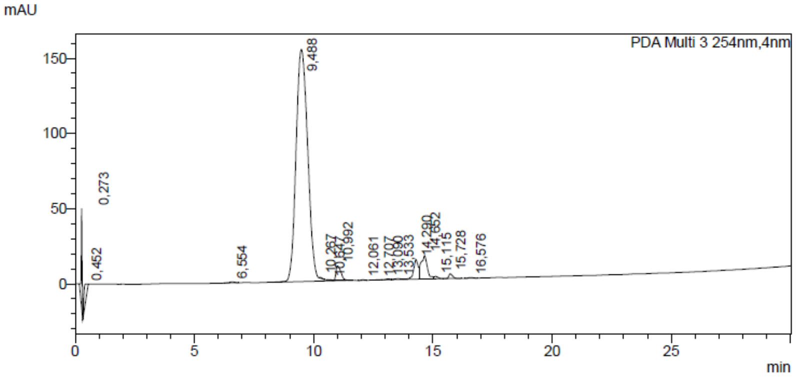

Compound-16d

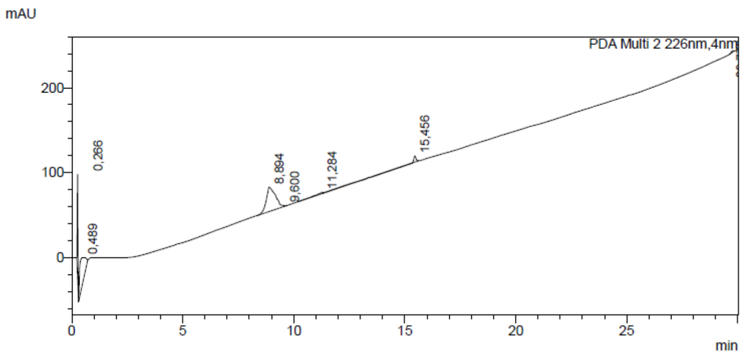

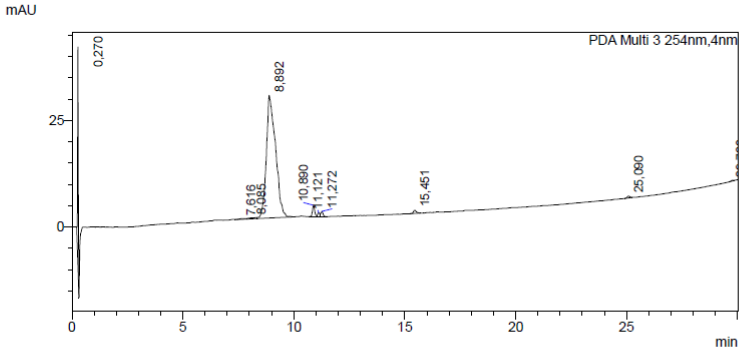

Compound-16e

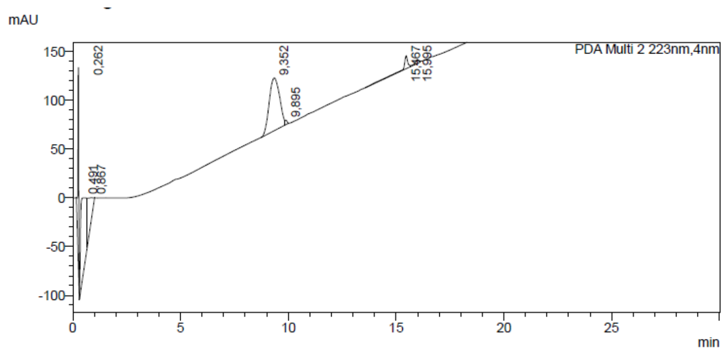

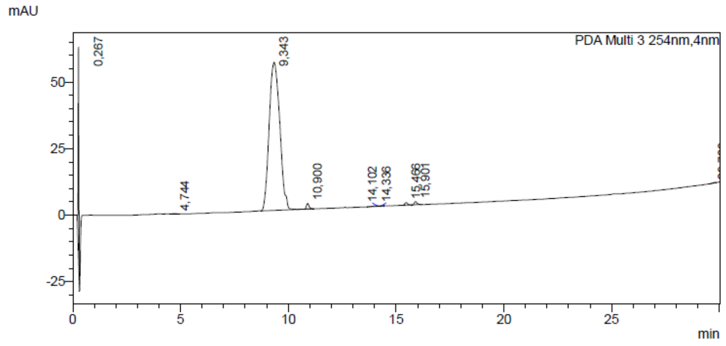

Compound-17

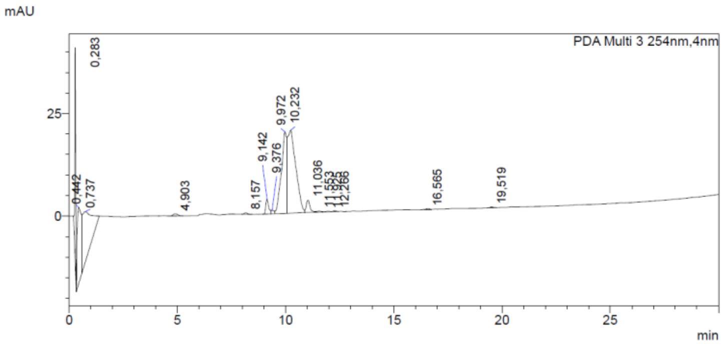

mAU
PDA Multi 3 254nm,4nm
0,283
0,442
0,737
4,903
8,157
9,142
9,376
9,972
10,232
11,036
16,565
19,519
25
0
0
5
10
15
20
25
min

Reference:

(1) Cooper, M. S.; Zhang, L.; Ibrahim, M.; Zhang, K.; Sun, X.; Roske, J.; Gohl, M.; Bronstrup, M.; Cowell, J. K.; Sauerhering, L.; et al. Diastereomeric Resolution Yields Highly Potent Inhibitor of SARS-CoV-2 Main Protease. *J Med Chem* **2022**, *65* (19), 13328-13342. DOI: 10.1021/acs.jmedchem.2c01131 From NLM Medline.

(2) Weiss, M. S.; Hilgenfeld, R. On the use of the merging R factor as a quality indicator for X-ray data. *J. Appl. Crystallogr.* **1997**, *30* (2), 203-205.

(3) Karplus, P. A.; Diederichs, K. Linking crystallographic model and data quality. *Science* **2012**, *336* (6084), 1030-1033.

(4) Chen, V. B.; Arendall, W. B.; Headd, J. J.; Keedy, D. A.; Immormino, R. M.; Kapral, G. J.; Murray, L. W.; Richardson, J. S.; Richardson, D. C. MolProbity: all-atom structure validation for macromolecular crystallography. *Acta Crystallogr. Sect. D. Biol. Crystallogr.* **2010**, *66* (1), 12-21.
